# The genetic regulatory network centered on *Pto-Wuschela* and its targets involved in wood formation revealed by association studies

**DOI:** 10.1038/srep16507

**Published:** 2015-11-09

**Authors:** Xiaohui Yang, Zunzheng Wei, Qingzhang Du, Jinhui Chen, Qingshi Wang, Mingyang Quan, Yuepeng Song, Jianbo Xie, Deqiang Zhang

**Affiliations:** 1 National Engineering Laboratory for Tree Breeding, College of Biological Sciences and Technology, Beijing Forestry University, No. 35, Qinghua East Road, Beijing 100083, P. R. China; 2 Key Laboratory of Genetics and Breeding in Forest Trees and Ornamental Plants, Ministry of Education, College of Biological Sciences and Technology, Beijing Forestry University, No. 35, Qinghua East Road, Beijing 100083, P. R. China; 3 Beijing Vegetable Research Center, Beijing Academy of Agriculture and Forestry Sciences, Key Laboratory of Biology and Genetic Improvement of Horticultural Crops (North China), Key Laboratory of Urban Agriculture (North), Ministry of Agriculture, No. 50, Zhanghua Road, Beijing 10097, China

## Abstract

Transcription factors (TFs) regulate gene expression and can strongly affect phenotypes. However, few studies have examined TF variants and TF interactions with their targets in plants. Here, we used genetic association in 435 unrelated individuals of *Populus tomentosa* to explore the variants in *Pto-Wuschela* and its targets to decipher the genetic regulatory network of *Pto-Wuschela*. Our bioinformatics and co-expression analysis identified 53 genes with the motif TCACGTGA as putative targets of *Pto-Wuschela*. Single-marker association analysis showed that *Pto-Wuschela* was associated with wood properties, which is in agreement with the observation that it has higher expression in stem vascular tissues in *Populus*. Also, SNPs in the 53 targets were associated with growth or wood properties under additive or dominance effects, suggesting these genes and *Pto-Wuschela* may act in the same genetic pathways that affect variation in these quantitative traits. Epistasis analysis indicated that 75.5% of these genes directly or indirectly interacted *Pto-Wuschela*, revealing the coordinated genetic regulatory network formed by *Pto-Wuschela* and its targets. Thus, our study provides an alternative method for dissection of the interactions between a TF and its targets, which will strength our understanding of the regulatory roles of TFs in complex traits in plants.

Perennial plants provide vital commercial products and raw materials for renewable energy. The characteristics of long-lived, large perennial plants, many of which have relatively large genomes, are determined by developmental processes, such as growth and wood formation. Regulation of these processes involves elaborate, coordinated dynamic networks of DNAs, RNAs, proteins, and metabolic intermediates. Transcription factors (TFs), also called *trans*-regulatory elements, function as major participants in genetic regulatory networks (GRNs) and regulate gene expression by binding to *cis*-regulatory elements in a sequence-specific manner. The interactions between TFs and their target genes induce spatiotemporal patterns of gene expression and play essential roles in plant growth and development. Establishing the architecture of GRNs will provide insights into the mechanisms of gene regulation and phenotypic variance in various organisms. For example, yeast (*Saccharomyces cerevisiae*) has a fully connected, circular GRN, in which nine TFs regulate the expression of approximately 800 cell-cycle genes^1^. In addition, some stage-specific TFs regulate the expression of other TFs that act in the next stage of the cell cycle^1^. In animals, inactivation of one hub gene that genetically interacts with many diverse genes with various molecular functions can enhance the loss-of-function phenotype resulting from mutation of the interacting genes. This depends on the interacting genes that are altered in combination^2^. In herbaceous plants, previous work has focused on many GRNs centered on TFs that act in different growth and developmental processes and shown that the GRNs in stem cells decipher the vital elements involved in stem cell maintenance and differentiation^3^. Understanding the GRNs formed by these TFs will therefore provide key information to harvest the fruits of essential research on growth and development, as well as disease.

GRNs can be deciphered by several approaches. For example, in yeast, the cell cycle GRN was constructed by genome-wide location and expression analysis^2^. However, identification of genetic interactions in multicellular organisms, except *C. elegans*, remains laborious. Several studies used RNAi to identify genetic interactions in cultured mammalian or fly cells, but this approach turned out to be inefficient, and did not entirely clarify whether the interactions identified in single cells also function in whole organisms^4^,^5^. Therefore, subsequent work has exploited several methods to identify genetic interactions. First, predicting further interactions based on existing genetic interactions found that ~20% of the genes with a common partner likely also interact with each other^6^. Second, genetic interactions can be predicted by integration of other genomic datasets, since proteins that share common functions likely interact with each other^7^,^8^. Third, genetic interactions identified in one species could be used to predict interactions in another species. For example, protein–protein interactions in humans can be predicted using data from model organisms such as fly, yeast, and worms^9^.

For elucidation of GRNs in plants, chromatin immunoprecipitation (ChIP) followed by DNA microarray analysis (ChIP-chip), detected target genes of the WUSCHEL TF in stem cells in *Arabidopsis thaliana*^10^. Liu *et al.* (2015) first used ChIP-seq to identify genome-wide targets of the ARBORKNOX1 TF and found that this TF is prone to target evolutionarily conserved genes in *Populus*^11^. These studies broadened our understanding of specific TFs and their targets in plants. However, the molecular technology used in animals and annual plants is not sufficient to study the complex GRNs acting in natural populations of trees, because GRNs not only include the interactions of the TF and its targets, but also include the interactions of targets with other targets. Fortunately, linkage disequilibrium (LD)-based association mapping provides an alternative method for annotating gene function and identifying GRNs^12^. Indeed, candidate gene-based single-nucleotide polymorphism (SNP) association analysis, with additive and dominant effects, has been widely used to detect significant alleles affecting growth and wood properties in tree species^13^,^14^. Previous studies indicated that association studies could be used as an alternative, powerful method for deciphering gene function and constructing GRNs^15^. Moreover, the epistatic effects detected by SNP-based association can identify interactions between genes, thus providing a powerful approach for deciphering GRNs in populations^16^.

*WUSCHEL* is an important TF involved in shoot apical meristem initiation and maintenance^10^. The function of WUSCHEL and its targets has been explored in Arabidopsis, but in forest trees, which significantly differ from herbaceous plants, its function may differ. In the present study, we integrated transcriptome profiling and SNP-based association to decipher the GRN of *WUSCHEL* in *Populus tomentosa*, an important commercial plantation tree species for pulp and timber in northern China. Here, we identified 4,762 SNPs from *Pto-Wuschela* and its 53 putative target genes in 435 unrelated individuals of *P. tomentosa*. Particularly, we used epistatic effects, which dissect these interactions of different SNPs, to explore the GRN of *Pto-Wuschela* and its target genes for tree growth and wood properties. Therefore, we not only clarify the putative roles of *Pto-Wuschela* and its target genes, but also provide insights into the GRN centered on *Pto-Wuschela* in *Populus*.

## Results

### Identification and tissue-specific expression of *Pto-Wuschela*

**Figure 1:**
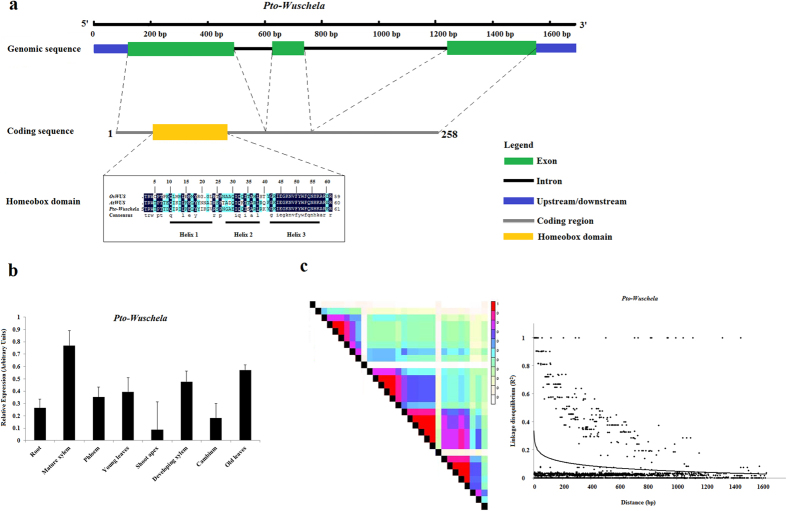
Summary of *Pto-Wuschela*. (**a**) Gene structure of *Pto-Wuschela*. *Pto-Wuschela* has three exons and two introns. The coding sequence contains a homeobox domain, which specified *Pto-Wuschela* as a WOX gene family member. The homeobox domain has three helixes: Helix 1 (10–23), Helix 2 (28–38), and Helix 3 (42–57). (**b**) Expression of *Pto-Wuschela*. The relative expression levels were measured by RT-qPCR in eight tissues, including root, mature xylem, phloem, young leaves, shoot apex, developing xylem, cambium, and old leaves. *Actin* was used as the internal control. (**c**) Decline of linkage disequilibrium within *Pto-Wushchela*. Pairwise correlations between SNPs are plotted against physical distance (bp) between SNPs. The curves describe the decay of r^2^ (Er2). Linkage disequilibrium of *Pto-Wushchela* decays within 300 bp.

We identified a full-length *Pto-Wuschela* clone by reverse-transcription polymerase chain reaction (RT-PCR) from a cDNA library constructed from RNA isolated from the cambium zone of *P. tomentosa*. The cDNA of *Pto-Wuschela* is 1,042 bp in length with a coding region of 777 bp encoding 258 amino acids (aa), flanked by a 123 bp 5′-untranslated region (UTR) and a 134 bp 3′-UTR. Amino acid sequence analysis showed Pto-Wuschela has 77% amino acid sequence identity with AtWUS, and 24.15% identity with OsWUS. Pto-Wuschela has a typical homeobox domain at aa 33–95 (Fig. 1a), with similarities of 75.81% and 64.52% to AtWUS and OsWUS, respectively. The homeobox domain has three helixes and Helix 3 showed 100% conservation between AtWUS and Pto-Wuschela.

Reverse transcription quantitative polymerase chain reaction (RT-qPCR) using gene-specific primers was performed in eight different tissues and organs to determine whether *Pto-Wuschela* shows tissue-specific expression. We observed that *Pto-Wuschela* expression occurred in all organs with different abundances (Fig. 1b). *Pto-Wuschela* had a higher transcription level in mature xylem (0.77), followed by old leaves (0.57) and developing xylem (0.476). Low transcript abundances were observed in cambium and shoot apex (0.18 and 0.09, respectively). The RT-qPCR results showed that *Pto-Wuschela* may play important roles in vascular tissue formation and development.

### Identification and isolation of Pto-Wuschela target genes

**Figure 2:**
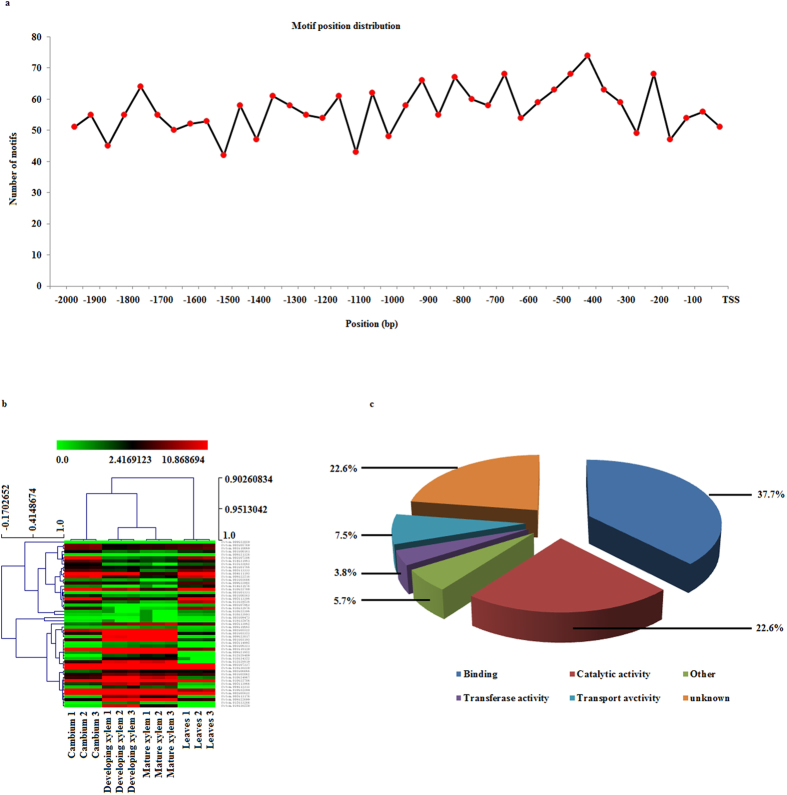
Summary of 53 selected candidate genes. (**a**) motif distribution of 53 selected genes. The promoter regions of the selected genes were collected and the motifs were located in these sequences. The most abundant regions containing the motifs were 300 to 600 bp and 650 to 950 bp upstream of the transcription start site. (**b**) transcript profiling of the 53 genes. Expression pattern of the 53 candidate genes were measured in cambium, developing xylem, mature xylem, and leaves. (**c**) gene ontology analysis of selected genes. These genes were annotated into six categories: binding, catalytic activity, transferase activity, transport activity, others, and unknown.

To detect the genes regulated by *Pto-Wuschela* at the whole-genome scale, we searched the promoter regions of all genes in *P. tomentosa* with the specific motif “TCACGTGA” and its alternative core motif “TAAT” using bioinformatics methods, detecting 318 genes. Then, we used transcriptome profiling datasets to select genes whose patterns of expression showed a strong correlation with *Pto-Wuschela* expression. In total, we identified 25 negatively (r < −0.8) and 28 positively (r > 0.8) correlated genes (Table S1). We analyzed motifs in the 53 targeted genes and found that the regions containing the most motifs were 300–600 bp and 650–950 bp upstream of the TSS, accounting for 17.03% and 16.50% of the total motifs, respectively (Fig. 2a). The transcript profiling data showed that the selected genes have similar expression patterns in developing xylem and mature xylem, compared with cambium and leaves (Fig. 2b). Genes encoding a tetratricopeptide repeat (TPR)-like superfamily protein, an acyl-CoA dehydrogenase-related protein, and auxin response factor 11 (ARF11) were more abundant in cambium than that in the other three tissues. These genes were annotated with JGI and TAIR gene models (Table S1). Of these genes, 37.7% encode proteins with predicted binding functions and 22.6% with predicted catalytic activity, indicating that these factors may activate or repress many more downstream genes. Only 3.8% of the genes encoded proteins involved in transferase activity (Fig. 2c) and 22.6% had unknown functions. Annotation of these genes may provide important information for deciphering the GRN centered on *Pto-Wuschela* in perennial plants.

### SNP identification and linkage disequilibrium in *Pto-Wuschela* and its target genes

To determine the intraspecific nucleotide diversity of *Pto-Wuschela*, we directly sequenced *Pto-Wuschela* in 40 unrelated individuals of *P. tomentosa* and obtained approximately 1,686 bp of genomic sequence. The 40 sequences of *Pto-Wuschela* were deposited in NCBI (http://www.ncbi.nlm.nih.gov/) under the accession numbers KR493384-KR493423. We also obtained sequences of the 53 putative target genes of *Pto-Wuschela*. Gene lengths varied from 2,315 to 15,130 bp, including 2,000 bp of promoter regions (Table S2). The genes were all deposited in NCBI under the accession numbers KT374071–KT374114.

To explore the nucleotide diversity of *Pto-Wuschela* and its target genes, we identified SNPs in *Pto-Wuschela* and its 53 putative target genes. In total, we identified 12,956 SNPs from these genes, with average frequencies of 1 SNP per 24 bp (Table S4). Of the SNPs detected, 36.7% were common SNPs (minor allele frequencies > 0.10). To detect important polymorphisms for the association analysis, we selected 19 common SNPs from *Pto-Wuschela* and 4,743 from the target genes for genotyping. The patterns of linkage disequilibrium (LD) decay of *Pto-Wuschela* were calculated and LD declined rapidly within 300 bp in *Pto-Wuschela* (*r*^*2*^ < 0.1, *P* < 0.001, Fig. 1c). We then analyzed the LD of the target genes and found that the level of LD between intragenic SNPs was much higher than between intergenic SNPs (Fig. S1).

### Significant associations with genotypic, additive, and dominant effects

To explore the effects of the SNPs in *Pto-Wuschela* and its target genes on phenotypes, we performed association tests between 4,762 SNPs from *Pto-Wuschela* and its target genes and ten phenotypic traits including the tree growth traits diameter at breast height (DBH), tree height (H), and stem volume (V), and the wood property traits hemicellulose content (HemC), holocellulose content (HolC), α-cellulose content, lignin content (LC), fiber length (FL), fiber width (FW), and microfiber angle (MFA), using a mixed linear model that includes population structure, in TASSEL 2.1. We identified 302 significant associations with 284 SNPs in *Pto-Wuschela* and its 53 target genes (*P* < 0.001); the percentage of variance explained by each SNP ranged from 0.118% (Potom.002G05522-SNP76 associated with DBH) to 15.15% (*Pto-Wuschela*-SNP3 associated with FW) (Table S3). Three unique SNPs from *Pto-Wuschela* (SNP3, SNP13, and SNP17) were significantly associated with three phenotypic traits, FW, FL, and H, suggesting that *Pto-Wuschela* has important roles in tree growth and wood properties (Table S3). The most significant association was between FW and *Pto-Wuschela*-SNP3 (*P* = 9.83E-07, R^2^ = 15.15%), which is located in the first exon, a synonymous substitution of CAA to CAG (Glutamine).

For SNPs in the putative target genes, we identified 299 significant associations with ten traits. For wood properties, we identified 87 significant associations, 51 of them with FW. For example, five SNPs in Potom.001G03606 (zinc knuckle (CCHC-type) family protein) were associated with FW, indicating its role in wood formation. For tree growth traits, 199 SNPs were closely linked to V, DBH, and H (Table S3). For example, 22 SNPs in Potom.008G22699 (multifunctional protein 2) were significantly associated with DBH, providing new evidence into the potential roles of Potom.008G22699 in tree growth and wood formation. Among the identified SNPs in targets, unsurprisingly, the SNPs in the Pto-Wuschela target sites were significantly associated with phenotypic traits. For example, the SNP Potom.001G03353-SNP143 (T > C), which was located in a Pto-Wuschela target site, was significantly associated with H (*P* = 4.44E-06).

To further dissect the genetic effect of SNPs in *Pto-Wuschela* and its target genes, we then used least squares tests, concentrating on additive and dominant effects, to identify associations with the ten quantitative traits. In total, 803 significant phenotype-genotype associations were identified as having additive or dominant effects (*P* ≤ 0.01, FDR < 0.1). Each trait was associated with 13 to 275 SNPs under additive or dominant effect models, indicating that these genes may play important roles in tree growth and wood formation (Table 1).

Significant SNPs under the additive model: Under the additive model, we identified 570 significant associations containing 377 unique SNPs from 46 genes (Table 1). Three SNPs from *Pto-Wuschela* (*Pto-Wuschela*-SNP11, *Pto-Wuschela*-SNP17, and *Pto-Wuschela*-SNP18) were associated with D, V, MFA, and FL with effects of 0.95, 0.00677, −1, and −0.04, respectively. Of these SNPs, *Pto-Wuschela*-SNP11, which was a synonymous mutation (TCA - TCC (serine)) located in the first exon, was associated with D and V, indicating the potential role of *Pto-Wuschela* in tree growth. The same situation was observed in *Pto-Wuschela*-SNP17 (third exon, synonymous mutation, AGT-AGC (serine)), which is significantly associated with FL, indicating that *Pto-Wuschela* may also be involved in wood fiber formation. Furthermore, 566 SNPs from 46 putative target genes were also associated with wood properties and tree growth under the additive model.

Significant SNPs under the dominant model: In total, we identified 232 significant associations with dominant effects, containing 179 SNPs across 10 phenotypic traits, with the effects of each SNP ranging from −34.2 to 19.3 (Table 1 and S4). Of these associations, 72 had negative effects and 161 had positive effects, indicating that the heterozygous genotypes of these SNP loci associated with higher phenotypic values compared with the homozygous genotypes. Among the 11 most significant associations, nine SNPs from three genes (one SNP from Potom.002G07963, seven SNPs from Potom.014G31981, and one SNP from Potom.013G29538) were associated with V, with the effects of each SNP ranging from −0.958 to 1.8. In these, Potom.002G07963, encoding a P-loop-containing nucleoside triphosphate hydrolase superfamily protein, was also associated with the growth trait DBH, and with the wood property traits MFA, HemC, HolC, and α-cellulose content. Potom.013G29538 was associated with H and V, and wood property traits FW, FL, HemC, HolC, LC, and α-cellulose content. Similarly, Potom.014G31981, whose function is unknown, is associated with DBH, V, MFA, FW, and α-cellulose content. Two SNPs in *Pto-Wuschela*, *Pto-Wuschela*-SNP3 and *Pto-Wuschela*-SNP14, were associated with MFA and HolC, respectively, indicating that Pto-Wuschela and its targets may be involved in the same processes of tree growth and wood formation.

In summary, 48 of the 53 putative target genes were detected in the single-locus association study. We found that 45 of the detected 48 putative target genes were associated with more than one wood property trait. For example, Potom.003G09473, which encodes cytokinin response factor 2, was associated with five wood property traits (FL, FW, HemC, HolC, and α-cellulose content). This gene also associated with two growth traits (DBH and V). For tree growth traits, 40 putative target genes were identified to be associated with more than one growth trait. For example, Potom.002G05769 (cyclin family protein) associated with DBH and V, as well as wood property traits FW and HemC. Meanwhile, we found that *Pto-Wuschela* was also associated with two traits of tree growth and four wood property traits, which suggests that *Pto-Wuschela* and these identified genes may participate in the same genetic pathways for complex quantitative traits.

### Significant SNP-SNP epistasis interactions

To analyze further the interaction between the TF and its targets, we conducted SNP-SNP association studies between SNPs from *Pto-Wuschela* and its putative target genes using the epiSNP package. In total, we detected 2,505 significant pairwise associations, including 1,592 SNP-SNP interactions associated with 10 phenotypic traits (*P* < 1.0E-02, FDR < 0.1, Table S5). Of the pairwise interactions, 23.4% (587) of the SNP-SNP interaction pairs representing 39 genes were associated with V, with *P-*values of 1.97E-06 to 1.0E-02, followed by HolC, which was associated with 15.00% (375) of the SNP-SNP pairs (8.05E-06 ≤ *P* ≤ 9.91E-03). Only 96 SNP-SNP interactions from 33 genes were associated with MFA (9.8E-03 ≤ *P* ≤ 3.3E-04) (Table 2). Among these associations, five SNPs from *Pto-Wuschela* interacted with 157 SNPs from 24 genes, and their SNP-SNP pairs were associated with all 10 growth and wood property traits.

**Figure 3:**
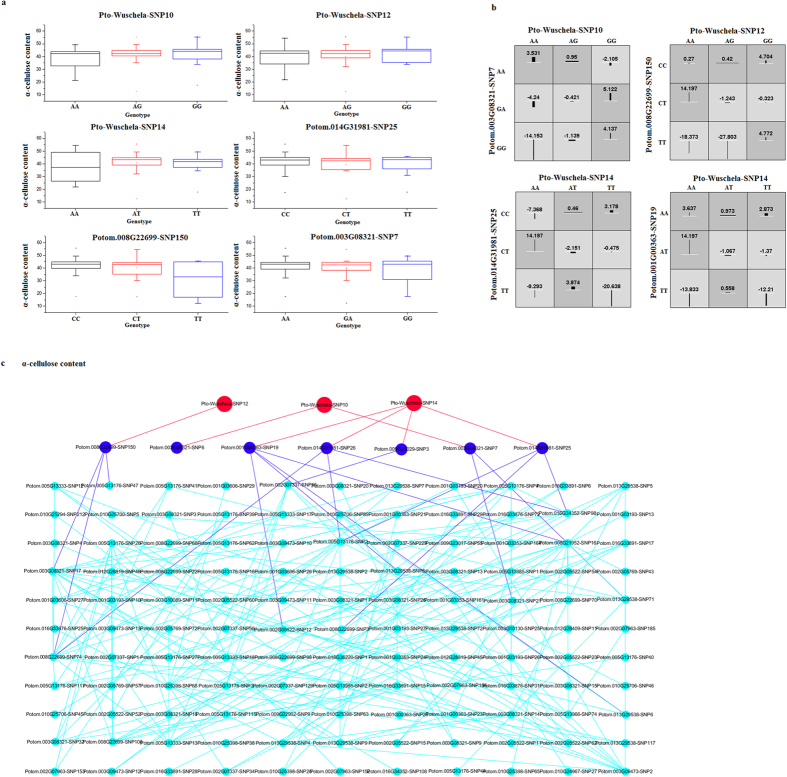
Phenotypic variation of single-locus genotypes, genotype-genotype combinations, and significant SNP-SNP pairs associated with α-cellulose contents. (**a**) box plots showing single-locus phenotypic variation of different genotypes of six SNPs. (**b**) square boxes show pairwise phenotypic variations of different genotype-genotype combinations. (**c**) SNP-SNP pairs identified to be associated with α-cellulose content. In total, 209 SNP-SNP pairs from 35 genes were identified. The SNP-SNP interactions were plotted by Cytoscape 3.2.1.

We found that the differences (phenotypic variation of different genotypes from the total average phenotype value) of genotype-genotype combinations were much higher than single-locus genotypes (Fig. 3a,b). For example, 209 SNP-SNP pairs (from 35 genes) associated with α-cellulose content (Fig. 3c). Taking *Pto-Wuschela*-SNP10 as an example, the difference between three genotypes (AA, AG, and GG) and the total average phenotypic value (40.14%) ranged from −1.59% (AG, 38.55%) to 1.66% (GG, 41.80%). For Potom.003G08321-SNP7, the homozygous genotype AA showed the highest α-cellulose content (41.21%, difference = 1.07%), while the homozygous genotype GG showed the lowest α-cellulose content (38.27%, difference = −1.87%). Strikingly, the difference between the average phenotypic value and different genotype-genotype combinations ranged from −14.153% (AA-GG) to 5.122% (GG-GA), which was remarkably higher than the phenotypic value of single locus genotypes. The same situation was also observed in other genotypic combinations (Fig. 3b). For example, the genotype-genotype combination of AA-CT for *Pto-Wuschela*-SNP12 × Potom.008G22699-SNP150 epistatic interaction had the highest α-cellulose content while AG-TT had the lowest α-cellulose content (Fig. 3b). The models of high- and low-value genotypes of single locus markedly differ across each of the different two-locus dimensions that were regarded as proof of epistatic interactions, also reveal gene-gene interactions between Pto-Wuschela and its targets.

Among the 209 different SNP-SNP pairs with epistasis effects associated with α-cellulose content, nine pairs were between SNPs from *Pto-Wuschela* and their putative targets (Table S5). Of these, four SNP-SNP pairs had A × A effects, two pairs had A × D effects, and three pairs had D × D effects (Table S5). For the A × A effect on α-cellulose content involving *Pto-Wuschela*-SNP14 and Potom.014G31981-SNP25 (located on chr5 and chr14, respectively), and the A-T gamete had the highest α-cellulose content while the T-T gamete had the lowest α-cellulose content (Table 3). This showed that the A and T alleles of *Pto-Wuschela*-SNP14 had significantly different effects when combined with the T allele of Potom.014G31981-SNP25. Also, *Pto-Wuschela*-SNP14 and Potom.014G31981-SNP25 did not have significant single-locus effects on α-cellulose content. The other three A × A effects were similar. For the A × D effect on SNP-SNP pair Potom.001G00363-SNP19 and *Pto-Wuschela*-SNP14, the A-AA allele-genotype combination had the highest α-cellulose content while the T-AA allele-genotype combination had the lowest α-cellulose content. For the D × D effects of *Pto-Wuschela*-SNP14 × Potom.014G31981-SNP25, representing the nine pairs of D × D effects involving the same trait, AA-CT had the highest α-cellulose content while AA-TT had the lowest α-cellulose content. For the remaining D × D effect, the genotype-genotype combination AA-TG for *Pto-Wuschela*-SNP14 × Potom.014G31981-SNP26 had the highest α-cellulose content while AA-GG had the lowest α-cellulose content (Table 3). These results showed that when *Pto-Wuschela*-SNP14 had the AA genotype, the three genotypes of Potom.014G31981-SNP26 had significantly different effects, indicating the epistatic interactions of Pto-Wuschela and its target genes.

**Figure 4:**
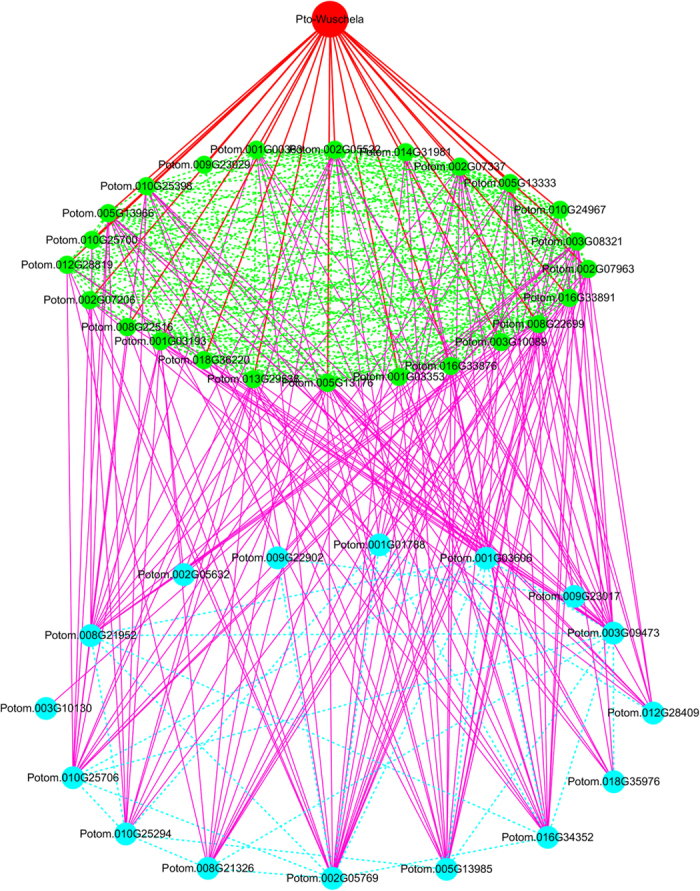
Hierarchical gene-gene interaction network formed by the epistatic interactions of *Pto-Wuschela* and candidate target genes. In total, 41 genes were detected in the pairwise analysis. *Pto-Wuschela* was placed at the top of the network, genes interacting directly with *Pto-Wuschela* were placed in the second layer and genes interacting indirectly with *Pto-Wuschela* were placed in the third layer. Red lines indicate interactions between the first and second layers. Purple lines indicate interactions between the second and third layers. Green lines indicate interactions between genes in the second layer. Light blue lines indicate interactions between genes in the third layer. Blue lines represent genes interacting within the third layer.

The SNP-SNP epistasis tests revealed a hierarchical gene-gene interaction network with *Pto-Wuschela* at the top, genes that interact directly with *Pto-Wuschela* in the second layer, and genes that interact indirectly with *Pto-Wuschela* in the third layer (Fig. 4). In total, this analysis identified 41 genes and these interactions included 24 genes, such as Potom.002G07206, Potom.002G07963, and Potom.010G24967 interacted with *Pto-Wuschela*. We constructed two networks using genes associated with HolC (375 epistasis interactions among 35 genes) and DBH (351 epistasis interactions among 38 genes) and we found substantial overlap (34 genes) in both networks (Fig. S4). We found that several genes in the second layer of the network associated with HolC (Fig. S2a), were in the third or fourth layer of the network associated with DBH (Fig. S2b), indicating the possibility that these genes play different roles in different genetic pathways.

## Discussion

Many crucial processes in long-lived perennial tree species, such as tree growth and wood formation, are controlled by complex GRNs, which include many kinds of interactions such as miRNA-mRNA, protein-DNA, lncRNA-mRNA, and lncRNA-miRNA interactions^17^. Of these, protein-DNA interactions, including TF-target interactions, have important functions in these networks. Here, we present a method combining transcription profiling and association analysis to detect the interaction of a TF and its targets, thus providing a new method for constructing a GRN.

Identification of Pto-Wuschela and its target genes was the first step in studying its TF-target interactions. TFs play their regulatory roles by binding to the promoter regions of their target genes. Also, the TF and its targets usually are co-expressed. Although some studies indicate that TF binding to a gene may not be sufficient to produce transcriptional variation^18^,^19^, the genes that show changes in expression may have important roles in biological processes. We compared the 60 amino acids of Pto-Wuschela and AtWUS (AT2G17950.1) that include the homeodoman, and found that the C-terminal helix-turn-helix, which interacts directly with the DNA motif, is 100% conserved between Arabidopsis and poplar, indicating that the specific motifs recognized by AtWUS are also likely recognized by Pto-Wuschela^20^. Therefore, we used the motif TCACGTGA and the alternative core element TAAT to select the candidate targets of Pto-Wuschela^10^. Then, we used transcriptome profiling, representing four different tissues, to calculate the Pearson’s product-moment correlations between expression of *Pto-Wuschela* and other genes. We selected as candidates the genes that had higher correlations with *Pto-Wuschela*, positively (r > 0.8) or negatively (r < −0.8). In total, we identified 53 genes, including Potom.003G09473, encoding cytokinin response factor 2, the Arabidopsis homolog of which was previously described as a target of AtWUS^10^. We found that several genes, including the homologs of *ATCLV1* (*Leucine-rich receptor-like protein kinase*), *SWITCH1*, and *JAZ5* (*jasmonate-zim-domain protein 5*) were not detected in our study. This probably because these genes’ regulatory interactions take place in stem cells, while our samples came from wood tissues. Moreover, we detected a group of target genes that associated with the cell cycle, such as Potom.002G05769, which encodes a cyclin family protein, and Potom.001G00363, which encodes mitogen-activated protein kinase kinase kinase 19. In addition, Potom.002G07206 (*ARF11*) was also identified as a target of Pto-Wuschela. These findings are consistent with the observation that WUS plays an essential role in stem cell maintenance, and participates in many processes including hormone signaling, metabolism, and development^13^. Taken together, these results demonstrate the power of our analysis strategy to detect interactions, prompting us to quantitatively investigate Pto-Wuschela-responsive genes associated with wood formation.

To fully understand the GRN centered on *Pto-Wuschela*, the functions of the genes in the network should be annotated in forest trees. However, few gene functional studies have been conducted in perennial plants because of their relatively large size, long generation times, and lack of mutants. To address this problem, population geneticists use genetic association to detect significant loci and/or functional genes for specific traits^21^. In our study, we identified 805 significant associations, including 501 SNPs from 53 genes, indicating that each of these genes may participate in at least one pathway involved in wood formation. More SNPs associated with DBH, V, and HolC under additive effects, and a few more SNPs associated with α-cellulose content, FL, and MFA under dominant effects^14^,^22^,^23^. In addition, SNPs with additive or dominant effects had different significances, indicating different contributions of the two effects to the overall significance (Figs. S3). These findings indicate that additive effects and dominant effects play more important roles in genetic variation associated with specific traits^15^. In total, 71% of the associations were under the additive model, while only 29% were under the dominance model, indicating that additive effects may play more important roles in controlling genetic variation of quantitative traits.

We analyzed *Pto-Wuschela* and 53 of its target genes under additive and dominant effects, and found that the 53 genes associated with more than one trait, indicating that these genes may be involved in same pathways^24^. Among these significant SNPs, *Pto-Wuschela*-SNP11 was associated with H and V, which is consistent with the result that *Pto-Wuschela* showed relatively high expression in mature and developing xylem tissues, and some expression in shoot apex and cambium (Fig. 1b), suggesting a role for Pto-Wuschela in shoot apical meristem maintenance and vascular development^11^,^25^. Nine SNPs from Potom.003G09473 (cytokinin response factor 2) also associated with V (Table S4), and the expression correlation between them was r = −0.91, revealing that *Pto-Wuschela* and Potom.003G09473 may be involved in the same process but play opposite roles. In addition, 11 SNPs from Potom.002G05769 (Cyclin family protein) and one SNP from *Pto-Wuschela* were associated with DBH, indicating that *Pto-Wuschela* and Potom.002G05769 may act in the same genetic pathways involved in peripheral growth. The expression correlation of the two genes was −0.82, consistent with the idea that Pto-Wuschela functions in stem cell maintenance, and may repress gene expression associated with the cell cycle. SNPs from *Pto-Wuschela* were associated with several tree growth and wood property traits, indicating that Pto-Wuschela is a crucial TF that has essential roles in perennial plant growth and development^11^,^25^,^26^. Moreover, we detected a target of Pto-Wuschela that responds to auxin, *ARF11*, which may regulate primary auxin response genes^27^. In addition, one gene involved in cell cycle or cell division, and two genes involved in hormone signaling, were also identified as targets of Pto-Wuschela. These results are consistent with a previous study showing that perennial plant growth is controlled by several processes, such as cell division and expansion in the shoot apex and cambium, development, and responses to hormones^28^. We detected two genes associated with cell wall biosynthesis (pectinacetylesterase family protein and carbohydrate-binding-like fold protein), which confirmed that *Pto-Wuschela* also affects wood properties. Furthermore, three transcription factors, Potom.001G03606 (zinc knuckle (CCHC-type) family protein), Potom.008G21326 (RING/FYVE/PHD zinc finger superfamily protein), and Potom.012G28409 (squamosa promoter-binding protein-like transcription factor family protein) were identified as the targets of Pto-Wuschela (Table S4), which may regulate more downstream genes. Also, Potom.002G07963 was associated with the growth trait DBH, and with the wood property traits MFA, HemC, HolC, and α-cellulose content. This gene has several functions, indicating that it may also participate in generating the building blocks of nucleic acids, and play essential roles in cell metabolism and regulation, such as providing energy and phosphate groups for phosphorylation, similar to the function of its homologous gene, AT4G13030.1. Potom.013G29538 encodes a homolog of an Arabidopsis Sec14p-like phosphatidylinositol transfer family protein, whose detailed function is unknown, but in our study, we found that Potom.013G29538 was associated with H and V, and wood property traits FW, FL, HemC, HolC, LC, and α-cellulose content, suggesting that it affects wood formation. These findings indicate that Pto-Wuschela functions in plant growth and development directly or indirectly through its target genes.

To further broaden our understanding of the genetic regulation of tree growth and wood formation, we developed a high-throughput method for GRN construction with *Pto-Wuschela* as the central hub, using epistasis analysis. Epistasis, also known as gene-gene interactions, is fundamentally important to understanding the functional elements of particular genetic pathways and essential mutants that drive adaptive evolution^29^,^30^. Epistasis analysis between polymorphic loci can be used to infer genetic networks affecting specific quantitative traits^16^. In our study, a three-layer hierarchical network was formed by epistatic interactions detected by epiSNP (Fig. 4). We found that the genes in the second layer interacted with more than two genes in the same layer, and interacted with more than one gene in the third layer, suggesting that these genes may act either within or between genetic pathways. In this respect, genetic interactions could help us understand gene function^31^. Moreover, analyzing the targets of TFs in the second and third layers will provide us more information to elucidate complex GRNs. We compared the networks constructed by genes associated with HolC and DBH and found many genes overlapped in both networks, indicating that these genes may participate in more than one pathway. Interestingly, genes in the second layer in the network associated with HolC (Fig. S4a) were in the third or fourth layer in the network associated with DBH (Fig. S4b), indicating that these genes have relatively diverse roles in different pathways.

Epistasis has been widely known as an essential factor of molecular evolution and was used to identify extra effects on phenotypic variation^32^,^33^. In this study, four types of SNP-SNP interactions were obtained: A × A (additive × additive), A × D (additive × dominant), D × A (dominant × additive, and D × D (dominant × dominant), representing the genetic interpretation of allele × allele, allele × genotype, genotype × allele, and genotype × genotype interactions, respectively^34^. For example, the interaction between *Pto-Wuschela*-SNP14 and Potom.014G31981-SNP25 showed an A × A effect with the A-T allele × allele having the highest α-cellulose content (Table 3), indicating that A-T is a desirable combination for selecting individuals with higher α-cellulose content. For the D × D effect, the genotype-genotype combination AA-TG for *Pto-Wuschela*-SNP14 × Potom.014G31981-SNP26 had the highest α-cellulose content (Table 3), indicating that the AA-TG genotype strongly affected α-cellulose content. Also, in trees with the AA genotype for *Pto-Wuschela*-SNP14, Potom.014G31981-SNP26 had significantly different genotypic effects, indicating the interaction of *Pto-Wuschela* and Potom.014G31981. The epistasis effects of *Pto-Wuschela*-SNP14 and Potom.014G31981-SNP26 contribute to α-cellulose content, suggesting that they may participate in the same pathways involved in wood formation.

The study of epistasis in forest trees remains in its infancy, but the use of epistasis to construct genetic architecture of quantitative traits has been implemented in several model animals^16^. However, work in animals indicates potential pitfalls of the approach. For example, a follow-on study failed to confirm the significant effects observed in a validation population in cattle; this may be caused by the dependence on LD, genetic differences between breeds, or even the specific animal population used. Here, 435 unrelated poplar individuals, whose LD is much lower than animals, were used for identifying epistasis effects, which may reduce the false positive rate in our study. However, further work should be performed to validate the significant epistasis effects^35^. Also, genome-wide deep sequencing and computer analysis software will make SNP-based association genetics analysis more powerful for construction of GRNs. Our study provides a powerful method to dissect genome-wide GRNs using epistasis analysis and expression data, which will be useful to decipher genetic interactions underlying quantitative traits of perennial plants.

## Methods

### Population and phenotype

A collection of 1,047 unrelated individuals of *P*. *tomentosa* was sampled in 1982 from an area of 1 million km^2^ along the Yellow River in northern China (30–40°N, 105–125°E), and were grown in Guan Xian County, Shandong Province, China (36°23′N, 115°47′E) using a randomized complete block design with three clonal replications^36^. We selected 435 unrelated individuals as an association population for SNP association studies.

Phenotypic data: Ten quantitative traits were measured from the 435 individuals, and the traits included: tree growth traits DBH, H, and V, and wood property traits HemC, HolC, α-cellulose content, LC, FL, FW, and MFA. The measurement of these phenotypic data was described in detail previously^31^,^37^.

### Identification and isolation of *Pto-Wuschela*

The cambium cDNA library was constructed using the Superscript k System (Life Technologies, Rockville, MD) from RNA isolated from cambium region tissue collected from one-year-old *P. tomentosa* clone “LM50” about 1.5 m above ground, as described previously. The cDNA library consisted of 5.0 × 10^6^ pfu and the insert size ranged from 1.0 to 4.0 kb. Random end-sequencing of 1000 cDNA clones and comparison with Arabidopsis sequences identified one full-length cDNA with high similarity of 86% to *AtWUS*. The gene was named *Pto-Wuschela*.

### Measurement of expression of *Pto-Wuschela* using RT-qPCR

RT-qPCR was performed on a 7500 Fast Real-Time PCR System using the SYBR Premix Ex Taq, as described in the manufacturer’s instructions. The cDNA template for the reactions was reverse-transcribed using total RNA extracted from root, shoot apex, cambium, developing xylem, mature xylem, phloem, young leaf, and old leaf. Primer Express 3.0 software (Applied Biosystems) was used to design primers for *Pto-Wuschela*. Poplar *Actin* (Accession number: EF145577) was used as the internal control. Triplicate technical and triplicate biological replicates were performed for all reactions. The results obtained from each tissue were standardized to *Actin*. Every reaction contained 2 μL of diluted cDNA (5 ng of total RNA), 10 μL of SYBR green PCR master mix (2×, Applied Biosystems) and 0.2 μmol each of the forward and the reverse primers, 0.4 μL ROX Reference Dye (50×) in a final volume of 20 μL. The conditions for PCR amplification were: 95 °C for 30 s, then 40 cycles at 95 °C for 3 s and 60 °C for 30 s for RT-qPCR amplification, and 1 cycle at 95 °C for 15 s, 60 °C for 1 min, and 95 °C for 15 s for dissociation.

### Identification of genes regulated by Pto-Wuschela

To identify genes that could be regulated by Pto-Wuschela, we used the conserved WUS-binding motifs “TCACGTGA” and “TAAT” to filter the promoter regions of total genes in *P. tomentosa*, because the DNA binding domain is highly conserved between AtWUS and Pto-Wuschela^10^. Then, transcriptome profiling datasets were used to find genes that showed expression levels with strong correlation to the expression of *Pto-Wuschela*. High-throughput transcriptome profiling of leaves, cambium, developing xylem, and mature xylem of *P. tomentosa* was performed by Shanghai Bio Institute (unpublished). Normalization and analysis of gene expression levels based on RNA-seq were conducted as previously described^38^. The significant differentially expressed genes were screened in the transcriptome data using fold-change (FC) ≥2 or ≤0.5, with a False Discovery Rate (FDR) q-value of 0.10 and p-value of 1.0E-03. Pearson product-moment correlation coefficient was calculated in R software, and r > 0.8 or r < −0.8, which indicate extremely strong correlation between expression of the TF and its targets, were used as thresholds for identification of candidate genes. Then we annotated these genes using the JGI gene models (http://popgenie.org/), and gene models of Arabidopsis (TAIR10) (http://www.arabidopsis.org/).

### Genome resequencing and genotyping

The 435 unrelated individuals were re-sequenced to >15× coverage (raw data) using the Illumina GA2 sequencing platform. The sequencing quality of these raw reads was generally high (90% with Phred quality score > 27). We first mapped the paired-end short reads of 100 bp back to the *Populus* reference genome sequence using the SOAPaligner (SOAP2) (version 2.20) with default options. The mapping rate in different accessions varied from 81% to 92%, and the effective mapping depth was ~11× for most individuals.

To get high-quality SNPs, we selected uniquely mapped single-end and paired-end reads to perform SNP calling. The genotype likelihood of the genomic site for each tree was calculated using SOAPsnp with default parameters^39^. To validate our SNP calling results, we randomly compared them with our previous SNP data from ten candidate genes in 120 trees, using data from genome resequencing with PCR-Sanger sequencing^28^. The accuracy of SNP calling was 99.7%, indicating the high quality of the SNP-calling platform. To identify homologs, we used BLASTX with a cutoff E-value < 1e-10, and identified gene-derived bi-allelic SNPs using UltraEdit 3.2 (http://www.ultraedit.com/).

### Statistical analysis

The mixed linear model (MLM) in the package TASSEL 2.1 (http://www.maizegenetics.net/) was used to identify single-marker associations in the association population with the consideration of population structure. epiSNP is a computer package designed for detecting single-locus associations under additive and dominant effect models, and pairwise epistasis effects using SNP markers in genome-wide association studies. One of the packages, EPISNP1, which uses a least-squares test for unrelated individuals, is suitable for identification of significant SNP markers using natural distributed forest populations^40^. The EPISNP1 program generates two results files: one for three single-locus effects of each SNP including SNP genotypic effect (m), additive (a), and dominance effects (d), and the other for four epistatic effects of each pair of SNPs, including additive × additive (A×A), additive × dominance (A×D), dominance × additive (D×A), and dominance × dominance (D×D). The additive and dominant effects were based on the MLM method and the epistasis effects were based on the extended Kempthorne model^41^. We used the “BH” method^42^ in p.adjust.methods to control the false discovery rate using R software. A cutoff of p ≤ 0.01 and FDR ≤ 0.01 were used for selecting significant associations.

## Additional Information

**How to cite this article**: Yang, X. *et al.* The genetic regulatory network centered on *Pto-Wuschela* and its targets involved in wood formation revealed by association studies. *Sci. Rep.* **5**, 16507; doi: 10.1038/srep16507 (2015).

## Supplementary Material

Supplementary Information

## Figures and Tables

**Table 1:** Significant SNPs associated with wood properties and tree growth traits under additive and dominant effect models in a *Populus tomentosa* association population (significance ≥ 2).

Traits	Additive effects			Dominant effects		
	SNPs	Genes	Significance	SNPs	Genes	Significance
LC (%)	14	6	2.01–3.05	10	8	2.03–3.07
HolC (%)	43	15	2.00–4.23	22	14	2.02–3.59
HemC (%)	29	19	2.00–3.42	28	18	2.03–3.70
CC (%)	14	8	2.14–3.27	16	10	2.00–3.73
FL (mm)	5	5	2.00–3.24	10	5	2.04–4.23
FW (μm)	34	18	2.00–4.19	29	17	2.00–6.86
MFA (°)	13	10	2.00–3.10	16	10	2.10–3.73
DBH (cm)	223	38	2.00–4.89	52	16	2.02–3.75
H (m)	6	4	2.01–2.74	7	6	2.01–2.81
V (m^3^)	193	30	2.00–4.558	42	18	2.00–5.99

LC: Lignin content; HolC: Holocellulose content; HemC: hemicellulose content; CC: α-cellulose content; FL: Fiber length; FW: Fiber width; MFA: Microfiber angle; DBH: Diameter at breast height; H: Tree height; V: Stem volume.

*Significance = log_10_ (1/p).

**Table 2:** Significant SNP-SNP interactions associated with wood properties and tree growth in a *Populus tomentosa* association population (*P* ≤ 0.01).

Traits	SNP-SNP	Genes	Test type	Test number	Significance*
MFA (°)	96	33	AA	31	2.01–3.43
			AD	25	2.09–3.48
			DA	21	2.03–3.37
			DD	19	2.01–2.65
DBH (cm)	351	38	AA	134	2.00–4.69
			AD	94	2.00–3.93
			DA	70	2.01–3.79
			DD	53	2.00–4.03
FL (mm)	290	34	AA	75	2.00–3.34
			AD	92	2.01–4.09
			DA	67	2.00–3.61
			DD	56	2.01–4.31
H (m)	99	33	AA	18	2.01–3.57
			AD	29	2.00–2.68
			DA	30	2.02–3.05
			DD	22	2.01–3.06
HemC (%)	248	39	AA	58	2.00–3.68
			AD	60	2.00–3.56
			DA	68	2.02–4.88
			DD	62	2.02–3.37
HolC (%)	375	35	AA	127	2.00–4.92
			AD	104	2.00–4.41
			DA	96	2.00–3.74
			DD	48	2.02–5.91
V (m3)	587	39	AA	170	2.00–6.37
			AD	156	2.00–5.49
			DA	142	2.00–4.53
			DD	119	2.00–5.70
FW (μm)	138	36	AA	21	2.01–2.60
			AD	43	2.01–5.13
			DA	42	2.00–3.57
			DD	32	2.00–4.61
CC (%)	209	35	AA	47	2.01–3.94
			AD	61	2.00–4.64
			DA	43	2.05–3.96
			DD	58	2.01–3.52
LC (%)	111	28	AA	36	2.02–2.94
			AD	13	2.01–3.26
			DA	32	2.01–3.16
			DD	30	2.00–3.69

LC: Lignin content; HolC: Holocellulose content; HemC: hemicellulose content; CC: α-cellulose content; FL: Fiber length; FW: Fiber width; MFA: Microfiber angle; DBH: Diameter at breast height; V: Stem volume.

^*^Significance = log_10_ (1/p).

**Table 3:** Significant SNPs interacting with *Pto-Wuschela*-SNP14 associated with α-cellulose content.

SNP1	SNP2	Test	Effect	*P_value*	Allele/Genotype		
					Type	Effect	Frequency
*Pto-Wuschela–SNP14*	Potom.014G31981–SNP25	AA	−5.02	0.0051	A-T	2.07	0.107
					T-C	0.514	0.426
					A-C	−0.637	0.343
					T-T	−1.8	0.124
*Pto-Wuschela–SNP14*	Potom.014G31981–SNP26	AA	−4.48	0.00466	A-G	1.72	0.13
					T-T	0.542	0.405
					A-T	−0.683	0.32
					T-G	−1.54	0.145
*Pto-Wuschela–SNP14*	Potom.001G00363–SNP19	DA	−2.7	0.00628	AA-A	8.86	0.0185
					TT-A	2.24	0.117
					AT-T	1.95	0.222
					AT-A	−0.872	0.556
					TT-T	−4.1	0.0556
					AA-T	−4.48	0.0309
*Pto-Wuschela–SNP14*	Potom.009G23029–SNP3	DD	−18.2	0.00813	AT-CC	11.4	0.0119
					AA-TC	10.6	0.0238
					TT-TC	2.58	0.131
					AT-TT	1.21	0.25
					TT-CC	0.0304	0.0119
					AT-TC	−0.282	0.524
					TT-TT	−0.87	0.0238
					AA-CC	−8.77	0.0119
					AA-TT	−10.7	0.0119
*Pto-Wuschela–SNP14*	Potom.014G31981–SNP25	DD	−19.1	0.00328	AA-CT	16.2	0.0125
					TT-CT	8.47	0.025
					TT-TT	2.18	0.025
					AT-CC	0.736	0.562
					AT-TT	0.0939	0.113
					TT-CC	−2.86	0.1
					AT-CT	−3.57	0.125
					AA-CC	−4.31	0.025
					AA-TT	−6.23	0.0125
*Pto-Wuschela–SNP14*	Potom.014G31981–SNP26	DD	−19.3	0.00311	AA-TG	15.8	0.0125
					TT-TG	9.71	0.025
					TT-GG	2.2	0.025
					AT-TT	0.778	0.525
					AT-GG	−0.399	0.163
					TT-TT	−2.93	0.1
					AT-TG	−3.44	0.113
					AA-TT	−4.47	0.025
					AA-GG	−6.28	0.0125
