# Supplementary material for: The genetic regulatory network centered on Pto-Wuschela and its targets involved in wood formation revealed by association studies
Source: Sci Rep. 2015 Nov 9;5:16507. doi: 10.1038/srep16507 (PMC4637887; doi:10.1038/srep16507)
Supplement: Supplementary Information [file srep16507-s1.pdf]

The genetic regulatory network centered on *Pto-Wuschela* and its targets involved in wood formation revealed by association studies

Xiaohui Yang[1,2*], Zunzheng Wei[3*], Qingzhang Du[1,2], Jinhui Chen[1,2], Qingshi Wang[1,2], Mingyang Quan[1,2], Yuepeng Song[1,2], Jianbo Xie[1,2] and Deqiang Zhang[1,2,*]

[1]National Engineering Laboratory for Tree Breeding, College of Biological Sciences and Technology, Beijing Forestry University, No. 35, Qinghua East Road, Beijing 100083, P. R. China;

[2]Key Laboratory of Genetics and Breeding in Forest Trees and Ornamental Plants, Ministry of Education, College of Biological Sciences and Technology, Beijing Forestry University, No. 35, Qinghua East Road, Beijing 100083, P. R. China;

[3]Beijing Vegetable Research Center, Beijing Academy of Agriculture and Forestry Sciences, Key Laboratory of Biology and Genetic Improvement of Horticultural Crops (North China), Key Laboratory of Urban Agriculture (North), Ministry of Agriculture, No. 50, Zhanghua Road, Beijing 10097, China.

[*]These authors contributed equally to this work.

To whom correspondence should be addressed. Tel: +86-10-62336007; Fax: +86-10-62336164. E-mail: DeqiangZhang@bjfu.edu.cn.

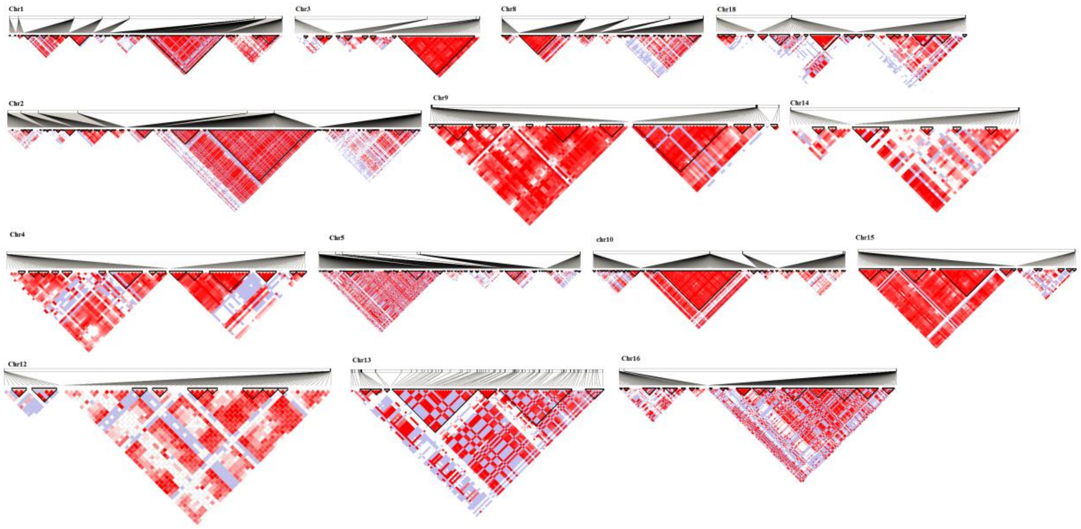

Figure S1 Linkage disequilibrium of the 53 candidate genes located in each chromosome.

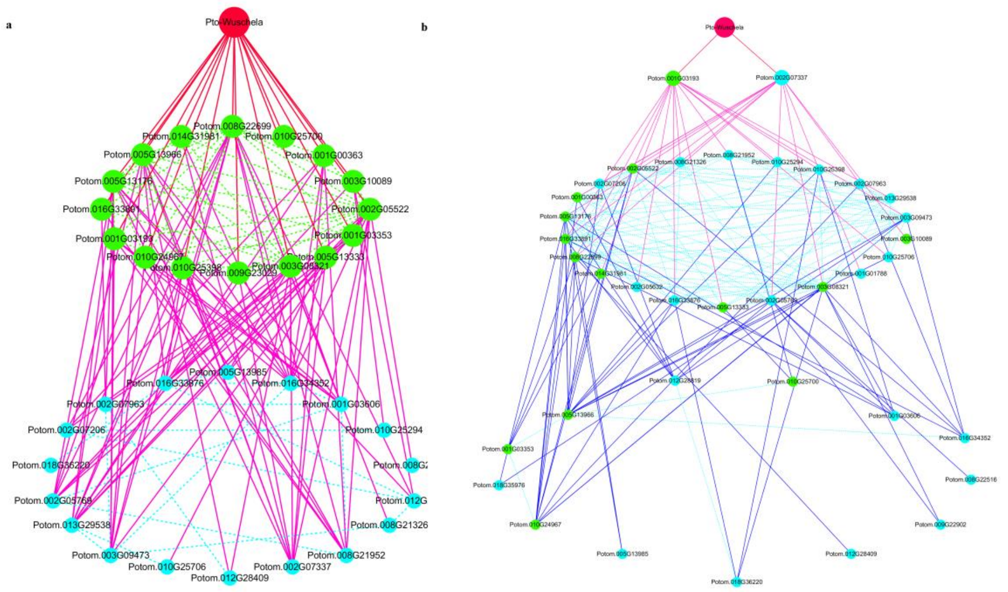

Figure S2 Gene-gene interaction network formed by the epistatic interactions of genes associated with holocellulose content and diameter at breast height. (a): epistatic interactions formed by genes associated with HolC, including 375 epistasis interactions among 35 genes. (b): epistatic interactions formed by genes associated with DBH, including 351 epistasis interactions among 38 genes.

Table S1 53 potential Pto-Wuschela target genes identified by bioinformatics and transcriptional profiling.

Populus tomentosa	Populus trichocarpa	TAIR	Gene names	Correlation
Potom.001G00161	Potri.001G018100.1	AT2G39725.1	LYR family of Fe/S cluster biogenesis protein	-0.92701
Potom.001G00363	Potri.001G042400.1	AT5G67080.1	mitogen-activated protein kinase kinase kinase 19	-0.86141
Potom.001G01331	Potri.001G119000.1	AT1G62790.2	Bifunctional inhibitor/lipid-transfer protein/seed storage 2S albumin superfamily protein	-0.80236
Potom.001G01788	Potri.001G161900.1	AT1G53800.1	Unknown protein	-0.96697
Potom.001G02062	Potri.014G181600.1	AT4G36660.1	Protein of unknown function (DUF1195)	0.904465
Potom.001G03193	Potri.001G280400.1	AT5G58530.1	Glutaredoxin family protein	0.990279
Potom.001G03353	Potri.001G295800.1	AT1G49890.1	Family of unknown function (DUF566)	0.946536
Potom.001G03606	Potri.001G318100.1	AT3G42860.1	zinc knuckle (CCHC-type) family protein	-0.8202
Potom.002G05522	Potri.002G010400.1	AT5G42330.1	Unknown protein	0.910642
Potom.002G05632	Potri.005G241100.1	AT4G24190.2	Chaperone protein htpG family protein	0.834866
Potom.002G05769	Potri.002G032300.1	AT5G45190.1	Cyclin family protein	-0.8221
Potom.002G06086	Potri.002G063100.1	AT1G71730.1	Unknown protein	0.804983
Potom.002G07206	Potri.002G172800.1	AT2G46530.1	auxin response factor 11	-0.81762
Potom.002G07337	Potri.002G194000.1	AT3G62360.1	Carbohydrate-binding-like fold	0.879314
Potom.002G07963	Potri.002G247400.1	AT4G13030.1	P-loop containing nucleoside triphosphate hydrolases superfamily protein	-0.94421
Potom.003G08321	Potri.003G018700.1	AT1G22030.1	Unknown protein	0.976655
Potom.003G09473	Potri.003G136300.1	AT4G23750.1	cytokinin response factor 2	-0.90571
Potom.003G10089	Potri.003G197700.1	AT3G54460.1	SNF2 domain-containing protein / helicase domain-containing protein / F-box family protein	-0.89507
Potom.003G10130	Potri.003G202900.1	AT3G08550.1	elongation defective 1 protein / ELD1 protein	0.965693
Potom.004G11193	Potri.004G087500.1	AT1G14900.1	high mobility group A	-0.90683
Potom.004G12312	Potri.004G206800.1	AT2G42005.1	Transmembrane amino acid transporter family protein	0.954128
Potom.005G13176	Potri.005G070300.1	AT5G64440.1	fatty acid amide hydrolase	0.874478
Potom.005G13333	Potri.005G085700.1	AT4G39470.1	Tetratricopeptide repeat (TPR)-like superfamily protein	-0.99005

Potom.005G13398	Potri.001G168200.1	AT1G54130.1	RELA/SPOT homolog 3	-0.84529
Potom.005G13966	Potri.005G146700.1	AT2G22830.1	squalene epoxidase 2	0.934927
Potom.005G13985	Potri.005G148700.1	AT4G37860.1	SPT2 chromatin protein	0.855555
Potom.005G14995	Potri.001G384100.1	AT1G78610.1	mechanosensitive channel of small conductance-like 6	0.999217
Potom.008G19885	Potri.008G042000.1	AT3G52260.2	Pseudouridine synthase family protein	0.816577
Potom.008G21326	Potri.008G081200.1	AT1G79350.1	RING/FYVE/PHD zinc finger superfamily protein	-0.94068
Potom.008G21952	Potri.008G144900.1	AT1G10850.1	Leucine-rich repeat protein kinase family protein	0.992949
Potom.008G22516	Potri.008G219000.1	AT3G06810.1	acyl-CoA dehydrogenase-related	-0.97227
Potom.008G22699	Potri.008G220400.1	AT3G06860.1	multifunctional protein 2	0.856291
Potom.009G22902	Potri.009G021200.1	AT5G53530.1	vacuolar protein sorting 26A	-0.86311
Potom.009G23017	Potri.009G031900.1	AT2G29050.1	RHOMBOID-like 1	0.9355
Potom.009G23029	Potri.009G032700.1	AT1G07530.1	SCARECROW-like 14	0.825607
Potom.010G24967	Potri.010G035300.1	AT1G69910.1	Protein kinase superfamily protein	0.871246
Potom.010G25294	Potri.010G105900.1	AT1G14830.1	DYNAMIN-like 1C	0.964059
Potom.010G25398	Potri.010G117100.1	AT1G71150.1	Unknown protein	-0.94331
Potom.010G25700	Potri.010G223700.1	AT4G14145.1	Unknown protein	-0.87606
Potom.010G25706	Potri.008G038700.1	AT2G01720.1	Ribophorin I	0.913224
Potom.012G28409	Potri.012G100700.1	AT5G50670.1	Squamosa promoter-binding protein-like (SBP domain) transcription factor family protein	0.984338
Potom.012G28819	Potri.012G142300.1	AT5G23870.3	Pectinacetylesterase family protein	0.925199
Potom.013G29538	Potri.013G068700.1	AT1G22180.2	Sec14p-like phosphatidylinositol transfer family protein	-0.80597
Potom.014G31876	Potri.014G162400.1	AT5G35732.1	Unknown protein	-0.90963
Potom.014G31981	Potri.014G174300.1	AT5G48340.1	Unknown protein	-0.85159
Potom.015G32682	Potri.015G036000.1	AT3G17880.1	tetraticopeptide domain-containing thioredoxin	-0.91467
Potom.015G33266	Potri.015G095000.1	AT5G41020.1	myb family transcription factor	0.809642
Potom.016G33876	Potri.016G015200.1	AT3G27200.1	Cupredoxin superfamily protein	-0.92597
Potom.016G33891	Potri.016G017800.1	AT5G46680.1	Pentatricopeptide repeat (PPR-like) superfamily protein	-0.85016

Potom.016G34352	Potri.016G121100.1	AT1G80930.1	MIF4G domain-containing protein / MA3 domain-containing protein	0.984077
Potom.018G35976	Potri.018G001700.1	-	-	-0.92987
Potom.018G36220	Potri.018G028900.1	AT4G32390.1	Nucleotide-sugar transporter family protein	0.890323
Potom.018G36550	Potri.018G065000.1	AT3G13980.1	Unknown protein	0.807841

Table S2 Gene information and single nucleotide polymorphisms of *Pto-Wuschela* and its putative targets.

**Gene name**	**Gene length**	**Number of Polymorphic Sites**	**Frequency (bp-1)**	**Common SNP in Promoter region**	**Common SNP in Gene region**	**Total common SNP**	**Gene models**
Pto-Wuschela	1685	63	27	0	18	18	Potri.005G114700
Potom.001G00161	2880	101	29	15	5	20	Potri.001G018100
Potom.001G00363	3525	153	23	51	4	55	Potri.001G042400
Potom.001G01331	3815	189	20	44	54	98	Potri.001G119000
Potom.001G01788	6569	191	34	5	66	71	Potri.001G161900
Potom.001G02062	3792	126	30	28	7	35	Potri.014G181600
Potom.001G03193	3309	192	17	71	13	84	Potri.001G280400
Potom.001G03353	9562	321	30	39	139	178	Potri.001G295800
Potom.001G03606	5057	282	18	39	51	90	Potri.001G318100
Potom.002G05522	7098	243	29	29	52	81	Potri.002G010400
Potom.002G05632	3613	214	16	66	22	88	Potri.005G241100
Potom.002G05769	5168	219	24	48	24	72	Potri.002G032300
Potom.002G06086	3771	217	17	18	10	28	Potri.002G063100
Potom.002G07206	6161	182	34	33	44	77	Potri.002G172800
Potom.002G07337	15130	628	24	65	263	328	Potri.002G194000
Potom.002G07963	12055	662	18	9	261	270	Potri.002G247400
Potom.003G08321	2948	92	32	22	16	38	Potri.003G018700
Potom.003G09473	3768	144	26	21	9	30	Potri.003G136300
Potom.003G10089	3005	114	26	24	8	32	Potri.003G197700
Potom.003G10130	7474	184	41	16	60	76	Potri.003G202900
Potom.004G11193	4296	195	22	24	54	78	Potri.004G087500
Potom.004G12312	3620	174	21	27	22	49	Potri.004G206800

Potom.005G13176	9533	485	20	72	240	312	Potri.005G070300
Potom.005G13333	5009	238	21	4	37	41	Potri.005G085700
Potom.005G13398	2315	183	13	62	4	66	Potri.001G168200
Potom.005G13966	6305	260	24	48	54	102	Potri.005G146700
Potom.005G13985	4930	168	29	6	25	31	Potri.005G148700
Potom.005G14995	3273	200	16	79	23	102	Potri.001G384100
Potom.008G19885	4408	79	56	1	25	26	Potri.008G042000
Potom.008G21326	5952	394	15	26	76	102	Potri.008G081200
Potom.008G21952	6200	207	30	15	29	44	Potri.008G144900
Potom.008G22516	12259	345	36	4	50	54	Potri.008G219000
Potom.008G22699	13553	576	24	54	139	193	Potri.008G220400
Potom.009G22902	8544	422	20	2	63	65	Potri.009G021200
Potom.009G23017	5726	218	26	15	47	62	Potri.009G031900
Potom.009G23029	4006	43	93	0	4	4	Potri.009G032700
Potom.010G24967	3914	206	19	81	24	105	Potri.010G035300
Potom.010G25294	13234	531	25	34	200	234	Potri.010G105900
Potom.010G25398	7158	417	17	55	16	71	Potri.010G117100
Potom.010G25700	5145	136	38	12	35	47	Potri.010G223700
Potom.010G25706	5477	287	19	58	40	98	Potri.008G038700
Potom.012G28409	3375	81	42	20	5	25	Potri.012G100700
Potom.012G28819	7390	219	34	6	53	59	Potri.012G142300
Potom.013G29538	6775	306	22	27	118	145	Potri.013G068700
Potom.014G31876	3080	96	32	16	5	21	Potri.014G162400
Potom.014G31981	5201	250	21	48	36	84	Potri.014G174300
Potom.015G32682	7704	292	26	39	95	134	Potri.015G036000
Potom.015G33266	4961	180	28	10	34	44	Potri.015G095000

Potom.016G33876	3557	261	14	54	58	112	Potri.016G015200
Potom.016G33891	2750	104	26	21	8	29	Potri.016G017800
Potom.016G34352	7689	455	17	78	179	257	Potri.016G121100
Potom.018G35976	2765	112	25	32	0	32	Potri.018G001700
Potom.018G36220	3791	160	24	40	54	94	Potri.018G028900
Potom.018G36550	3228	159	20	61	9	70	Potri.018G065000

Table S3 Significant SNPs identified by association analysis using TASSEL2.1.

Trait	Locus	p_value	R^2
Diameter at breast height	Potom.002G05522-SNP76	6.71E-05	0.00118
Diameter at breast height	Potom.002G07337-SNP118	1.21E-17	0.001284
Diameter at breast height	Potom.005G13985-SNP21	5.95E-09	0.001328
Diameter at breast height	Potom.018G36220-SNP10	1.67E-73	0.001424
Tree height	Potom.015G32682-SNP29	8.45E-09	0.001431
Diameter at breast height	Potom.010G24967-SNP2	5.51E-32	0.001575
Diameter at breast height	Potom.008G22516-SNP31	1.52E-33	0.001585
Diameter at breast height	Potom.015G32682-SNP84	4.28E-23	0.00166
Diameter at breast height	Potom.015G32682-SNP83	4.28E-23	0.00166
Diameter at breast height	Potom.008G19885-SNP19	6.14E-04	0.001664
Diameter at breast height	Potom.018G36550-SNP48	2.72E-14	0.00183
Tree height	Potom.008G22699-SNP148	1.79E-06	0.00194
Diameter at breast height	Potom.012G28819-SNP59	8.27E-17	0.002009
Tree height	Potom.001G03353-SNP150	1.93E-09	0.002023
Diameter at breast height	Potom.002G07963-SNP149	3.38E-24	0.002065
Diameter at breast height	Potom.015G32682-SNP58	2.78E-31	0.002143
Diameter at breast height	Potom.008G21326-SNP44	2.78E-31	0.002143
Diameter at breast height	Potom.002G07337-SNP91	2.78E-31	0.002143
Diameter at breast height	Potom.002G07337-SNP90	2.78E-31	0.002143
Diameter at breast height	Potom.002G07206-SNP61	2.78E-31	0.002143
Diameter at breast height	Potom.002G05632-SNP88	2.78E-31	0.002143
Diameter at breast height	Potom.001G01788-SNP44	2.78E-31	0.002143
Tree height	Potom.018G35976-SNP7	6.91E-18	0.002353
Tree height	Potom.009G22902-SNP47	6.91E-18	0.002353
Tree height	Potom.009G22902-SNP46	6.91E-18	0.002353
Tree height	Potom.008G21326-SNP9	6.91E-18	0.002353
Tree height	Potom.004G12312-SNP6	6.91E-18	0.002353
Tree height	Potom.005G13176-SNP88	6.88E-18	0.002366
Diameter at breast height	Potom.001G03353-SNP31	4.94E-52	0.002864
Diameter at breast height	Potom.001G03353-SNP30	4.94E-52	0.002864
Tree height	Potom.002G07337-SNP112	3.86E-08	0.003485
Diameter at breast height	Potom.003G10130-SNP67	4.52E-21	0.003705
Diameter at breast height	Potom.001G03353-SNP125	8.68E-67	0.003851
Diameter at breast height	Potom.001G03193-SNP2	8.68E-67	0.003851
Diameter at breast height	Potom.001G01331-SNP75	8.68E-67	0.003851
Tree height	Potom.018G36220-SNP84	5.35E-13	0.003908
Tree height	Potom.001G02062-SNP7	5.12E-12	0.003987
Tree height	Potom.002G07337-SNP202	5.11E-16	0.004535
Tree height	Potom.002G07337-SNP201	5.11E-16	0.004535
Diameter at breast height	Potom.015G32682-SNP26	5.19E-33	0.005254
Diameter at breast height	Potom.016G33876-SNP101	9.53E-83	0.005338
Tree height	Potom.005G13176-SNP275	8.97E-07	0.005521

Diameter at breast height	Potom.001G00363-SNP31	1.09E-09	0.005531
Tree height	Potom.002G07963-SNP251	1.25E-13	0.005541
Tree height	Potom.005G14995-SNP53	1.59E-16	0.005622
Tree height	Potom.001G03353-SNP99	4.44E-06	0.005645
Tree height	Potom.001G03353-SNP94	4.44E-06	0.005645
Tree height	Potom.001G03353-SNP9	4.44E-06	0.005645
Tree height	Potom.001G03353-SNP79	4.44E-06	0.005645
Tree height	Potom.001G03353-SNP48	4.44E-06	0.005645
Tree height	Potom.001G03353-SNP153	4.44E-06	0.005645
Tree height	Potom.001G03353-SNP145	4.44E-06	0.005645
Tree height	Potom.001G03353-SNP143	4.44E-06	0.005645
Tree height	Potom.001G03353-SNP130	4.44E-06	0.005645
Tree height	Potom.001G03353-SNP10	4.44E-06	0.005645
Diameter at breast height	Potom.005G13176-SNP29	3.06E-30	0.005818
Tree height	Potom.009G23029-SNP4	1.25E-12	0.006091
Tree height	Potom.002G07963-SNP140	6.73E-12	0.006381
Diameter at breast height	Potom.001G03193-SNP15	8.96E-24	0.006931
Tree height	Potom.002G07337-SNP220	2.65E-13	0.006971
Tree height	Potom.002G07337-SNP219	2.65E-13	0.006971
Tree height	Potom.001G03353-SNP136	2.91E-43	0.007552
Diameter at breast height	Potom.004G12312-SNP17	1.17E-22	0.007957
Diameter at breast height	Potom.002G07337-SNP169	1.17E-22	0.007957
Diameter at breast height	Potom.002G07206-SNP59	1.17E-22	0.007957
Diameter at breast height	Potom.002G05632-SNP82	1.17E-22	0.007957
Diameter at breast height	Potom.001G03353-SNP135	2.38E-08	0.00875
Diameter at breast height	Potom.001G01331-SNP82	4.41E-27	0.009171
Diameter at breast height	Potom.001G01331-SNP81	4.41E-27	0.009171
Diameter at breast height	Potom.002G07963-SNP67	1.25E-06	0.009446
Diameter at breast height	Potom.002G07963-SNP199	2.00E-16	0.009695
Diameter at breast height	Potom.016G34352-SNP12	2.37E-19	0.010034
Diameter at breast height	Potom.002G07206-SNP52	1.31E-39	0.010255
Diameter at breast height	Potom.001G03353-SNP66	1.76E-38	0.010749
Diameter at breast height	Potom.018G36220-SNP29	1.41E-11	0.011
Diameter at breast height	Potom.009G22902-SNP51	8.99E-25	0.0115
Tree height	Potom.018G36220-SNP10	2.42E-17	0.011875
Tree height	Potom.015G32682-SNP64	7.27E-12	0.011875
Tree height	Potom.001G03353-SNP43	3.56E-10	0.011914
Diameter at breast height	Potom.016G34352-SNP119	5.89E-14	0.012
Tree height	Potom.003G10130-SNP75	8.34E-11	0.013
Diameter at breast height	Potom.018G36220-SNP83	7.72E-14	0.0135
Diameter at breast height	Potom.008G22699-SNP102	2.51E-21	0.014
Diameter at breast height	Potom.005G13176-SNP58	1.36E-13	0.0185
Diameter at breast height	Potom.002G07963-SNP100	7.46E-22	0.0205
Tree height	Potom.002G07963-SNP101	1.44E-21	0.021

Diameter at breast height	Potom.010G25294-SNP184	9.85E-21	0.022
Diameter at breast height	Potom.010G25294-SNP24	7.38E-26	0.0225
Diameter at breast height	Potom.010G25294-SNP23	7.38E-26	0.0225
Diameter at breast height	Potom.002G07963-SNP139	9.63E-17	0.0235
Diameter at breast height	Potom.002G07963-SNP101	2.27E-130	0.026
Diameter at breast height	Potom.002G07206-SNP21	7.25E-20	0.0275
Diameter at breast height	Potom.002G07337-SNP85	4.33E-25	0.034
Diameter at breast height	Potom.002G07206-SNP67	3.81E-15	0.034
Stem volume	Potom.008G19885-SNP21	9.35E-04	0.0609
Stem volume	Potom.008G22699-SNP58	9.29E-04	0.06095
Stem volume	Potom.008G22699-SNP162	9.29E-04	0.06095
Stem volume	Potom.005G14995-SNP11	9.29E-04	0.06095
Holocellulose content	Potom.004G11193-SNP69	9.31E-04	0.06145
Diameter at breast height	Potom.005G13176-SNP272	9.69E-04	0.0617
Diameter at breast height	Potom.005G13176-SNP264	9.26E-04	0.06215
Diameter at breast height	Potom.005G13176-SNP262	9.26E-04	0.06215
Diameter at breast height	Potom.005G13176-SNP245	9.26E-04	0.06215
Diameter at breast height	Potom.005G13176-SNP242	9.26E-04	0.06215
Stem volume	Potom.001G03353-SNP135	8.21E-04	0.06215
α-cellulose content	Potom.008G19885-SNP26	9.32E-04	0.0627
Fiber width	Potom.010G25294-SNP179	1.00E-03	0.06285
Fiber width	Potom.002G07963-SNP188	8.91E-04	0.0629
Fiber width	Potom.008G22699-SNP78	8.59E-04	0.063
Fiber width	Potom.010G25294-SNP193	8.91E-04	0.0634
Fiber width	Potom.005G13176-SNP59	9.60E-04	0.0634
α-cellulose content	Potom.003G10089-SNP31	9.32E-04	0.06355
Fiber width	Potom.015G33266-SNP25	9.71E-04	0.06365
Stem volume	Potom.008G22699-SNP67	7.61E-04	0.06365
Diameter at breast height	Potom.002G07337-SNP184	7.94E-04	0.06365
Fiber width	Potom.003G10130-SNP26	8.65E-04	0.06385
Holocellulose content	Potom.002G05522-SNP40	5.59E-04	0.06395
Diameter at breast height	Potom.005G13176-SNP305	7.68E-04	0.064
Fiber width	Potom.015G32682-SNP62	8.15E-04	0.0642
Fiber width	Potom.010G25294-SNP28	8.15E-04	0.0642
Diameter at breast height	Potom.008G22699-SNP174	8.20E-04	0.0642
Stem volume	Potom.005G13966-SNP91	7.86E-04	0.0642
Diameter at breast height	Potom.015G33266-SNP35	9.50E-04	0.06435
Diameter at breast height	Potom.016G34352-SNP241	7.27E-04	0.0645
Stem volume	Potom.001G03353-SNP5	6.35E-04	0.0646
Fiber width	Potom.001G03353-SNP126	8.31E-04	0.0646
Diameter at breast height	Potom.005G13176-SNP101	7.14E-04	0.0647
Diameter at breast height	Potom.001G02062-SNP9	7.65E-04	0.0652
Fiber width	Potom.015G32682-SNP64	7.43E-04	0.06525
Fiber width	Potom.016G33891-SNP9	7.00E-04	0.0653

Fiber width	Potom.008G21326-SNP89	7.40E-04	0.0653
Diameter at breast height	Potom.010G25294-SNP74	6.57E-04	0.0655
Diameter at breast height	Potom.010G25398-SNP2	6.46E-04	0.06565
Diameter at breast height	Potom.005G13966-SNP23	6.46E-04	0.06565
α-cellulose content	Potom.002G07963-SNP109	6.24E-04	0.0659
Diameter at breast height	Potom.010G25706-SNP31	6.18E-04	0.0661
Diameter at breast height	Potom.002G07963-SNP68	7.75E-04	0.0662
Fiber width	Potom.002G05769-SNP51	7.21E-04	0.0664
Fiber width	Potom.002G05522-SNP20	6.55E-04	0.0665
Holocellulose content	Potom.004G11193-SNP68	6.88E-04	0.0668
Fiber width	Potom.005G13176-SNP286	6.35E-04	0.06695
Microfiber angle	Potom.018G36220-SNP4	8.92E-04	0.067
Stem volume	Potom.016G34352-SNP86	7.59E-04	0.0672
Diameter at breast height	Potom.016G34352-SNP211	5.43E-04	0.06735
Diameter at breast height	Potom.008G22699-SNP160	5.43E-04	0.06735
Holocellulose content	Potom.004G11193-SNP23	3.84E-04	0.0674
Holocellulose content	Potom.004G11193-SNP22	3.84E-04	0.0674
Fiber width	Potom.009G22902-SNP42	6.26E-04	0.06745
Fiber width	Potom.015G32682-SNP65	6.11E-04	0.0675
Fiber width	Potom.001G01788-SNP33	5.72E-04	0.0675
Diameter at breast height	Potom.016G34352-SNP122	5.31E-04	0.06755
Stem volume	Potom.016G33891-SNP22	4.73E-04	0.06755
Fiber width	Potom.008G21326-SNP99	6.09E-04	0.06765
Fiber width	Potom.003G10130-SNP23	6.41E-04	0.06765
Stem volume	Potom.004G11193-SNP46	4.79E-04	0.06805
Stem volume	Potom.001G03193-SNP37	6.38E-04	0.06835
Fiber width	Potom.002G05632-SNP70	5.42E-04	0.0685
Stem volume	Potom.018G36550-SNP68	4.09E-04	0.0688
Stem volume	Potom.016G33891-SNP18	8.22E-04	0.0688
Fiber width	Potom.008G21326-SNP91	5.02E-04	0.06935
Stem volume	Potom.001G03353-SNP51	3.85E-04	0.0694
Stem volume	Potom.001G03353-SNP131	3.85E-04	0.0694
Stem volume	Potom.001G03353-SNP129	3.85E-04	0.0694
Stem volume	Potom.001G03353-SNP127	3.85E-04	0.0694
Stem volume	Potom.001G03353-SNP121	3.85E-04	0.0694
Microfiber angle	Potom.018G36220-SNP3	7.48E-04	0.06975
Diameter at breast height	Potom.012G28819-SNP16	4.05E-04	0.0702
Fiber width	Potom.001G03606-SNP52	7.79E-04	0.07025
Fiber width	Potom.002G06086-SNP14	4.53E-04	0.0705
Fiber width	Potom.001G03606-SNP56	6.80E-04	0.0708
Fiber width	Potom.001G03606-SNP55	6.80E-04	0.0708
Fiber width	Potom.001G03606-SNP54	7.19E-04	0.071
Microfiber angle	Potom.018G36220-SNP6	5.54E-04	0.07175
Microfiber angle	Potom.018G36220-SNP5	5.54E-04	0.07175

Diameter at breast height	Potom.005G13966-SNP68	6.93E-04	0.0718
Fiber width	Potom.002G07963-SNP192	3.75E-04	0.07235
Stem volume	Potom.001G03353-SNP95	2.73E-04	0.07265
Fiber width	Potom.016G34352-SNP118	3.73E-04	0.0727
Holocellulose content	Potom.008G19885-SNP26	2.11E-04	0.0729
Stem volume	Potom.001G00161-SNP12	2.49E-04	0.0742
Stem volume	Potom.008G22699-SNP181	2.05E-04	0.0753
Holocellulose content	Potom.016G34352-SNP166	8.41E-04	0.07545
Stem volume	Potom.002G05522-SNP23	1.00E-03	0.07545
Hemicellulose content	Potom.002G07206-SNP63	2.16E-04	0.0756
Stem volume	Potom.016G33876-SNP17	1.87E-04	0.07615
Fiber width	Potom.001G03606-SNP44	3.55E-04	0.0763
Hemicellulose content	Potom.012G28819-SNP45	1.00E-03	0.0764
Fiber width	Potom.005G13398-SNP25	2.83E-04	0.07655
Lignin content	Potom.005G13176-SNP177	2.17E-04	0.0767
Fiber width	Potom.002G07206-SNP46	2.42E-04	0.0767
Tree height	Potom.013G29538-SNP97	1.00E-03	0.0769
Diameter at breast height	Potom.002G07963-SNP32	2.01E-04	0.07695
Diameter at breast height	Potom.005G14995-SNP78	2.01E-04	0.077
Holocellulose content	Potom.004G11193-SNP36	7.41E-04	0.07775
Diameter at breast height	Potom.005G13966-SNP63	2.52E-04	0.0782
Fiber width	Potom.018G36550-SNP25	9.92E-04	0.07855
Microfiber angle	Potom.005G13176-SNP233	1.00E-03	0.0787
Holocellulose content	Potom.004G11193-SNP30	1.00E-03	0.0787
Stem volume	Potom.001G03353-SNP137	1.43E-04	0.0787
Diameter at breast height	Potom.018G36550-SNP68	1.57E-04	0.07935
Hemicellulose content	Potom.002G05522-SNP73	9.01E-04	0.07935
Fiber width	Potom.005G13966-SNP59	2.21E-04	0.0796
Diameter at breast height	Potom.005G13966-SNP44	1.52E-04	0.0796
α-cellulose content	Potom.002G05522-SNP24	9.87E-04	0.07965
Diameter at breast height	Potom.005G14995-SNP68	1.47E-04	0.07995
Diameter at breast height	Potom.002G06086-SNP27	3.97E-04	0.07995
Microfiber angle	Potom.005G13176-SNP275	8.90E-04	0.08025
Diameter at breast height	Potom.008G22699-SNP173	7.42E-04	0.0804
Stem volume	Potom.008G19885-SNP26	1.18E-04	0.0805
Diameter at breast height	Potom.005G13985-SNP28	1.79E-04	0.08055
Diameter at breast height	Potom.005G13966-SNP9	1.33E-04	0.0809
Diameter at breast height	Potom.008G22699-SNP137	6.92E-04	0.0811
Diameter at breast height	Potom.004G11193-SNP65	3.10E-04	0.0818
Diameter at breast height	Potom.008G22699-SNP54	6.04E-04	0.0819
Diameter at breast height	Potom.016G33891-SNP27	5.91E-04	0.08275
Diameter at breast height	Potom.016G33891-SNP20	1.57E-04	0.08275
Stem volume	Potom.002G05769-SNP3	5.24E-04	0.08275
Diameter at breast height	Potom.008G22699-SNP96	1.41E-04	0.0828

Diameter at breast height	Potom.015G33266-SNP33	8.87E-04	0.08285
Diameter at breast height	Potom.005G13966-SNP97	5.82E-04	0.0829
Fiber width	Potom.016G33891-SNP5	1.36E-04	0.083
Diameter at breast height	Potom.008G22699-SNP55	5.49E-04	0.0831
Diameter at breast height	Potom.016G34352-SNP77	1.04E-04	0.08325
Diameter at breast height	Potom.008G22699-SNP18	6.14E-04	0.0835
Fiber width	Potom.016G33891-SNP4	1.12E-04	0.08355
Fiber width	Potom.016G33891-SNP3	1.12E-04	0.08355
Hemicellulose content	Potom.008G22699-SNP107	6.29E-04	0.0836
Holocellulose content	Potom.005G13176-SNP22	5.56E-04	0.085
Holocellulose content	Potom.005G13176-SNP21	5.56E-04	0.085
Diameter at breast height	Potom.005G13966-SNP18	7.86E-05	0.0859
Fiber width	Potom.009G23017-SNP55	4.54E-04	0.08595
Fiber width	Potom.008G22699-SNP80	4.87E-04	0.08595
Diameter at breast height	Potom.010G25398-SNP22	5.02E-04	0.08615
Diameter at breast height	Potom.016G34352-SNP83	6.44E-04	0.0863
Stem volume	Potom.018G36220-SNP1	1.00E-03	0.08635
Fiber width	Potom.008G22699-SNP81	4.93E-04	0.08695
Diameter at breast height	Potom.005G13966-SNP91	8.74E-05	0.0871
Fiber width	Potom.016G33891-SNP2	4.16E-04	0.0874
Fiber width	Potom.010G25706-SNP19	5.04E-04	0.08755
Diameter at breast height	Potom.008G22699-SNP56	8.39E-05	0.08765
Diameter at breast height	Potom.016G33891-SNP19	7.93E-05	0.088
Diameter at breast height	Potom.001G03193-SNP31	9.17E-04	0.0881
Diameter at breast height	Potom.016G34352-SNP76	3.44E-04	0.0882
Diameter at breast height	Potom.016G34352-SNP162	3.28E-04	0.08865
Holocellulose content	Potom.013G29538-SNP102	4.81E-05	0.08905
Diameter at breast height	Potom.008G22699-SNP58	5.16E-05	0.0898
Diameter at breast height	Potom.008G22699-SNP162	5.16E-05	0.0898
Diameter at breast height	Potom.005G14995-SNP11	5.16E-05	0.0898
Diameter at breast height	Potom.016G33891-SNP21	5.23E-05	0.0913
Diameter at breast height	Potom.015G33266-SNP32	2.89E-04	0.09145
α-cellulose content	Potom.001G03606-SNP23	2.55E-04	0.092
Stem volume	Potom.016G33876-SNP21	3.10E-05	0.09285
Holocellulose content	Potom.004G11193-SNP35	1.27E-04	0.09345
Fiber width	Potom.016G33891-SNP8	2.37E-04	0.0942
Diameter at breast height	Potom.008G22699-SNP152	5.83E-04	0.0948
Diameter at breast height	Potom.008G22699-SNP88	1.66E-04	0.0954
Diameter at breast height	Potom.001G03193-SNP30	4.08E-04	0.09625
Diameter at breast height	Potom.005G13176-SNP282	6.25E-05	0.097
Diameter at breast height	Potom.005G13176-SNP281	6.09E-05	0.0972
Diameter at breast height	Potom.016G34352-SNP86	4.69E-05	0.09725
Diameter at breast height	Potom.008G22699-SNP83	1.81E-04	0.09775
Holocellulose content	Potom.003G09473-SNP4	8.21E-04	0.09775

Diameter at breast height	Potom.002G07963-SNP75	5.09E-04	0.09785
Diameter at breast height	Potom.008G22699-SNP64	1.19E-04	0.09865
Diameter at breast height	Potom.008G22699-SNP167	1.19E-04	0.09865
Stem volume	Potom.008G22699-SNP71	1.32E-04	0.0997
Diameter at breast height	Potom.008G22699-SNP68	1.49E-04	0.1004
Diameter at breast height	Potom.016G33891-SNP18	3.38E-05	0.1038
Diameter at breast height	Potom.008G22699-SNP85	1.15E-04	0.105
α-cellulose content	Potom.008G22699-SNP107	8.46E-05	0.10515
Stem volume	Potom.014G31981-SNP32	1.30E-04	0.1052
Stem volume	Potom.014G31981-SNP30	1.30E-04	0.1052
Stem volume	Potom.013G29538-SNP1	2.72E-04	0.1053
Stem volume	Potom.014G31981-SNP34	1.30E-04	0.10535
Stem volume	Potom.003G09473-SNP21	6.97E-05	0.1054
Diameter at breast height	Potom.016G34352-SNP149	1.17E-04	0.10615
Fiber width	Potom.002G07963-SNP255	8.70E-05	0.1065
Diameter at breast height	Potom.008G22699-SNP164	1.03E-04	0.10745
Fiber length	Potom.005G13985-SNP25	4.47E-05	0.1075
Stem volume	Potom.001G03193-SNP33	1.99E-04	0.10765
Holocellulose content	Potom.003G09473-SNP5	4.24E-04	0.10915
Holocellulose content	Potom.003G09473-SNP6	5.03E-04	0.11005
Stem volume	Potom.014G31981-SNP33	8.50E-05	0.11075
Stem volume	Potom.014G31981-SNP31	6.90E-05	0.11165
Fiber width	Potom.009G23017-SNP54	3.48E-05	0.115
Diameter at breast height	Potom.003G09473-SNP25	9.62E-05	0.1157
Stem volume	Potom.014G31981-SNP35	3.98E-05	0.1163
α-cellulose content	Potom.015G32682-SNP107	4.27E-06	0.1182
Stem volume	Potom.014G31981-SNP36	4.47E-05	0.11825
Diameter at breast height	Potom.008G22699-SNP63	1.39E-05	0.1192
Holocellulose content	Potom.004G11193-SNP24	1.04E-04	0.1222
Diameter at breast height	Potom.008G22699-SNP71	1.47E-05	0.12305
Diameter at breast height	Potom.018G36220-SNP1	3.50E-05	0.12635
Fiber width	Potom.016G33891-SNP16	2.52E-06	0.12915
Fiber width	Potom.016G33891-SNP15	2.70E-05	0.13225
Fiber width	Potom.005G14995-SNP52	3.38E-07	0.13555
Stem volume	Potom.002G07963-SNP75	1.67E-05	0.13595
Fiber width	Potom.005G14995-SNP54	3.56E-06	0.1465
Fiber width	Potom.016G33891-SNP17	3.59E-06	0.1509
Fiber width	Pto-Wuschela-SNP3	9.83E-07	0.1515
Fiber length	Pto-Wuschela-SNP17	1.30E-04	0.07735
Tree height	Pto-Wuschela-SNP13	6.30E-04	0.0425

Table S4 Single significant results of additive effects and dominant effects for each SNP identified by epiSNP package.

Chr	Locus	Trait	Test	Effect	P value	Allele/ Geno	A/G Effect	A/G Frequency						
20	Potom.003G08321-SNP26	Microfiber angle	a	1.65	0.0019	1	1.12	0.323	2	-0.533	0.677			
20	Potom.003G08321-SNP1	Diameter at breast height	a	-4.84	0.00385	2	4.14	0.145	1	-0.7	0.855			
20	Potom.003G08321-SNP12	Diameter at breast height	a	4.48	0.00222	1	0.459	0.898	2	-4.02	0.102			
20	Potom.003G08321-SNP13	Diameter at breast height	a	-4.32	0.00674	2	3.59	0.169	1	-0.729	0.831			
20	Potom.003G08321-SNP13	Diameter at breast height	d	9.15	0.00572	12	7.19	0.0375	11	0.48	0.812	22	-4.4	0.15
20	Potom.003G08321-SNP14	Diameter at breast height	d	9.88	0.00505	12	7.62	0.0375	11	0.0643	0.887	22	-4.57	0.075
20	Potom.003G08321-SNP15	Diameter at breast height	a	4.08	0.00797	1	3.61	0.114	2	-0.465	0.886			
20	Potom.003G08321-SNP26	Diameter at breast height	a	-2.38	0.00269	2	0.768	0.677	1	-1.61	0.323			
20	Potom.003G08321-SNP38	Diameter at breast height	a	-4.41	0.00761	2	3.64	0.175	1	-0.774	0.825			
20	Potom.003G08321-SNP4	Diameter at breast height	a	-4.89	0.0038	2	4.15	0.15	1	-0.733	0.85			
20	Potom.003G09473-SNP1	Diameter at breast height	a	-4.91	0.00187	2	4.05	0.175	1	-0.859	0.825			
20	Potom.003G09473-	Diameter at	a	-4.81	0.0057	2	4.24	0.118	1	-0.57	0.882			

	SNP10	breast height									
20	Potom.003G09473-SNP12	Diameter at breast height	a	-4.74	0.00623	2	4.18	0.118	1	-0.561	0.882
20	Potom.003G09473-SNP16	Diameter at breast height	a	-6.08	0.000186	2	5.05	0.169	1	-1.03	0.831
20	Potom.003G09473-SNP21	Diameter at breast height	a	-7.34	0.00027	2	6.84	0.0679	1	-0.498	0.932
20	Potom.003G09473-SNP23	Diameter at breast height	a	-4.94	0.000221	2	3.57	0.278	1	-1.38	0.722
20	Potom.003G09473-SNP25	Diameter at breast height	a	-4.35	0.000409	2	2.87	0.342	1	-1.49	0.658
20	Potom.003G09473-SNP3	Diameter at breast height	a	7.18	0.000658	1	0.42	0.942	2	-6.76	0.0584
20	Potom.003G09473-SNP7	Diameter at breast height	a	-4.06	0.00416	2	3.13	0.229	1	-0.927	0.771
20	Potom.003G10089-SNP16	Diameter at breast height	a	-4.69	0.00203	2	3.72	0.207	1	-0.972	0.793
20	Potom.003G10089-SNP17	Diameter at breast height	a	-4.53	0.00247	2	3.58	0.21	1	-0.95	0.79
20	Potom.003G10089-SNP19	Diameter at breast height	a	-4.48	0.00254	2	3.55	0.207	1	-0.93	0.793
20	Potom.003G10089-SNP20	Diameter at breast height	a	-4.64	0.00209	2	3.69	0.205	1	-0.951	0.795
20	Potom.003G10089-SNP27	Diameter at breast height	a	-2.46	0.000425	2	0.836	0.661	1	-1.63	0.339
20	Potom.003G10130-	Diameter at	a	-5.08	0.00335	2	4.34	0.146	1	-0.742	0.854

	SNP11	breast height												
20	Potom.003G10130-SNP13	Diameter at breast height	a	-4.53	0.00169	2	3.51	0.226	1	-1.02	0.774			
20	Potom.003G10130-SNP14	Diameter at breast height	a	3.2	0.00946	1	0.494	0.846	2	-2.71	0.154			
20	Potom.003G10130-SNP15	Diameter at breast height	a	1.86	0.00415	1	0.695	0.627	2	-1.17	0.373			
20	Potom.003G10130-SNP4	Diameter at breast height	a	-5.26	3.03E-05	2	3.52	0.331	1	-1.74	0.669			
20	Potom.003G09473-SNP15	Fiber length	d	-0.116	0.00638	22	0.188	0.0122	11	0.00285	0.768	12	-0.0204	0.22
20	Potom.003G09473-SNP21	Fiber length	d	0.11	0.00792	12	0.0614	0.0854	11	-0.00333	0.89	22	-0.0935	0.0244
20	Potom.003G09473-SNP7	Fiber length	d	-0.113	0.00457	22	0.00875	0.211	11	0.00562	0.732	12	-0.106	0.0563
20	Potom.003G10089-SNP8	Tree height	d	-4.46	0.00736	22	1.41	0.0395	11	0.0984	0.921	12	-3.7	0.0395
20	Potom.003G08321-SNP28	Hemicellulose content	a	-5.07	0.0061	2	4.42	0.129	1	-0.656	0.871			
20	Potom.003G08321-SNP31	Hemicellulose content	a	-6.89	0.000382	2	6.07	0.119	1	-0.82	0.881			
20	Potom.003G09473-SNP1	Hemicellulose content	d	16.2	0.00764	12	16	0.0247	22	0.0691	0.173	11	-0.507	0.802
20	Potom.003G10130-SNP70	Hemicellulose content	a	1.28	0.00789	1	0.609	0.524	2	-0.67	0.476			
20	Potom.003G09473-	Holocellulose	d	-29.7	0.000499	22	6.02	0.132	11	-0.471	0.853	1	-26.9	0.014

	SNP4	content										2		7
20	Potom.003G09473-SNP5	Holocellulose content	d	-29.3	0.000543	22	7.39	0.121	11	-0.584	0.864	12	-25.8	0.0152
20	Potom.003G09473-SNP6	Holocellulose content	d	-29.3	0.000642	22	7.43	0.125	11	-0.61	0.859	12	-25.9	0.0156
20	Potom.003G10130-SNP3	Holocellulose content	a	7.33	0.00922	1	1.15	0.843	2	-6.19	0.157			
20	Potom.003G10130-SNP31	Holocellulose content	a	2.07	0.00554	1	1.16	0.441	2	-0.914	0.559			
20	Potom.003G08321-SNP1	Stem volume	a	-0.363	0.00212	2	0.311	0.145	1	-0.0525	0.855			
20	Potom.003G08321-SNP13	Stem volume	a	-0.319	0.00312	2	0.265	0.169	1	-0.0538	0.831			
20	Potom.003G08321-SNP13	Stem volume	d	0.846	0.0002	12	0.69	0.0375	11	0.0317	0.812	22	-0.344	0.15
20	Potom.003G08321-SNP14	Stem volume	d	0.891	0.000227	12	0.692	0.0375	11	0.00477	0.887	22	-0.402	0.075
20	Potom.003G08321-SNP15	Stem volume	a	0.331	0.00165	1	0.294	0.114	2	-0.0377	0.886			
20	Potom.003G08321-SNP3	Stem volume	a	0.216	0.0073	1	0.141	0.348	2	-0.075	0.652			
20	Potom.003G08321-SNP33	Stem volume	a	-0.223	0.00812	2	0.147	0.344	1	-0.0768	0.656			
20	Potom.003G08321-SNP37	Stem volume	a	-0.27	0.0065	2	0.201	0.256	1	-0.0693	0.744			
20	Potom.003G08321-	Stem volume	a	-0.356	0.0018	2	0.293	0.175	1	-0.0623	0.825			

	SNP38													
20	Potom.003G08321-SNP4	Stem volume	a	-0.37	0.00158	2	0.315	0.15	1	-0.0555	0.85			
20	Potom.003G09473-SNP1	Stem volume	a	-0.317	0.00423	2	0.261	0.175	1	-0.0555	0.825			
20	Potom.003G09473-SNP10	Stem volume	d	0.492	0.00665	12	0.335	0.0526	11	0.0146	0.855	22	-0.327	0.0921
20	Potom.003G09473-SNP12	Stem volume	d	0.489	0.00751	12	0.335	0.0526	11	0.0142	0.855	22	-0.323	0.0921
20	Potom.003G09473-SNP16	Stem volume	a	-0.358	0.00172	2	0.298	0.169	1	-0.0606	0.831			
20	Potom.003G09473-SNP21	Stem volume	a	-0.579	2.79E-05	2	0.54	0.0679	1	-0.0393	0.932			
20	Potom.003G09473-SNP23	Stem volume	a	-0.324	0.000611	2	0.234	0.278	1	-0.0902	0.722			
20	Potom.003G09473-SNP25	Stem volume	a	-0.294	0.000827	2	0.194	0.342	1	-0.1	0.658			
20	Potom.003G09473-SNP4	Stem volume	a	-0.403	0.00266	2	0.346	0.14	1	-0.0562	0.86			
20	Potom.003G09473-SNP6	Stem volume	a	-0.357	0.00906	2	0.31	0.133	1	-0.0474	0.867			
20	Potom.003G10089-SNP16	Stem volume	a	-0.311	0.00386	2	0.247	0.207	1	-0.0645	0.793			
20	Potom.003G10089-SNP17	Stem volume	a	-0.304	0.0047	2	0.24	0.21	1	-0.0638	0.79			
20	Potom.003G10089-	Stem volume	a	-0.303	0.00445	2	0.24	0.207	1	-0.0629	0.793			

	SNP19													
20	Potom.003G10089-SNP20	Stem volume	a	-0.31	0.00367	2	0.247	0.205	1	-0.0636	0.795			
20	Potom.003G10089-SNP27	Stem volume	a	-0.171	0.000519	2	0.0581	0.661	1	-0.113	0.339			
20	Potom.003G10130-SNP11	Stem volume	a	-0.324	0.00435	2	0.277	0.146	1	-0.0474	0.854			
20	Potom.003G10130-SNP13	Stem volume	a	-0.306	0.00327	2	0.237	0.226	1	-0.0691	0.774			
20	Potom.003G10130-SNP4	Stem volume	a	-0.354	0.000111	2	0.237	0.331	1	-0.117	0.669			
20	Potom.003G09473-SNP14	Fiber width	a	-2.21	0.000785	2	2.04	0.0769	1	-0.17	0.923			
20	Potom.003G09473-SNP15	Fiber width	a	-1.35	0.00567	2	1.19	0.122	1	-0.165	0.878			
20	Potom.003G09473-SNP17	Fiber width	a	-1.9	0.00219	2	1.68	0.118	1	-0.225	0.882			
20	Potom.003G09473-SNP18	Fiber width	a	-1.97	0.00245	2	1.74	0.115	1	-0.226	0.885			
20	Potom.003G09473-SNP19	Fiber width	a	-1.9	0.00364	2	1.68	0.115	1	-0.218	0.885			
20	Potom.003G09473-SNP20	Fiber width	a	-1.87	0.00471	2	1.65	0.118	1	-0.22	0.882			
20	Potom.003G10089-SNP6	Fiber width	a	-1.4	0.00504	2	1.24	0.116	1	-0.162	0.884			
20	Potom.003G09473-	α-cellulose	d	-19.1	0.00146	22	3.37	0.173	11	-0.188	0.802	1	-17.5	0.024

	SNP1	content										2		7
18	Potom.018G36220-SNP41	Microfiber angle	d	-4.13	0.00782	22	5.89	0.0238	11	1.56	0.131	12	-0.408	0.845
18	Potom.018G35976-SNP31	Diameter at breast height	a	2.31	0.00737	1	0.578	0.75	2	-1.73	0.25			
18	Potom.018G35976-SNP32	Diameter at breast height	a	1.65	0.00756	1	0.61	0.631	2	-1.04	0.369			
18	Potom.018G36220-SNP1	Diameter at breast height	a	-4.58	0.000013	2	2.51	0.451	1	-2.06	0.549			
18	Potom.018G36220-SNP26	Diameter at breast height	a	-4.22	0.00532	2	3.81	0.0952	1	-0.401	0.905			
18	Potom.018G36220-SNP42	Diameter at breast height	a	-1.95	0.00456	2	0.752	0.614	1	-1.2	0.386			
18	Potom.018G36220-SNP43	Diameter at breast height	a	-2.04	0.00239	2	0.799	0.608	1	-1.24	0.392			
18	Potom.018G36220-SNP78	Diameter at breast height	a	-4.41	0.000722	2	3.88	0.119	1	-0.525	0.881			
18	Potom.018G36550-SNP12	Diameter at breast height	a	5	0.000903	1	0.482	0.904	2	-4.51	0.0964			
18	Potom.018G36550-SNP57	Diameter at breast height	a	-4.71	0.00586	2	4.34	0.0774	1	-0.364	0.923			
18	Potom.018G36550-SNP63	Diameter at breast height	a	-2.56	0.00016	2	0.853	0.667	1	-1.71	0.333			
18	Potom.018G35976-SNP12	Tree height	a	0.543	0.00617	1	0.243	0.554	2	-0.301	0.446			
18	Potom.018G35976-	Tree height	d	-2.44	0.00461	22	3.59	0.037	11	0.482	0.287	1	-0.405	0.675

	SNP13							5				2		
18	Potom.018G36220-SNP81	Hemicellulose content	d	9.27	0.00256	12	0.873	0.906	22	-7.69	0.0471	11	-9.12	0.0471
18	Potom.018G36550-SNP56	Hemicellulose content	a	2.77	0.00869	1	0.888	0.679	2	-1.88	0.321			
18	Potom.018G36220-SNP78	Holocellulose content	a	5.65	0.00964	1	0.698	0.876	2	-4.95	0.124			
18	Potom.018G36220-SNP8	Holocellulose content	a	8.89	0.00108	1	0.732	0.918	2	-8.16	0.0824			
18	Potom.018G36220-SNP81	Holocellulose content	d	9.2	0.00954	12	0.866	0.906	11	-8.21	0.0471	22	-8.46	0.0471
18	Potom.018G35976-SNP13	Stem volume	d	-0.425	0.00157	22	0.636	0.0375	11	0.0776	0.287	12	-0.0684	0.675
18	Potom.018G36220-SNP1	Stem volume	a	-0.307	0.000117	2	0.169	0.451	1	-0.138	0.549			
18	Potom.018G36220-SNP14	Stem volume	d	-0.433	0.00628	11	0.688	0.0238	22	0.0981	0.167	12	-0.0404	0.81
18	Potom.018G36220-SNP43	Stem volume	a	-0.127	0.00783	2	0.0497	0.608	1	-0.0772	0.392			
18	Potom.018G36220-SNP78	Stem volume	a	-0.271	0.00342	2	0.238	0.119	1	-0.0322	0.881			
18	Potom.018G36220-SNP8	Stem volume	a	-0.316	0.00854	2	0.292	0.0774	1	-0.0245	0.923			
18	Potom.018G36550-SNP63	Stem volume	a	-0.17	0.000383	2	0.0567	0.667	1	-0.113	0.333			
18	Potom.018G36220-	Fiber width	d	2.43	0.00293	12	0.196	0.812	22	-0.384	0.165	1	-4.09	0.023

	SNP14											1		5
18	Potom.018G36550-SNP25	Fiber width	d	3.48	0.0049	12	0.95	0.0952	11	-0.0343	0.893	22	-5.02	0.0119
16	Potom.016G33876-SNP56	Microfiber angle	a	0.886	0.00703	1	0.417	0.53	2	-0.47	0.47			
16	Potom.016G33891-SNP2	Microfiber angle	d	-5.95	0.00711	22	9.09	0.0119	11	0.12	0.833	12	-1.35	0.155
16	Potom.016G33876-SNP10	Diameter at breast height	a	-4.99	0.00853	2	4.64	0.0714	1	-0.357	0.929			
16	Potom.016G33876-SNP56	Diameter at breast height	a	-1.31	0.00455	2	0.686	0.476	1	-0.623	0.524			
16	Potom.016G33876-SNP71	Diameter at breast height	d	4.96	0.00928	12	1	0.716	11	-2.02	0.247	22	-5.89	0.037
16	Potom.016G33876-SNP72	Diameter at breast height	a	-2.75	0.00112	2	1.29	0.532	1	-1.46	0.468			
16	Potom.016G33876-SNP81	Diameter at breast height	a	-4.91	0.00785	2	4.59	0.0655	1	-0.321	0.935			
16	Potom.016G33876-SNP98	Diameter at breast height	a	-4.91	0.00785	2	4.59	0.0655	1	-0.321	0.935			
16	Potom.016G33891-SNP15	Diameter at breast height	a	-3.54	0.0047	2	2.32	0.345	1	-1.22	0.655			
16	Potom.016G33891-SNP17	Diameter at breast height	a	-4.82	0.000221	2	3.37	0.301	1	-1.45	0.699			
16	Potom.016G33891-SNP26	Diameter at breast height	a	-3.36	0.00145	2	1.9	0.434	1	-1.46	0.566			
16	Potom.016G33891-	Diameter at	a	-4.81	0.00143	2	4.01	0.167	1	-0.801	0.833			

	SNP27	breast height												
16	Potom.016G33891-SNP29	Diameter at breast height	a	-4.1	0.00306	2	3.17	0.226	1	-0.925	0.774			
16	Potom.016G34352-SNP103	Diameter at breast height	a	2.39	0.00303	1	0.655	0.726	2	-1.74	0.274			
16	Potom.016G34352-SNP162	Diameter at breast height	a	-4.44	7.25E-05	2	3.7	0.167	1	-0.741	0.833			
16	Potom.016G34352-SNP168	Diameter at breast height	a	-4.15	0.000172	2	3.46	0.167	1	-0.692	0.833			
16	Potom.016G34352-SNP234	Diameter at breast height	a	-4.46	0.00943	2	3.98	0.107	1	-0.478	0.893			
16	Potom.016G34352-SNP76	Diameter at breast height	a	-4.48	4.31E-05	2	3.76	0.161	1	-0.72	0.839			
16	Potom.016G34352-SNP82	Diameter at breast height	a	-4.21	0.000185	2	3.56	0.155	1	-0.651	0.845			
16	Potom.016G34352-SNP83	Diameter at breast height	a	-4.28	0.000322	2	3.61	0.156	1	-0.669	0.844			
16	Potom.016G34352-SNP85	Diameter at breast height	a	-4.1	0.000176	2	3.23	0.212	1	-0.87	0.788			
16	Potom.016G34352-SNP93	Diameter at breast height	a	-4.68	2.25E-05	2	3.86	0.175	1	-0.82	0.825			
16	Potom.016G34352-SNP98	Diameter at breast height	d	-4.22	0.00953	11	4.04	0.0595	22	2.65	0.167	12	-0.881	0.774
16	Potom.016G33876-SNP107	Fiber length	d	0.0882	0.00462	12	0.00981	0.847	11	-0.0436	0.129	22	-0.113	0.0235
16	Potom.016G33876-	Fiber length	d	0.0737	0.00536	12	0.0113	0.812	11	-0.0403	0.153	2	-0.084	0.035

	SNP108											2	7	3
16	Potom.016G33876-SNP112	Fiber length	d	0.0793	0.00905	12	0.0113	0.706	11	-0.0193	0.271	22	-0.117	0.0235
16	Potom.016G33876-SNP46	Hemicellulose content	d	9.07	0.00821	12	0.671	0.882	11	-2.79	0.0941	22	-14	0.0235
16	Potom.016G33891-SNP6	Hemicellulose content	d	7.79	0.00509	12	5.56	0.141	11	-0.418	0.741	22	-4.04	0.118
16	Potom.016G33876-SNP27	Holocellulose content	a	10.7	0.00571	1	0.573	0.946	2	-10.1	0.0536			
16	Potom.016G33876-SNP46	Holocellulose content	d	12.8	0.00086	12	0.756	0.882	11	-1.43	0.0941	22	-22.6	0.0235
16	Potom.016G34352-SNP162	Holocellulose content	a	5.03	0.00713	1	0.858	0.829	2	-4.17	0.171			
16	Potom.016G34352-SNP166	Holocellulose content	a	-1.73	0.0011	2	0.883	0.488	1	-0.842	0.512			
16	Potom.016G34352-SNP76	Holocellulose content	a	4.81	0.00736	1	0.793	0.835	2	-4.02	0.165			
16	Potom.016G33876-SNP38	Lignin content	a	-0.542	0.00334	2	0.297	0.452	1	-0.245	0.548			
16	Potom.016G34352-SNP110	Lignin content	a	0.491	0.0012	1	0.245	0.5	2	-0.245	0.5			
16	Potom.016G34352-SNP201	Lignin content	d	2.66	0.00609	12	0.214	0.906	11	-1.67	0.0706	22	-3.23	0.0235
16	Potom.016G34352-SNP230	Lignin content	a	0.294	0.00736	1	0.147	0.5	2	-0.147	0.5			
16	Potom.016G34352-	Lignin content	d	4.41	0.00939	12	4.02	0.027	11	-0.0677	0.905	2	-0.701	0.067

	SNP49											2		6
16	Potom.016G33876-SNP27	Stem volume	a	-0.487	0.00207	2	0.46	0.0542	1	-0.0264	0.946			
16	Potom.016G33876-SNP31	Stem volume	d	-0.29	0.00562	11	0.282	0.0964	22	0.174	0.12	12	-0.0615	0.783
16	Potom.016G33876-SNP38	Stem volume	d	-0.436	0.00418	22	0.877	0.0241	12	-0.0185	0.843	11	-0.0419	0.133
16	Potom.016G33876-SNP47	Stem volume	a	-0.0804	0.00937	2	0.0369	0.542	1	-0.0436	0.458			
16	Potom.016G33876-SNP55	Stem volume	a	-0.0783	0.00858	2	0.0391	0.5	1	-0.0391	0.5			
16	Potom.016G33876-SNP56	Stem volume	a	-0.104	0.00108	2	0.0543	0.476	1	-0.0493	0.524			
16	Potom.016G33876-SNP72	Stem volume	a	-0.176	0.00356	2	0.0826	0.532	1	-0.0938	0.468			
16	Potom.016G33876-SNP81	Stem volume	a	-0.336	0.00912	2	0.314	0.0655	1	-0.022	0.935			
16	Potom.016G33876-SNP83	Stem volume	a	-0.357	0.00906	2	0.335	0.0602	1	-0.0215	0.94			
16	Potom.016G33876-SNP98	Stem volume	a	-0.336	0.00912	2	0.314	0.0655	1	-0.022	0.935			
16	Potom.016G33891-SNP15	Stem volume	a	-0.253	0.00403	2	0.166	0.345	1	-0.087	0.655			
16	Potom.016G33891-SNP17	Stem volume	a	-0.325	0.000455	2	0.227	0.301	1	-0.0981	0.699			
16	Potom.016G33891-	Stem volume	a	-0.238	0.00139	2	0.135	0.434	1	-0.103	0.566			

	SNP26													
16	Potom.016G33891-SNP27	Stem volume	a	-0.323	0.00224	2	0.269	0.167	1	-0.0538	0.833			
16	Potom.016G33891-SNP7	Stem volume	a	-0.35	0.00563	2	0.305	0.128	1	-0.0448	0.872			
16	Potom.016G34352-SNP103	Stem volume	a	0.157	0.00599	1	0.0429	0.726	2	-0.114	0.274			
16	Potom.016G34352-SNP162	Stem volume	a	-0.279	0.000414	2	0.232	0.167	1	-0.0465	0.833			
16	Potom.016G34352-SNP168	Stem volume	a	-0.263	0.000741	2	0.219	0.167	1	-0.0439	0.833			
16	Potom.016G34352-SNP234	Stem volume	a	-0.311	0.00957	2	0.278	0.107	1	-0.0333	0.893			
16	Potom.016G34352-SNP76	Stem volume	a	-0.292	0.000168	2	0.245	0.161	1	-0.047	0.839			
16	Potom.016G34352-SNP82	Stem volume	a	-0.27	0.000704	2	0.228	0.155	1	-0.0417	0.845			
16	Potom.016G34352-SNP83	Stem volume	a	-0.266	0.00142	2	0.224	0.156	1	-0.0416	0.844			
16	Potom.016G34352-SNP85	Stem volume	a	-0.281	0.000492	2	0.222	0.212	1	-0.0596	0.788			
16	Potom.016G34352-SNP93	Stem volume	a	-0.312	5.35E-05	2	0.257	0.175	1	-0.0547	0.825			
16	Potom.016G34352-SNP98	Stem volume	d	-0.304	0.00823	11	0.31	0.0595	22	0.174	0.167	12	-0.0614	0.774
16	Potom.016G33891-	Fiber width	d	2.67	1.81E-05	12	2.1	0.187	11	-0.332	0.547	2	-0.794	0.267

	SNP15											2		
16	Potom.016G33891-SNP17	Fiber width	d	3.47	7.22E-07	12	2.9	0.123	11	-0.199	0.63	22	-0.942	0.247
16	Potom.016G33891-SNP2	Fiber width	d	2.94	0.01	12	0.624	0.153	11	-0.0497	0.835	22	-4.58	0.0118
16	Potom.016G33891-SNP2	Fiber width	a	-1.56	0.00486	2	1.42	0.0882	1	-0.137	0.912			
16	Potom.016G33891-SNP28	Fiber width	d	2.33	0.0015	12	0.726	0.298	11	-0.161	0.667	22	-3.05	0.0357
16	Potom.016G33891-SNP8	Fiber width	a	-2.82	0.000064	2	2.65	0.0595	1	-0.168	0.94			
16	Potom.016G34352-SNP41	Fiber width	d	6.21	0.00271	12	6.07	0.0123	11	0.257	0.568	22	-0.527	0.42
16	Potom.016G33891-SNP17	α-cellulose content	a	5.63	0.00721	1	1.74	0.692	2	-3.89	0.308			
14	Potom.014G31981-SNP25	Microfiber angle	d	-3.8	0.00254	22	1.28	0.139	11	0.44	0.696	12	-2.94	0.165
14	Potom.014G31876-SNP18	Diameter at breast height	a	3.58	0.00605	1	0.453	0.873	2	-3.13	0.127			
14	Potom.014G31981-SNP22	Diameter at breast height	a	2.03	0.0096	1	0.579	0.714	2	-1.45	0.286			
14	Potom.014G31981-SNP27	Diameter at breast height	a	6.47	0.00359	1	0.324	0.95	2	-6.15	0.05			
14	Potom.014G31981-SNP30	Diameter at breast height	d	16	0.00327	12	14.3	0.0132	11	0.0917	0.908	22	-3.44	0.0789
14	Potom.014G31981-	Diameter at	d	15.3	0.00396	12	13.3	0.013	11	0.315	0.882	2	-4.3	0.105

	SNP31	breast height						2				2		
14	Potom.014G31981-SNP32	Diameter at breast height	d	16	0.00327	12	14.3	0.0132	11	0.0917	0.908	22	-3.44	0.0789
14	Potom.014G31981-SNP33	Diameter at breast height	d	15.4	0.00411	12	13.3	0.0133	11	0.265	0.893	22	-4.44	0.0933
14	Potom.014G31981-SNP34	Diameter at breast height	d	16	0.00309	12	14.3	0.0132	11	0.0969	0.908	22	-3.5	0.0789
14	Potom.014G31981-SNP35	Diameter at breast height	d	15.1	0.00468	12	12.5	0.013	11	0.203	0.922	22	-5.39	0.0649
14	Potom.014G31981-SNP36	Diameter at breast height	d	15.2	0.00494	12	12.6	0.0133	11	0.214	0.92	22	-5.46	0.0667
14	Potom.014G31981-SNP39	Diameter at breast height	a	-5.83	0.00254	2	5.19	0.11	1	-0.644	0.89			
14	Potom.014G31981-SNP44	Diameter at breast height	a	-4.68	0.00515	2	3.94	0.158	1	-0.74	0.842			
14	Potom.014G31981-SNP78	Diameter at breast height	a	-3.86	0.00406	2	0.541	0.86	1	-3.32	0.14			
14	Potom.014G31981-SNP30	Stem volume	d	1.79	1.45E-06	12	1.65	0.0132	11	0.00197	0.908	22	-0.297	0.0789
14	Potom.014G31981-SNP31	Stem volume	d	1.75	1.74E-06	12	1.59	0.0132	11	0.0159	0.882	22	-0.332	0.105
14	Potom.014G31981-SNP32	Stem volume	d	1.79	1.45E-06	12	1.65	0.0132	11	0.00197	0.908	22	-0.297	0.0789
14	Potom.014G31981-SNP33	Stem volume	d	1.75	2.16E-06	12	1.57	0.0133	11	0.0142	0.893	22	-0.361	0.0933
14	Potom.014G31981-	Stem volume	d	1.8	1.42E-06	12	1.65	0.013	11	0.00234	0.908	2	-0.301	0.078

	SNP34							2				2		9
14	Potom.014G31981-SNP35	Stem volume	a	-0.394	0.00654	2	0.366	0.0714	1	-0.0282	0.929			
14	Potom.014G31981-SNP35	Stem volume	d	1.7	2.66E-06	12	1.46	0.013	11	0.0153	0.922	22	-0.509	0.0649
14	Potom.014G31981-SNP36	Stem volume	a	-0.399	0.00653	2	0.37	0.0733	1	-0.0293	0.927			
14	Potom.014G31981-SNP36	Stem volume	d	1.71	3.27E-06	12	1.46	0.0133	11	0.016	0.92	22	-0.513	0.0667
14	Potom.014G31981-SNP39	Stem volume	a	-0.471	0.000759	2	0.419	0.11	1	-0.0519	0.89			
14	Potom.014G31981-SNP40	Stem volume	a	-0.345	0.00543	2	0.294	0.146	1	-0.0502	0.854			
14	Potom.014G31981-SNP41	Stem volume	a	-0.345	0.00543	2	0.294	0.146	1	-0.0502	0.854			
14	Potom.014G31981-SNP44	Stem volume	a	-0.319	0.00754	2	0.269	0.158	1	-0.0505	0.842			
14	Potom.014G31981-SNP26	Fiber width	d	1.59	0.00998	12	1.21	0.15	11	-0.0642	0.65	22	-0.698	0.2
14	Potom.014G31981-SNP61	Fiber width	d	-3.21	0.00304	22	5.29	0.0119	11	0.0742	0.762	12	-0.528	0.226
14	Potom.014G31981-SNP80	Fiber width	d	7.06	0.000747	12	6.93	0.012	11	-0.0757	0.892	22	-0.166	0.0964
14	Potom.014G31981-SNP81	Fiber width	d	7.12	0.000629	12	7.07	0.0119	22	0.0104	0.107	11	-0.0968	0.881
13	Potom.013G29538-	Diameter at	a	0.931	0.00279	1	0.471	0.494	2	-0.46	0.506			

	SNP103	breast height												
13	Potom.013G29538-SNP135	Diameter at breast height	a	-1.37	0.00561	2	0.622	0.548	1	-0.753	0.452			
13	Potom.013G29538-SNP2	Fiber length	d	-0.0578	0.00666	22	0.062	0.0759	11	0.0201	0.291	12	-0.0167	0.633
13	Potom.013G29538-SNP1	Tree height	d	-4.54	0.00407	22	6.4	0.0139	11	0.106	0.847	12	-1.28	0.139
13	Potom.013G29538-SNP41	Tree height	d	2.71	0.00916	12	0.218	0.917	11	-1.86	0.0476	22	-3.12	0.0357
13	Potom.013G29538-SNP100	Hemicellulose content	a	-1.14	0.00913	2	0.565	0.506	1	-0.578	0.494			
13	Potom.013G29538-SNP12	Hemicellulose content	a	8.79	0.00957	1	0.573	0.935	2	-8.22	0.0652			
13	Potom.013G29538-SNP13	Hemicellulose content	a	8.88	0.00798	1	0.563	0.937	2	-8.32	0.0634			
13	Potom.013G29538-SNP130	Hemicellulose content	d	-12.8	0.00365	22	23.6	0.0118	11	0.935	0.165	12	-0.524	0.824
13	Potom.013G29538-SNP131	Hemicellulose content	d	-12.8	0.00365	22	23.6	0.0118	11	0.935	0.165	12	-0.524	0.824
13	Potom.013G29538-SNP136	Hemicellulose content	d	12.6	0.00439	12	0.414	0.859	22	-0.583	0.129	11	-23.8	0.0118
13	Potom.013G29538-SNP37	Hemicellulose content	a	-4.69	0.00879	2	4	0.147	1	-0.69	0.853			
13	Potom.013G29538-SNP100	Holocellulose content	d	12.9	0.00327	12	0.65	0.941	22	-3.09	0.0353	11	-21.4	0.0235
13	Potom.013G29538-	Lignin content	d	-3.6	0.00293	11	7.12	0.011	12	-0.0839	0.859	2	-0.090	0.129

	SNP136							8				2	8	
13	Potom.013G29538-SNP1	Stem volume	d	-0.958	1.63E-05	22	1.31	0.0139	11	0.026	0.847	12	-0.289	0.139
13	Potom.013G29538-SNP10	Stem volume	d	-0.392	0.00611	22	0.331	0.058	11	0.0404	0.71	12	-0.206	0.232
13	Potom.013G29538-SNP103	Stem volume	a	0.0673	0.00204	1	0.0341	0.494	2	-0.0333	0.506			
13	Potom.013G29538-SNP11	Stem volume	d	-0.373	0.00788	22	0.265	0.0563	11	0.0385	0.746	12	-0.221	0.197
13	Potom.013G29538-SNP135	Stem volume	a	-0.0977	0.00513	2	0.0442	0.548	1	-0.0535	0.452			
13	Potom.013G29538-SNP2	Stem volume	d	-0.343	0.00113	22	0.461	0.0769	11	0.0611	0.282	12	-0.0822	0.641
13	Potom.013G29538-SNP4	Stem volume	d	-0.273	0.00484	22	0.319	0.11	11	0.0847	0.183	12	-0.0714	0.707
13	Potom.013G29538-SNP1	Fiber width	d	3.5	0.00688	12	1.01	0.137	11	-0.0835	0.849	22	-4.9	0.0137
13	Potom.013G29538-SNP100	α-cellulose content	d	13.1	0.00107	12	0.763	0.941	22	-11.7	0.0353	11	-12.9	0.0235
13	Potom.013G29538-SNP103	α-cellulose content	d	10.8	0.00791	12	0.589	0.939	22	-4.42	0.0366	11	-16	0.0244
13	Potom.013G29538-SNP117	α-cellulose content	d	5.9	0.00688	12	1.42	0.753	22	-3.45	0.141	11	-5.5	0.106
13	Potom.013G29538-SNP129	α-cellulose content	a	6.57	0.00518	1	0.58	0.912	2	-5.99	0.0882			
13	Potom.013G29538-	α-cellulose	d	13.3	0.0035	12	0.744	0.824	11	-2.08	0.165	2	-23.1	0.011

	SNP130	content										2		8
13	Potom.013G29538-SNP131	α-cellulose content	d	13.3	0.0035	12	0.744	0.824	11	-2.08	0.165	22	-23.1	0.0118
12	Potom.012G28409-SNP7	Diameter at breast height	d	-7.26	0.006	22	8.82	0.0244	11	0.144	0.854	12	-2.77	0.122
12	Potom.012G28819-SNP3	Diameter at breast height	a	4.52	0.00271	1	0.431	0.905	2	-4.09	0.0952			
12	Potom.012G28819-SNP8	Diameter at breast height	a	3.68	0.0093	1	0.603	0.836	2	-3.07	0.164			
12	Potom.012G28409-SNP7	Tree height	d	-3.92	0.00154	22	4.4	0.0244	11	0.112	0.854	12	-1.66	0.122
12	Potom.012G28819-SNP45	Hemicellulose content	d	8.77	0.000197	12	5.04	0.247	11	-0.96	0.659	22	-6.51	0.0941
12	Potom.012G28409-SNP7	Stem volume	d	-0.511	0.00709	22	0.595	0.0244	11	0.0126	0.854	12	-0.207	0.122
12	Potom.012G28819-SNP39	Fiber width	d	6.24	0.00293	12	5.79	0.0118	11	0.00784	0.906	22	-0.913	0.0824
10	Potom.010G25700-SNP15	Microfiber angle	a	-4.68	0.00472	2	4.37	0.0676	1	-0.316	0.932			
10	Potom.010G24967-SNP67	Diameter at breast height	a	1.71	0.000519	1	0.835	0.513	2	-0.879	0.487			
10	Potom.010G24967-SNP68	Diameter at breast height	a	1.25	0.00644	1	0.578	0.539	2	-0.676	0.461			
10	Potom.010G25294-SNP115	Diameter at breast height	a	-1.62	0.00416	2	0.635	0.607	1	-0.982	0.393			
10	Potom.010G25294-	Diameter at	a	-1.69	0.0039	2	0.675	0.601	1	-1.02	0.399			

	SNP152	breast height												
10	Potom.010G25398-SNP22	Diameter at breast height	d	12.6	0.000176	12	3.8	0.0952	11	-0.171	0.893	22	-17.5	0.0119
10	Potom.010G25398-SNP61	Diameter at breast height	a	-4.87	0.00205	2	4.04	0.171	1	-0.832	0.829			
10	Potom.010G25398-SNP62	Diameter at breast height	a	-5.83	1.66E-05	2	4.21	0.278	1	-1.62	0.722			
10	Potom.010G25398-SNP65	Diameter at breast height	a	-4.32	4.51E-05	2	2.5	0.421	1	-1.82	0.579			
10	Potom.010G25398-SNP69	Diameter at breast height	a	-5.37	1.46E-05	2	3.59	0.331	1	-1.78	0.669			
10	Potom.010G25700-SNP19	Diameter at breast height	a	-5.25	0.00295	2	0.492	0.906	1	-4.76	0.0938			
10	Potom.010G25700-SNP2	Diameter at breast height	a	4.85	0.00174	1	0.491	0.899	2	-4.36	0.101			
10	Potom.010G25700-SNP34	Diameter at breast height	a	-5.34	0.000833	2	4.48	0.161	1	-0.858	0.839			
10	Potom.010G25700-SNP45	Diameter at breast height	a	3.81	0.00665	1	0.452	0.881	2	-3.36	0.119			
10	Potom.010G25700-SNP7	Diameter at breast height	a	-4.79	0.00433	2	4.04	0.157	1	-0.75	0.843			
10	Potom.010G25706-SNP59	Diameter at breast height	a	-4.4	0.00624	2	3.59	0.184	1	-0.807	0.816			
10	Potom.010G24967-SNP85	Tree height	d	3.82	0.00981	12	0.184	0.94	11	-2.43	0.0476	22	-4.84	0.0119
10	Potom.010G25398-	Tree height	d	3.95	0.00459	12	3.26	0.048	11	-0.0535	0.867	2	-1.32	0.084

	SNP63							2				2		3
10	Potom.010G25706-SNP69	Tree height	a	0.564	0.00288	1	0.255	0.548	2	-0.309	0.452			
10	Potom.010G25398-SNP5	Hemicellulose content	a	-1.99	0.00933	2	1.09	0.453	1	-0.901	0.547			
10	Potom.010G25398-SNP5	Holocellulose content	a	-2.84	0.000873	2	1.55	0.453	1	-1.29	0.547			
10	Potom.010G25398-SNP58	Holocellulose content	a	7.64	0.00985	1	1.06	0.862	2	-6.59	0.138			
10	Potom.010G25398-SNP63	Holocellulose content	a	8.82	0.00331	1	0.997	0.887	2	-7.82	0.113			
10	Potom.010G25398-SNP65	Holocellulose content	d	7.28	0.00607	12	5.95	0.193	22	-0.817	0.325	11	-1.83	0.482
10	Potom.010G25398-SNP68	Holocellulose content	a	10.3	0.000958	1	0.996	0.904	2	-9.34	0.0964			
10	Potom.010G25398-SNP70	Holocellulose content	a	9.72	0.00105	1	1.33	0.863	2	-8.39	0.137			
10	Potom.010G24967-SNP68	Lignin content	a	0.558	0.00285	1	0.254	0.545	2	-0.304	0.455			
10	Potom.010G25398-SNP26	Lignin content	d	1.54	0.00774	12	0.852	0.296	11	-0.205	0.593	22	-1.18	0.111
10	Potom.010G25700-SNP34	Lignin content	d	-4.63	0.00518	11	0.14	0.835	22	-0.0656	0.141	12	-4.59	0.0235
10	Potom.010G24967-SNP67	Stem volume	a	0.123	0.000279	1	0.0598	0.513	2	-0.063	0.487			
10	Potom.010G25294-	Stem volume	a	-0.122	0.00209	2	0.0479	0.607	1	-0.074	0.393			

	SNP115													
10	Potom.010G25294-SNP152	Stem volume	a	-0.132	0.00134	2	0.0526	0.601	1	-0.0793	0.399			
10	Potom.010G25398-SNP22	Stem volume	d	0.795	0.000712	12	0.226	0.0952	11	-0.009	0.893	22	-1.13	0.0119
10	Potom.010G25398-SNP62	Stem volume	a	-0.382	9.04E-05	2	0.275	0.278	1	-0.106	0.722			
10	Potom.010G25398-SNP65	Stem volume	a	-0.273	0.000348	2	0.158	0.421	1	-0.115	0.579			
10	Potom.010G25398-SNP69	Stem volume	a	-0.351	0.000085	2	0.235	0.331	1	-0.116	0.669			
10	Potom.010G25398-SNP70	Stem volume	a	-0.351	0.00722	2	0.306	0.128	1	-0.0449	0.872			
10	Potom.010G25700-SNP34	Stem volume	a	-0.294	0.00999	2	0.247	0.161	1	-0.0472	0.839			
10	Potom.010G25706-SNP59	Stem volume	a	-0.323	0.00513	2	0.264	0.184	1	-0.0593	0.816			
10	Potom.010G25706-SNP69	Stem volume	a	0.0811	0.00535	1	0.0367	0.548	2	-0.0444	0.452			
10	Potom.010G25706-SNP87	Stem volume	d	-0.476	0.00213	22	0.867	0.0247	11	0.0143	0.259	12	-0.0351	0.716
10	Potom.010G25294-SNP123	Fiber width	a	-1.17	0.00584	2	0.997	0.147	1	-0.172	0.853			
10	Potom.010G25700-SNP13	Fiber width	d	2.43	0.00192	12	1.54	0.119	11	-0.075	0.81	22	-1.72	0.0714
10	Potom.010G25700-	Fiber width	a	-1.19	0.00859	2	1.03	0.129	1	-0.154	0.871			

	SNP37													
10	Potom.010G25706-SNP19	Fiber width	a	-1.39	0.00265	2	1.2	0.135	1	-0.188	0.865			
10	Potom.010G25706-SNP38	Fiber width	a	-2.96	0.000709	2	2.74	0.0758	1	-0.224	0.924			
10	Potom.010G25706-SNP40	Fiber width	d	2.47	0.00494	12	1.24	0.132	11	-0.0829	0.829	22	-2.38	0.0395
10	Potom.010G25706-SNP88	Fiber width	a	-1.34	0.00568	2	1.18	0.122	1	-0.163	0.878			
10	Potom.010G25294-SNP215	α-cellulose content	a	7.53	0.00236	1	0.665	0.912	2	-6.87	0.0882			
9	Potom.009G23017-SNP56	Diameter at breast height	a	-5.34	0.00497	2	4.76	0.108	1	-0.579	0.892			
9	Potom.009G23017-SNP61	Diameter at breast height	a	-6.48	0.00213	2	5.92	0.0854	1	-0.553	0.915			
9	Potom.009G22902-SNP13	Hemicellulose content	d	9.11	0.00818	12	2.31	0.271	11	-0.446	0.706	22	-13.2	0.0235
9	Potom.009G22902-SNP14	Hemicellulose content	d	9.11	0.00818	12	2.31	0.271	11	-0.446	0.706	22	-13.2	0.0235
9	Potom.009G22902-SNP25	Hemicellulose content	d	14.4	0.00301	12	3.69	0.129	11	-0.267	0.859	22	-21.1	0.0118
9	Potom.009G22902-SNP29	Hemicellulose content	a	6.71	0.0035	1	0.592	0.912	2	-6.12	0.0882			
9	Potom.009G22902-SNP55	Hemicellulose content	d	10.4	0.00492	12	3.81	0.165	11	-0.401	0.812	22	-12.9	0.0235
9	Potom.009G22902-	Hemicellulose	d	9.72	0.0045	12	2.47	0.271	11	-0.478	0.706	2	-14	0.023

	SNP57	content									2		5
9	Potom.009G23017-SNP43	Hemicellulose content	d	-11.9	0.00801	22	23.3	0.0118	11	-0.0888	0.0941	12	-0.297	0.894
9	Potom.009G23029-SNP3	Holocellulose content	d	9.58	0.00227	12	1.18	0.679	11	-0.814	0.286	22	-16	0.0357
9	Potom.009G23017-SNP56	Stem volume	a	-0.373	0.00612	2	0.332	0.108	1	-0.0404	0.892			
9	Potom.009G23017-SNP61	Stem volume	a	-0.482	0.00138	2	0.44	0.0854	1	-0.0411	0.915			
9	Potom.009G22902-SNP63	Fiber width	a	-1.26	0.00856	2	1.11	0.119	1	-0.15	0.881			
9	Potom.009G23017-SNP51	Fiber width	d	2.4	0.00629	12	1	0.123	11	-0.0255	0.84	22	-2.77	0.037
9	Potom.009G23017-SNP52	Fiber width	d	2.4	0.00629	12	1	0.123	11	-0.0255	0.84	22	-2.77	0.037
9	Potom.009G23017-SNP53	Fiber width	d	2.51	0.00788	12	0.842	0.134	11	-0.0387	0.841	22	-3.3	0.0244
9	Potom.009G23017-SNP54	Fiber width	d	3.38	0.000126	12	1.59	0.111	11	-0.0545	0.852	22	-3.52	0.037
9	Potom.009G23017-SNP55	Fiber width	d	3.21	0.000331	12	1.6	0.106	11	-0.0668	0.859	22	-3.16	0.0353
9	Potom.009G23017-SNP60	Fiber width	a	-2.19	0.00458	2	2.04	0.0649	1	-0.142	0.935			
9	Potom.009G23029-SNP1	Fiber width	a	-1.41	0.00148	2	1.22	0.137	1	-0.193	0.863			
8	Potom.008G21952-	Microfiber	a	-3.56	0.0074	2	3.29	0.075	1	-0.268	0.925			

	SNP43	angle						3						
8	Potom.008G22699-SNP70	Microfiber angle	d	4.32	0.00794	12	3.83	0.0732	11	0.2	0.585	22	-1.16	0.341
8	Potom.008G21326-SNP101	Diameter at breast height	a	6.26	0.00668	1	0.313	0.95	2	-5.95	0.05			
8	Potom.008G21952-SNP1	Diameter at breast height	d	10.1	0.00367	12	6.52	0.0482	11	-0.156	0.928	22	-7.02	0.0241
8	Potom.008G21952-SNP25	Diameter at breast height	d	10.3	0.00127	12	2.3	0.155	11	-0.202	0.833	22	-15.8	0.0119
8	Potom.008G21952-SNP43	Diameter at breast height	a	4.75	0.00423	1	0.39	0.918	2	-4.36	0.0822			
8	Potom.008G22516-SNP45	Diameter at breast height	a	3.81	0.00544	1	0.431	0.887	2	-3.38	0.113			
8	Potom.008G22516-SNP5	Diameter at breast height	a	4.65	0.00919	1	0.332	0.929	2	-4.32	0.0714			
8	Potom.008G22516-SNP7	Diameter at breast height	a	-4.65	0.00231	2	3.76	0.19	1	-0.886	0.81			
8	Potom.008G22699-SNP103	Diameter at breast height	a	-2.44	0.000505	2	0.828	0.661	1	-1.61	0.339			
8	Potom.008G22699-SNP137	Diameter at breast height	d	10.8	0.000512	12	2.3	0.167	22	-0.223	0.821	11	-16.8	0.0119
8	Potom.008G22699-SNP14	Diameter at breast height	a	-4.93	0.00435	2	4.25	0.139	1	-0.683	0.861			
8	Potom.008G22699-SNP148	Diameter at breast height	a	3.91	0.00443	1	0.442	0.887	2	-3.47	0.113			
8	Potom.008G22699-	Diameter at	a	-4.91	0.00479	2	4.26	0.133	1	-0.651	0.867			

	SNP15	breast height												
8	Potom.008G22699-SNP152	Diameter at breast height	a	-5.62	8.01E-05	2	4.39	0.219	1	-1.23	0.781			
8	Potom.008G22699-SNP153	Diameter at breast height	a	-2.4	0.00447	2	0.874	0.636	1	-1.53	0.364			
8	Potom.008G22699-SNP154	Diameter at breast height	a	0.902	0.00313	1	0.445	0.506	2	-0.456	0.494			
8	Potom.008G22699-SNP16	Diameter at breast height	a	-5.03	0.00363	2	4.36	0.134	1	-0.675	0.866			
8	Potom.008G22699-SNP164	Diameter at breast height	a	-4.32	0.000055	2	3.6	0.167	1	-0.72	0.833			
8	Potom.008G22699-SNP167	Diameter at breast height	a	-4.05	0.000118	2	3.37	0.167	1	-0.675	0.833			
8	Potom.008G22699-SNP168	Diameter at breast height	a	-2.41	0.000312	2	0.817	0.661	1	-1.59	0.339			
8	Potom.008G22699-SNP17	Diameter at breast height	a	-5.31	0.00216	2	4.56	0.14	1	-0.745	0.86			
8	Potom.008G22699-SNP170	Diameter at breast height	a	-2.49	0.00019	2	0.845	0.661	1	-1.65	0.339			
8	Potom.008G22699-SNP172	Diameter at breast height	a	-2.15	0.00222	2	0.729	0.661	1	-1.42	0.339			
8	Potom.008G22699-SNP173	Diameter at breast height	a	-2.73	0.000113	2	0.909	0.667	1	-1.82	0.333			
8	Potom.008G22699-SNP18	Diameter at breast height	d	11.6	0.0031	12	9.43	0.0244	11	0.333	0.866	22	-4.72	0.11
8	Potom.008G22699-	Diameter at	a	-4.93	0.00435	2	4.25	0.139	1	-0.683	0.861			

	SNP19	breast height												
8	Potom.008G22699-SNP190	Diameter at breast height	a	-4.38	0.000117	2	3.57	0.185	1	-0.808	0.815			
8	Potom.008G22699-SNP192	Diameter at breast height	d	5.87	0.00868	12	0.884	0.524	11	-0.526	0.452	22	-9.45	0.0238
8	Potom.008G22699-SNP2	Diameter at breast height	d	6.53	0.00677	12	3.27	0.0952	11	-0.00182	0.857	22	-6.51	0.0476
8	Potom.008G22699-SNP20	Diameter at breast height	a	-4.94	0.0046	2	4.25	0.14	1	-0.692	0.86			
8	Potom.008G22699-SNP22	Diameter at breast height	a	-3.7	0.00735	2	3	0.189	1	-0.7	0.811			
8	Potom.008G22699-SNP23	Diameter at breast height	a	-4.5	0.00914	2	3.93	0.127	1	-0.569	0.873			
8	Potom.008G22699-SNP3	Diameter at breast height	a	-2.58	0.00161	2	1.5	0.421	1	-1.09	0.579			
8	Potom.008G22699-SNP38	Diameter at breast height	a	-5.32	0.00308	2	4.75	0.107	1	-0.57	0.893			
8	Potom.008G22699-SNP54	Diameter at breast height	a	-4.69	0.000155	2	3.25	0.307	1	-1.44	0.693			
8	Potom.008G22699-SNP55	Diameter at breast height	a	-4.89	0.000124	2	3.39	0.307	1	-1.5	0.693			
8	Potom.008G22699-SNP57	Diameter at breast height	a	5.38	0.00449	1	0.361	0.933	2	-5.02	0.0671			
8	Potom.008G22699-SNP63	Diameter at breast height	d	-9.73	0.00111	11	1.75	0.651	22	-2.46	0.313	12	-10.1	0.0361
8	Potom.008G22699-	Diameter at	a	-4.69	6.71E-05	2	3.13	0.331	1	-1.55	0.669			

	SNP63	breast height												
8	Potom.008G22699-SNP64	Diameter at breast height	a	-5.54	1.39E-05	2	3.79	0.315	1	-1.75	0.685			
8	Potom.008G22699-SNP68	Diameter at breast height	a	-5.35	1.61E-05	2	3.54	0.338	1	-1.8	0.662			
8	Potom.008G22699-SNP69	Diameter at breast height	a	-5.71	1.53E-05	2	4.12	0.278	1	-1.59	0.722			
8	Potom.008G22699-SNP70	Diameter at breast height	a	-4.25	0.00052	2	2.67	0.37	1	-1.57	0.63			
8	Potom.008G22699-SNP71	Diameter at breast height	a	-5.45	3.13E-05	2	4.33	0.206	1	-1.12	0.794			
8	Potom.008G22699-SNP72	Diameter at breast height	a	-4.63	0.000162	2	3.86	0.167	1	-0.771	0.833			
8	Potom.008G22699-SNP75	Diameter at breast height	a	-4.28	0.000081	2	3.6	0.161	1	-0.689	0.839			
8	Potom.008G22699-SNP83	Diameter at breast height	a	-5.51	2.66E-05	2	3.77	0.315	1	-1.73	0.685			
8	Potom.008G22699-SNP85	Diameter at breast height	a	-5.35	2.89E-05	2	3.73	0.304	1	-1.62	0.696			
8	Potom.008G22699-SNP88	Diameter at breast height	a	-5.5	0.000017	2	3.77	0.315	1	-1.74	0.685			
8	Potom.008G22699-SNP89	Diameter at breast height	a	-5.76	6.27E-05	2	4.24	0.263	1	-1.51	0.737			
8	Potom.008G22699-SNP98	Diameter at breast height	a	-6.11	0.000929	2	5.42	0.113	1	-0.688	0.887			
8	Potom.008G21952-	Fiber length	d	0.0922	0.00505	12	0.024	0.262	11	-0.0044	0.714	2	-0.132	0.023

	SNP29											2		8
8	Potom.008G21952-SNP30	Fiber length	d	0.0922	0.00505	12	0.024	0.262	11	-0.0044	0.714	22	-0.132	0.0238
8	Potom.008G21326-SNP45	Hemicellulose content	a	-5.2	0.00524	2	4.56	0.124	1	-0.643	0.876			
8	Potom.008G21952-SNP43	Hemicellulose content	a	6.96	0.00745	1	0.557	0.92	2	-6.4	0.08			
8	Potom.008G21952-SNP44	Hemicellulose content	d	-11.1	0.00183	22	15	0.0235	11	0.465	0.765	12	-3.34	0.212
8	Potom.008G22699-SNP107	Hemicellulose content	d	-11.4	0.00254	22	11.3	0.0241	11	0.534	0.843	12	-5.45	0.133
8	Potom.008G21952-SNP25	Holocellulose content	a	7.08	0.00523	1	0.666	0.906	2	-6.41	0.0941			
8	Potom.008G21952-SNP32	Holocellulose content	a	9.89	0.0058	1	0.916	0.907	2	-8.97	0.0926			
8	Potom.008G21952-SNP37	Holocellulose content	a	13.8	0.000235	1	0.986	0.929	2	-12.8	0.0714			
8	Potom.008G21952-SNP38	Holocellulose content	a	13.8	0.000235	1	0.986	0.929	2	-12.8	0.0714			
8	Potom.008G22699-SNP17	Holocellulose content	d	-31	0.00128	22	1.88	0.133	11	0.132	0.855	12	-30	0.012
8	Potom.008G21952-SNP1	Stem volume	d	0.741	0.00241	12	0.446	0.0482	11	-0.00802	0.928	22	-0.583	0.0241
8	Potom.008G21952-SNP25	Stem volume	d	0.605	0.00689	12	0.132	0.155	11	-0.0112	0.833	22	-0.935	0.0119
8	Potom.008G22516-	Stem volume	a	-0.316	0.0035	2	0.256	0.19	1	-0.0603	0.81			

	SNP7													
8	Potom.008G22699-SNP103	Stem volume	a	-0.16	0.00124	2	0.0543	0.661	1	-0.106	0.339			
8	Potom.008G22699-SNP137	Stem volume	d	0.583	0.00909	12	0.123	0.167	22	-0.0118	0.821	11	-0.908	0.0119
8	Potom.008G22699-SNP14	Stem volume	a	-0.34	0.0064	2	0.293	0.139	1	-0.047	0.861			
8	Potom.008G22699-SNP15	Stem volume	a	-0.338	0.00686	2	0.293	0.133	1	-0.0448	0.867			
8	Potom.008G22699-SNP152	Stem volume	a	-0.317	0.000626	2	0.247	0.219	1	-0.0695	0.781			
8	Potom.008G22699-SNP153	Stem volume	a	-0.172	0.00426	2	0.0625	0.636	1	-0.109	0.364			
8	Potom.008G22699-SNP154	Stem volume	a	0.0663	0.00186	1	0.0328	0.506	2	-0.0336	0.494			
8	Potom.008G22699-SNP16	Stem volume	a	-0.35	0.00434	2	0.303	0.134	1	-0.0469	0.866			
8	Potom.008G22699-SNP164	Stem volume	a	-0.278	0.000247	2	0.232	0.167	1	-0.0463	0.833			
8	Potom.008G22699-SNP167	Stem volume	a	-0.263	0.00043	2	0.219	0.167	1	-0.0439	0.833			
8	Potom.008G22699-SNP168	Stem volume	a	-0.162	0.000588	2	0.0549	0.661	1	-0.107	0.339			
8	Potom.008G22699-SNP17	Stem volume	a	-0.355	0.00366	2	0.305	0.14	1	-0.0498	0.86			
8	Potom.008G22699-	Stem volume	a	-0.166	0.000423	2	0.0563	0.661	1	-0.11	0.339			

	SNP170													
8	Potom.008G22699-SNP172	Stem volume	a	-0.148	0.00272	2	0.0504	0.661	1	-0.0981	0.339			
8	Potom.008G22699-SNP173	Stem volume	a	-0.185	0.000208	2	0.0616	0.667	1	-0.123	0.333			
8	Potom.008G22699-SNP18	Stem volume	d	1.03	0.00023	12	0.876	0.0244	11	0.0156	0.866	22	-0.317	0.11
8	Potom.008G22699-SNP19	Stem volume	a	-0.34	0.0064	2	0.293	0.139	1	-0.047	0.861			
8	Potom.008G22699-SNP190	Stem volume	a	-0.283	0.000452	2	0.23	0.185	1	-0.0521	0.815			
8	Potom.008G22699-SNP2	Stem volume	d	0.44	0.00993	12	0.223	0.0952	11	-0.000687	0.857	22	-0.433	0.0476
8	Potom.008G22699-SNP20	Stem volume	a	-0.34	0.00673	2	0.292	0.14	1	-0.0477	0.86			
8	Potom.008G22699-SNP23	Stem volume	d	0.626	0.00735	12	0.479	0.0361	11	0.0195	0.855	22	-0.313	0.108
8	Potom.008G22699-SNP3	Stem volume	a	-0.179	0.00226	2	0.104	0.421	1	-0.0753	0.579			
8	Potom.008G22699-SNP38	Stem volume	a	-0.365	0.00399	2	0.326	0.107	1	-0.0391	0.893			
8	Potom.008G22699-SNP54	Stem volume	a	-0.273	0.000642	2	0.189	0.307	1	-0.084	0.693			
8	Potom.008G22699-SNP55	Stem volume	a	-0.312	0.000529	2	0.216	0.307	1	-0.0957	0.693			
8	Potom.008G22699-	Stem volume	a	-0.318	0.000243	2	0.213	0.331	1	-0.105	0.669			

	SNP63													
8	Potom.008G22699-SNP64	Stem volume	a	-0.36	7.01E-05	2	0.246	0.315	1	-0.113	0.685			
8	Potom.008G22699-SNP68	Stem volume	a	-0.345	0.000109	2	0.229	0.338	1	-0.116	0.662			
8	Potom.008G22699-SNP69	Stem volume	a	-0.388	0.000034	2	0.28	0.278	1	-0.108	0.722			
8	Potom.008G22699-SNP70	Stem volume	a	-0.289	0.000668	2	0.182	0.37	1	-0.107	0.63			
8	Potom.008G22699-SNP71	Stem volume	d	0.375	0.00338	12	0.215	0.138	11	0.0242	0.725	22	-0.343	0.138
8	Potom.008G22699-SNP71	Stem volume	a	-0.341	0.000246	2	0.27	0.206	1	-0.0703	0.794			
8	Potom.008G22699-SNP72	Stem volume	a	-0.325	0.000171	2	0.271	0.167	1	-0.0542	0.833			
8	Potom.008G22699-SNP75	Stem volume	a	-0.274	0.000355	2	0.23	0.161	1	-0.0441	0.839			
8	Potom.008G22699-SNP83	Stem volume	a	-0.347	0.00019	2	0.238	0.315	1	-0.109	0.685			
8	Potom.008G22699-SNP85	Stem volume	a	-0.362	6.08E-05	2	0.252	0.304	1	-0.11	0.696			
8	Potom.008G22699-SNP88	Stem volume	a	-0.359	7.37E-05	2	0.246	0.315	1	-0.113	0.685			
8	Potom.008G22699-SNP89	Stem volume	a	-0.396	0.000105	2	0.292	0.263	1	-0.104	0.737			
8	Potom.008G22699-	Stem volume	a	-0.506	0.000121	2	0.449	0.113	1	-0.057	0.887			

	SNP98													
8	Potom.008G21326-SNP42	Fiber width	a	-1.15	0.00539	2	0.977	0.153	1	-0.176	0.847			
8	Potom.008G21326-SNP43	Fiber width	a	-1.17	0.00597	2	0.994	0.147	1	-0.171	0.853			
8	Potom.008G21326-SNP51	Fiber width	a	-1.23	0.0067	2	1.06	0.137	1	-0.168	0.863			
8	Potom.008G21326-SNP58	Fiber width	a	-1.15	0.00696	2	0.976	0.149	1	-0.171	0.851			
8	Potom.008G21952-SNP32	Fiber width	d	6.98	0.00102	12	6.72	0.0123	11	-0.0452	0.901	22	-0.488	0.0864
8	Potom.008G22516-SNP43	Fiber width	a	-1.97	0.0012	2	1.76	0.108	1	-0.212	0.892			
8	Potom.008G22699-SNP107	Fiber width	a	-1.62	0.00871	2	1.47	0.0904	1	-0.146	0.91			
8	Potom.008G22699-SNP149	Fiber width	a	-1.57	0.00171	2	1.38	0.119	1	-0.186	0.881			
8	Potom.008G22699-SNP80	Fiber width	a	-1.38	0.00342	2	1.22	0.118	1	-0.162	0.882			
8	Potom.008G22699-SNP81	Fiber width	a	-1.38	0.00365	2	1.22	0.119	1	-0.164	0.881			
8	Potom.008G21952-SNP16	α-cellulose content	a	7.03	0.00125	1	1.4	0.801	2	-5.63	0.199			
8	Potom.008G22699-SNP107	α-cellulose content	d	14.5	0.000185	12	6.77	0.133	11	-0.642	0.843	22	-14.8	0.0241
5	Potom.005G13176-	Microfiber	d	-5.96	0.00318	11	10.2	0.011	22	0.831	0.262	1	-0.466	0.726

	SNP126	angle						9				2		
5	Potom.005G13176-SNP233	Microfiber angle	d	-7.38	0.000213	11	12.8	0.0119	22	0.883	0.262	12	-0.529	0.726
5	Potom.005G13176-SNP275	Microfiber angle	d	-7.44	0.000185	11	12.8	0.0119	22	0.967	0.25	12	-0.535	0.738
5	Potom.005G13176-SNP277	Microfiber angle	d	-4.23	0.00494	11	6.43	0.0238	22	0.957	0.25	12	-0.54	0.726
5	Potom.005G13176-SNP279	Microfiber angle	d	-4.23	0.00494	11	6.43	0.0238	22	0.957	0.25	12	-0.54	0.726
5	Potom.005G13966-SNP5	Microfiber angle	a	-0.544	0.00751	2	0.282	0.482	1	-0.262	0.518			
5	Potom.005G13985-SNP25	Microfiber angle	d	12.8	0.000738	12	12.5	0.012	11	-0.0466	0.795	22	-0.589	0.193
5	Pto-Wuschela-SNP 18	Microfiber angle	a	-1	0.00584	2	0.487	0.513	1	-0.513	0.487			
5	Potom.005G13176-SNP117	Diameter at breast height	a	-1.96	0.0058	2	0.828	0.577	1	-1.13	0.423			
5	Potom.005G13176-SNP126	Diameter at breast height	a	-1.59	0.00921	2	0.595	0.625	1	-0.992	0.375			
5	Potom.005G13176-SNP129	Diameter at breast height	a	1.77	0.00265	1	0.676	0.619	2	-1.1	0.381			
5	Potom.005G13176-SNP131	Diameter at breast height	a	-3.96	0.00121	2	3.42	0.137	1	-0.542	0.863			
5	Potom.005G13176-SNP141	Diameter at breast height	a	-3.83	0.003	2	3.35	0.125	1	-0.479	0.875			
5	Potom.005G13176-	Diameter at	a	-3.87	0.002	2	3.31	0.143	1	-0.552	0.857			

	SNP147	breast height									
5	Potom.005G13176-SNP15	Diameter at breast height	a	-2.82	0.0059	2	2.19	0.223	1	-0.628	0.777
5	Potom.005G13176-SNP156	Diameter at breast height	a	-3.46	0.00638	2	3	0.131	1	-0.453	0.869
5	Potom.005G13176-SNP16	Diameter at breast height	a	-2.46	0.00804	2	1.39	0.438	1	-1.08	0.562
5	Potom.005G13176-SNP166	Diameter at breast height	a	-1.99	0.00436	2	0.827	0.583	1	-1.16	0.417
5	Potom.005G13176-SNP168	Diameter at breast height	a	-2.03	0.00229	2	0.844	0.583	1	-1.18	0.417
5	Potom.005G13176-SNP169	Diameter at breast height	a	-2.11	0.000385	2	0.83	0.607	1	-1.28	0.393
5	Potom.005G13176-SNP175	Diameter at breast height	a	-1.77	0.00366	2	0.685	0.613	1	-1.09	0.387
5	Potom.005G13176-SNP178	Diameter at breast height	a	-1.97	0.0024	2	0.785	0.601	1	-1.18	0.399
5	Potom.005G13176-SNP179	Diameter at breast height	a	-2.01	0.00147	2	0.788	0.607	1	-1.22	0.393
5	Potom.005G13176-SNP181	Diameter at breast height	a	-2.01	0.00147	2	0.788	0.607	1	-1.22	0.393
5	Potom.005G13176-SNP183	Diameter at breast height	a	-1.99	0.00311	2	0.771	0.613	1	-1.22	0.387
5	Potom.005G13176-SNP185	Diameter at breast height	a	-1.98	0.00257	2	0.754	0.619	1	-1.23	0.381
5	Potom.005G13176-	Diameter at	a	-2.09	0.000698	2	0.834	0.601	1	-1.26	0.399

	SNP194	breast height												
5	Potom.005G13176-SNP20	Diameter at breast height	a	-4.89	0.0015	2	4.01	0.179	1	-0.878	0.821			
5	Potom.005G13176-SNP21	Diameter at breast height	a	-5.58	0.000413	2	4.6	0.175	1	-0.979	0.825			
5	Potom.005G13176-SNP22	Diameter at breast height	a	-5.58	0.000413	2	4.6	0.175	1	-0.979	0.825			
5	Potom.005G13176-SNP225	Diameter at breast height	d	9.46	0.00181	12	1.6	0.25	11	-0.293	0.738	22	-15.4	0.0119
5	Potom.005G13176-SNP230	Diameter at breast height	d	9.46	0.00181	12	1.6	0.25	11	-0.293	0.738	22	-15.4	0.0119
5	Potom.005G13176-SNP233	Diameter at breast height	a	-1.71	0.00484	2	0.64	0.625	1	-1.07	0.375			
5	Potom.005G13176-SNP246	Diameter at breast height	a	1.58	0.0094	1	0.593	0.625	2	-0.988	0.375			
5	Potom.005G13176-SNP252	Diameter at breast height	a	2.06	0.000418	1	0.784	0.619	2	-1.27	0.381			
5	Potom.005G13176-SNP263	Diameter at breast height	a	-2.08	0.000397	2	0.791	0.619	1	-1.29	0.381			
5	Potom.005G13176-SNP275	Diameter at breast height	a	-1.79	0.00194	2	0.694	0.613	1	-1.1	0.387			
5	Potom.005G13176-SNP277	Diameter at breast height	a	-1.97	0.0012	2	0.764	0.613	1	-1.21	0.387			
5	Potom.005G13176-SNP279	Diameter at breast height	a	-1.97	0.0012	2	0.764	0.613	1	-1.21	0.387			
5	Potom.005G13176-	Diameter at	a	-2.95	0.00704	2	2.31	0.216	1	-0.638	0.784			

	SNP3	breast height												
5	Potom.005G13176-SNP30	Diameter at breast height	a	-2.17	0.00343	2	0.912	0.579	1	-1.26	0.421			
5	Potom.005G13176-SNP306	Diameter at breast height	a	-3.65	0.00397	2	3.19	0.125	1	-0.456	0.875			
5	Potom.005G13176-SNP71	Diameter at breast height	d	7.92	0.00904	12	0.555	0.869	11	-2.87	0.119	22	-11.9	0.0119
5	Potom.005G13176-SNP78	Diameter at breast height	a	-1.79	0.00627	2	0.659	0.631	1	-1.13	0.369			
5	Potom.005G13176-SNP83	Diameter at breast height	a	-3.72	0.00385	2	3.21	0.137	1	-0.509	0.863			
5	Potom.005G13333-SNP16	Diameter at breast height	a	4.25	0.00547	1	0.415	0.902	2	-3.84	0.0976			
5	Potom.005G13966-SNP100	Diameter at breast height	a	-3.89	0.00177	2	2.54	0.348	1	-1.35	0.652			
5	Potom.005G13966-SNP36	Diameter at breast height	a	-2.37	0.0007	2	0.853	0.639	1	-1.51	0.361			
5	Potom.005G13966-SNP74	Diameter at breast height	a	-3.92	0.001	2	2.49	0.364	1	-1.43	0.636			
5	Potom.005G13966-SNP89	Diameter at breast height	a	-5.03	9.12E-05	2	3.47	0.309	1	-1.56	0.691			
5	Potom.005G13966-SNP90	Diameter at breast height	a	-4.83	0.000586	2	3.67	0.24	1	-1.16	0.76			
5	Potom.005G13966-SNP92	Diameter at breast height	a	-5.62	1.62E-05	2	3.95	0.296	1	-1.66	0.704			
5	Potom.005G13966-	Diameter at	a	-5.14	2.34E-05	2	3.44	0.331	1	-1.7	0.669			

	SNP93	breast height												
5	Potom.005G13966-SNP97	Diameter at breast height	a	-4.95	8.41E-05	2	4.07	0.179	1	-0.884	0.821			
5	Potom.005G13985-SNP17	Diameter at breast height	a	-4.06	0.00811	2	3.3	0.187	1	-0.758	0.813			
5	Potom.005G13985-SNP20	Diameter at breast height	a	-4.1	0.00659	2	3.56	0.131	1	-0.537	0.869			
5	Potom.005G13985-SNP25	Diameter at breast height	a	-3.94	0.00653	2	3.11	0.211	1	-0.831	0.789			
5	Potom.005G13985-SNP26	Diameter at breast height	a	-4.12	0.00904	2	3.34	0.189	1	-0.779	0.811			
5	Potom.005G13985-SNP4	Diameter at breast height	a	-3.99	0.00959	2	3.24	0.187	1	-0.744	0.813			
5	Potom.005G13985-SNP6	Diameter at breast height	a	-4.26	0.00588	2	3.46	0.187	1	-0.796	0.813			
5	Potom.005G13985-SNP7	Diameter at breast height	a	-4.5	0.00367	2	3.6	0.199	1	-0.894	0.801			
5	Pto-Wuschela-SNP11	Diameter at breast height	a	0.95	0.01	1	0.506	0.468	2	-0.444	0.532			
5	Potom.005G13966-SNP5	Fiber length	a	0.0119	0.00339	1	0.00574	0.518	2	-0.00616	0.482			
5	Potom.005G13985-SNP25	Fiber length	d	-0.304	5.84E-05	22	0.0224	0.202	11	-0.00131	0.786	12	-0.294	0.0119
5	Potom.005G13985-SNP3	Fiber length	a	0.0671	0.01	1	0.00439	0.935	2	-0.0627	0.0655			
5	Pto-Wuschela-SNP	Fiber length	a	-0.04	0.000581	2	0.0296	0.26	1	-0.0104	0.74			

	17													
5	Potom.005G13176-SNP129	Hemicellulose content	d	-11.5	0.00798	22	22.6	0.0118	11	-0.145	0.259	12	-0.312	0.729
5	Potom.005G13176-SNP20	Hemicellulose content	d	-18.6	0.00212	22	3.13	0.177	11	-0.153	0.797	12	-17.1	0.0253
5	Potom.005G13176-SNP21	Hemicellulose content	d	-26.3	0.00197	22	2.45	0.179	11	-0.144	0.808	12	-25.2	0.0128
5	Potom.005G13176-SNP22	Hemicellulose content	d	-26.3	0.00197	22	2.45	0.179	11	-0.144	0.808	12	-25.2	0.0128
5	Potom.005G13176-SNP46	Hemicellulose content	a	-2.09	0.00622	2	1.2	0.424	1	-0.883	0.576			
5	Potom.005G13333-SNP7	Hemicellulose content	a	-7.64	0.00334	2	7.1	0.0706	1	-0.54	0.929			
5	Potom.005G13966-SNP52	Hemicellulose content	a	1.21	0.00535	1	0.582	0.518	2	-0.625	0.482			
5	Potom.005G13966-SNP88	Hemicellulose content	a	8.95	0.00361	1	0.53	0.941	2	-8.42	0.0592			
5	Potom.005G14995-SNP53	Hemicellulose content	a	1.99	0.000488	1	0.912	0.542	2	-1.08	0.458			
5	Potom.005G14995-SNP61	Hemicellulose content	d	-11.8	0.00784	22	23.2	0.0118	11	-0.162	0.118	12	-0.291	0.871
5	Potom.005G13176-SNP183	Holocellulose content	a	2.92	0.00773	1	1.8	0.382	2	-1.12	0.618			
5	Potom.005G13176-SNP185	Holocellulose content	a	2.86	0.0071	1	1.79	0.376	2	-1.08	0.624			
5	Potom.005G13176-	Holocellulose	a	7.1	0.00683	1	1.35	0.81	2	-5.75	0.19			

	SNP20	content												
5	Potom.005G13176-SNP20	Holocellulose content	d	-19.1	0.00464	22	6.81	0.177	11	-0.998	0.797	12	-16.2	0.0253
5	Potom.005G13176-SNP21	Holocellulose content	a	6.94	0.00774	1	1.29	0.814	2	-5.65	0.186			
5	Potom.005G13176-SNP21	Holocellulose content	d	-34.2	0.000256	22	6.72	0.179	11	-0.997	0.808	12	-31.3	0.0128
5	Potom.005G13176-SNP22	Holocellulose content	a	6.94	0.00774	1	1.29	0.814	2	-5.65	0.186			
5	Potom.005G13176-SNP22	Holocellulose content	d	-34.2	0.000256	22	6.72	0.179	11	-0.997	0.808	12	-31.3	0.0128
5	Potom.005G13176-SNP273	Holocellulose content	d	15.2	0.0039	12	0.378	0.929	11	-0.0854	0.0588	22	-29.5	0.0118
5	Potom.005G13176-SNP41	Holocellulose content	a	3.61	0.00959	1	1.94	0.463	2	-1.67	0.537			
5	Potom.005G13176-SNP62	Holocellulose content	a	3.59	0.00791	1	2.03	0.435	2	-1.56	0.565			
5	Potom.005G13176-SNP68	Holocellulose content	d	-8.7	0.0093	22	9.57	0.0471	11	0.39	0.753	12	-3.72	0.2
5	Potom.005G13398-SNP1	Holocellulose content	a	8.89	0.000844	1	0.878	0.901	2	-8.01	0.0988			
5	Potom.005G13398-SNP5	Holocellulose content	a	7.23	0.00617	1	1.32	0.818	2	-5.91	0.182			
5	Potom.005G13398-SNP64	Holocellulose content	a	8.18	0.00245	1	1.41	0.827	2	-6.77	0.173			
5	Potom.005G13966-	Holocellulose	d	19.3	0.00108	12	7.92	0.092	11	-0.486	0.895	2	-22.4	0.013

	SNP88	content						1				2		2
5	Potom.005G13966-SNP95	Holocellulose content	d	10.2	0.00576	12	0.731	0.702	11	-0.247	0.274	22	-18.7	0.0238
5	Potom.005G13966-SNP96	Holocellulose content	d	10.2	0.00576	12	0.731	0.702	11	-0.247	0.274	22	-18.7	0.0238
5	Pto-Wuschela-SNP 14	Holocellulose content	d	7.68	0.00922	12	1.22	0.788	22	-2.99	0.165	11	-9.93	0.0471
5	Potom.005G14995-SNP42	Lignin content	a	-0.861	0.00983	2	0.619	0.28	1	-0.241	0.72			
5	Potom.005G13176-SNP103	Stem volume	a	-0.111	0.00888	2	0.0418	0.625	1	-0.0697	0.375			
5	Potom.005G13176-SNP117	Stem volume	a	-0.153	0.00229	2	0.0645	0.577	1	-0.0881	0.423			
5	Potom.005G13176-SNP126	Stem volume	a	-0.12	0.00479	2	0.0452	0.625	1	-0.0753	0.375			
5	Potom.005G13176-SNP129	Stem volume	a	0.129	0.00187	1	0.0492	0.619	2	-0.0799	0.381			
5	Potom.005G13176-SNP137	Stem volume	a	-0.228	0.00853	2	0.197	0.137	1	-0.0312	0.863			
5	Potom.005G13176-SNP141	Stem volume	a	-0.276	0.00223	2	0.242	0.125	1	-0.0345	0.875			
5	Potom.005G13176-SNP155	Stem volume	a	-0.125	0.00751	2	0.0491	0.607	1	-0.0759	0.393			
5	Potom.005G13176-SNP156	Stem volume	a	-0.242	0.00609	2	0.21	0.131	1	-0.0317	0.869			
5	Potom.005G13176-	Stem volume	a	-0.129	0.00971	2	0.0547	0.577	1	-0.0748	0.423			

	SNP157										
5	Potom.005G13176-SNP16	Stem volume	a	-0.18	0.00611	2	0.101	0.438	1	-0.0789	0.562
5	Potom.005G13176-SNP166	Stem volume	a	-0.137	0.005	2	0.0572	0.583	1	-0.08	0.417
5	Potom.005G13176-SNP168	Stem volume	a	-0.14	0.00276	2	0.0582	0.583	1	-0.0815	0.417
5	Potom.005G13176-SNP169	Stem volume	a	-0.145	0.000542	2	0.0569	0.607	1	-0.0879	0.393
5	Potom.005G13176-SNP172	Stem volume	a	-0.118	0.00938	2	0.0457	0.613	1	-0.0724	0.387
5	Potom.005G13176-SNP175	Stem volume	a	-0.128	0.00282	2	0.0496	0.613	1	-0.0785	0.387
5	Potom.005G13176-SNP178	Stem volume	a	-0.142	0.0018	2	0.0567	0.601	1	-0.0855	0.399
5	Potom.005G13176-SNP179	Stem volume	a	-0.144	0.00111	2	0.0567	0.607	1	-0.0877	0.393
5	Potom.005G13176-SNP181	Stem volume	a	-0.144	0.00111	2	0.0567	0.607	1	-0.0877	0.393
5	Potom.005G13176-SNP183	Stem volume	a	-0.142	0.00271	2	0.055	0.613	1	-0.0871	0.387
5	Potom.005G13176-SNP185	Stem volume	a	-0.137	0.00287	2	0.0523	0.619	1	-0.0851	0.381
5	Potom.005G13176-SNP194	Stem volume	a	-0.145	0.000785	2	0.058	0.601	1	-0.0874	0.399
5	Potom.005G13176-	Stem volume	a	-0.29	0.00627	2	0.243	0.162	1	-0.0472	0.838

	SNP2										
5	Potom.005G13176-SNP20	Stem volume	a	-0.323	0.00139	2	0.265	0.179	1	-0.0579	0.821
5	Potom.005G13176-SNP21	Stem volume	a	-0.356	0.000647	2	0.294	0.175	1	-0.0624	0.825
5	Potom.005G13176-SNP22	Stem volume	a	-0.356	0.000647	2	0.294	0.175	1	-0.0624	0.825
5	Potom.005G13176-SNP233	Stem volume	a	-0.125	0.00328	2	0.0469	0.625	1	-0.0782	0.375
5	Potom.005G13176-SNP240	Stem volume	a	-0.11	0.00988	2	0.0411	0.625	1	-0.0686	0.375
5	Potom.005G13176-SNP246	Stem volume	a	0.119	0.00518	1	0.0447	0.625	2	-0.0746	0.375
5	Potom.005G13176-SNP252	Stem volume	a	0.139	0.000813	1	0.0528	0.619	2	-0.0857	0.381
5	Potom.005G13176-SNP263	Stem volume	a	-0.141	0.000633	2	0.0538	0.619	1	-0.0874	0.381
5	Potom.005G13176-SNP275	Stem volume	a	-0.132	0.00115	2	0.051	0.613	1	-0.0809	0.387
5	Potom.005G13176-SNP277	Stem volume	a	-0.141	0.00104	2	0.0544	0.613	1	-0.0863	0.387
5	Potom.005G13176-SNP279	Stem volume	a	-0.141	0.00104	2	0.0544	0.613	1	-0.0863	0.387
5	Potom.005G13176-SNP3	Stem volume	a	-0.224	0.00342	2	0.175	0.216	1	-0.0483	0.784
5	Potom.005G13176-	Stem volume	a	-0.153	0.00314	2	0.0644	0.579	1	-0.0886	0.421

	SNP30										
5	Potom.005G13176-SNP302	Stem volume	a	-0.123	0.0055	2	0.0467	0.619	1	-0.0759	0.381
5	Potom.005G13176-SNP306	Stem volume	a	-0.26	0.00347	2	0.228	0.125	1	-0.0325	0.875
5	Potom.005G13176-SNP4	Stem volume	a	-0.192	0.00923	2	0.151	0.211	1	-0.0404	0.789
5	Potom.005G13176-SNP78	Stem volume	a	-0.129	0.00484	2	0.0477	0.631	1	-0.0815	0.369
5	Potom.005G13176-SNP79	Stem volume	a	-0.112	0.00847	2	0.044	0.607	1	-0.068	0.393
5	Potom.005G13176-SNP83	Stem volume	a	-0.253	0.00488	2	0.219	0.137	1	-0.0347	0.863
5	Potom.005G13966-SNP100	Stem volume	a	-0.277	0.0018	2	0.18	0.348	1	-0.0961	0.652
5	Potom.005G13966-SNP36	Stem volume	a	-0.161	0.0011	2	0.0581	0.639	1	-0.103	0.361
5	Potom.005G13966-SNP74	Stem volume	a	-0.275	0.00116	2	0.175	0.364	1	-0.1	0.636
5	Potom.005G13966-SNP89	Stem volume	a	-0.323	0.000398	2	0.223	0.309	1	-0.0998	0.691
5	Potom.005G13966-SNP90	Stem volume	a	-0.281	0.00537	2	0.214	0.24	1	-0.0676	0.76
5	Potom.005G13966-SNP92	Stem volume	a	-0.37	7.82E-05	2	0.261	0.296	1	-0.11	0.704
5	Potom.005G13966-	Stem volume	a	-0.338	0.000113	2	0.226	0.331	1	-0.112	0.669

	SNP93													
5	Potom.005G13966-SNP97	Stem volume	a	-0.344	0.000104	2	0.283	0.179	1	-0.0614	0.821			
5	Pto-Wuschela-SNP11	Stem volume	a	0.0677	0.01	1	0.036	0.468	2	-0.0316	0.532			
5	Potom.005G13398-SNP5	Fiber width	d	6.5	0.00173	12	6.49	0.0118	22	0.0977	0.176	11	-0.115	0.812
5	Potom.005G13966-SNP5	Fiber width	a	0.354	0.000878	1	0.171	0.518	2	-0.183	0.482			
5	Pto-Wuschela-SNP3	Fiber width	d	-4.3	1.39E-07	22	5.97	0.025	11	1.91	0.0875	12	-0.356	0.887
5	Potom.005G13176-SNP129	α-cellulose content	d	14.3	0.0014	12	1.15	0.729	11	-2.13	0.259	22	-24.1	0.0118
5	Potom.005G13966-SNP52	α-cellulose content	a	-1.28	0.00418	2	0.661	0.482	1	-0.616	0.518			
5	Potom.005G13966-SNP61	α-cellulose content	d	12.2	0.00849	12	2	0.263	11	-0.377	0.725	22	-20.1	0.0125
4	Potom.004G12312-SNP30	Holocellulose content	d	6.55	0.00579	12	1.87	0.714	22	-2.02	0.143	11	-7.34	0.143
4	Potom.004G12312-SNP30	Holocellulose content	a	-3.55	0.00381	2	1.77	0.5	1	-1.77	0.5			
4	Potom.004G12312-SNP30	Fiber width	d	-4.75	0.000652	22	3.96	0.143	11	2.82	0.143	12	-1.36	0.714
2	Potom.002G05522-SNP46	Microfiber angle	d	-6.51	0.00191	22	11.8	0.0135	11	0.24	0.459	12	-0.51	0.527
2	Potom.002G05632-	Microfiber	a	-1.66	0.00819	2	1.28	0.228	1	-0.379	0.772			

	SNP49	angle												
2	Potom.002G05632-SNP51	Microfiber angle	a	-1.82	0.00647	2	1.42	0.218	1	-0.397	0.782			
2	Potom.002G05632-SNP53	Microfiber angle	a	-2.09	0.00251	2	1.69	0.193	1	-0.403	0.807			
2	Potom.002G07337-SNP2	Microfiber angle	a	-0.655	0.0099	2	0.351	0.464	1	-0.304	0.536			
2	Potom.002G05522-SNP1	Diameter at breast height	a	-4.45	0.00238	2	3.58	0.196	1	-0.874	0.804			
2	Potom.002G05522-SNP23	Diameter at breast height	a	-3.24	0.00162	2	2.29	0.292	1	-0.945	0.708			
2	Potom.002G05522-SNP24	Diameter at breast height	a	-3.83	0.000913	2	3.25	0.152	1	-0.584	0.848			
2	Potom.002G05522-SNP3	Diameter at breast height	d	6.18	0.00311	12	3.44	0.123	11	-0.0252	0.802	22	-5.46	0.0741
2	Potom.002G05522-SNP64	Diameter at breast height	a	-6.27	0.00253	2	5.94	0.0536	1	-0.336	0.946			
2	Potom.002G05522-SNP8	Diameter at breast height	a	3.94	0.00927	1	0.375	0.905	2	-3.57	0.0952			
2	Potom.002G05522-SNP9	Diameter at breast height	a	-4.84	0.00123	2	3.83	0.208	1	-1.01	0.792			
2	Potom.002G05769-SNP13	Diameter at breast height	a	-5.78	0.00118	2	5.09	0.119	1	-0.688	0.881			
2	Potom.002G05769-SNP16	Diameter at breast height	a	-7.54	0.00105	2	6.92	0.0823	1	-0.62	0.918			
2	Potom.002G05769-	Diameter at	a	-5.46	0.00311	2	5.1	0.065	1	-0.358	0.935			

	SNP3	breast height						5						
2	Potom.002G05769-SNP34	Diameter at breast height	a	-6.21	0.00199	2	5.56	0.105	1	-0.652	0.895			
2	Potom.002G05769-SNP38	Diameter at breast height	a	-6.19	0.00226	2	5.59	0.0964	1	-0.596	0.904			
2	Potom.002G05769-SNP49	Diameter at breast height	a	-6.09	0.00226	2	5.47	0.101	1	-0.616	0.899			
2	Potom.002G05769-SNP50	Diameter at breast height	a	-6.09	0.00226	2	5.47	0.101	1	-0.616	0.899			
2	Potom.002G05769-SNP54	Diameter at breast height	a	-6.09	0.00226	2	5.47	0.101	1	-0.616	0.899			
2	Potom.002G05769-SNP61	Diameter at breast height	a	-7.01	0.000708	2	6.35	0.0949	1	-0.666	0.905			
2	Potom.002G05769-SNP66	Diameter at breast height	a	-6.09	0.00226	2	5.47	0.101	1	-0.616	0.899			
2	Potom.002G05769-SNP71	Diameter at breast height	a	-5.11	0.00883	2	4.55	0.108	1	-0.554	0.892			
2	Potom.002G07206-SNP69	Diameter at breast height	d	6.48	0.00841	12	0.399	0.917	11	-2.12	0.0595	22	-10.1	0.0238
2	Potom.002G07337-SNP12	Diameter at breast height	d	8.46	0.00414	12	0.746	0.833	11	-3.07	0.155	22	-12.4	0.0119
2	Potom.002G07337-SNP129	Diameter at breast height	a	-4.43	0.00902	2	3.64	0.178	1	-0.787	0.822			
2	Potom.002G07337-SNP135	Diameter at breast height	a	0.736	0.00611	1	0.359	0.512	2	-0.377	0.488			
2	Potom.002G07337-	Diameter at	a	-2.22	0.00974	2	1.14	0.487	1	-1.08	0.513			

	SNP15	breast height												
2	Potom.002G07337-SNP16	Diameter at breast height	a	-2.39	0.00312	2	0.937	0.608	1	-1.46	0.392			
2	Potom.002G07337-SNP21	Diameter at breast height	d	9.47	0.0083	12	3.05	0.105	11	-0.175	0.882	22	-12.7	0.0132
2	Potom.002G07337-SNP22	Diameter at breast height	a	1.41	0.0082	1	0.579	0.59	2	-0.834	0.41			
2	Potom.002G07337-SNP229	Diameter at breast height	d	-4.7	0.00567	11	5.59	0.0595	22	2.33	0.119	12	-0.742	0.821
2	Potom.002G07337-SNP31	Diameter at breast height	d	10.7	0.00255	12	4.09	0.0732	11	-0.154	0.915	22	-13	0.0122
2	Potom.002G07337-SNP41	Diameter at breast height	d	6.93	0.00637	12	0.471	0.929	11	-5.47	0.0476	22	-7.45	0.0238
2	Potom.002G07337-SNP44	Diameter at breast height	d	10.1	0.00264	12	2.71	0.119	11	-0.172	0.869	22	-14.5	0.0119
2	Potom.002G07337-SNP51	Diameter at breast height	d	9.21	0.00728	12	2.73	0.107	11	-0.159	0.881	22	-12.8	0.0119
2	Potom.002G07337-SNP56	Diameter at breast height	a	-4.64	0.00668	2	3.88	0.162	1	-0.752	0.838			
2	Potom.002G07963-SNP137	Diameter at breast height	a	-1.81	0.00151	2	0.734	0.595	1	-1.08	0.405			
2	Potom.002G07963-SNP138	Diameter at breast height	a	-1.81	0.00151	2	0.734	0.595	1	-1.08	0.405			
2	Potom.002G07963-SNP18	Diameter at breast height	d	10.2	0.000972	12	2.02	0.198	11	-0.252	0.79	22	-16.2	0.0123
2	Potom.002G07963-	Diameter at	d	8.55	0.00684	12	1.51	0.232	11	-0.24	0.756	2	-13.8	0.012

	SNP19	breast height										2		2
2	Potom.002G07963-SNP20	Diameter at breast height	d	8.56	0.00614	12	1.51	0.226	11	-0.231	0.762	22	-13.9	0.0119
2	Potom.002G07963-SNP207	Diameter at breast height	a	-1.43	0.00522	2	0.615	0.571	1	-0.819	0.429			
2	Potom.002G07963-SNP21	Diameter at breast height	d	8.25	0.00901	12	1.45	0.229	11	-0.225	0.759	22	-13.4	0.012
2	Potom.002G07963-SNP22	Diameter at breast height	d	8.91	0.00446	12	1.63	0.217	11	-0.235	0.771	22	-14.3	0.012
2	Potom.002G07963-SNP220	Diameter at breast height	a	-5.02	0.00215	2	4.6	0.0843	1	-0.424	0.916			
2	Potom.002G07963-SNP23	Diameter at breast height	d	8.79	0.00602	12	1.81	0.179	11	-0.197	0.81	22	-13.8	0.0119
2	Potom.002G07963-SNP230	Diameter at breast height	a	-4.24	0.00994	2	3.61	0.149	1	-0.631	0.851			
2	Potom.002G07963-SNP25	Diameter at breast height	d	10.2	0.00104	12	2	0.19	11	-0.237	0.798	22	-16.1	0.0119
2	Potom.002G07963-SNP26	Diameter at breast height	a	-3.25	0.0024	2	2.75	0.155	1	-0.504	0.845			
2	Potom.002G07963-SNP26	Diameter at breast height	d	10.3	0.000404	12	1.67	0.286	11	-0.396	0.702	22	-16.8	0.0119
2	Potom.002G07963-SNP261	Diameter at breast height	a	-6.01	0.00868	2	5.5	0.0855	1	-0.514	0.914			
2	Potom.002G07963-SNP39	Diameter at breast height	d	7.84	0.00809	12	0.905	0.536	11	-0.726	0.452	22	-13.1	0.0119
2	Potom.002G07963-	Diameter at	d	8.26	0.00522	12	1.07	0.44	11	-0.557	0.548	2	-13.8	0.011

	SNP45	breast height										2		9
2	Potom.002G07963-SNP75	Diameter at breast height	d	-9.24	0.00136	22	16.3	0.0154	11	0.833	0.262	12	-0.649	0.723
2	Potom.002G07337-SNP2	Fiber length	a	0.0148	0.00588	1	0.00678	0.541	2	-0.00799	0.459			
2	Potom.002G05522-SNP23	Tree height	a	-1.48	0.00208	2	1.05	0.292	1	-0.432	0.708			
2	Potom.002G05522-SNP46	Tree height	a	1.23	0.00182	1	0.341	0.723	2	-0.891	0.277			
2	Potom.002G07337-SNP15	Tree height	a	-1.01	0.00986	2	0.517	0.487	1	-0.492	0.513			
2	Potom.002G07337-SNP56	Tree height	a	-2.07	0.00873	2	1.73	0.162	1	-0.335	0.838			
2	Potom.002G05522-SNP72	Hemicellulose content	a	-2.14	0.0046	2	1.25	0.417	1	-0.89	0.583			
2	Potom.002G05522-SNP73	Hemicellulose content	d	7.63	0.000905	12	1.58	0.795	22	-5.75	0.0723	11	-6.35	0.133
2	Potom.002G05522-SNP74	Hemicellulose content	a	-2.15	0.00266	2	1.23	0.428	1	-0.919	0.572			
2	Potom.002G05769-SNP21	Hemicellulose content	a	-5.21	0.00794	2	4.6	0.118	1	-0.613	0.882			
2	Potom.002G05769-SNP22	Hemicellulose content	a	-5.21	0.00794	2	4.6	0.118	1	-0.613	0.882			
2	Potom.002G05769-SNP70	Hemicellulose content	d	5.93	0.009	12	3.22	0.286	11	-0.676	0.607	22	-4.75	0.107
2	Potom.002G07337-	Hemicellulose	a	1.29	0.0063	1	0.616	0.524	2	-0.677	0.476			

	SNP42	content												
2	Potom.002G07337-SNP43	Hemicellulose content	a	1.29	0.0063	1	0.616	0.524	2	-0.677	0.476			
2	Potom.002G07337-SNP50	Hemicellulose content	a	3.58	0.00274	1	0.96	0.732	2	-2.62	0.268			
2	Potom.002G07963-SNP216	Hemicellulose content	a	0.97	0.00957	1	0.473	0.512	2	-0.497	0.488			
2	Potom.002G07963-SNP224	Hemicellulose content	d	8.94	0.00794	12	0.695	0.859	11	-2.22	0.118	22	-14.3	0.0235
2	Potom.002G05522-SNP3	Holocellulose content	d	-10.7	0.00202	22	7.09	0.0732	11	0.521	0.793	12	-6.95	0.134
2	Potom.002G07337-SNP106	Holocellulose content	a	-2.24	0.00996	2	1.28	0.429	1	-0.96	0.571			
2	Potom.002G07337-SNP21	Holocellulose content	a	9.15	0.00388	1	0.654	0.929	2	-8.5	0.0714			
2	Potom.002G07337-SNP312	Holocellulose content	a	2.07	0.00553	1	1.16	0.441	2	-0.914	0.559			
2	Potom.002G07337-SNP51	Holocellulose content	a	7.9	0.00816	1	0.558	0.929	2	-7.34	0.0706			
2	Potom.002G07963-SNP18	Holocellulose content	a	6.22	0.00661	1	0.72	0.884	2	-5.5	0.116			
2	Potom.002G07963-SNP7	Holocellulose content	d	-13.4	0.00817	22	23.3	0.0118	11	2.14	0.153	12	-0.72	0.835
2	Potom.002G05632-SNP86	Lignin content	d	2.91	0.00453	12	0.194	0.929	11	-2.23	0.0471	22	-3.2	0.0235
2	Potom.002G07337-	Lignin content	d	3.75	0.0071	12	1.06	0.117	11	-0.0628	0.87	2	-5.31	0.013

	SNP21											2		
2	Potom.002G07337-SNP58	Lignin content	d	3.56	0.00232	12	0.8	0.186	11	-0.0889	0.8	22	-5.42	0.0143
2	Potom.002G05522-SNP1	Stem volume	a	-0.272	0.00905	2	0.218	0.196	1	-0.0534	0.804			
2	Potom.002G05522-SNP23	Stem volume	a	-0.262	0.000317	2	0.185	0.292	1	-0.0764	0.708			
2	Potom.002G05522-SNP24	Stem volume	a	-0.234	0.00454	2	0.198	0.152	1	-0.0356	0.848			
2	Potom.002G05522-SNP54	Stem volume	a	-0.26	0.00152	2	0.206	0.21	1	-0.0546	0.79			
2	Potom.002G05522-SNP64	Stem volume	a	-0.463	0.0014	2	0.438	0.0536	1	-0.0248	0.946			
2	Potom.002G05522-SNP9	Stem volume	a	-0.297	0.00526	2	0.235	0.208	1	-0.0618	0.792			
2	Potom.002G05769-SNP13	Stem volume	a	-0.418	0.000844	2	0.369	0.119	1	-0.0498	0.881			
2	Potom.002G05769-SNP16	Stem volume	a	-0.562	0.000521	2	0.516	0.0823	1	-0.0462	0.918			
2	Potom.002G05769-SNP3	Stem volume	d	-0.715	0.0011	22	0.881	0.0119	11	0.0203	0.881	12	-0.264	0.107
2	Potom.002G05769-SNP3	Stem volume	a	-0.484	7.34E-05	2	0.452	0.0655	1	-0.0317	0.935			
2	Potom.002G05769-SNP34	Stem volume	a	-0.47	0.000755	2	0.42	0.105	1	-0.0493	0.895			
2	Potom.002G05769-	Stem volume	a	-0.398	0.00601	2	0.359	0.097	1	-0.0388	0.902			

	SNP36							6						
2	Potom.002G05769-SNP38	Stem volume	a	-0.468	0.000996	2	0.423	0.0964	1	-0.0451	0.904			
2	Potom.002G05769-SNP49	Stem volume	a	-0.462	0.00102	2	0.415	0.101	1	-0.0467	0.899			
2	Potom.002G05769-SNP50	Stem volume	a	-0.462	0.00102	2	0.415	0.101	1	-0.0467	0.899			
2	Potom.002G05769-SNP54	Stem volume	a	-0.462	0.00102	2	0.415	0.101	1	-0.0467	0.899			
2	Potom.002G05769-SNP61	Stem volume	a	-0.537	0.000202	2	0.486	0.0949	1	-0.051	0.905			
2	Potom.002G05769-SNP66	Stem volume	a	-0.462	0.00102	2	0.415	0.101	1	-0.0467	0.899			
2	Potom.002G05769-SNP71	Stem volume	a	-0.409	0.00263	2	0.365	0.108	1	-0.0444	0.892			
2	Potom.002G07337-SNP129	Stem volume	a	-0.336	0.00477	2	0.277	0.178	1	-0.0598	0.822			
2	Potom.002G07337-SNP135	Stem volume	a	0.0551	0.00346	1	0.0269	0.512	2	-0.0282	0.488			
2	Potom.002G07337-SNP16	Stem volume	a	-0.165	0.00368	2	0.0647	0.608	1	-0.101	0.392			
2	Potom.002G07337-SNP229	Stem volume	d	-0.389	0.001	11	0.472	0.0595	22	0.183	0.119	12	-0.0608	0.821
2	Potom.002G07337-SNP56	Stem volume	a	-0.371	0.00205	2	0.311	0.162	1	-0.0603	0.838			
2	Potom.002G07963-	Stem volume	a	-0.158	0.0092	2	0.114	0.278	1	-0.044	0.722			

	SNP12													
2	Potom.002G07963-SNP137	Stem volume	a	-0.118	0.00352	2	0.0478	0.595	1	-0.0704	0.405			
2	Potom.002G07963-SNP138	Stem volume	a	-0.118	0.00352	2	0.0478	0.595	1	-0.0704	0.405			
2	Potom.002G07963-SNP18	Stem volume	d	0.578	0.00875	12	0.114	0.198	11	-0.0143	0.79	22	-0.914	0.0123
2	Potom.002G07963-SNP25	Stem volume	d	0.59	0.00671	12	0.116	0.19	11	-0.0137	0.798	22	-0.934	0.0119
2	Potom.002G07963-SNP26	Stem volume	d	0.576	0.00506	12	0.098	0.286	11	-0.0241	0.702	22	-0.931	0.0119
2	Potom.002G07963-SNP26	Stem volume	a	-0.242	0.00169	2	0.205	0.155	1	-0.0375	0.845			
2	Potom.002G07963-SNP75	Stem volume	d	-0.956	1.03E-06	22	1.74	0.0154	11	0.056	0.262	12	-0.0573	0.723
2	Potom.002G05522-SNP33	Fiber width	a	-1.68	0.00103	2	1.5	0.106	1	-0.178	0.894			
2	Potom.002G05769-SNP15	Fiber width	d	2.55	0.00823	12	1.15	0.113	11	-0.0703	0.863	22	-2.73	0.025
2	Potom.002G05769-SNP58	Fiber width	d	3.34	0.00532	12	0.835	0.118	11	-0.0457	0.871	22	-4.96	0.0118
2	Potom.002G07206-SNP14	Fiber width	d	-2.28	0.00859	22	1.95	0.039	11	0.145	0.805	12	-1.24	0.156
2	Potom.002G07963-SNP152	Fiber width	d	2.13	0.00654	12	0.794	0.19	11	-0.0756	0.774	22	-2.6	0.0357
2	Potom.002G07963-	Fiber width	a	0.369	0.000624	1	0.178	0.518	2	-0.191	0.482			

	SNP210													
2	Potom.002G07963-SNP255	Fiber width	d	3.02	0.00619	12	0.565	0.214	11	-0.0822	0.774	22	-4.82	0.0119
2	Potom.002G07963-SNP255	Fiber width	a	-1.36	0.00367	2	1.2	0.119	1	-0.162	0.881			
2	Potom.002G05522-SNP23	α-cellulose content	a	4.27	0.0067	1	1.23	0.712	2	-3.04	0.288			
2	Potom.002G05522-SNP24	α-cellulose content	a	6.25	0.000541	1	0.903	0.855	2	-5.34	0.145			
2	Potom.002G05522-SNP33	α-cellulose content	d	14.1	0.00318	12	2.79	0.188	11	-0.331	0.8	22	-22.2	0.0118
2	Potom.002G07337-SNP103	α-cellulose content	d	12.8	0.00665	11	1.71	0.0588	12	0.231	0.929	22	-26.8	0.0118
2	Potom.002G07963-SNP156	α-cellulose content	d	7.71	0.00847	12	1.73	0.447	11	-0.732	0.518	22	-11.2	0.0353
2	Potom.002G07963-SNP197	α-cellulose content	a	-1.49	0.00451	2	0.789	0.471	1	-0.701	0.529			
2	Potom.002G07963-SNP73	α-cellulose content	a	-3.14	0.00127	2	2.03	0.353	1	-1.11	0.647			
2	Potom.002G07963-SNP75	α-cellulose content	d	12.1	0.00821	12	1.07	0.712	11	-1.67	0.273	22	-20.4	0.0152
1	Potom.001G00363-SNP5	Microfiber angle	d	7.91	0.00455	12	7.39	0.0241	11	0.0155	0.795	22	-1.05	0.181
1	Potom.001G01788-SNP16	Microfiber angle	d	-4.49	0.00737	22	5.48	0.0238	11	2.85	0.0595	12	-0.327	0.917
1	Potom.001G01788-	Microfiber	d	-6.88	0.000615	11	12.2	0.011	22	0.628	0.274	1	-0.445	0.714

	SNP34	angle						9				2		
1	Potom.001G01788-SNP42	Microfiber angle	d	-6.03	0.00584	22	7.41	0.0119	11	4.04	0.0476	12	-0.299	0.94
1	Potom.001G03193-SNP38	Microfiber angle	a	-5.06	0.00295	2	4.76	0.0592	1	-0.3	0.941			
1	Potom.001G03353-SNP104	Microfiber angle	d	-7.32	0.00219	22	7.32	0.012	11	6.8	0.0241	12	-0.261	0.964
1	Potom.001G03606-SNP34	Microfiber angle	a	-4.47	0.0017	2	4.23	0.0536	1	-0.24	0.946			
1	Potom.001G03606-SNP87	Microfiber angle	a	-3.86	0.000792	2	3.56	0.0783	1	-0.302	0.922			
1	Potom.001G00161-SNP13	Diameter at breast height	a	-4.87	0.000976	2	3.84	0.212	1	-1.03	0.788			
1	Potom.001G00161-SNP19	Diameter at breast height	d	11.6	0.000959	12	3.9	0.0843	11	-0.16	0.904	22	-15.3	0.012
1	Potom.001G00161-SNP20	Diameter at breast height	d	11.6	0.000959	12	3.9	0.0843	11	-0.16	0.904	22	-15.3	0.012
1	Potom.001G00161-SNP3	Diameter at breast height	a	-4.82	0.0034	2	4.09	0.151	1	-0.726	0.849			
1	Potom.001G01331-SNP70	Diameter at breast height	a	4.86	0.0057	1	0.356	0.927	2	-4.5	0.0732			
1	Potom.001G01331-SNP73	Diameter at breast height	a	3.67	0.00891	1	0.43	0.883	2	-3.24	0.117			
1	Potom.001G01331-SNP83	Diameter at breast height	a	3.3	0.00739	1	0.452	0.863	2	-2.85	0.137			
1	Potom.001G01788-	Diameter at	a	-2.04	0.00082	2	0.752	0.631	1	-1.29	0.369			

	SNP34	breast height												
1	Potom.001G01788-SNP53	Diameter at breast height	a	-2.17	0.00156	2	0.722	0.667	1	-1.44	0.333			
1	Potom.001G03193-SNP27	Diameter at breast height	a	-3.75	0.00282	2	2.58	0.312	1	-1.17	0.688			
1	Potom.001G03193-SNP28	Diameter at breast height	d	8.61	0.00835	12	1.98	0.181	11	-0.218	0.806	22	-13	0.0139
1	Potom.001G03193-SNP29	Diameter at breast height	d	8.45	0.00854	12	1.84	0.192	11	-0.22	0.795	22	-13	0.0137
1	Potom.001G03193-SNP30	Diameter at breast height	d	8.5	0.000269	12	4.23	0.145	11	-0.355	0.816	22	-8.19	0.0395
1	Potom.001G03193-SNP31	Diameter at breast height	d	8.64	0.000238	12	4.57	0.133	11	-0.36	0.827	22	-7.78	0.04
1	Potom.001G03193-SNP4	Diameter at breast height	a	-4.53	0.000945	2	4.04	0.108	1	-0.491	0.892			
1	Potom.001G03193-SNP44	Diameter at breast height	a	-4.56	0.00262	2	3.73	0.182	1	-0.829	0.818			
1	Potom.001G03193-SNP63	Diameter at breast height	a	3.06	0.00245	1	0.624	0.796	2	-2.44	0.204			
1	Potom.001G03353-SNP118	Diameter at breast height	a	-2.01	0.00212	2	0.717	0.643	1	-1.29	0.357			
1	Potom.001G03353-SNP154	Diameter at breast height	a	-1.98	0.00164	2	0.718	0.637	1	-1.26	0.363			
1	Potom.001G03353-SNP161	Diameter at breast height	a	-2.07	0.00193	2	0.797	0.614	1	-1.27	0.386			
1	Potom.001G03353-	Diameter at	a	-2.61	0.000555	2	1.13	0.568	1	-1.48	0.432			

	SNP164	breast height											
1	Potom.001G03353-SNP35	Diameter at breast height	a	-1.56	0.00391	2	0.632	0.595	1	-0.929	0.405		
1	Potom.001G03353-SNP57	Diameter at breast height	a	2.46	0.000175	1	0.849	0.655	2	-1.61	0.345		
1	Potom.001G03353-SNP86	Diameter at breast height	a	-2.26	0.000456	2	0.795	0.649	1	-1.47	0.351		
1	Potom.001G03606-SNP23	Diameter at breast height	d	9.86	0.00328	12	2.66	0.119	11	-0.17	0.869	22	-14.2	0.0119
1	Potom.001G03606-SNP24	Diameter at breast height	d	10.7	0.00265	12	3.67	0.0833	11	-0.155	0.905	22	-13.9	0.0119
1	Potom.001G03606-SNP25	Diameter at breast height	d	11.1	0.00164	12	3.78	0.0833	11	-0.157	0.905	22	-14.6	0.0119
1	Potom.001G01788-SNP43	Fiber length	a	0.0143	0.00835	1	0.00756	0.471	2	-0.00672	0.529		
1	Potom.001G01331-SNP39	Hemicellulose content	d	13	0.00402	12	2.07	0.259	11	-0.388	0.729	22	-21.4	0.0118
1	Potom.001G02062-SNP7	Hemicellulose content	a	-4.74	0.00866	2	4.1	0.135	1	-0.641	0.865		
1	Potom.001G03193-SNP1	Hemicellulose content	a	9.81	0.00782	1	0.532	0.946	2	-9.28	0.0542		
1	Potom.001G03353-SNP154	Hemicellulose content	d	14	0.00128	12	0.945	0.694	22	-1.24	0.294	11	-24.8	0.0118
1	Potom.001G03353-SNP165	Hemicellulose content	d	11.1	0.000616	12	1.05	0.78	11	-1.93	0.195	22	-18.2	0.0244
1	Potom.001G03606-	Hemicellulose	d	13.3	0.00927	12	5.63	0.082	11	-0.316	0.906	2	-15.1	0.011

	SNP34	content						4				2		8
1	Potom.001G03606-SNP35	Hemicellulose content	a	9.83	0.00114	1	0.585	0.94	2	-9.25	0.0595			
1	Potom.001G00161-SNP11	Holocellulose content	a	12.3	0.00133	1	0.988	0.92	2	-11.3	0.0802			
1	Potom.001G00363-SNP52	Holocellulose content	a	9.8	0.00798	1	0.94	0.904	2	-8.86	0.0959			
1	Potom.001G03193-SNP1	Holocellulose content	a	16.5	5.93E-05	1	0.894	0.946	2	-15.6	0.0542			
1	Potom.001G03193-SNP15	Holocellulose content	a	10.6	0.00997	1	0.669	0.937	2	-9.91	0.0633			
1	Potom.001G03193-SNP5	Holocellulose content	a	1.81	0.00511	1	0.98	0.459	2	-0.831	0.541			
1	Potom.001G03193-SNP50	Holocellulose content	a	2.08	0.00255	1	1.12	0.465	2	-0.969	0.535			
1	Potom.001G03193-SNP52	Holocellulose content	a	2.16	0.00301	1	1.14	0.471	2	-1.02	0.529			
1	Potom.001G03353-SNP165	Holocellulose content	d	10.2	0.00616	12	0.634	0.78	11	-0.172	0.195	22	-18.9	0.0244
1	Potom.001G03353-SNP24	Holocellulose content	a	9.38	0.00112	1	0.858	0.909	2	-8.52	0.0915			
1	Potom.001G03353-SNP25	Holocellulose content	a	4.54	0.00684	1	1.73	0.62	2	-2.81	0.38			
1	Potom.001G03606-SNP24	Holocellulose content	a	10.6	0.00455	1	0.5	0.953	2	-10.1	0.0471			
1	Potom.001G03606-	Holocellulose	d	18.4	0.00144	12	7.71	0.082	11	-0.428	0.906	2	-21	0.011

	SNP34	content						4				2		8
1	Potom.001G03606-SNP35	Holocellulose content	a	10.9	0.00236	1	0.647	0.94	2	-10.2	0.0595			
1	Potom.001G01788-SNP62	Lignin content	d	3.33	0.00192	12	0.185	0.941	22	-2.22	0.0353	11	-4.07	0.0235
1	Potom.001G03193-SNP17	Lignin content	a	1.1	0.00225	1	0.293	0.733	2	-0.805	0.267			
1	Potom.001G03193-SNP18	Lignin content	a	1.1	0.00225	1	0.293	0.733	2	-0.805	0.267			
1	Potom.001G03193-SNP19	Lignin content	a	1.29	0.000843	1	0.31	0.761	2	-0.985	0.239			
1	Potom.001G03193-SNP21	Lignin content	a	1.37	0.000736	1	0.3	0.781	2	-1.07	0.219			
1	Potom.001G03193-SNP22	Lignin content	a	1.27	0.002	1	0.273	0.785	2	-0.996	0.215			
1	Potom.001G03193-SNP23	Lignin content	a	1.35	0.000707	1	0.295	0.782	2	-1.06	0.218			
1	Potom.001G03193-SNP24	Lignin content	a	1.31	0.000996	1	0.283	0.785	2	-1.03	0.215			
1	Potom.001G03193-SNP25	Lignin content	a	1.25	0.00238	1	0.265	0.789	2	-0.988	0.211			
1	Potom.001G03353-SNP154	Lignin content	d	-3.99	0.000845	11	7.31	0.0118	22	0.229	0.294	12	-0.221	0.694
1	Potom.001G03353-SNP25	Lignin content	a	-1.15	0.00322	2	0.711	0.38	1	-0.436	0.62			
1	Potom.001G00161-	Stem volume	a	-0.27	0.00555	2	0.213	0.212	1	-0.0571	0.788			

	SNP13													
1	Potom.001G00161-SNP19	Stem volume	d	0.737	0.00269	12	0.231	0.0843	11	-0.00818	0.904	22	-1	0.012
1	Potom.001G00161-SNP20	Stem volume	d	0.737	0.00269	12	0.231	0.0843	11	-0.00818	0.904	22	-1	0.012
1	Potom.001G01788-SNP34	Stem volume	a	-0.118	0.00672	2	0.0436	0.631	1	-0.0745	0.369			
1	Potom.001G01788-SNP53	Stem volume	a	-0.151	0.00179	2	0.0502	0.667	1	-0.1	0.333			
1	Potom.001G03193-SNP27	Stem volume	a	-0.256	0.00441	2	0.176	0.312	1	-0.0799	0.688			
1	Potom.001G03193-SNP30	Stem volume	d	0.524	0.00135	12	0.247	0.145	11	-0.0179	0.816	22	-0.536	0.0395
1	Potom.001G03193-SNP31	Stem volume	d	0.541	0.00111	12	0.269	0.133	11	-0.0181	0.827	22	-0.525	0.04
1	Potom.001G03193-SNP33	Stem volume	d	-0.764	0.000302	22	1.02	0.0139	11	0.0234	0.847	12	-0.245	0.139
1	Potom.001G03193-SNP33	Stem volume	a	-0.413	0.000229	2	0.379	0.0833	1	-0.0344	0.917			
1	Potom.001G03193-SNP44	Stem volume	a	-0.336	0.00226	2	0.275	0.182	1	-0.061	0.818			
1	Potom.001G03353-SNP118	Stem volume	a	-0.15	0.0012	2	0.0534	0.643	1	-0.0962	0.357			
1	Potom.001G03353-SNP154	Stem volume	a	-0.148	0.000751	2	0.0538	0.637	1	-0.0943	0.363			
1	Potom.001G03353-	Stem volume	a	-0.149	0.00144	2	0.0575	0.614	1	-0.0917	0.386			

	SNP161													
1	Potom.001G03353-SNP164	Stem volume	a	-0.173	0.000388	2	0.0749	0.568	1	-0.0984	0.432			
1	Potom.001G03353-SNP57	Stem volume	a	0.175	0.000145	1	0.0603	0.655	2	-0.114	0.345			
1	Potom.001G03353-SNP86	Stem volume	a	-0.161	0.000366	2	0.0567	0.649	1	-0.105	0.351			
1	Potom.001G00161-SNP14	Fiber width	d	6.11	0.00262	12	5.8	0.0118	11	0.217	0.718	22	-0.827	0.271
1	Potom.001G02062-SNP8	Fiber width	a	-1.33	0.00863	2	1.19	0.102	1	-0.136	0.898			
1	Potom.001G03193-SNP32	Fiber width	a	1.58	0.01	1	0.154	0.903	2	-1.43	0.0972			
1	Potom.001G03606-SNP42	Fiber width	a	-1.54	0.00759	2	1.42	0.0833	1	-0.129	0.917			
1	Potom.001G03606-SNP49	Fiber width	a	-1.22	0.00746	2	1.06	0.129	1	-0.157	0.871			
1	Potom.001G03606-SNP23	α-cellulose content	d	-13.5	0.00525	22	20.5	0.0118	11	0.149	0.871	12	-3.16	0.118
1	Potom.001G03606-SNP23	α-cellulose content	a	8.22	0.00142	1	0.58	0.929	2	-7.64	0.0706			
1	Potom.001G03606-SNP29	α-cellulose content	d	-13.6	0.00765	22	2.25	0.2	11	-0.0127	0.765	12	-12.5	0.0353

Table S5 Significant pairwise results of SNP-SNP interactions.

Chr1	Locus1	Chr2	Locus2	Trait	Test	Effect	P_value
2	Potom.002G05522-SNP54	5	Potom.005G13176-SNP115	Lignin content	DD	5.06E+00	1.00E-02
2	Potom.002G05522-SNP60	2	Potom.002G07206-SNP38	Diameter at breast height	DD	-1.06E+01	1.00E-02
2	Potom.002G05769-SNP13	5	Potom.005G13966-SNP90	Hemicellulose content	AD	-1.84E+01	1.00E-02
5	Potom.005G13176-SNP15	5	Potom.005G13966-SNP97	Stem volume	AD	4.79E-01	1.00E-02
5	Potom.005G13176-SNP115	16	Potom.016G33891-SNP13	Diameter at breast height	AD	6.46E+00	1.00E-02
8	Potom.008G22516-SNP12	10	Potom.010G25706-SNP45	Fiber width	DD	7.10E+00	1.00E-02
8	Potom.008G22699-SNP100	13	Potom.013G29538-SNP15	Stem volume	DD	7.07E-01	1.00E-02
16	Potom.016G33891-SNP17	20	Potom.003G08321-SNP26	Tree height	AD	-3.95E+00	1.00E-02
2	Potom.002G05522-SNP12	5	Pto-Wuschela-SNP5	Fiber width	DA	-2.04E+00	9.99E-03
2	Potom.002G05769-SNP59	8	Potom.008G22516-SNP48	Stem volume	AA	-6.14E-01	9.99E-03
1	Potom.001G00363-SNP19	20	Potom.003G08321-SNP3	Fiber width	DA	-3.05E+00	9.97E-03
2	Potom.002G05769-SNP72	16	Potom.016G33876-SNP73	Fiber width	DA	-1.85E+00	9.97E-03
5	Potom.005G13176-SNP39	8	Potom.008G22699-SNP74	Diameter at breast height	AA	3.01E+00	9.97E-03
5	Potom.005G13176-SNP44	16	Potom.016G33876-SNP56	Stem volume	DA	1.53E-01	9.97E-03
10	Potom.010G24967-SNP40	13	Potom.013G29538-SNP72	Fiber length	DA	-1.03E-01	9.95E-03
1	Potom.001G00363-SNP23	5	Potom.005G13985-SNP17	Hemicellulose content	AA	1.32E+01	9.94E-03
8	Potom.008G22699-SNP74	20	Potom.003G08321-SNP33	Stem volume	AA	2.53E-01	9.94E-03
2	Potom.002G07337-SNP15	2	Potom.002G07963-SNP152	Stem volume	DA	-6.31E-01	9.93E-03
5	Potom.005G13176-SNP47	5	Potom.005G13333-SNP12	Fiber width	DA	-2.03E+00	9.93E-03
8	Potom.008G21952-SNP16	10	Potom.010G25398-SNP26	Stem volume	AD	-6.21E-01	9.93E-03
2	Potom.002G07963-SNP72	5	Potom.005G13176-SNP47	Fiber length	AA	-3.07E-02	9.92E-03
5	Potom.005G13333-SNP13	16	Potom.016G33891-SNP6	α-cellulose content	AD	1.42E+01	9.92E-03
10	Potom.010G25398-SNP26	20	Potom.003G08321-SNP17	Tree height	AD	3.03E+00	9.92E-03
1	Potom.001G03353-SNP164	2	Potom.002G05522-SNP4	Holocellulose content	DA	-6.53E+00	9.91E-03

5	Potom.005G13966-SNP97	13	Potom.013G29538-SNP7	Hemicellulose content	AA	1.01E+01	9.91E-03
1	Potom.001G03606-SNP27	16	Potom.016G34352-SNP98	Holocellulose content	AA	9.39E+00	9.90E-03
2	Potom.002G05522-SNP1	20	Potom.003G09473-SNP11	α-cellulose content	AD	-1.42E+01	9.90E-03
5	Potom.005G13985-SNP20	16	Potom.016G33876-SNP25	Holocellulose content	DA	2.03E+01	9.90E-03
1	Potom.001G00363-SNP23	8	Potom.008G22699-SNP71	Holocellulose content	AD	-1.61E+01	9.89E-03
1	Potom.001G03606-SNP26	2	Potom.002G07963-SNP231	Fiber length	AA	1.10E-01	9.89E-03
1	Potom.001G03193-SNP27	12	Potom.012G28409-SNP23	Stem volume	DA	-3.25E-01	9.88E-03
2	Potom.002G05522-SNP60	8	Potom.008G22699-SNP69	Tree height	AA	2.15E+00	9.88E-03
10	Potom.010G24967-SNP45	10	Potom.010G25398-SNP26	Lignin content	DA	-2.70E+00	9.88E-03
2	Potom.002G05522-SNP60	5	Potom.005G13333-SNP12	α-cellulose content	AA	5.91E+00	9.87E-03
5	Potom.005G13966-SNP77	16	Potom.016G33891-SNP6	Tree height	AD	-2.13E+00	9.87E-03
1	Potom.001G03193-SNP20	16	Potom.016G33876-SNP74	Fiber width	DD	-3.89E+00	9.86E-03
2	Potom.002G07337-SNP229	5	Potom.005G13966-SNP97	Fiber length	AD	8.16E-02	9.85E-03
14	Potom.014G31981-SNP26	16	Potom.016G34352-SNP109	Holocellulose content	AD	1.24E+01	9.85E-03
1	Potom.001G03606-SNP26	10	Potom.010G25398-SNP26	Tree height	DD	5.84E+00	9.84E-03
5	Potom.005G13333-SNP13	13	Potom.013G29538-SNP4	Stem volume	DD	6.07E-01	9.83E-03
2	Potom.002G05522-SNP60	20	Potom.003G08321-SNP14	α-cellulose content	DD	2.95E+01	9.82E-03
20	Potom.003G08321-SNP20	20	Potom.003G09473-SNP11	Stem volume	DD	-7.65E-01	9.81E-03
16	Potom.016G33876-SNP73	18	Potom.018G36220-SNP42	Microfiber angle	AA	2.19E+00	9.80E-03
13	Potom.013G29538-SNP71	14	Potom.014G31981-SNP25	Fiber width	AD	-2.35E+00	9.79E-03
10	Potom.010G24967-SNP43	14	Potom.014G31981-SNP25	Diameter at breast height	DA	-7.69E+00	9.77E-03
13	Potom.013G29538-SNP72	20	Potom.003G09473-SNP2	Fiber length	AA	-6.30E-02	9.77E-03
2	Potom.002G07337-SNP16	5	Potom.005G13333-SNP13	Fiber length	DD	1.25E-01	9.76E-03
8	Potom.008G22699-SNP38	8	Potom.008G22699-SNP98	Stem volume	AD	8.94E-01	9.76E-03
8	Potom.008G22699-SNP74	13	Potom.013G29538-SNP117	Fiber width	AD	-1.47E+00	9.76E-03
5	Potom.005G13176-SNP41	16	Potom.016G33876-SNP71	Diameter at breast height	AD	-7.26E+00	9.75E-03

12	Potom.012G28819-SNP45	14	Potom.014G31981-SNP26	Fiber width	DD	4.42E+00	9.75E-03
10	Potom.010G25706-SNP89	20	Potom.003G08321-SNP1	Stem volume	DA	6.17E-01	9.74E-03
1	Potom.001G03193-SNP27	5	Potom.005G13333-SNP17	Diameter at breast height	AA	3.82E+00	9.71E-03
2	Potom.002G05522-SNP62	13	Potom.013G29538-SNP4	Lignin content	DD	-3.72E+00	9.70E-03
5	Pto-Wuschela-SNP10	8	Potom.008G22699-SNP72	Holocellulose content	AA	6.40E+00	9.70E-03
14	Potom.014G31981-SNP25	16	Potom.016G34352-SNP58	Holocellulose content	AD	-2.02E+01	9.70E-03
8	Potom.008G22699-SNP70	20	Potom.003G08321-SNP26	Microfiber angle	DD	8.92E+00	9.69E-03
2	Potom.002G05522-SNP23	16	Potom.016G34352-SNP22	Stem volume	AD	3.88E-01	9.68E-03
2	Potom.002G05522-SNP73	5	Potom.005G13176-SNP27	Holocellulose content	AD	9.34E+00	9.68E-03
5	Potom.005G13176-SNP183	20	Potom.003G08321-SNP37	Lignin content	DD	6.78E+00	9.68E-03
10	Potom.010G25706-SNP45	20	Potom.003G09473-SNP9	Lignin content	AD	5.11E+00	9.68E-03
20	Potom.003G08321-SNP17	20	Potom.003G09473-SNP2	α-cellulose content	AD	-8.30E+00	9.68E-03
1	Potom.001G03193-SNP20	16	Potom.016G33876-SNP73	Diameter at breast height	AD	-7.92E+00	9.67E-03
2	Potom.002G07337-SNP15	8	Potom.008G21326-SNP69	Fiber length	DA	-6.88E-02	9.67E-03
2	Potom.002G07963-SNP70	5	Potom.005G13176-SNP44	Holocellulose content	DA	1.25E+01	9.67E-03
2	Potom.002G07963-SNP71	5	Potom.005G13176-SNP44	Holocellulose content	DA	1.25E+01	9.67E-03
5	Pto-Wuschela-SNP5	8	Potom.008G22699-SNP72	Fiber width	AA	-1.80E+00	9.67E-03
2	Potom.002G05522-SNP12	20	Potom.003G08321-SNP38	Stem volume	AA	-5.04E-01	9.65E-03
2	Potom.002G05522-SNP15	8	Potom.008G22699-SNP100	Stem volume	DA	3.13E-01	9.65E-03
2	Potom.002G05522-SNP73	16	Potom.016G34352-SNP58	Holocellulose content	AD	1.24E+01	9.65E-03
1	Potom.001G01788-SNP70	13	Potom.013G29538-SNP72	Fiber length	DA	-9.19E-02	9.64E-03
1	Potom.001G03353-SNP25	2	Potom.002G05522-SNP62	Stem volume	AA	2.20E-01	9.64E-03
2	Potom.002G05522-SNP1	5	Potom.005G13333-SNP13	Hemicellulose content	DA	-1.58E+01	9.64E-03
2	Potom.002G05769-SNP25	20	Potom.003G08321-SNP26	Diameter at breast height	DD	-1.14E+01	9.64E-03
13	Potom.013G29538-SNP9	20	Potom.003G08321-SNP14	α-cellulose content	DD	2.95E+01	9.64E-03
5	Potom.005G13333-SNP17	16	Potom.016G34352-SNP98	Stem volume	DA	-2.37E-01	9.63E-03

8	Potom.008G21326-SNP20	16	Potom.016G33891-SNP26	Stem volume	AA	-4.21E-01	9.63E-03
2	Potom.002G05522-SNP62	8	Potom.008G22699-SNP98	Stem volume	AD	6.92E-01	9.62E-03
2	Potom.002G05522-SNP62	20	Potom.003G08321-SNP14	α-cellulose content	DD	2.95E+01	9.61E-03
8	Potom.008G21952-SNP16	8	Potom.008G22699-SNP68	α-cellulose content	DD	2.73E+01	9.61E-03
2	Potom.002G07206-SNP24	8	Potom.008G22699-SNP22	Holocellulose content	AA	-9.58E+00	9.60E-03
5	Potom.005G13176-SNP16	5	Potom.005G13176-SNP284	Tree height	AD	-3.61E+00	9.59E-03
16	Potom.016G33891-SNP29	20	Potom.003G09473-SNP2	Fiber length	AD	1.21E-01	9.59E-03
1	Potom.001G01788-SNP70	16	Potom.016G33876-SNP25	Diameter at breast height	DA	-1.16E+01	9.58E-03
2	Potom.002G05522-SNP4	10	Potom.010G24967-SNP45	Fiber length	AA	4.90E-02	9.57E-03
2	Potom.002G05522-SNP60	5	Potom.005G13333-SNP12	Fiber length	DD	1.16E-01	9.57E-03
13	Potom.013G29538-SNP72	20	Potom.003G09473-SNP2	Stem volume	DA	3.93E-01	9.57E-03
2	Potom.002G07337-SNP1	5	Potom.005G13966-SNP77	Tree height	AA	-2.56E+00	9.56E-03
2	Potom.002G07337-SNP147	5	Pto-Wuschela-SNP5	Tree height	DA	-3.78E+00	9.56E-03
1	Potom.001G03193-SNP20	2	Potom.002G05522-SNP4	Hemicellulose content	DA	-8.09E+00	9.54E-03
1	Potom.001G03193-SNP27	8	Potom.008G22699-SNP3	Diameter at breast height	AA	4.84E+00	9.54E-03
5	Potom.005G13333-SNP13	16	Potom.016G33891-SNP15	Fiber width	DD	-4.08E+00	9.54E-03
16	Potom.016G33891-SNP26	20	Potom.003G08321-SNP26	Fiber length	DD	-1.72E-01	9.54E-03
2	Potom.002G05522-SNP4	5	Potom.005G13176-SNP62	Microfiber angle	AA	3.12E+00	9.53E-03
1	Potom.001G03606-SNP27	8	Potom.008G22699-SNP71	Holocellulose content	AA	1.32E+01	9.52E-03
2	Potom.002G07337-SNP229	5	Potom.005G13176-SNP162	Diameter at breast height	AA	-3.59E+00	9.51E-03
16	Potom.016G33876-SNP31	18	Potom.018G36220-SNP81	Holocellulose content	DD	1.94E+01	9.51E-03
1	Potom.001G03193-SNP31	10	Potom.010G25294-SNP212	Tree height	DD	-6.29E+00	9.50E-03
5	Potom.005G13176-SNP41	5	Potom.005G13333-SNP12	Tree height	AD	2.43E+00	9.50E-03
10	Potom.010G25700-SNP5	13	Potom.013G29538-SNP15	Microfiber angle	AA	-3.52E+00	9.50E-03
12	Potom.012G28819-SNP27	20	Potom.003G09473-SNP11	Hemicellulose content	AA	-1.10E+01	9.50E-03
1	Potom.001G03193-SNP20	13	Potom.013G29538-SNP72	Stem volume	DA	-4.84E-01	9.49E-03

2	Potom.002G05769-SNP57	12	Potom.012G28819-SNP8	Microfiber angle	AA	5.91E+00	9.49E-03
2	Potom.002G07337-SNP229	10	Potom.010G25398-SNP26	α-cellulose content	AD	6.63E+00	9.49E-03
1	Potom.001G03353-SNP25	20	Potom.003G08321-SNP17	Lignin content	AA	1.46E+00	9.47E-03
2	Potom.002G05522-SNP73	20	Potom.003G09473-SNP13	Lignin content	AA	2.10E+00	9.47E-03
2	Potom.002G07337-SNP34	5	Potom.005G13176-SNP47	Hemicellulose content	AD	-4.89E+00	9.47E-03
13	Potom.013G29538-SNP135	20	Potom.003G08321-SNP17	Hemicellulose content	DD	1.72E+01	9.47E-03
5	Potom.005G13333-SNP18	8	Potom.008G22699-SNP74	Lignin content	DD	4.52E+00	9.46E-03
8	Potom.008G21326-SNP55	10	Potom.010G24967-SNP58	Fiber length	DA	-1.85E-01	9.46E-03
2	Potom.002G05522-SNP23	10	Potom.010G25294-SNP173	Microfiber angle	AA	1.74E+00	9.45E-03
2	Potom.002G05769-SNP43	20	Potom.003G10089-SNP11	Hemicellulose content	AA	1.21E+01	9.45E-03
1	Potom.001G03353-SNP25	2	Potom.002G05769-SNP13	Holocellulose content	AA	-9.58E+00	9.44E-03
2	Potom.002G05522-SNP12	20	Potom.003G08321-SNP15	α-cellulose content	AD	1.44E+01	9.43E-03
2	Potom.002G07337-SNP34	16	Potom.016G33891-SNP15	α-cellulose content	DD	1.64E+01	9.43E-03
8	Potom.008G21326-SNP52	16	Potom.016G33891-SNP26	Stem volume	AA	-4.58E-01	9.43E-03
5	Potom.005G13176-SNP4	16	Potom.016G33876-SNP31	α-cellulose content	DD	1.80E+01	9.42E-03
5	Potom.005G13176-SNP117	8	Potom.008G22699-SNP108	Fiber width	DA	2.23E+00	9.42E-03
8	Potom.008G22699-SNP108	12	Potom.012G28819-SNP46	α-cellulose content	AD	9.41E+00	9.42E-03
2	Potom.002G07963-SNP147	16	Potom.016G33891-SNP29	Holocellulose content	AD	-1.05E+01	9.41E-03
2	Potom.002G07963-SNP231	5	Potom.005G13176-SNP15	Hemicellulose content	AA	8.83E+00	9.41E-03
2	Potom.002G07963-SNP185	16	Potom.016G33891-SNP6	Stem volume	AA	-2.07E-01	9.40E-03
10	Potom.010G24967-SNP45	20	Potom.003G08321-SNP10	Microfiber angle	DA	-3.29E+00	9.39E-03
1	Potom.001G00363-SNP21	8	Potom.008G22699-SNP108	Fiber length	AD	1.28E-01	9.38E-03
1	Potom.001G03606-SNP27	8	Potom.008G22699-SNP38	Stem volume	AA	-6.03E-01	9.38E-03
5	Potom.005G13176-SNP2	16	Potom.016G33876-SNP31	Stem volume	DA	-4.68E-01	9.38E-03
1	Potom.001G01788-SNP70	16	Potom.016G33876-SNP73	Diameter at breast height	DA	-4.95E+00	9.37E-03
1	Potom.001G03606-SNP27	8	Potom.008G22699-SNP38	Tree height	DA	6.17E+00	9.36E-03

2	Potom.002G07337-SNP16	20	Potom.003G10089-SNP7	Diameter at breast height	DA	1.01E+01	9.36E-03
2	Potom.002G05522-SNP54	8	Potom.008G22699-SNP74	Stem volume	DD	7.29E-01	9.35E-03
2	Potom.002G05522-SNP73	5	Potom.005G13333-SNP12	Fiber length	DD	1.42E-01	9.33E-03
2	Potom.002G05769-SNP70	2	Potom.002G07963-SNP170	Stem volume	AA	-2.09E-01	9.33E-03
2	Potom.002G07337-SNP15	2	Potom.002G07963-SNP153	Stem volume	DA	-6.42E-01	9.33E-03
5	Potom.005G13176-SNP117	8	Potom.008G21952-SNP16	α-cellulose content	DD	1.90E+01	9.33E-03
1	Potom.001G03606-SNP26	10	Potom.010G25706-SNP46	Lignin content	AA	1.88E+00	9.32E-03
2	Potom.002G05522-SNP13	8	Potom.008G22516-SNP12	Fiber width	AD	5.61E+00	9.32E-03
2	Potom.002G05522-SNP14	8	Potom.008G22516-SNP12	Fiber width	AD	5.61E+00	9.32E-03
13	Potom.013G29538-SNP5	20	Potom.003G09473-SNP10	Holocellulose content	AA	1.45E+01	9.31E-03
20	Potom.003G08321-SNP9	20	Potom.003G08321-SNP20	Tree height	DD	-5.87E+00	9.31E-03
8	Potom.008G21326-SNP10	12	Potom.012G28819-SNP9	Tree height	AD	-2.99E+00	9.30E-03
12	Potom.012G28819-SNP8	16	Potom.016G33891-SNP6	Stem volume	AA	-5.39E-01	9.30E-03
13	Potom.013G29538-SNP72	16	Potom.016G34352-SNP108	Fiber length	AA	-5.46E-02	9.30E-03
2	Potom.002G05769-SNP57	2	Potom.002G07963-SNP229	Hemicellulose content	DD	-2.21E+01	9.29E-03
2	Potom.002G07337-SNP1	5	Potom.005G13333-SNP18	α-cellulose content	AA	-3.49E+00	9.29E-03
16	Potom.016G33891-SNP28	20	Potom.003G09473-SNP11	Diameter at breast height	DD	-1.21E+01	9.29E-03
13	Potom.013G29538-SNP72	20	Potom.003G09473-SNP2	Fiber length	DA	7.69E-02	9.28E-03
2	Potom.002G05522-SNP12	13	Potom.013G29538-SNP5	Diameter at breast height	AD	-6.06E+00	9.27E-03
2	Potom.002G05522-SNP15	14	Potom.014G31981-SNP26	Fiber length	AA	-8.93E-02	9.27E-03
2	Potom.002G07963-SNP72	20	Potom.003G08321-SNP7	Fiber length	AA	3.89E-02	9.27E-03
2	Potom.002G07963-SNP231	16	Potom.016G34352-SNP44	Diameter at breast height	AA	-8.65E+00	9.27E-03
5	Potom.005G13176-SNP26	13	Potom.013G29538-SNP71	α-cellulose content	DD	1.98E+01	9.25E-03
2	Potom.002G05522-SNP54	5	Potom.005G13333-SNP12	Stem volume	AD	4.71E-01	9.24E-03
1	Potom.001G01788-SNP70	5	Potom.005G13966-SNP74	Fiber length	DA	-1.10E-01	9.23E-03
1	Potom.001G03193-SNP20	2	Potom.002G05522-SNP62	Holocellulose content	AA	7.03E+00	9.23E-03

1	Potom.001G03193-SNP26	10	Potom.010G25398-SNP26	Diameter at breast height	AD	-7.24E+00	9.23E-03
13	Potom.013G29538-SNP4	20	Potom.003G09473-SNP13	Stem volume	AA	4.50E-01	9.23E-03
1	Potom.001G03193-SNP27	1	Potom.001G03606-SNP27	Holocellulose content	AA	1.31E+01	9.22E-03
10	Potom.010G24967-SNP67	16	Potom.016G33891-SNP28	Diameter at breast height	AD	-7.25E+00	9.22E-03
10	Potom.010G24967-SNP43	20	Potom.003G09473-SNP12	Holocellulose content	DA	1.70E+01	9.21E-03
16	Potom.016G33876-SNP20	18	Potom.018G36220-SNP42	Microfiber angle	DA	-4.49E+00	9.21E-03
2	Potom.002G05522-SNP73	8	Potom.008G21952-SNP16	Hemicellulose content	DA	-1.18E+01	9.20E-03
5	Potom.005G13176-SNP16	20	Potom.003G08321-SNP26	α-cellulose content	AD	1.24E+01	9.20E-03
2	Potom.002G05522-SNP62	10	Potom.010G24967-SNP27	Tree height	AA	-1.87E+00	9.19E-03
1	Potom.001G03193-SNP46	5	Pto-Wuschela-SNP5	Diameter at breast height	DA	-6.86E+00	9.18E-03
2	Potom.002G05522-SNP15	2	Potom.002G05522-SNP54	α-cellulose content	AA	9.44E+00	9.16E-03
5	Potom.005G13333-SNP13	13	Potom.013G29538-SNP5	Stem volume	DD	6.18E-01	9.15E-03
10	Potom.010G24967-SNP43	13	Potom.013G29538-SNP72	Hemicellulose content	AD	-8.42E+00	9.15E-03
2	Potom.002G07963-SNP231	5	Potom.005G13176-SNP2	Stem volume	AA	-5.33E-01	9.14E-03
2	Potom.002G07963-SNP147	16	Potom.016G34352-SNP109	Lignin content	AA	2.42E+00	9.13E-03
5	Potom.005G13985-SNP20	8	Potom.008G22699-SNP38	Fiber length	AD	1.60E-01	9.13E-03
2	Potom.002G07337-SNP34	16	Potom.016G33891-SNP17	α-cellulose content	DD	1.77E+01	9.11E-03
5	Potom.005G13966-SNP97	13	Potom.013G29538-SNP9	Stem volume	AD	5.28E-01	9.11E-03
5	Potom.005G13176-SNP27	8	Potom.008G22699-SNP22	Diameter at breast height	AA	-7.23E+00	9.10E-03
5	Potom.005G13333-SNP12	10	Potom.010G25700-SNP13	Fiber length	DA	1.75E-01	9.10E-03
13	Potom.013G29538-SNP4	20	Potom.003G09473-SNP13	Fiber length	AA	9.69E-02	9.10E-03
8	Potom.008G21326-SNP52	13	Potom.013G29538-SNP71	Fiber width	AD	-3.18E+00	9.08E-03
8	Potom.008G22699-SNP22	10	Potom.010G25706-SNP89	Stem volume	DA	-4.37E-01	9.08E-03
1	Potom.001G03193-SNP27	10	Potom.010G25398-SNP5	Holocellulose content	AD	1.38E+01	9.07E-03
5	Potom.005G13333-SNP13	16	Potom.016G33891-SNP15	Diameter at breast height	DD	-1.33E+01	9.07E-03
5	Potom.005G13966-SNP97	10	Potom.010G25398-SNP63	Stem volume	AD	-6.84E-01	9.07E-03

10	Potom.010G24967-SNP43	20	Potom.003G09473-SNP2	Fiber length	DA	7.43E-02	9.06E-03
1	Potom.001G03353-SNP24	20	Potom.003G08321-SNP9	α-cellulose content	AD	-1.46E+01	9.05E-03
2	Potom.002G07963-SNP72	12	Potom.012G28409-SNP6	Holocellulose content	DA	2.14E+01	9.05E-03
1	Potom.001G01788-SNP70	16	Potom.016G33876-SNP94	Fiber length	DA	-7.51E-02	9.04E-03
10	Potom.010G24967-SNP45	14	Potom.014G31981-SNP25	Diameter at breast height	DA	-7.71E+00	9.04E-03
1	Potom.001G00363-SNP21	20	Potom.003G08321-SNP1	Stem volume	AA	-6.92E-01	9.03E-03
2	Potom.002G05522-SNP73	20	Potom.003G08321-SNP17	Lignin content	DA	2.58E+00	9.03E-03
8	Potom.008G21952-SNP16	10	Potom.010G25700-SNP13	Diameter at breast height	AA	-9.31E+00	9.02E-03
5	Potom.005G13176-SNP30	12	Potom.012G28819-SNP8	Diameter at breast height	AA	-6.51E+00	9.01E-03
2	Potom.002G05769-SNP43	14	Potom.014G31981-SNP25	Fiber width	DA	-2.85E+00	9.00E-03
1	Potom.001G03606-SNP27	20	Potom.003G08321-SNP9	Stem volume	AD	7.04E-01	8.99E-03
2	Potom.002G05522-SNP4	10	Potom.010G25398-SNP26	Microfiber angle	DD	-7.00E+00	8.98E-03
8	Potom.008G22516-SNP43	20	Potom.003G08321-SNP5	Microfiber angle	DD	1.04E+01	8.98E-03
2	Potom.002G05769-SNP57	20	Potom.003G08321-SNP32	Hemicellulose content	DA	8.83E+00	8.97E-03
1	Potom.001G03193-SNP10	10	Potom.010G24967-SNP43	Fiber length	AA	-6.01E-02	8.96E-03
5	Potom.005G13985-SNP20	8	Potom.008G22699-SNP72	Stem volume	DD	8.01E-01	8.96E-03
1	Potom.001G00363-SNP23	5	Potom.005G13985-SNP1	α-cellulose content	DD	-2.81E+01	8.95E-03
1	Potom.001G00363-SNP23	5	Potom.005G13985-SNP2	α-cellulose content	DD	-2.81E+01	8.95E-03
2	Potom.002G05769-SNP72	2	Potom.002G07963-SNP170	Diameter at breast height	AA	-3.28E+00	8.93E-03
2	Potom.002G07963-SNP70	18	Potom.018G36220-SNP1	Fiber length	AD	-5.40E-02	8.93E-03
5	Potom.005G13176-SNP2	13	Potom.013G29538-SNP15	Microfiber angle	AA	-3.17E+00	8.93E-03
2	Potom.002G05522-SNP73	20	Potom.003G08321-SNP7	Tree height	AD	1.69E+00	8.92E-03
5	Potom.005G13176-SNP47	5	Potom.005G13333-SNP12	Lignin content	DD	-3.45E+00	8.92E-03
5	Potom.005G13176-SNP117	20	Potom.003G08321-SNP18	Hemicellulose content	DA	-7.35E+00	8.92E-03
8	Potom.008G22699-SNP9	20	Potom.003G09473-SNP13	Diameter at breast height	AD	-9.30E+00	8.92E-03
10	Potom.010G25706-SNP46	20	Potom.003G08321-SNP9	Lignin content	AA	2.31E+00	8.92E-03

13	Potom.013G29538-SNP71	14	Potom.014G31981-SNP26	Fiber width	DA	-2.97E+00	8.92E-03
2	Potom.002G05522-SNP12	10	Potom.010G25398-SNP63	Holocellulose content	AA	-1.41E+01	8.91E-03
5	Potom.005G13176-SNP4	16	Potom.016G34352-SNP98	Holocellulose content	AA	6.67E+00	8.91E-03
5	Potom.005G13966-SNP89	16	Potom.016G33876-SNP94	Diameter at breast height	DA	-9.43E+00	8.90E-03
5	Potom.005G13333-SNP17	10	Potom.010G24967-SNP43	Lignin content	DD	-3.53E+00	8.89E-03
20	Potom.003G08321-SNP3	20	Potom.003G08321-SNP26	Diameter at breast height	DA	3.67E+00	8.89E-03
5	Potom.005G13176-SNP4	13	Potom.013G29538-SNP4	α-cellulose content	DA	1.18E+01	8.88E-03
14	Potom.014G31981-SNP26	20	Potom.003G09473-SNP13	Diameter at breast height	AA	-6.90E+00	8.88E-03
1	Potom.001G03193-SNP20	10	Potom.010G25398-SNP26	Holocellulose content	AD	1.03E+01	8.87E-03
1	Potom.001G03193-SNP26	20	Potom.003G09473-SNP2	Stem volume	AD	5.26E-01	8.87E-03
2	Potom.002G05522-SNP60	5	Potom.005G13176-SNP68	Tree height	AA	2.35E+00	8.87E-03
2	Potom.002G05632-SNP14	20	Potom.003G08321-SNP3	Stem volume	AA	1.66E-01	8.86E-03
2	Potom.002G05769-SNP43	14	Potom.014G31981-SNP25	Fiber width	DD	4.34E+00	8.85E-03
8	Potom.008G21952-SNP16	16	Potom.016G34352-SNP109	Hemicellulose content	DD	-1.78E+01	8.85E-03
8	Potom.008G22699-SNP74	16	Potom.016G33891-SNP15	Diameter at breast height	DA	8.02E+00	8.85E-03
1	Potom.001G03193-SNP46	5	Pto-Wuschela-SNP5	Fiber width	AA	-1.01E+00	8.84E-03
5	Potom.005G13176-SNP27	8	Potom.008G22699-SNP22	Stem volume	AA	-5.38E-01	8.84E-03
8	Potom.008G21326-SNP10	10	Potom.010G24967-SNP58	Fiber length	DA	-1.85E-01	8.84E-03
5	Pto-Wuschela-SNP10	5	Potom.005G13333-SNP18	Fiber width	DD	-2.91E+00	8.83E-03
1	Potom.001G03606-SNP26	10	Potom.010G25706-SNP45	Lignin content	AA	1.88E+00	8.81E-03
1	Potom.001G00363-SNP19	5	Pto-Wuschela-SNP10	Lignin content	AA	-1.32E+00	8.78E-03
10	Potom.010G25398-SNP26	13	Potom.013G29538-SNP4	Stem volume	AA	3.35E-01	8.77E-03
1	Potom.001G00363-SNP31	5	Potom.005G13985-SNP4	Fiber length	AD	-2.04E-01	8.76E-03
1	Potom.001G03193-SNP13	8	Potom.008G21952-SNP16	Diameter at breast height	DA	8.84E+00	8.75E-03
1	Potom.001G03193-SNP20	5	Potom.005G13176-SNP2	α-cellulose content	DD	-2.66E+01	8.75E-03
5	Potom.005G13176-SNP2	13	Potom.013G29538-SNP16	Microfiber angle	AA	-3.16E+00	8.75E-03

8	Potom.008G22699-SNP38	20	Potom.003G09473-SNP13	Fiber length	DD	-2.22E-01	8.75E-03
10	Potom.010G24967-SNP45	20	Potom.003G09473-SNP2	Fiber length	AA	-5.16E-02	8.75E-03
2	Potom.002G05522-SNP62	20	Potom.003G09473-SNP11	Stem volume	AD	-4.93E-01	8.73E-03
2	Potom.002G05769-SNP70	5	Potom.005G13333-SNP12	Stem volume	AD	5.91E-01	8.73E-03
2	Potom.002G07337-SNP56	14	Potom.014G31981-SNP25	Holocellulose content	AA	1.52E+01	8.73E-03
5	Potom.005G13333-SNP12	5	Potom.005G13985-SNP5	Fiber length	AD	1.80E-01	8.73E-03
1	Potom.001G03353-SNP25	2	Potom.002G05522-SNP60	Stem volume	DA	-3.52E-01	8.69E-03
5	Potom.005G13333-SNP13	16	Potom.016G33891-SNP15	α-cellulose content	AD	1.39E+01	8.69E-03
5	Potom.005G13333-SNP17	10	Potom.010G24967-SNP45	Lignin content	DD	-3.50E+00	8.69E-03
12	Potom.012G28819-SNP27	13	Potom.013G29538-SNP72	Fiber length	AA	-1.14E-01	8.69E-03
5	Potom.005G13176-SNP44	8	Potom.008G22699-SNP22	Fiber length	DA	-1.13E-01	8.68E-03
8	Potom.008G22699-SNP100	13	Potom.013G29538-SNP16	Stem volume	DD	7.08E-01	8.68E-03
1	Potom.001G00363-SNP19	13	Potom.013G29538-SNP8	α-cellulose content	AD	1.33E+01	8.67E-03
2	Potom.002G05522-SNP60	8	Potom.008G21952-SNP16	Diameter at breast height	AA	-5.36E+00	8.67E-03
2	Potom.002G05522-SNP62	8	Potom.008G21952-SNP16	Diameter at breast height	AA	-5.36E+00	8.67E-03
8	Potom.008G22699-SNP100	13	Potom.013G29538-SNP15	Fiber length	AD	6.08E-02	8.67E-03
13	Potom.013G29538-SNP7	20	Potom.003G08321-SNP18	Fiber width	DA	1.96E+00	8.67E-03
2	Potom.002G05522-SNP1	8	Potom.008G21952-SNP16	Holocellulose content	AD	1.63E+01	8.66E-03
2	Potom.002G07337-SNP229	5	Pto-Wuschela-SNP5	Fiber width	DA	-2.14E+00	8.66E-03
8	Potom.008G21326-SNP69	12	Potom.012G28819-SNP45	Holocellulose content	AD	8.58E+00	8.66E-03
8	Potom.008G22699-SNP74	14	Potom.014G31981-SNP26	α-cellulose content	AA	-5.66E+00	8.66E-03
2	Potom.002G07206-SNP24	2	Potom.002G07963-SNP163	Fiber length	AA	-3.66E-02	8.65E-03
8	Potom.008G21326-SNP69	20	Potom.003G08321-SNP3	Diameter at breast height	AA	4.42E+00	8.65E-03
10	Potom.010G25398-SNP26	10	Potom.010G25706-SNP89	Stem volume	AD	5.43E-01	8.65E-03
10	Potom.010G25398-SNP68	13	Potom.013G29538-SNP72	Hemicellulose content	DA	-2.12E+01	8.65E-03
16	Potom.016G33876-SNP20	20	Potom.003G08321-SNP17	Holocellulose content	DD	1.52E+01	8.65E-03

5	Pto-Wuschela-SNP10	14	Potom.014G31981-SNP26	Hemicellulose content	DA	-1.22E+01	8.64E-03
10	Potom.010G24967-SNP27	16	Potom.016G33891-SNP28	Diameter at breast height	AA	-5.22E+00	8.63E-03
1	Potom.001G03193-SNP27	5	Potom.005G13176-SNP162	Fiber length	DA	9.21E-02	8.62E-03
2	Potom.002G07206-SNP24	2	Potom.002G07337-SNP229	Fiber width	AA	-1.61E+00	8.61E-03
1	Potom.001G03193-SNP26	10	Potom.010G25398-SNP26	Tree height	AD	-3.35E+00	8.60E-03
2	Potom.002G07963-SNP156	12	Potom.012G28409-SNP11	α-cellulose content	AA	-7.97E+00	8.60E-03
1	Potom.001G03193-SNP31	2	Potom.002G05522-SNP60	Tree height	AD	-4.12E+00	8.59E-03
1	Potom.001G00363-SNP54	5	Pto-Wuschela-SNP10	Hemicellulose content	AD	-1.24E+01	8.56E-03
1	Potom.001G00363-SNP55	5	Pto-Wuschela-SNP10	Hemicellulose content	AD	-1.24E+01	8.56E-03
2	Potom.002G05522-SNP15	16	Potom.016G33891-SNP13	Diameter at breast height	AD	1.00E+01	8.55E-03
2	Potom.002G07206-SNP24	10	Potom.010G24967-SNP40	Fiber length	AD	-1.03E-01	8.55E-03
2	Potom.002G07963-SNP72	5	Potom.005G13176-SNP44	Diameter at breast height	AA	-3.13E+00	8.55E-03
5	Potom.005G13176-SNP16	10	Potom.010G24967-SNP43	Diameter at breast height	AD	-6.34E+00	8.55E-03
8	Potom.008G21326-SNP52	10	Potom.010G24967-SNP58	Fiber length	DA	-1.88E-01	8.55E-03
8	Potom.008G22699-SNP72	13	Potom.013G29538-SNP9	Stem volume	AD	5.85E-01	8.55E-03
2	Potom.002G07337-SNP56	5	Potom.005G13966-SNP89	Stem volume	AA	-6.36E-01	8.54E-03
12	Potom.012G28819-SNP45	16	Potom.016G33876-SNP31	Holocellulose content	DA	-8.12E+00	8.54E-03
13	Potom.013G29538-SNP5	13	Potom.013G29538-SNP72	Fiber length	DD	1.25E-01	8.54E-03
1	Potom.001G01788-SNP70	2	Potom.002G07963-SNP170	Fiber width	DD	-4.38E+00	8.53E-03
1	Potom.001G03193-SNP20	8	Potom.008G22699-SNP72	Fiber width	DD	4.64E+00	8.53E-03
1	Potom.001G03606-SNP26	8	Potom.008G22699-SNP3	Stem volume	AD	-5.13E-01	8.53E-03
5	Potom.005G13333-SNP18	20	Potom.003G09473-SNP11	Hemicellulose content	DD	-1.92E+01	8.52E-03
16	Potom.016G33876-SNP20	18	Potom.018G36220-SNP43	Microfiber angle	DA	-4.52E+00	8.52E-03
2	Potom.002G07963-SNP153	5	Potom.005G13176-SNP39	α-cellulose content	AA	8.63E+00	8.51E-03
5	Potom.005G13176-SNP27	16	Potom.016G33876-SNP94	Fiber length	AD	1.17E-01	8.51E-03
8	Potom.008G22699-SNP22	13	Potom.013G29538-SNP72	Stem volume	DA	-5.71E-01	8.51E-03

10	Potom.010G25706-SNP45	20	Potom.003G08321-SNP9	Lignin content	AA	2.31E+00	8.51E-03
2	Potom.002G07963-SNP170	10	Potom.010G24967-SNP27	Diameter at breast height	AD	-4.73E+00	8.50E-03
5	Potom.005G13176-SNP16	12	Potom.012G28409-SNP23	Stem volume	AA	-2.58E-01	8.50E-03
5	Potom.005G13985-SNP5	20	Potom.003G09473-SNP2	Fiber length	DD	1.76E-01	8.50E-03
1	Potom.001G00363-SNP19	16	Potom.016G34352-SNP108	Hemicellulose content	DD	1.81E+01	8.49E-03
1	Potom.001G03353-SNP161	8	Potom.008G22699-SNP3	Diameter at breast height	DD	-9.35E+00	8.49E-03
1	Potom.001G03606-SNP29	10	Potom.010G25398-SNP68	Stem volume	AA	-6.53E-01	8.49E-03
10	Potom.010G25398-SNP68	13	Potom.013G29538-SNP9	Holocellulose content	DA	1.72E+01	8.49E-03
1	Potom.001G03193-SNP26	1	Potom.001G03606-SNP26	Tree height	AD	-5.15E+00	8.48E-03
5	Potom.005G13966-SNP74	13	Potom.013G29538-SNP16	Microfiber angle	AA	-2.92E+00	8.47E-03
10	Potom.010G25294-SNP212	20	Potom.003G10089-SNP11	Lignin content	AD	-3.55E+00	8.47E-03
9	Potom.009G23029-SNP3	13	Potom.013G29538-SNP5	Stem volume	AD	2.94E-01	8.46E-03
5	Pto-Wuschela-SNP5	20	Potom.003G08321-SNP3	Holocellulose content	AA	-6.46E+00	8.45E-03
1	Potom.001G03606-SNP27	10	Potom.010G24967-SNP58	Holocellulose content	AA	1.92E+01	8.44E-03
2	Potom.002G05769-SNP72	10	Potom.010G25706-SNP89	Stem volume	AD	6.04E-01	8.44E-03
5	Potom.005G13176-SNP44	5	Potom.005G13966-SNP90	Fiber length	AA	1.12E-01	8.44E-03
8	Potom.008G22699-SNP100	13	Potom.013G29538-SNP16	Fiber length	AD	6.03E-02	8.44E-03
10	Potom.010G24967-SNP43	20	Potom.003G09473-SNP2	Stem volume	AD	4.51E-01	8.44E-03
2	Potom.002G05522-SNP3	2	Potom.002G07337-SNP15	Diameter at breast height	AD	-8.99E+00	8.43E-03
2	Potom.002G05769-SNP57	14	Potom.014G31981-SNP78	Diameter at breast height	AA	7.46E+00	8.43E-03
8	Potom.008G22699-SNP108	16	Potom.016G33891-SNP13	Fiber length	AA	-9.68E-02	8.43E-03
13	Potom.013G29538-SNP71	20	Potom.003G08321-SNP3	Hemicellulose content	AD	-7.38E+00	8.43E-03
1	Potom.001G01788-SNP70	16	Potom.016G33876-SNP71	Diameter at breast height	DA	-4.92E+00	8.42E-03
5	Potom.005G13966-SNP74	13	Potom.013G29538-SNP15	Microfiber angle	AA	-2.98E+00	8.42E-03
5	Pto-Wuschela-SNP5	20	Potom.003G08321-SNP6	Fiber width	AA	1.51E+00	8.41E-03
13	Potom.013G29538-SNP5	20	Potom.003G09473-SNP2	α-cellulose content	AA	-4.77E+00	8.41E-03

13	Potom.013G29538-SNP5	20	Potom.003G09473-SNP12	Holocellulose content	AA	1.40E+01	8.41E-03
1	Potom.001G03193-SNP26	2	Potom.002G05522-SNP60	Lignin content	AA	-2.24E+00	8.40E-03
2	Potom.002G07337-SNP15	2	Potom.002G07963-SNP152	Diameter at breast height	DA	-9.00E+00	8.40E-03
5	Potom.005G13333-SNP18	13	Potom.013G29538-SNP14	Holocellulose content	DA	2.38E+01	8.40E-03
8	Potom.008G22699-SNP108	13	Potom.013G29538-SNP14	Holocellulose content	AA	1.21E+01	8.39E-03
16	Potom.016G33876-SNP94	20	Potom.003G08321-SNP3	Holocellulose content	DA	-1.32E+01	8.39E-03
2	Potom.002G05522-SNP60	20	Potom.003G09473-SNP12	Hemicellulose content	AA	9.60E+00	8.38E-03
2	Potom.002G07963-SNP229	20	Potom.003G10089-SNP7	Hemicellulose content	DA	1.73E+01	8.38E-03
10	Potom.010G25706-SNP89	13	Potom.013G29538-SNP7	Stem volume	DA	4.68E-01	8.38E-03
13	Potom.013G29538-SNP8	20	Potom.003G08321-SNP18	Hemicellulose content	DD	1.86E+01	8.38E-03
2	Potom.002G05522-SNP13	8	Potom.008G21952-SNP16	Holocellulose content	AD	1.82E+01	8.37E-03
2	Potom.002G05522-SNP14	8	Potom.008G21952-SNP16	Holocellulose content	AD	1.82E+01	8.37E-03
2	Potom.002G05522-SNP15	10	Potom.010G25398-SNP26	Diameter at breast height	AD	-7.76E+00	8.37E-03
2	Potom.002G05769-SNP25	8	Potom.008G22699-SNP108	Fiber length	AA	-6.22E-02	8.37E-03
1	Potom.001G03353-SNP25	2	Potom.002G05522-SNP54	Diameter at breast height	DA	-7.21E+00	8.35E-03
5	Pto-Wuschela-SNP5	20	Potom.003G08321-SNP3	Fiber width	AD	-2.35E+00	8.35E-03
12	Potom.012G28819-SNP27	20	Potom.003G09473-SNP13	Hemicellulose content	AA	-1.09E+01	8.35E-03
8	Potom.008G21952-SNP16	10	Potom.010G25294-SNP212	Stem volume	AA	-4.95E-01	8.34E-03
2	Potom.002G07337-SNP229	20	Potom.003G08321-SNP2	Tree height	DA	4.00E+00	8.33E-03
16	Potom.016G33891-SNP28	20	Potom.003G09473-SNP13	Diameter at breast height	DD	-1.23E+01	8.32E-03
1	Potom.001G03606-SNP27	20	Potom.003G08321-SNP13	Lignin content	AA	3.58E+00	8.31E-03
2	Potom.002G05522-SNP23	2	Potom.002G07963-SNP153	Diameter at breast height	DA	-7.01E+00	8.31E-03
2	Potom.002G07963-SNP185	5	Potom.005G13966-SNP74	α-cellulose content	DA	1.64E+01	8.31E-03
10	Potom.010G25398-SNP38	16	Potom.016G33891-SNP17	Fiber width	AD	-1.79E+00	8.31E-03
1	Potom.001G00363-SNP23	20	Potom.003G09473-SNP9	Stem volume	DD	-1.19E+00	8.29E-03
2	Potom.002G05522-SNP54	8	Potom.008G22699-SNP72	Hemicellulose content	AD	1.32E+01	8.29E-03

2	Potom.002G05522-SNP15	2	Potom.002G05522-SNP54	α-cellulose content	DA	-1.01E+01	8.28E-03
5	Potom.005G13966-SNP74	12	Potom.012G28409-SNP23	Hemicellulose content	AD	1.37E+01	8.28E-03
10	Potom.010G24967-SNP43	20	Potom.003G09473-SNP2	Holocellulose content	DD	1.76E+01	8.28E-03
20	Potom.003G08321-SNP17	20	Potom.003G09473-SNP2	α-cellulose content	DD	-1.34E+01	8.28E-03
2	Potom.002G05522-SNP60	20	Potom.003G09473-SNP11	Stem volume	AD	-4.96E-01	8.27E-03
5	Potom.005G13333-SNP13	20	Potom.003G08321-SNP21	Hemicellulose content	DD	-1.53E+01	8.26E-03
5	Potom.005G13966-SNP90	8	Potom.008G22699-SNP3	Tree height	DD	-6.81E+00	8.25E-03
1	Potom.001G03193-SNP10	20	Potom.003G10089-SNP11	Hemicellulose content	AD	-3.64E+00	8.24E-03
1	Potom.001G03193-SNP20	16	Potom.016G33876-SNP20	Diameter at breast height	DA	-4.13E+00	8.24E-03
1	Potom.001G03353-SNP25	16	Potom.016G33891-SNP27	Fiber length	DD	-2.17E-01	8.24E-03
2	Potom.002G07206-SNP24	8	Potom.008G22699-SNP9	Holocellulose content	AA	-9.65E+00	8.24E-03
5	Potom.005G13966-SNP89	10	Potom.010G25398-SNP26	Stem volume	DD	-1.04E+00	8.24E-03
5	Pto-Wuschela-SNP10	20	Potom.003G08321-SNP6	α-cellulose content	AA	-4.60E+00	8.23E-03
8	Potom.008G22699-SNP108	14	Potom.014G31981-SNP26	Fiber length	AA	-5.95E-02	8.22E-03
10	Potom.010G24967-SNP43	20	Potom.003G08321-SNP10	Lignin content	DD	-4.10E+00	8.22E-03
1	Potom.001G03193-SNP20	13	Potom.013G29538-SNP72	Holocellulose content	DD	1.69E+01	8.21E-03
2	Potom.002G05522-SNP60	5	Potom.005G13176-SNP155	Microfiber angle	AD	-5.02E+00	8.21E-03
2	Potom.002G07337-SNP56	5	Potom.005G13176-SNP2	α-cellulose content	AD	1.80E+01	8.20E-03
1	Potom.001G00363-SNP19	13	Potom.013G29538-SNP6	α-cellulose content	AD	1.33E+01	8.18E-03
5	Pto-Wuschela-SNP12	5	Potom.005G13176-SNP162	Holocellulose content	DD	-1.45E+01	8.18E-03
8	Potom.008G22699-SNP69	16	Potom.016G33891-SNP28	Stem volume	AD	5.99E-01	8.18E-03
1	Potom.001G03193-SNP26	5	Potom.005G13333-SNP12	α-cellulose content	DA	-1.27E+01	8.17E-03
5	Potom.005G13966-SNP97	10	Potom.010G25398-SNP63	Stem volume	DD	-9.97E-01	8.16E-03
8	Potom.008G22699-SNP22	20	Potom.003G08321-SNP17	α-cellulose content	DD	1.71E+01	8.16E-03
5	Potom.005G13966-SNP89	5	Potom.005G13985-SNP1	Fiber length	AD	-1.51E-01	8.15E-03
5	Potom.005G13966-SNP89	5	Potom.005G13985-SNP2	Fiber length	AD	-1.51E-01	8.15E-03

8	Potom.008G22699-SNP108	14	Potom.014G31981-SNP25	Fiber length	DD	1.43E-01	8.15E-03
13	Potom.013G29538-SNP71	20	Potom.003G08321-SNP7	Lignin content	AA	1.82E+00	8.14E-03
5	Pto-Wuschela-SNP14	9	Potom.009G23029-SNP3	α-cellulose content	DD	-1.82E+01	8.13E-03
1	Potom.001G03353-SNP24	8	Potom.008G22699-SNP108	Lignin content	DA	3.68E+00	8.12E-03
2	Potom.002G07963-SNP72	5	Potom.005G13176-SNP162	Fiber length	AA	-5.09E-02	8.12E-03
10	Potom.010G25700-SNP13	12	Potom.012G28819-SNP9	Fiber width	AA	-2.54E+00	8.12E-03
2	Potom.002G05522-SNP23	10	Potom.010G25706-SNP89	Stem volume	DD	6.10E-01	8.11E-03
2	Potom.002G07337-SNP34	8	Potom.008G21952-SNP16	Hemicellulose content	AA	-6.54E+00	8.11E-03
5	Potom.005G13176-SNP44	16	Potom.016G34352-SNP98	Tree height	AA	-2.22E+00	8.11E-03
20	Potom.003G08321-SNP10	20	Potom.003G08321-SNP26	Diameter at breast height	AA	-4.13E+00	8.11E-03
1	Potom.001G00363-SNP23	8	Potom.008G21952-SNP16	Diameter at breast height	DA	1.35E+01	8.10E-03
2	Potom.002G05522-SNP15	5	Pto-Wuschela-SNP12	Hemicellulose content	AD	-1.01E+01	8.10E-03
5	Potom.005G13176-SNP44	10	Potom.010G24967-SNP45	Fiber length	DD	-1.15E-01	8.09E-03
16	Potom.016G33891-SNP26	20	Potom.003G08321-SNP26	Diameter at breast height	DA	3.09E+00	8.09E-03
1	Potom.001G03193-SNP27	2	Potom.002G05522-SNP73	Lignin content	AA	1.37E+00	8.08E-03
12	Potom.012G28819-SNP27	20	Potom.003G09473-SNP2	Fiber length	DD	1.83E-01	8.08E-03
1	Potom.001G03193-SNP13	2	Potom.002G05522-SNP12	Stem volume	DA	6.44E-01	8.07E-03
1	Potom.001G03353-SNP164	16	Potom.016G34352-SNP108	Lignin content	DD	-3.46E+00	8.07E-03
10	Potom.010G25706-SNP89	20	Potom.003G08321-SNP3	Stem volume	DA	-4.84E-01	8.07E-03
13	Potom.013G29538-SNP6	20	Potom.003G08321-SNP18	Hemicellulose content	DD	1.86E+01	8.06E-03
16	Potom.016G34352-SNP234	20	Potom.003G09473-SNP12	Diameter at breast height	AA	-8.64E+00	8.06E-03
2	Potom.002G05769-SNP43	14	Potom.014G31981-SNP25	Holocellulose content	AA	1.24E+01	8.05E-03
2	Potom.002G05522-SNP12	2	Potom.002G05522-SNP60	Tree height	AD	-2.61E+00	8.03E-03
2	Potom.002G05522-SNP23	10	Potom.010G25398-SNP63	Holocellulose content	DA	1.55E+01	8.03E-03
5	Potom.005G13176-SNP62	10	Potom.010G24967-SNP45	Diameter at breast height	DA	-4.02E+00	8.03E-03
2	Potom.002G05769-SNP57	2	Potom.002G07337-SNP15	Diameter at breast height	AD	-1.06E+01	8.02E-03

8	Potom.008G21326-SNP69	16	Potom.016G33876-SNP56	Diameter at breast height	AD	-6.55E+00	8.02E-03
1	Potom.001G01788-SNP70	16	Potom.016G33876-SNP108	Diameter at breast height	AA	-2.65E+00	8.01E-03
2	Potom.002G05522-SNP73	8	Potom.008G22699-SNP190	Fiber width	DA	2.45E+00	8.01E-03
5	Pto-Wuschela-SNP14	10	Potom.010G25700-SNP5	Hemicellulose content	DA	-1.25E+01	8.01E-03
5	Potom.005G13966-SNP90	10	Potom.010G25398-SNP26	Tree height	DA	-6.42E+00	8.01E-03
1	Potom.001G03606-SNP26	2	Potom.002G05769-SNP13	Holocellulose content	AD	1.89E+01	7.99E-03
5	Potom.005G13333-SNP12	14	Potom.014G31981-SNP26	Fiber length	DA	1.05E-01	7.99E-03
8	Potom.008G22699-SNP70	20	Potom.003G08321-SNP32	α-cellulose content	AD	1.33E+01	7.99E-03
13	Potom.013G29538-SNP4	20	Potom.003G08321-SNP14	α-cellulose content	DD	2.89E+01	7.99E-03
5	Potom.005G13176-SNP26	8	Potom.008G22699-SNP74	α-cellulose content	DA	-1.47E+01	7.98E-03
5	Potom.005G13176-SNP194	10	Potom.010G25398-SNP26	Fiber length	DA	-1.22E-01	7.98E-03
1	Potom.001G03193-SNP10	8	Potom.008G22699-SNP108	α-cellulose content	AD	-3.94E+00	7.97E-03
2	Potom.002G05522-SNP62	20	Potom.003G09473-SNP12	Hemicellulose content	AA	9.69E+00	7.97E-03
2	Potom.002G07337-SNP15	9	Potom.009G23029-SNP3	α-cellulose content	DA	-5.60E+00	7.97E-03
5	Potom.005G13176-SNP2	10	Potom.010G25706-SNP46	Lignin content	AA	2.36E+00	7.97E-03
5	Potom.005G13333-SNP13	20	Potom.003G08321-SNP17	α-cellulose content	AA	-6.31E+00	7.97E-03
2	Potom.002G05522-SNP12	2	Potom.002G05522-SNP60	Diameter at breast height	AD	-5.71E+00	7.96E-03
16	Potom.016G33891-SNP29	20	Potom.003G08321-SNP21	Holocellulose content	DD	2.13E+01	7.95E-03
1	Potom.001G03353-SNP25	2	Potom.002G05522-SNP12	Holocellulose content	DA	-1.15E+01	7.94E-03
5	Pto-Wuschela-SNP12	20	Potom.003G08321-SNP5	Hemicellulose content	DA	-1.15E+01	7.94E-03
8	Potom.008G22699-SNP22	12	Potom.012G28409-SNP11	Stem volume	AA	-5.77E-01	7.93E-03
5	Potom.005G13176-SNP47	8	Potom.008G22699-SNP150	α-cellulose content	DD	1.85E+01	7.92E-03
5	Potom.005G13176-SNP47	20	Potom.003G08321-SNP6	Fiber width	AA	2.37E+00	7.92E-03
5	Potom.005G13176-SNP2	5	Potom.005G13985-SNP17	Diameter at breast height	AA	-7.97E+00	7.91E-03
12	Potom.012G28409-SNP23	20	Potom.003G08321-SNP3	Stem volume	DA	-5.17E-01	7.91E-03
2	Potom.002G05522-SNP73	10	Potom.010G24967-SNP45	Lignin content	AD	2.28E+00	7.90E-03

8	Potom.008G22699-SNP9	12	Potom.012G28409-SNP23	Stem volume	AA	-3.49E-01	7.90E-03
10	Potom.010G25706-SNP89	12	Potom.012G28819-SNP9	Stem volume	DD	5.90E-01	7.90E-03
2	Potom.002G05522-SNP12	2	Potom.002G05522-SNP62	Diameter at breast height	AD	-5.72E+00	7.89E-03
5	Potom.005G13176-SNP44	12	Potom.012G28409-SNP23	Stem volume	AD	4.06E-01	7.89E-03
10	Potom.010G25706-SNP89	13	Potom.013G29538-SNP8	α-cellulose content	DA	-1.12E+01	7.89E-03
2	Potom.002G05632-SNP14	13	Potom.013G29538-SNP71	Hemicellulose content	AD	-1.18E+01	7.88E-03
2	Potom.002G05632-SNP14	20	Potom.003G08321-SNP3	Diameter at breast height	AA	2.43E+00	7.88E-03
5	Potom.005G13176-SNP2	20	Potom.003G09473-SNP13	α-cellulose content	DD	-2.00E+01	7.88E-03
1	Potom.001G03193-SNP20	18	Potom.018G36220-SNP81	Fiber width	DA	-1.30E+00	7.87E-03
1	Potom.001G03353-SNP25	13	Potom.013G29538-SNP7	Stem volume	DD	-7.54E-01	7.87E-03
2	Potom.002G05522-SNP15	8	Potom.008G22699-SNP108	Stem volume	AD	4.97E-01	7.87E-03
5	Potom.005G13333-SNP13	13	Potom.013G29538-SNP5	Diameter at breast height	DD	9.05E+00	7.87E-03
5	Potom.005G13966-SNP77	16	Potom.016G33891-SNP28	Tree height	AD	-3.55E+00	7.87E-03
10	Potom.010G24967-SNP40	20	Potom.003G09473-SNP11	Fiber length	DA	-1.17E-01	7.87E-03
2	Potom.002G07337-SNP34	16	Potom.016G33891-SNP17	Fiber length	DA	-1.23E-01	7.86E-03
5	Potom.005G13333-SNP17	13	Potom.013G29538-SNP16	Tree height	DD	-4.31E+00	7.86E-03
8	Potom.008G22699-SNP74	13	Potom.013G29538-SNP5	Stem volume	AD	3.02E-01	7.86E-03
1	Potom.001G03353-SNP164	16	Potom.016G34352-SNP98	α-cellulose content	AD	-9.72E+00	7.85E-03
5	Pto-Wuschela-SNP5	5	Potom.005G13176-SNP155	Microfiber angle	AD	-5.09E+00	7.85E-03
10	Potom.010G25294-SNP212	20	Potom.003G10089-SNP11	Holocellulose content	AA	1.24E+01	7.85E-03
12	Potom.012G28819-SNP46	16	Potom.016G33876-SNP94	Hemicellulose content	AD	-1.27E+01	7.85E-03
1	Potom.001G03193-SNP13	2	Potom.002G05522-SNP1	Hemicellulose content	AD	-1.83E+01	7.84E-03
5	Potom.005G13333-SNP12	20	Potom.003G10089-SNP11	Tree height	DA	3.53E+00	7.84E-03
5	Potom.005G13333-SNP17	8	Potom.008G22699-SNP3	Fiber length	DA	6.97E-02	7.84E-03
1	Potom.001G03193-SNP13	8	Potom.008G21952-SNP16	Stem volume	AA	-4.89E-01	7.83E-03
2	Potom.002G05522-SNP60	20	Potom.003G08321-SNP13	α-cellulose content	DD	2.91E+01	7.83E-03

2	Potom.002G05769-SNP57	2	Potom.002G07963-SNP229	Tree height	AA	-3.67E+00	7.83E-03
8	Potom.008G22699-SNP108	12	Potom.012G28819-SNP45	α-cellulose content	AD	8.22E+00	7.83E-03
2	Potom.002G05769-SNP37	8	Potom.008G21326-SNP69	Diameter at breast height	AA	-8.24E+00	7.82E-03
5	Pto-Wuschela-SNP14	20	Potom.003G08321-SNP33	Holocellulose content	DA	-1.43E+01	7.82E-03
5	Potom.005G13176-SNP3	16	Potom.016G33876-SNP31	Stem volume	DA	-3.78E-01	7.82E-03
1	Potom.001G00363-SNP19	2	Potom.002G05522-SNP12	α-cellulose content	DA	-8.94E+00	7.81E-03
1	Potom.001G00363-SNP54	2	Potom.002G05522-SNP12	Hemicellulose content	DD	-2.64E+01	7.81E-03
1	Potom.001G00363-SNP55	2	Potom.002G05522-SNP12	Hemicellulose content	DD	-2.64E+01	7.81E-03
2	Potom.002G07206-SNP24	2	Potom.002G07337-SNP229	Lignin content	DA	1.29E+00	7.81E-03
2	Potom.002G05522-SNP52	5	Potom.005G13176-SNP39	α-cellulose content	AA	-2.34E+00	7.80E-03
1	Potom.001G03193-SNP20	8	Potom.008G22699-SNP72	Holocellulose content	AA	-9.02E+00	7.79E-03
1	Potom.001G03193-SNP30	2	Potom.002G05522-SNP23	Fiber length	AA	-1.10E-01	7.79E-03
2	Potom.002G05769-SNP72	5	Potom.005G13333-SNP12	Diameter at breast height	AD	9.70E+00	7.79E-03
20	Potom.003G08321-SNP20	20	Potom.003G09473-SNP2	Stem volume	DA	5.30E-01	7.79E-03
2	Potom.002G07963-SNP147	16	Potom.016G33891-SNP29	Fiber length	DD	-1.93E-01	7.78E-03
16	Potom.016G33876-SNP20	18	Potom.018G36220-SNP43	Diameter at breast height	AA	-5.25E+00	7.78E-03
1	Potom.001G00363-SNP26	5	Potom.005G13176-SNP4	Diameter at breast height	AD	1.08E+01	7.77E-03
5	Pto-Wuschela-SNP5	8	Potom.008G22699-SNP68	Fiber width	DD	4.79E+00	7.77E-03
8	Potom.008G22699-SNP108	20	Potom.003G09473-SNP25	Fiber width	AD	2.80E+00	7.77E-03
16	Potom.016G33876-SNP72	18	Potom.018G36220-SNP1	Lignin content	DD	4.62E+00	7.76E-03
2	Potom.002G05522-SNP73	5	Potom.005G13176-SNP44	Lignin content	AD	1.12E+00	7.74E-03
2	Potom.002G07337-SNP15	12	Potom.012G28819-SNP8	Fiber length	DA	-1.31E-01	7.74E-03
8	Potom.008G21326-SNP55	12	Potom.012G28819-SNP9	Tree height	AD	-3.12E+00	7.73E-03
8	Potom.008G22699-SNP3	14	Potom.014G31981-SNP25	α-cellulose content	DD	1.65E+01	7.73E-03
8	Potom.008G22699-SNP9	12	Potom.012G28409-SNP23	Fiber width	AA	2.13E+00	7.73E-03
2	Potom.002G05522-SNP62	12	Potom.012G28409-SNP23	Tree height	AD	2.45E+00	7.72E-03

2	Potom.002G07206-SNP24	8	Potom.008G22699-SNP38	Holocellulose content	AA	-1.32E+01	7.72E-03
5	Potom.005G13176-SNP183	16	Potom.016G33876-SNP72	Fiber length	AA	7.05E-02	7.72E-03
18	Potom.018G35976-SNP25	20	Potom.003G09473-SNP2	Tree height	AD	3.04E+00	7.72E-03
2	Potom.002G05769-SNP57	16	Potom.016G34352-SNP108	Hemicellulose content	AA	-8.16E+00	7.71E-03
5	Pto-Wuschela-SNP14	16	Potom.016G33891-SNP26	Holocellulose content	DA	-1.15E+01	7.71E-03
1	Potom.001G03193-SNP27	8	Potom.008G21952-SNP16	Diameter at breast height	AA	-7.45E+00	7.70E-03
2	Potom.002G05522-SNP23	16	Potom.016G34352-SNP22	Diameter at breast height	AD	5.61E+00	7.70E-03
2	Potom.002G07337-SNP229	10	Potom.010G25398-SNP26	Stem volume	AD	-3.22E-01	7.70E-03
5	Pto-Wuschela-SNP5	16	Potom.016G33891-SNP26	Hemicellulose content	AD	7.31E+00	7.70E-03
5	Pto-Wuschela-SNP5	20	Potom.003G08321-SNP7	Fiber width	AA	1.48E+00	7.70E-03
5	Potom.005G13176-SNP62	5	Potom.005G13985-SNP1	Diameter at breast height	DD	-1.33E+01	7.70E-03
5	Potom.005G13176-SNP62	5	Potom.005G13985-SNP2	Diameter at breast height	DD	-1.33E+01	7.70E-03
8	Potom.008G22699-SNP72	12	Potom.012G28409-SNP11	Stem volume	AA	-4.32E-01	7.69E-03
2	Potom.002G05522-SNP1	5	Potom.005G13333-SNP12	Hemicellulose content	DA	-1.57E+01	7.68E-03
2	Potom.002G05522-SNP62	20	Potom.003G08321-SNP13	α-cellulose content	DD	2.91E+01	7.68E-03
16	Potom.016G34352-SNP234	20	Potom.003G09473-SNP10	Diameter at breast height	AA	-8.72E+00	7.68E-03
5	Potom.005G13176-SNP26	13	Potom.013G29538-SNP71	α-cellulose content	DA	1.00E+01	7.67E-03
10	Potom.010G24967-SNP45	20	Potom.003G09473-SNP2	Fiber length	DA	7.39E-02	7.67E-03
10	Potom.010G25706-SNP89	13	Potom.013G29538-SNP6	α-cellulose content	DA	-1.12E+01	7.67E-03
5	Potom.005G13176-SNP27	8	Potom.008G22699-SNP2	Holocellulose content	DD	-3.10E+01	7.66E-03
5	Potom.005G13176-SNP40	8	Potom.008G21326-SNP69	Tree height	DA	-2.39E+00	7.66E-03
12	Potom.012G28819-SNP8	16	Potom.016G33891-SNP15	Fiber width	AD	2.78E+00	7.66E-03
1	Potom.001G03353-SNP25	5	Potom.005G13985-SNP1	Microfiber angle	DA	5.14E+00	7.65E-03
1	Potom.001G03353-SNP25	5	Potom.005G13985-SNP2	Microfiber angle	DA	5.14E+00	7.65E-03
2	Potom.002G05522-SNP23	2	Potom.002G07963-SNP229	Hemicellulose content	DD	-1.85E+01	7.65E-03
2	Potom.002G07206-SNP24	8	Potom.008G22699-SNP108	Fiber length	DD	1.23E-01	7.64E-03

8	Potom.008G21326-SNP69	10	Potom.010G25294-SNP212	Stem volume	AA	-4.15E-01	7.64E-03
5	Potom.005G13985-SNP6	20	Potom.003G08321-SNP2	Holocellulose content	AD	2.04E+01	7.63E-03
16	Potom.016G34352-SNP44	20	Potom.003G08321-SNP3	Holocellulose content	AD	1.93E+01	7.63E-03
18	Potom.018G36220-SNP10	20	Potom.003G08321-SNP10	Diameter at breast height	DA	6.15E+00	7.63E-03
1	Potom.001G03193-SNP20	20	Potom.003G09473-SNP2	α-cellulose content	DA	1.09E+01	7.62E-03
5	Potom.005G13176-SNP27	13	Potom.013G29538-SNP5	Hemicellulose content	DD	2.04E+01	7.62E-03
5	Potom.005G13176-SNP2	10	Potom.010G25706-SNP45	Lignin content	AA	2.36E+00	7.61E-03
2	Potom.002G05522-SNP23	14	Potom.014G31981-SNP26	Holocellulose content	DA	1.24E+01	7.60E-03
2	Potom.002G05522-SNP60	20	Potom.003G08321-SNP32	Stem volume	AA	2.37E-01	7.60E-03
5	Potom.005G13176-SNP4	20	Potom.003G08321-SNP17	α-cellulose content	DA	-1.16E+01	7.60E-03
1	Potom.001G03193-SNP26	8	Potom.008G21326-SNP69	Stem volume	AD	-5.75E-01	7.59E-03
5	Potom.005G13985-SNP6	13	Potom.013G29538-SNP5	Stem volume	AD	-7.24E-01	7.59E-03
2	Potom.002G05522-SNP15	13	Potom.013G29538-SNP10	Stem volume	DD	8.04E-01	7.58E-03
8	Potom.008G22699-SNP74	16	Potom.016G33876-SNP56	Stem volume	DA	2.58E-01	7.58E-03
10	Potom.010G25398-SNP26	10	Potom.010G25398-SNP63	α-cellulose content	DA	1.50E+01	7.58E-03
18	Potom.018G36220-SNP37	20	Potom.003G09473-SNP13	Fiber length	AD	8.29E-02	7.57E-03
1	Potom.001G00363-SNP19	2	Potom.002G05522-SNP12	Holocellulose content	DA	-1.06E+01	7.56E-03
2	Potom.002G05522-SNP60	5	Potom.005G13176-SNP26	Diameter at breast height	DA	-7.64E+00	7.56E-03
1	Potom.001G01788-SNP70	10	Potom.010G25294-SNP173	Fiber width	AD	-3.32E+00	7.55E-03
2	Potom.002G05522-SNP4	2	Potom.002G05522-SNP15	Holocellulose content	AA	-6.61E+00	7.55E-03
2	Potom.002G05769-SNP25	8	Potom.008G21326-SNP69	Diameter at breast height	AA	-5.66E+00	7.55E-03
18	Potom.018G36220-SNP10	20	Potom.003G08321-SNP5	Diameter at breast height	AA	-2.85E+00	7.54E-03
5	Potom.005G13176-SNP41	8	Potom.008G21326-SNP69	Tree height	DA	-2.22E+00	7.53E-03
8	Potom.008G21326-SNP20	12	Potom.012G28819-SNP9	Tree height	AD	-3.08E+00	7.53E-03
1	Potom.001G03193-SNP13	2	Potom.002G07963-SNP231	Stem volume	DA	6.99E-01	7.52E-03
1	Potom.001G03606-SNP26	16	Potom.016G34352-SNP43	Holocellulose content	AA	1.59E+01	7.52E-03

2	Potom.002G05522-SNP12	13	Potom.013G29538-SNP71	Fiber width	DD	-4.20E+00	7.52E-03
2	Potom.002G07206-SNP24	2	Potom.002G07337-SNP229	Diameter at breast height	AA	3.90E+00	7.52E-03
5	Pto-Wuschela-SNP10	16	Potom.016G33876-SNP71	Fiber length	DA	-5.07E-02	7.52E-03
1	Potom.001G03193-SNP13	10	Potom.010G25706-SNP89	Diameter at breast height	DA	-9.14E+00	7.51E-03
2	Potom.002G05522-SNP4	10	Potom.010G24967-SNP43	Holocellulose content	AD	7.91E+00	7.51E-03
16	Potom.016G33891-SNP28	16	Potom.016G34352-SNP108	Diameter at breast height	DD	-1.23E+01	7.50E-03
2	Potom.002G05522-SNP1	20	Potom.003G09473-SNP10	Hemicellulose content	DD	2.79E+01	7.49E-03
2	Potom.002G05769-SNP57	12	Potom.012G28819-SNP9	Diameter at breast height	DA	-9.77E+00	7.49E-03
2	Potom.002G07337-SNP144	8	Potom.008G22699-SNP83	Lignin content	AA	1.42E+00	7.49E-03
2	Potom.002G07337-SNP145	8	Potom.008G22699-SNP83	Lignin content	AA	1.42E+00	7.49E-03
10	Potom.010G24967-SNP27	16	Potom.016G33891-SNP13	Stem volume	AD	4.38E-01	7.48E-03
2	Potom.002G05522-SNP62	5	Potom.005G13176-SNP26	Diameter at breast height	DA	-7.66E+00	7.47E-03
8	Potom.008G21952-SNP16	8	Potom.008G22699-SNP22	Holocellulose content	DA	-1.93E+01	7.47E-03
2	Potom.002G05769-SNP70	2	Potom.002G07963-SNP170	Diameter at breast height	AA	-3.07E+00	7.46E-03
12	Potom.012G28819-SNP45	20	Potom.003G08321-SNP5	Stem volume	AA	-5.10E-01	7.46E-03
10	Potom.010G24967-SNP27	20	Potom.003G08321-SNP10	Diameter at breast height	AD	-7.92E+00	7.43E-03
5	Potom.005G13966-SNP90	8	Potom.008G22699-SNP72	Stem volume	DA	-6.80E-01	7.41E-03
5	Pto-Wuschela-SNP14	5	Potom.005G13966-SNP74	Hemicellulose content	AA	3.91E+00	7.40E-03
5	Potom.005G13176-SNP162	16	Potom.016G34352-SNP110	Microfiber angle	AA	2.05E+00	7.40E-03
5	Pto-Wuschela-SNP5	16	Potom.016G33891-SNP26	Fiber width	AA	-1.35E+00	7.39E-03
20	Potom.003G08321-SNP4	20	Potom.003G09473-SNP2	Diameter at breast height	DA	1.49E+01	7.38E-03
2	Potom.002G07337-SNP15	9	Potom.009G23029-SNP3	Stem volume	AD	-5.40E-01	7.37E-03
2	Potom.002G07337-SNP34	12	Potom.012G28819-SNP45	Fiber length	DA	-1.22E-01	7.37E-03
1	Potom.001G00363-SNP53	2	Potom.002G05522-SNP12	Lignin content	DA	5.74E+00	7.36E-03
1	Potom.001G00363-SNP53	18	Potom.018G35976-SNP20	Hemicellulose content	DD	-2.12E+01	7.36E-03
1	Potom.001G03353-SNP25	10	Potom.010G24967-SNP45	Diameter at breast height	DA	-5.02E+00	7.36E-03

2	Potom.002G05522-SNP23	16	Potom.016G33891-SNP15	Fiber width	DD	3.48E+00	7.36E-03
5	Potom.005G13176-SNP2	14	Potom.014G31981-SNP25	Lignin content	AA	3.27E+00	7.36E-03
10	Potom.010G24967-SNP53	16	Potom.016G33876-SNP108	Microfiber angle	AA	2.72E+00	7.36E-03
2	Potom.002G05769-SNP43	10	Potom.010G24967-SNP53	Fiber width	DD	5.72E+00	7.35E-03
5	Potom.005G13176-SNP2	14	Potom.014G31981-SNP26	Holocellulose content	DA	-1.64E+01	7.35E-03
1	Potom.001G01788-SNP70	16	Potom.016G33876-SNP56	Diameter at breast height	AA	2.53E+00	7.34E-03
2	Potom.002G05522-SNP1	20	Potom.003G09473-SNP13	Holocellulose content	DA	-2.03E+01	7.34E-03
2	Potom.002G05522-SNP1	5	Potom.005G13966-SNP97	Holocellulose content	DD	2.42E+01	7.33E-03
5	Potom.005G13966-SNP89	5	Potom.005G13985-SNP1	Holocellulose content	AD	1.76E+01	7.33E-03
5	Potom.005G13966-SNP89	5	Potom.005G13985-SNP2	Holocellulose content	AD	1.76E+01	7.33E-03
13	Potom.013G29538-SNP4	20	Potom.003G09473-SNP2	Holocellulose content	AA	-5.53E+00	7.33E-03
20	Potom.003G08321-SNP1	20	Potom.003G09473-SNP2	Diameter at breast height	DA	1.46E+01	7.33E-03
1	Potom.001G00363-SNP19	16	Potom.016G34352-SNP109	Hemicellulose content	AD	1.05E+01	7.32E-03
5	Potom.005G13176-SNP154	16	Potom.016G34352-SNP98	Microfiber angle	DA	-3.41E+00	7.32E-03
9	Potom.009G22902-SNP9	20	Potom.003G08321-SNP7	Diameter at breast height	AA	6.19E+00	7.32E-03
10	Potom.010G24967-SNP27	10	Potom.010G24967-SNP67	Diameter at breast height	DA	-4.56E+00	7.32E-03
1	Potom.001G03606-SNP26	8	Potom.008G22699-SNP108	Lignin content	DA	-3.40E+00	7.31E-03
5	Potom.005G13176-SNP16	10	Potom.010G24967-SNP45	Diameter at breast height	AD	-6.46E+00	7.31E-03
13	Potom.013G29538-SNP5	20	Potom.003G08321-SNP14	α-cellulose content	DD	2.96E+01	7.31E-03
8	Potom.008G21326-SNP19	12	Potom.012G28819-SNP9	Tree height	AD	-3.15E+00	7.30E-03
8	Potom.008G21326-SNP55	16	Potom.016G33891-SNP26	Diameter at breast height	AA	-6.74E+00	7.30E-03
8	Potom.008G21326-SNP66	20	Potom.003G08321-SNP3	Diameter at breast height	AA	5.48E+00	7.30E-03
1	Potom.001G01788-SNP70	2	Potom.002G05769-SNP70	Fiber length	AA	-6.37E-02	7.29E-03
5	Pto-Wuschela-SNP18	5	Potom.005G13176-SNP162	Lignin content	AD	1.23E+00	7.29E-03
8	Potom.008G21326-SNP69	20	Potom.003G09473-SNP7	Holocellulose content	AA	1.05E+01	7.29E-03
5	Potom.005G13176-SNP2	20	Potom.003G09473-SNP11	α-cellulose content	DD	-2.00E+01	7.27E-03

2	Potom.002G05522-SNP73	5	Potom.005G13333-SNP12	Hemicellulose content	DD	1.34E+01	7.26E-03
5	Potom.005G13985-SNP1	14	Potom.014G31981-SNP26	Fiber length	DA	-1.52E-01	7.25E-03
5	Potom.005G13985-SNP2	14	Potom.014G31981-SNP26	Fiber length	DA	-1.52E-01	7.25E-03
8	Potom.008G22699-SNP98	16	Potom.016G33876-SNP56	Holocellulose content	DA	-1.56E+01	7.25E-03
13	Potom.013G29538-SNP8	20	Potom.003G08321-SNP18	Stem volume	AD	5.52E-01	7.25E-03
2	Potom.002G07206-SNP24	12	Potom.012G28819-SNP27	Diameter at breast height	DD	-1.41E+01	7.24E-03
5	Potom.005G13176-SNP2	13	Potom.013G29538-SNP4	Holocellulose content	AA	8.90E+00	7.24E-03
8	Potom.008G21326-SNP55	16	Potom.016G33891-SNP26	Stem volume	AA	-4.74E-01	7.24E-03
1	Potom.001G03193-SNP46	5	Pto-Wuschela-SNP14	Holocellulose content	AA	1.11E+01	7.23E-03
2	Potom.002G05522-SNP3	20	Potom.003G09473-SNP2	Stem volume	AA	-5.02E-01	7.23E-03
2	Potom.002G05522-SNP4	2	Potom.002G05522-SNP12	Holocellulose content	DA	-9.86E+00	7.23E-03
2	Potom.002G05522-SNP23	20	Potom.003G08321-SNP21	Fiber length	AA	4.18E-02	7.23E-03
2	Potom.002G05769-SNP57	20	Potom.003G08321-SNP7	Stem volume	AA	4.88E-01	7.23E-03
8	Potom.008G22699-SNP38	8	Potom.008G22699-SNP74	Stem volume	AD	6.73E-01	7.23E-03
13	Potom.013G29538-SNP7	16	Potom.016G33891-SNP29	Diameter at breast height	AA	-6.01E+00	7.23E-03
20	Potom.003G08321-SNP6	20	Potom.003G08321-SNP17	Tree height	DA	-2.69E+00	7.23E-03
10	Potom.010G25398-SNP65	12	Potom.012G28409-SNP23	Stem volume	AA	-2.21E-01	7.22E-03
1	Potom.001G00363-SNP19	2	Potom.002G07337-SNP16	Stem volume	AD	-4.74E-01	7.21E-03
2	Potom.002G07206-SNP38	20	Potom.003G08321-SNP2	Fiber length	DD	-2.16E-01	7.21E-03
1	Potom.001G03606-SNP26	2	Potom.002G07963-SNP231	Holocellulose content	DA	-1.93E+01	7.20E-03
2	Potom.002G05522-SNP4	10	Potom.010G24967-SNP45	Holocellulose content	AD	7.86E+00	7.20E-03
2	Potom.002G07337-SNP56	5	Potom.005G13176-SNP15	Hemicellulose content	AD	1.59E+01	7.20E-03
2	Potom.002G07963-SNP152	5	Pto-Wuschela-SNP5	Hemicellulose content	AA	-8.26E+00	7.20E-03
5	Potom.005G13176-SNP115	13	Potom.013G29538-SNP71	Hemicellulose content	DA	-1.27E+01	7.20E-03
10	Potom.010G25706-SNP79	12	Potom.012G28819-SNP9	Tree height	DA	-2.92E+00	7.20E-03
2	Potom.002G05522-SNP12	20	Potom.003G09473-SNP2	Diameter at breast height	AD	9.00E+00	7.19E-03

2	Potom.002G05769-SNP25	20	Potom.003G08321-SNP26	Stem volume	DD	-8.46E-01	7.19E-03
13	Potom.013G29538-SNP8	20	Potom.003G08321-SNP17	α-cellulose content	AA	7.62E+00	7.19E-03
2	Potom.002G05769-SNP59	9	Potom.009G22902-SNP9	Diameter at breast height	AA	-8.89E+00	7.17E-03
5	Pto-Wuschela-SNP14	20	Potom.003G10089-SNP11	Lignin content	DA	3.07E+00	7.17E-03
8	Potom.008G22699-SNP108	18	Potom.018G36220-SNP43	Fiber length	DA	6.09E-02	7.17E-03
10	Potom.010G25398-SNP26	20	Potom.003G09473-SNP2	Tree height	DA	3.27E+00	7.17E-03
20	Potom.003G08321-SNP10	20	Potom.003G08321-SNP32	Fiber length	DA	1.06E-01	7.16E-03
1	Potom.001G03193-SNP13	16	Potom.016G33891-SNP29	Diameter at breast height	DA	1.15E+01	7.15E-03
8	Potom.008G22699-SNP85	10	Potom.010G25398-SNP5	Holocellulose content	AA	6.20E+00	7.15E-03
2	Potom.002G07337-SNP229	16	Potom.016G33891-SNP28	Fiber width	DA	-3.18E+00	7.14E-03
8	Potom.008G22699-SNP3	20	Potom.003G08321-SNP1	Stem volume	DD	1.23E+00	7.14E-03
8	Potom.008G22699-SNP108	18	Potom.018G36220-SNP42	Tree height	DA	2.14E+00	7.14E-03
13	Potom.013G29538-SNP6	20	Potom.003G08321-SNP18	Stem volume	AD	5.49E-01	7.12E-03
5	Potom.005G13333-SNP13	13	Potom.013G29538-SNP5	Holocellulose content	AA	6.62E+00	7.11E-03
5	Potom.005G13333-SNP13	13	Potom.013G29538-SNP5	Stem volume	AA	-2.85E-01	7.11E-03
13	Potom.013G29538-SNP8	20	Potom.003G08321-SNP17	Stem volume	AA	-3.19E-01	7.11E-03
10	Potom.010G25398-SNP26	20	Potom.003G08321-SNP18	Fiber width	AA	1.23E+00	7.10E-03
2	Potom.002G05522-SNP60	20	Potom.003G09473-SNP10	Hemicellulose content	AA	9.61E+00	7.09E-03
2	Potom.002G07337-SNP15	16	Potom.016G33891-SNP13	Microfiber angle	DD	-9.30E+00	7.08E-03
5	Potom.005G13176-SNP23	16	Potom.016G34352-SNP44	Holocellulose content	AD	-2.37E+01	7.08E-03
5	Potom.005G13966-SNP97	20	Potom.003G09473-SNP9	Hemicellulose content	DA	-1.70E+01	7.08E-03
16	Potom.016G33891-SNP15	20	Potom.003G08321-SNP26	α-cellulose content	AD	1.34E+01	7.07E-03
2	Potom.002G05522-SNP3	2	Potom.002G05522-SNP4	Hemicellulose content	DD	1.85E+01	7.06E-03
5	Potom.005G13176-SNP2	8	Potom.008G22699-SNP74	Holocellulose content	AA	-7.30E+00	7.06E-03
1	Potom.001G00363-SNP19	2	Potom.002G05522-SNP4	Microfiber angle	DA	3.50E+00	7.05E-03
2	Potom.002G07963-SNP147	16	Potom.016G33891-SNP29	Lignin content	AA	2.43E+00	7.05E-03

2	Potom.002G05522-SNP73	20	Potom.003G09473-SNP11	Lignin content	AA	2.13E+00	7.04E-03
5	Potom.005G13333-SNP17	9	Potom.009G23017-SNP54	Fiber width	DA	-3.39E+00	7.04E-03
5	Potom.005G13985-SNP6	20	Potom.003G08321-SNP2	Fiber length	DA	2.19E-01	7.04E-03
8	Potom.008G22699-SNP3	8	Potom.008G22699-SNP72	Fiber width	AD	-2.85E+00	7.04E-03
1	Potom.001G03606-SNP26	2	Potom.002G07337-SNP15	Hemicellulose content	DD	1.69E+01	7.03E-03
2	Potom.002G05522-SNP62	5	Potom.005G13176-SNP155	Microfiber angle	AD	-5.12E+00	7.03E-03
10	Potom.010G25294-SNP212	13	Potom.013G29538-SNP72	Hemicellulose content	AD	-1.39E+01	7.02E-03
16	Potom.016G33891-SNP29	20	Potom.003G08321-SNP2	Holocellulose content	DD	-2.93E+01	7.02E-03
16	Potom.016G33891-SNP28	20	Potom.003G08321-SNP21	Holocellulose content	DD	2.29E+01	7.01E-03
5	Pto-Wuschela-SNP5	10	Potom.010G24967-SNP45	Fiber length	AD	-7.02E-02	6.99E-03
5	Potom.005G13176-SNP40	20	Potom.003G08321-SNP32	α-cellulose content	AD	1.18E+01	6.99E-03
2	Potom.002G05522-SNP23	12	Potom.012G28819-SNP45	Hemicellulose content	DD	1.52E+01	6.98E-03
2	Potom.002G05522-SNP62	5	Potom.005G13333-SNP13	Lignin content	DA	2.67E+00	6.98E-03
5	Potom.005G13176-SNP155	16	Potom.016G34352-SNP98	Microfiber angle	DA	-4.06E+00	6.98E-03
5	Potom.005G13333-SNP12	16	Potom.016G33876-SNP31	Stem volume	DA	2.85E-01	6.98E-03
10	Potom.010G25398-SNP38	13	Potom.013G29538-SNP117	α-cellulose content	AA	7.38E+00	6.98E-03
1	Potom.001G03606-SNP41	2	Potom.002G05769-SNP72	Stem volume	AA	-5.40E-01	6.97E-03
16	Potom.016G33891-SNP26	20	Potom.003G08321-SNP26	Microfiber angle	DA	-2.32E+00	6.97E-03
2	Potom.002G05522-SNP4	2	Potom.002G05522-SNP15	Holocellulose content	AD	-7.04E+00	6.96E-03
8	Potom.008G22699-SNP38	20	Potom.003G09473-SNP11	Fiber length	DD	-2.22E-01	6.96E-03
1	Potom.001G03606-SNP27	2	Potom.002G07963-SNP230	Fiber length	AA	1.38E-01	6.95E-03
8	Potom.008G21326-SNP69	16	Potom.016G33891-SNP26	Stem volume	AA	-3.07E-01	6.95E-03
10	Potom.010G24967-SNP40	20	Potom.003G09473-SNP13	Fiber length	DA	-1.22E-01	6.95E-03
5	Potom.005G13333-SNP12	16	Potom.016G33891-SNP17	Fiber width	DD	-4.76E+00	6.93E-03
5	Potom.005G13966-SNP90	10	Potom.010G25398-SNP68	Diameter at breast height	DD	1.79E+01	6.93E-03
16	Potom.016G33876-SNP31	16	Potom.016G34352-SNP45	Holocellulose content	AD	1.67E+01	6.93E-03

5	Potom.005G13176-SNP24	16	Potom.016G34352-SNP44	Holocellulose content	AD	-2.36E+01	6.92E-03
10	Potom.010G25398-SNP26	10	Potom.010G25706-SNP89	Stem volume	AA	2.43E-01	6.92E-03
12	Potom.012G28409-SNP23	20	Potom.003G08321-SNP3	Stem volume	AA	2.72E-01	6.92E-03
12	Potom.012G28819-SNP8	20	Potom.003G08321-SNP26	Hemicellulose content	AD	-1.43E+01	6.92E-03
1	Potom.001G03193-SNP20	5	Potom.005G13333-SNP12	Tree height	AA	-2.78E+00	6.91E-03
5	Potom.005G13176-SNP2	8	Potom.008G22699-SNP3	Diameter at breast height	AD	-7.66E+00	6.91E-03
5	Potom.005G13176-SNP26	8	Potom.008G22699-SNP74	Hemicellulose content	DA	1.43E+01	6.91E-03
8	Potom.008G22699-SNP98	16	Potom.016G33876-SNP55	Holocellulose content	DA	-1.58E+01	6.90E-03
5	Potom.005G13176-SNP41	5	Potom.005G13176-SNP193	Fiber length	AA	-2.15E-02	6.89E-03
5	Potom.005G13176-SNP41	8	Potom.008G22699-SNP108	Fiber length	AD	7.32E-02	6.87E-03
8	Potom.008G22699-SNP63	8	Potom.008G22699-SNP74	Microfiber angle	DD	1.33E+01	6.87E-03
1	Potom.001G03193-SNP30	10	Potom.010G25294-SNP212	Diameter at breast height	DD	-1.27E+01	6.85E-03
1	Potom.001G03606-SNP26	8	Potom.008G22699-SNP3	Diameter at breast height	AD	-7.35E+00	6.85E-03
2	Potom.002G07963-SNP152	10	Potom.010G25706-SNP79	Tree height	DA	-4.15E+00	6.85E-03
8	Potom.008G22699-SNP76	20	Potom.003G08321-SNP3	Fiber length	DA	1.34E-01	6.85E-03
1	Potom.001G03193-SNP20	20	Potom.003G09473-SNP2	Fiber length	DD	1.68E-01	6.84E-03
5	Potom.005G13176-SNP194	20	Potom.003G08321-SNP37	Diameter at breast height	AA	8.04E+00	6.84E-03
1	Potom.001G03193-SNP26	2	Potom.002G05522-SNP62	Stem volume	DA	-5.29E-01	6.83E-03
5	Potom.005G13176-SNP115	16	Potom.016G33876-SNP25	α-cellulose content	DA	1.96E+01	6.83E-03
8	Potom.008G22699-SNP108	16	Potom.016G34352-SNP108	Fiber length	DA	6.24E-02	6.83E-03
5	Potom.005G13176-SNP47	10	Potom.010G25294-SNP173	Hemicellulose content	AA	-4.59E+00	6.82E-03
8	Potom.008G22699-SNP3	8	Potom.008G22699-SNP72	Stem volume	AA	3.03E-01	6.82E-03
1	Potom.001G00363-SNP19	2	Potom.002G05522-SNP4	Holocellulose content	DA	-8.26E+00	6.81E-03
1	Potom.001G03606-SNP26	20	Potom.003G08321-SNP1	Tree height	DD	7.74E+00	6.81E-03
2	Potom.002G05522-SNP15	5	Potom.005G13985-SNP1	Fiber length	AD	-1.36E-01	6.81E-03
2	Potom.002G05522-SNP15	5	Potom.005G13985-SNP2	Fiber length	AD	-1.36E-01	6.81E-03

5	Potom.005G13176-SNP27	16	Potom.016G34352-SNP98	Holocellulose content	AA	1.01E+01	6.81E-03
2	Potom.002G07206-SNP24	13	Potom.013G29538-SNP2	Stem volume	AD	-4.31E-01	6.80E-03
20	Potom.003G08321-SNP13	20	Potom.003G08321-SNP20	Tree height	DD	-9.50E+00	6.80E-03
5	Potom.005G13333-SNP13	20	Potom.003G09473-SNP9	Diameter at breast height	AD	-1.47E+01	6.79E-03
5	Potom.005G13966-SNP77	16	Potom.016G33891-SNP6	Diameter at breast height	AA	-3.56E+00	6.79E-03
16	Potom.016G34352-SNP44	20	Potom.003G09473-SNP10	Holocellulose content	AA	1.61E+01	6.79E-03
5	Potom.005G13176-SNP47	20	Potom.003G08321-SNP7	Fiber width	AA	2.21E+00	6.78E-03
5	Potom.005G13985-SNP4	10	Potom.010G25294-SNP212	Fiber length	DA	2.21E-01	6.78E-03
20	Potom.003G08321-SNP20	20	Potom.003G09473-SNP13	Diameter at breast height	AA	4.35E+00	6.78E-03
2	Potom.002G07963-SNP147	20	Potom.003G08321-SNP9	Microfiber angle	DA	-6.54E+00	6.77E-03
5	Potom.005G13176-SNP62	10	Potom.010G24967-SNP43	Diameter at breast height	DA	-4.11E+00	6.77E-03
5	Potom.005G13176-SNP62	18	Potom.018G36220-SNP1	Fiber length	AA	9.44E-02	6.77E-03
2	Potom.002G07963-SNP153	5	Pto-Wuschela-SNP5	Hemicellulose content	AA	-8.68E+00	6.76E-03
5	Potom.005G13176-SNP4	20	Potom.003G09473-SNP12	α-cellulose content	DD	-2.85E+01	6.75E-03
5	Potom.005G13333-SNP12	8	Potom.008G22699-SNP3	Tree height	DD	3.65E+00	6.75E-03
5	Pto-Wuschela-SNP18	5	Potom.005G13966-SNP100	Lignin content	DD	6.12E+00	6.74E-03
2	Potom.002G05769-SNP43	8	Potom.008G22699-SNP108	Fiber length	AA	-6.71E-02	6.73E-03
20	Potom.003G08321-SNP10	20	Potom.003G08321-SNP26	Hemicellulose content	AD	1.20E+01	6.73E-03
1	Potom.001G00363-SNP23	13	Potom.013G29538-SNP9	α-cellulose content	DD	-3.03E+01	6.72E-03
5	Potom.005G13176-SNP2	20	Potom.003G09473-SNP13	Holocellulose content	DD	-2.21E+01	6.72E-03
5	Potom.005G13966-SNP74	12	Potom.012G28819-SNP45	α-cellulose content	AA	9.76E+00	6.72E-03
8	Potom.008G22699-SNP71	10	Potom.010G24967-SNP27	Fiber length	AD	1.66E-01	6.72E-03
8	Potom.008G22699-SNP71	10	Potom.010G25706-SNP89	Fiber width	DD	4.81E+00	6.72E-03
16	Potom.016G33891-SNP28	20	Potom.003G08321-SNP21	α-cellulose content	DD	2.11E+01	6.72E-03
1	Potom.001G00363-SNP19	8	Potom.008G22699-SNP71	Stem volume	AD	6.69E-01	6.71E-03
5	Potom.005G13333-SNP13	16	Potom.016G33891-SNP17	α-cellulose content	AD	2.04E+01	6.71E-03

10	Potom.010G25398-SNP68	13	Potom.013G29538-SNP9	α-cellulose content	DA	1.71E+01	6.71E-03
12	Potom.012G28409-SNP23	20	Potom.003G08321-SNP3	Holocellulose content	AD	7.96E+00	6.71E-03
2	Potom.002G05522-SNP15	5	Potom.005G13966-SNP90	Diameter at breast height	AD	1.02E+01	6.70E-03
2	Potom.002G05769-SNP57	8	Potom.008G21326-SNP20	Tree height	AD	4.51E+00	6.70E-03
1	Potom.001G03193-SNP10	2	Potom.002G07337-SNP34	α-cellulose content	DA	1.02E+01	6.69E-03
2	Potom.002G05522-SNP62	20	Potom.003G09473-SNP10	Hemicellulose content	AA	9.70E+00	6.69E-03
2	Potom.002G07337-SNP15	5	Potom.005G13176-SNP2	α-cellulose content	AD	1.54E+01	6.69E-03
5	Potom.005G13176-SNP162	8	Potom.008G22699-SNP22	Stem volume	DD	-7.38E-01	6.69E-03
2	Potom.002G07337-SNP144	5	Potom.005G13985-SNP1	Fiber length	AD	-8.35E-02	6.68E-03
2	Potom.002G07337-SNP144	5	Potom.005G13985-SNP2	Fiber length	AD	-8.35E-02	6.68E-03
2	Potom.002G07337-SNP145	5	Potom.005G13985-SNP1	Fiber length	AD	-8.35E-02	6.68E-03
2	Potom.002G07337-SNP145	5	Potom.005G13985-SNP2	Fiber length	AD	-8.35E-02	6.68E-03
20	Potom.003G08321-SNP20	20	Potom.003G09473-SNP13	Stem volume	AA	2.93E-01	6.68E-03
1	Potom.001G03193-SNP26	20	Potom.003G09473-SNP2	Diameter at breast height	AD	7.68E+00	6.67E-03
13	Potom.013G29538-SNP9	20	Potom.003G08321-SNP14	Holocellulose content	AA	1.54E+01	6.67E-03
8	Potom.008G21952-SNP16	13	Potom.013G29538-SNP72	Diameter at breast height	AA	-5.80E+00	6.66E-03
8	Potom.008G22516-SNP48	10	Potom.010G25700-SNP19	Microfiber angle	AD	7.90E+00	6.66E-03
8	Potom.008G22699-SNP74	10	Potom.010G24967-SNP45	Diameter at breast height	AD	-4.83E+00	6.66E-03
1	Potom.001G00363-SNP19	2	Potom.002G05522-SNP23	Diameter at breast height	AA	-4.50E+00	6.65E-03
5	Potom.005G13176-SNP3	20	Potom.003G09473-SNP12	α-cellulose content	DD	-2.93E+01	6.65E-03
10	Potom.010G24967-SNP45	20	Potom.003G09473-SNP2	Stem volume	AD	4.62E-01	6.65E-03
10	Potom.010G25398-SNP26	18	Potom.018G36220-SNP43	Diameter at breast height	DA	-5.57E+00	6.63E-03
5	Pto-Wuschela-SNP14	10	Potom.010G25700-SNP5	Hemicellulose content	AA	5.82E+00	6.62E-03
5	Potom.005G13176-SNP44	8	Potom.008G22699-SNP108	Microfiber angle	DD	5.79E+00	6.62E-03
5	Potom.005G13966-SNP77	16	Potom.016G33891-SNP28	Stem volume	AA	-2.89E-01	6.62E-03
9	Potom.009G22902-SNP9	20	Potom.003G08321-SNP6	Diameter at breast height	AA	6.46E+00	6.61E-03

1	Potom.001G03606-SNP27	5	Potom.005G13333-SNP17	α-cellulose content	DD	2.19E+01	6.59E-03
5	Potom.005G13176-SNP26	20	Potom.003G08321-SNP32	Diameter at breast height	AD	-9.15E+00	6.59E-03
1	Potom.001G03193-SNP30	14	Potom.014G31981-SNP6	Diameter at breast height	AD	-1.03E+01	6.58E-03
2	Potom.002G07963-SNP72	20	Potom.003G08321-SNP6	Fiber length	AA	4.14E-02	6.58E-03
1	Potom.001G03193-SNP20	20	Potom.003G09473-SNP2	α-cellulose content	AD	-9.05E+00	6.57E-03
10	Potom.010G25294-SNP173	20	Potom.003G10089-SNP11	Hemicellulose content	AD	-4.24E+00	6.57E-03
10	Potom.010G25398-SNP26	10	Potom.010G25398-SNP63	Hemicellulose content	AD	-1.79E+01	6.57E-03
13	Potom.013G29538-SNP16	20	Potom.003G08321-SNP21	Hemicellulose content	DD	1.54E+01	6.57E-03
5	Potom.005G13966-SNP90	8	Potom.008G22699-SNP72	Hemicellulose content	AA	9.07E+00	6.54E-03
5	Potom.005G13985-SNP4	16	Potom.016G33876-SNP31	Fiber length	DA	2.09E-01	6.54E-03
2	Potom.002G05522-SNP4	2	Potom.002G05522-SNP15	Lignin content	DD	4.22E+00	6.52E-03
2	Potom.002G07963-SNP147	16	Potom.016G33891-SNP27	Fiber length	DA	1.44E-01	6.52E-03
2	Potom.002G05522-SNP60	20	Potom.003G08321-SNP14	Holocellulose content	AA	1.41E+01	6.51E-03
5	Potom.005G13985-SNP20	16	Potom.016G33876-SNP94	Holocellulose content	DA	1.20E+01	6.51E-03
1	Potom.001G00363-SNP23	5	Potom.005G13176-SNP115	Diameter at breast height	DA	9.02E+00	6.50E-03
2	Potom.002G05769-SNP13	5	Potom.005G13176-SNP27	Hemicellulose content	DA	1.72E+01	6.49E-03
13	Potom.013G29538-SNP6	20	Potom.003G08321-SNP17	α-cellulose content	AA	7.70E+00	6.49E-03
13	Potom.013G29538-SNP8	20	Potom.003G08321-SNP18	Fiber width	DA	2.21E+00	6.49E-03
1	Potom.001G03606-SNP26	8	Potom.008G22699-SNP108	Fiber length	AD	1.24E-01	6.48E-03
2	Potom.002G05769-SNP13	5	Potom.005G13966-SNP97	Holocellulose content	AA	1.24E+01	6.48E-03
2	Potom.002G05522-SNP13	20	Potom.003G08321-SNP1	Stem volume	AA	-6.27E-01	6.47E-03
2	Potom.002G05522-SNP14	20	Potom.003G08321-SNP1	Stem volume	AA	-6.27E-01	6.47E-03
2	Potom.002G05632-SNP14	5	Potom.005G13176-SNP62	Stem volume	AA	-2.52E-01	6.47E-03
5	Pto-Wuschela-SNP5	20	Potom.003G08321-SNP17	Holocellulose content	AA	4.52E+00	6.47E-03
5	Pto-Wuschela-SNP12	18	Potom.018G36220-SNP1	Hemicellulose content	DA	-1.08E+01	6.47E-03
10	Potom.010G25398-SNP26	18	Potom.018G36220-SNP42	Diameter at breast height	DA	-5.65E+00	6.47E-03

1	Potom.001G03193-SNP20	16	Potom.016G33876-SNP74	Diameter at breast height	AD	-8.41E+00	6.46E-03
5	Potom.005G13176-SNP16	20	Potom.003G08321-SNP32	α-cellulose content	AD	1.23E+01	6.46E-03
5	Potom.005G13985-SNP1	20	Potom.003G09473-SNP2	Microfiber angle	DD	-9.82E+00	6.46E-03
5	Potom.005G13985-SNP2	20	Potom.003G09473-SNP2	Microfiber angle	DD	-9.82E+00	6.46E-03
2	Potom.002G05522-SNP12	5	Potom.005G13966-SNP90	Tree height	DD	-7.04E+00	6.45E-03
2	Potom.002G05522-SNP23	8	Potom.008G22699-SNP3	Diameter at breast height	DA	5.75E+00	6.45E-03
2	Potom.002G05522-SNP62	10	Potom.010G24967-SNP45	Fiber length	DA	-7.22E-02	6.45E-03
2	Potom.002G05769-SNP72	12	Potom.012G28819-SNP8	Microfiber angle	AA	5.17E+00	6.45E-03
2	Potom.002G07337-SNP144	5	Potom.005G13985-SNP5	Fiber length	AA	-4.75E-02	6.45E-03
2	Potom.002G07337-SNP145	5	Potom.005G13985-SNP5	Fiber length	AA	-4.75E-02	6.45E-03
8	Potom.008G21326-SNP19	16	Potom.016G33891-SNP26	Stem volume	AA	-4.63E-01	6.44E-03
20	Potom.003G08321-SNP10	20	Potom.003G08321-SNP20	Stem volume	DD	-8.42E-01	6.43E-03
1	Potom.001G03193-SNP10	20	Potom.003G10089-SNP11	α-cellulose content	DA	1.51E+01	6.42E-03
2	Potom.002G05522-SNP1	20	Potom.003G09473-SNP11	Holocellulose content	DA	-2.04E+01	6.41E-03
1	Potom.001G03353-SNP25	5	Pto-Wuschela-SNP12	Hemicellulose content	DD	-1.70E+01	6.40E-03
2	Potom.002G05522-SNP15	16	Potom.016G33876-SNP55	Holocellulose content	AA	3.49E+00	6.40E-03
2	Potom.002G07337-SNP15	20	Potom.003G09473-SNP2	Tree height	DD	4.46E+00	6.39E-03
5	Potom.005G13966-SNP90	8	Potom.008G22699-SNP9	Stem volume	DD	-1.21E+00	6.39E-03
16	Potom.016G33876-SNP31	16	Potom.016G34352-SNP109	Holocellulose content	AA	-5.60E+00	6.39E-03
16	Potom.016G33891-SNP17	20	Potom.003G08321-SNP26	α-cellulose content	AD	1.43E+01	6.39E-03
8	Potom.008G22699-SNP71	10	Potom.010G25706-SNP89	Stem volume	DA	-5.05E-01	6.38E-03
20	Potom.003G08321-SNP7	20	Potom.003G08321-SNP17	Tree height	DA	-2.34E+00	6.38E-03
2	Potom.002G05522-SNP12	5	Potom.005G13985-SNP1	Hemicellulose content	DD	-1.70E+01	6.37E-03
2	Potom.002G05522-SNP12	5	Potom.005G13985-SNP2	Hemicellulose content	DD	-1.70E+01	6.37E-03
13	Potom.013G29538-SNP5	20	Potom.003G08321-SNP14	Tree height	DD	-9.06E+00	6.37E-03
8	Potom.008G21952-SNP16	10	Potom.010G25700-SNP5	α-cellulose content	DD	2.82E+01	6.36E-03

10	Potom.010G25294-SNP212	20	Potom.003G10089-SNP11	α-cellulose content	AD	1.38E+01	6.36E-03
10	Potom.010G25398-SNP63	16	Potom.016G33891-SNP29	α-cellulose content	DA	-1.99E+01	6.36E-03
1	Potom.001G00363-SNP53	2	Potom.002G07337-SNP15	Hemicellulose content	AD	1.35E+01	6.35E-03
1	Potom.001G03193-SNP20	16	Potom.016G33876-SNP73	Lignin content	DA	-2.04E+00	6.35E-03
1	Potom.001G03353-SNP25	10	Potom.010G24967-SNP43	Diameter at breast height	DA	-5.07E+00	6.35E-03
10	Potom.010G25706-SNP46	20	Potom.003G09473-SNP9	Diameter at breast height	AD	1.22E+01	6.35E-03
16	Potom.016G33876-SNP74	18	Potom.018G36220-SNP42	Microfiber angle	AA	2.50E+00	6.35E-03
16	Potom.016G33891-SNP29	20	Potom.003G08321-SNP17	Holocellulose content	DD	-1.86E+01	6.35E-03
5	Potom.005G13985-SNP17	10	Potom.010G24967-SNP58	Fiber length	DD	-3.01E-01	6.34E-03
1	Potom.001G03193-SNP26	2	Potom.002G05522-SNP60	Stem volume	DA	-5.34E-01	6.33E-03
5	Potom.005G13333-SNP12	16	Potom.016G33891-SNP27	Fiber length	DD	-2.12E-01	6.33E-03
1	Potom.001G00363-SNP21	13	Potom.013G29538-SNP9	α-cellulose content	DD	-3.03E+01	6.32E-03
1	Potom.001G01788-SNP70	2	Potom.002G07337-SNP1	Diameter at breast height	DD	-9.67E+00	6.32E-03
5	Potom.005G13966-SNP90	8	Potom.008G22699-SNP3	Stem volume	DD	-9.54E-01	6.32E-03
2	Potom.002G05522-SNP54	8	Potom.008G21952-SNP16	Diameter at breast height	AA	-6.33E+00	6.30E-03
1	Potom.001G00363-SNP26	20	Potom.003G08321-SNP14	α-cellulose content	AD	-2.16E+01	6.29E-03
10	Potom.010G25294-SNP173	20	Potom.003G10089-SNP11	Fiber width	AD	-1.02E+00	6.29E-03
1	Potom.001G00363-SNP19	5	Pto-Wuschela-SNP14	α-cellulose content	AD	-1.27E+01	6.28E-03
8	Potom.008G22699-SNP108	16	Potom.016G33891-SNP29	Fiber length	DA	1.13E-01	6.28E-03
18	Potom.018G36220-SNP10	20	Potom.003G08321-SNP5	Lignin content	AA	-1.20E+00	6.28E-03
1	Potom.001G03606-SNP29	10	Potom.010G25398-SNP68	Tree height	DD	-9.73E+00	6.27E-03
5	Potom.005G13966-SNP97	16	Potom.016G33891-SNP14	Fiber width	AD	5.55E+00	6.27E-03
1	Potom.001G03193-SNP20	16	Potom.016G33876-SNP71	Diameter at breast height	AD	-8.29E+00	6.26E-03
1	Potom.001G03353-SNP25	14	Potom.014G31981-SNP25	Fiber width	AD	2.99E+00	6.26E-03
2	Potom.002G05769-SNP57	8	Potom.008G22699-SNP108	Fiber length	AA	-9.30E-02	6.25E-03
13	Potom.013G29538-SNP71	16	Potom.016G34352-SNP108	Hemicellulose content	DA	-1.28E+01	6.25E-03

2	Potom.002G05632-SNP15	5	Potom.005G13176-SNP62	Diameter at breast height	AA	-2.82E+00	6.24E-03
14	Potom.014G31981-SNP26	20	Potom.003G08321-SNP17	Fiber length	AD	1.04E-01	6.24E-03
2	Potom.002G05522-SNP1	20	Potom.003G09473-SNP12	Hemicellulose content	DD	2.87E+01	6.23E-03
5	Potom.005G13176-SNP47	16	Potom.016G33891-SNP13	Fiber length	AD	1.06E-01	6.23E-03
1	Potom.001G00363-SNP19	5	Potom.005G13176-SNP194	Diameter at breast height	DA	5.68E+00	6.22E-03
5	Potom.005G13176-SNP2	8	Potom.008G22699-SNP71	Holocellulose content	AA	1.29E+01	6.22E-03
2	Potom.002G07206-SNP24	20	Potom.003G08321-SNP17	Tree height	AA	1.46E+00	6.21E-03
2	Potom.002G05522-SNP54	8	Potom.008G21952-SNP16	Stem volume	AD	4.70E-01	6.20E-03
2	Potom.002G05769-SNP13	20	Potom.003G08321-SNP1	Hemicellulose content	DA	2.09E+01	6.20E-03
8	Potom.008G21326-SNP10	16	Potom.016G33891-SNP26	Stem volume	AA	-4.80E-01	6.20E-03
2	Potom.002G05522-SNP73	10	Potom.010G24967-SNP45	Hemicellulose content	AA	-6.60E+00	6.19E-03
8	Potom.008G21952-SNP16	20	Potom.003G09473-SNP13	Stem volume	AA	-5.11E-01	6.19E-03
10	Potom.010G24967-SNP45	20	Potom.003G08321-SNP10	Lignin content	DD	-4.22E+00	6.19E-03
12	Potom.012G28819-SNP9	18	Potom.018G36220-SNP10	Diameter at breast height	AA	-2.70E+00	6.19E-03
1	Potom.001G00363-SNP54	5	Pto-Wuschela-SNP14	Holocellulose content	AD	-1.53E+01	6.18E-03
1	Potom.001G00363-SNP55	5	Pto-Wuschela-SNP14	Holocellulose content	AD	-1.53E+01	6.18E-03
5	Potom.005G13176-SNP44	10	Potom.010G24967-SNP43	Stem volume	AA	-2.54E-01	6.17E-03
8	Potom.008G22699-SNP108	8	Potom.008G22699-SNP153	Tree height	DA	2.59E+00	6.17E-03
13	Potom.013G29538-SNP6	20	Potom.003G08321-SNP18	Fiber width	DA	2.21E+00	6.17E-03
5	Potom.005G13176-SNP44	16	Potom.016G34352-SNP98	Holocellulose content	AA	7.07E+00	6.15E-03
1	Potom.001G03606-SNP26	8	Potom.008G21952-SNP16	Holocellulose content	DA	1.67E+01	6.14E-03
10	Potom.010G25706-SNP45	20	Potom.003G09473-SNP9	Diameter at breast height	AD	1.22E+01	6.14E-03
2	Potom.002G05522-SNP54	20	Potom.003G09473-SNP2	Fiber length	DA	1.17E-01	6.12E-03
2	Potom.002G07963-SNP153	10	Potom.010G25706-SNP79	Tree height	DA	-4.33E+00	6.10E-03
5	Pto-Wuschela-SNP5	10	Potom.010G24967-SNP43	Fiber length	AD	-7.18E-02	6.10E-03
8	Potom.008G21326-SNP10	16	Potom.016G33891-SNP26	Diameter at breast height	AA	-6.85E+00	6.09E-03

10	Potom.010G25398-SNP26	20	Potom.003G09473-SNP2	Fiber length	AD	1.06E-01	6.09E-03
8	Potom.008G22699-SNP74	20	Potom.003G10089-SNP11	Diameter at breast height	DA	7.19E+00	6.08E-03
5	Potom.005G13176-SNP25	16	Potom.016G34352-SNP44	Holocellulose content	AD	-2.38E+01	6.07E-03
10	Potom.010G25706-SNP89	13	Potom.013G29538-SNP7	Holocellulose content	DD	1.66E+01	6.07E-03
13	Potom.013G29538-SNP9	20	Potom.003G08321-SNP18	Hemicellulose content	DD	1.93E+01	6.07E-03
5	Potom.005G13176-SNP193	10	Potom.010G24967-SNP27	Diameter at breast height	AD	-4.72E+00	6.06E-03
1	Potom.001G03606-SNP29	20	Potom.003G08321-SNP14	Stem volume	DA	9.61E-01	6.05E-03
2	Potom.002G05522-SNP62	13	Potom.013G29538-SNP2	Stem volume	AA	2.30E-01	6.05E-03
5	Pto-Wuschela-SNP5	5	Potom.005G13176-SNP41	Hemicellulose content	DD	-1.16E+01	6.05E-03
5	Potom.005G13176-SNP47	5	Potom.005G13333-SNP12	Hemicellulose content	AD	-6.28E+00	6.05E-03
5	Potom.005G13176-SNP44	10	Potom.010G24967-SNP45	Stem volume	AA	-2.67E-01	6.04E-03
2	Potom.002G07963-SNP71	5	Potom.005G13176-SNP157	Fiber length	AA	-5.72E-02	6.03E-03
10	Potom.010G25398-SNP26	20	Potom.003G09473-SNP12	Stem volume	DA	-7.02E-01	6.03E-03
1	Potom.001G03606-SNP26	5	Potom.005G13333-SNP12	Lignin content	DD	5.70E+00	6.02E-03
2	Potom.002G05522-SNP54	20	Potom.003G08321-SNP17	Stem volume	AA	-3.33E-01	6.02E-03
5	Potom.005G13176-SNP2	20	Potom.003G09473-SNP11	Holocellulose content	DD	-2.21E+01	6.02E-03
5	Potom.005G13176-SNP27	20	Potom.003G09473-SNP13	Fiber length	AA	1.13E-01	6.02E-03
5	Potom.005G13333-SNP13	13	Potom.013G29538-SNP4	Stem volume	AA	-2.38E-01	6.02E-03
2	Potom.002G05522-SNP15	2	Potom.002G05522-SNP73	Holocellulose content	AD	-1.08E+01	6.01E-03
2	Potom.002G05522-SNP60	13	Potom.013G29538-SNP4	Lignin content	DD	-3.91E+00	6.01E-03
8	Potom.008G22699-SNP108	16	Potom.016G33891-SNP6	α-cellulose content	AD	9.95E+00	6.01E-03
1	Potom.001G03353-SNP24	5	Potom.005G13985-SNP1	Fiber length	AA	1.20E-01	6.00E-03
1	Potom.001G03353-SNP24	5	Potom.005G13985-SNP2	Fiber length	AA	1.20E-01	6.00E-03
8	Potom.008G21952-SNP16	10	Potom.010G25700-SNP13	Stem volume	AA	-7.04E-01	6.00E-03
10	Potom.010G25398-SNP68	13	Potom.013G29538-SNP5	Tree height	DA	-6.07E+00	6.00E-03
13	Potom.013G29538-SNP4	20	Potom.003G09473-SNP2	α-cellulose content	AA	-4.49E+00	6.00E-03

18	Potom.018G36220-SNP37	20	Potom.003G09473-SNP11	Fiber length	AD	8.29E-02	6.00E-03
1	Potom.001G00363-SNP19	5	Pto-Wuschela-SNP14	Lignin content	AD	3.64E+00	5.99E-03
20	Potom.003G08321-SNP2	20	Potom.003G08321-SNP26	Diameter at breast height	DA	8.21E+00	5.99E-03
1	Potom.001G03353-SNP25	14	Potom.014G31981-SNP26	Diameter at breast height	DA	-8.01E+00	5.98E-03
2	Potom.002G05522-SNP54	13	Potom.013G29538-SNP5	Stem volume	AA	3.50E-01	5.97E-03
8	Potom.008G21326-SNP19	16	Potom.016G33891-SNP26	Diameter at breast height	AA	-6.64E+00	5.97E-03
10	Potom.010G25398-SNP68	20	Potom.003G08321-SNP4	Diameter at breast height	DD	-1.98E+01	5.97E-03
10	Potom.010G25706-SNP89	13	Potom.013G29538-SNP9	Holocellulose content	DD	1.68E+01	5.97E-03
2	Potom.002G07337-SNP15	2	Potom.002G07963-SNP153	Diameter at breast height	DA	-9.42E+00	5.96E-03
5	Potom.005G13176-SNP27	18	Potom.018G35976-SNP13	Diameter at breast height	AD	-9.33E+00	5.94E-03
5	Potom.005G13966-SNP97	10	Potom.010G25398-SNP63	Hemicellulose content	DA	-1.55E+01	5.94E-03
14	Potom.014G31981-SNP25	20	Potom.003G09473-SNP11	Stem volume	AA	-4.96E-01	5.94E-03
2	Potom.002G05522-SNP62	20	Potom.003G08321-SNP14	Holocellulose content	AA	1.43E+01	5.93E-03
20	Potom.003G08321-SNP20	20	Potom.003G09473-SNP11	Diameter at breast height	AA	4.39E+00	5.93E-03
5	Potom.005G13176-SNP44	13	Potom.013G29538-SNP71	Fiber width	AD	-3.13E+00	5.92E-03
8	Potom.008G22699-SNP100	20	Potom.003G08321-SNP33	Microfiber angle	AD	3.43E+00	5.92E-03
1	Potom.001G00363-SNP26	5	Potom.005G13176-SNP3	Stem volume	AD	7.86E-01	5.91E-03
1	Potom.001G03193-SNP10	5	Potom.005G13176-SNP117	α-cellulose content	AD	-5.40E+00	5.91E-03
2	Potom.002G05522-SNP4	20	Potom.003G09473-SNP25	Hemicellulose content	DA	-1.21E+01	5.91E-03
20	Potom.003G08321-SNP11	20	Potom.003G09473-SNP2	Microfiber angle	DD	-8.82E+00	5.91E-03
5	Potom.005G13176-SNP27	20	Potom.003G09473-SNP2	Fiber length	AD	1.36E-01	5.90E-03
8	Potom.008G22699-SNP3	20	Potom.003G08321-SNP4	Stem volume	DD	1.24E+00	5.90E-03
2	Potom.002G05522-SNP23	8	Potom.008G22699-SNP3	Stem volume	DA	4.20E-01	5.89E-03
5	Pto-Wuschela-SNP5	12	Potom.012G28819-SNP45	Hemicellulose content	AA	-5.87E+00	5.88E-03
5	Potom.005G13985-SNP20	20	Potom.003G08321-SNP38	Stem volume	AA	-5.99E-01	5.87E-03
5	Potom.005G13985-SNP5	8	Potom.008G22699-SNP108	Tree height	DD	6.85E+00	5.86E-03

8	Potom.008G22699-SNP22	20	Potom.003G08321-SNP9	Lignin content	DD	-5.93E+00	5.86E-03
2	Potom.002G07337-SNP1	13	Potom.013G29538-SNP71	Fiber width	AD	-2.90E+00	5.85E-03
8	Potom.008G22699-SNP71	10	Potom.010G25706-SNP89	Stem volume	DD	-8.06E-01	5.85E-03
8	Potom.008G22699-SNP108	18	Potom.018G36220-SNP43	Tree height	DA	2.12E+00	5.85E-03
10	Potom.010G25706-SNP89	13	Potom.013G29538-SNP7	α-cellulose content	DA	-1.11E+01	5.84E-03
8	Potom.008G22699-SNP38	16	Potom.016G33891-SNP28	Hemicellulose content	AD	1.75E+01	5.82E-03
5	Pto-Wuschela-SNP5	10	Potom.010G24967-SNP53	Fiber length	AD	-9.17E-02	5.81E-03
10	Potom.010G24967-SNP53	20	Potom.003G09473-SNP13	Hemicellulose content	AA	-1.22E+01	5.81E-03
12	Potom.012G28819-SNP27	20	Potom.003G09473-SNP9	Fiber length	DA	-1.79E-01	5.81E-03
10	Potom.010G25398-SNP26	20	Potom.003G09473-SNP10	Stem volume	DA	-7.03E-01	5.80E-03
13	Potom.013G29538-SNP4	20	Potom.003G08321-SNP10	Diameter at breast height	AD	7.17E+00	5.80E-03
1	Potom.001G03606-SNP27	5	Potom.005G13176-SNP27	Lignin content	DD	-6.70E+00	5.79E-03
5	Potom.005G13985-SNP17	8	Potom.008G22699-SNP98	Diameter at breast height	AA	-9.60E+00	5.79E-03
8	Potom.008G21952-SNP16	13	Potom.013G29538-SNP72	Stem volume	AD	-5.48E-01	5.79E-03
10	Potom.010G25398-SNP68	13	Potom.013G29538-SNP72	Holocellulose content	AD	2.12E+01	5.79E-03
13	Potom.013G29538-SNP15	20	Potom.003G08321-SNP21	Hemicellulose content	DD	1.58E+01	5.79E-03
10	Potom.010G25398-SNP68	20	Potom.003G08321-SNP4	Tree height	DD	-9.91E+00	5.78E-03
5	Pto-Wuschela-SNP5	5	Potom.005G13176-SNP162	Holocellulose content	AA	5.07E+00	5.77E-03
5	Potom.005G13176-SNP41	8	Potom.008G21326-SNP20	Tree height	DA	-3.53E+00	5.77E-03
5	Potom.005G13333-SNP13	10	Potom.010G25706-SNP78	Holocellulose content	AA	-8.77E+00	5.77E-03
8	Potom.008G21952-SNP16	13	Potom.013G29538-SNP5	α-cellulose content	DD	-1.84E+01	5.77E-03
8	Potom.008G22699-SNP69	16	Potom.016G33891-SNP28	Hemicellulose content	AD	1.47E+01	5.77E-03
13	Potom.013G29538-SNP16	16	Potom.016G33891-SNP17	Hemicellulose content	AA	-6.73E+00	5.77E-03
1	Potom.001G03606-SNP27	5	Potom.005G13966-SNP90	Holocellulose content	AA	1.43E+01	5.76E-03
2	Potom.002G05769-SNP13	5	Potom.005G13966-SNP90	Stem volume	AD	-9.41E-01	5.76E-03
16	Potom.016G33876-SNP31	18	Potom.018G36220-SNP81	Holocellulose content	AA	1.02E+01	5.76E-03

8	Potom.008G21952-SNP16	8	Potom.008G22699-SNP69	Fiber length	AA	1.03E-01	5.75E-03
13	Potom.013G29538-SNP15	20	Potom.003G08321-SNP9	Microfiber angle	AA	-3.71E+00	5.75E-03
13	Potom.013G29538-SNP16	20	Potom.003G08321-SNP9	Microfiber angle	AA	-3.70E+00	5.75E-03
2	Potom.002G05522-SNP60	10	Potom.010G24967-SNP45	Fiber length	DA	-7.33E-02	5.74E-03
2	Potom.002G07337-SNP229	5	Potom.005G13176-SNP162	Stem volume	AA	-2.62E-01	5.74E-03
14	Potom.014G31981-SNP26	20	Potom.003G08321-SNP11	Diameter at breast height	AD	-8.87E+00	5.74E-03
16	Potom.016G33891-SNP15	20	Potom.003G08321-SNP26	Diameter at breast height	AD	-8.27E+00	5.74E-03
2	Potom.002G05522-SNP54	2	Potom.002G07963-SNP163	Fiber length	AD	-1.21E-01	5.72E-03
1	Potom.001G00363-SNP21	8	Potom.008G22699-SNP108	Lignin content	DA	-4.70E+00	5.71E-03
1	Potom.001G03353-SNP164	18	Potom.018G36220-SNP10	Microfiber angle	DD	7.07E+00	5.71E-03
5	Pto-Wuschela-SNP12	20	Potom.003G10089-SNP11	Holocellulose content	DA	-1.18E+01	5.71E-03
10	Potom.010G25398-SNP63	16	Potom.016G33891-SNP29	Diameter at breast height	DA	1.21E+01	5.70E-03
1	Potom.001G03606-SNP26	10	Potom.010G25398-SNP68	Fiber width	DD	5.97E+00	5.69E-03
5	Potom.005G13333-SNP18	18	Potom.018G36220-SNP1	α-cellulose content	DD	1.36E+01	5.69E-03
8	Potom.008G22699-SNP108	16	Potom.016G33891-SNP15	α-cellulose content	AD	8.98E+00	5.69E-03
5	Potom.005G13176-SNP44	16	Potom.016G33876-SNP73	Microfiber angle	AA	2.14E+00	5.68E-03
8	Potom.008G21952-SNP16	13	Potom.013G29538-SNP4	α-cellulose content	AD	1.23E+01	5.68E-03
5	Potom.005G13176-SNP27	5	Potom.005G13966-SNP97	Holocellulose content	AA	1.04E+01	5.67E-03
5	Potom.005G13176-SNP27	8	Potom.008G22699-SNP2	Diameter at breast height	AA	-8.73E+00	5.67E-03
5	Potom.005G13966-SNP89	16	Potom.016G34352-SNP109	Diameter at breast height	DD	1.75E+01	5.67E-03
1	Potom.001G03353-SNP25	2	Potom.002G05522-SNP23	Diameter at breast height	AA	4.22E+00	5.66E-03
2	Potom.002G05522-SNP23	5	Potom.005G13333-SNP12	Stem volume	AD	5.29E-01	5.66E-03
2	Potom.002G05769-SNP43	13	Potom.013G29538-SNP71	Hemicellulose content	AD	-1.48E+01	5.64E-03
5	Pto-Wuschela-SNP5	5	Potom.005G13176-SNP40	Hemicellulose content	DD	-1.29E+01	5.64E-03
2	Potom.002G05769-SNP70	20	Potom.003G08321-SNP17	Holocellulose content	AD	1.10E+01	5.63E-03
2	Potom.002G05522-SNP15	10	Potom.010G25398-SNP26	Diameter at breast height	DD	-1.29E+01	5.62E-03

8	Potom.008G22699-SNP74	13	Potom.013G29538-SNP117	α-cellulose content	AA	-4.44E+00	5.62E-03
2	Potom.002G07337-SNP34	8	Potom.008G22699-SNP70	Diameter at breast height	DA	-8.37E+00	5.61E-03
5	Pto-Wuschela-SNP14	9	Potom.009G23029-SNP3	Holocellulose content	AA	7.73E+00	5.61E-03
13	Potom.013G29538-SNP4	20	Potom.003G08321-SNP10	Fiber length	AD	1.04E-01	5.61E-03
1	Potom.001G03193-SNP13	1	Potom.001G03353-SNP25	Stem volume	DD	1.15E+00	5.60E-03
1	Potom.001G03193-SNP20	5	Potom.005G13176-SNP2	Holocellulose content	AA	-1.15E+01	5.60E-03
5	Potom.005G13333-SNP17	13	Potom.013G29538-SNP15	Tree height	DD	-4.53E+00	5.60E-03
2	Potom.002G07337-SNP229	10	Potom.010G25398-SNP63	α-cellulose content	AD	-1.95E+01	5.59E-03
5	Potom.005G13966-SNP74	10	Potom.010G24967-SNP43	Fiber length	AA	-6.00E-02	5.59E-03
16	Potom.016G34352-SNP108	20	Potom.003G09473-SNP2	Diameter at breast height	AD	6.35E+00	5.59E-03
5	Potom.005G13966-SNP89	16	Potom.016G33876-SNP94	Holocellulose content	AD	1.51E+01	5.58E-03
16	Potom.016G34352-SNP108	20	Potom.003G08321-SNP20	Fiber length	AA	5.35E-02	5.56E-03
5	Potom.005G13176-SNP26	20	Potom.003G08321-SNP32	α-cellulose content	DD	1.86E+01	5.55E-03
5	Potom.005G13333-SNP12	18	Potom.018G36220-SNP1	Fiber width	AD	2.23E+00	5.55E-03
16	Potom.016G33876-SNP25	20	Potom.003G08321-SNP17	Stem volume	AA	-5.90E-01	5.55E-03
1	Potom.001G01788-SNP70	2	Potom.002G05522-SNP12	Fiber length	DA	-1.02E-01	5.54E-03
2	Potom.002G05522-SNP23	20	Potom.003G08321-SNP10	Stem volume	DD	-6.62E-01	5.54E-03
9	Potom.009G22902-SNP9	9	Potom.009G23017-SNP55	α-cellulose content	DD	2.52E+01	5.54E-03
5	Pto-Wuschela-SNP5	8	Potom.008G22699-SNP72	Hemicellulose content	AA	7.44E+00	5.52E-03
2	Potom.002G05522-SNP52	5	Potom.005G13176-SNP62	Hemicellulose content	AA	2.38E+00	5.51E-03
2	Potom.002G05522-SNP4	5	Potom.005G13966-SNP74	Microfiber angle	AD	3.68E+00	5.50E-03
2	Potom.002G05522-SNP13	20	Potom.003G08321-SNP1	Diameter at breast height	AD	1.37E+01	5.50E-03
2	Potom.002G05522-SNP14	20	Potom.003G08321-SNP1	Diameter at breast height	AD	1.37E+01	5.50E-03
2	Potom.002G05522-SNP62	10	Potom.010G24967-SNP67	Stem volume	DA	-3.14E-01	5.50E-03
2	Potom.002G07963-SNP72	16	Potom.016G33891-SNP15	Fiber length	AA	-4.29E-02	5.50E-03
5	Potom.005G13176-SNP27	20	Potom.003G09473-SNP11	Fiber length	AA	1.11E-01	5.50E-03

5	Potom.005G13966-SNP74	12	Potom.012G28409-SNP23	Holocellulose content	AA	7.48E+00	5.50E-03
1	Potom.001G03193-SNP20	16	Potom.016G33876-SNP74	Lignin content	DA	-2.08E+00	5.48E-03
5	Potom.005G13176-SNP27	8	Potom.008G22699-SNP3	Hemicellulose content	DD	1.72E+01	5.48E-03
5	Potom.005G13176-SNP39	8	Potom.008G22699-SNP74	Stem volume	AA	2.25E-01	5.46E-03
5	Potom.005G13176-SNP41	8	Potom.008G21326-SNP66	Tree height	DA	-3.03E+00	5.46E-03
2	Potom.002G07963-SNP229	20	Potom.003G10089-SNP7	Fiber width	AD	3.84E+00	5.45E-03
5	Potom.005G13966-SNP77	13	Potom.013G29538-SNP72	Stem volume	AA	-1.86E-01	5.45E-03
5	Potom.005G13985-SNP20	8	Potom.008G22699-SNP72	Stem volume	AA	-5.04E-01	5.45E-03
2	Potom.002G05769-SNP70	5	Potom.005G13333-SNP12	Fiber length	AD	1.19E-01	5.44E-03
1	Potom.001G01788-SNP70	16	Potom.016G34352-SNP58	Fiber length	AA	9.54E-02	5.43E-03
2	Potom.002G05522-SNP23	14	Potom.014G31981-SNP25	Holocellulose content	DA	1.23E+01	5.43E-03
5	Pto-Wuschela-SNP5	10	Potom.010G24967-SNP27	Holocellulose content	AA	-6.23E+00	5.43E-03
2	Potom.002G05522-SNP13	13	Potom.013G29538-SNP5	Fiber length	DD	-1.89E-01	5.42E-03
2	Potom.002G05522-SNP14	13	Potom.013G29538-SNP5	Fiber length	DD	-1.89E-01	5.42E-03
2	Potom.002G05522-SNP54	20	Potom.003G08321-SNP18	Fiber width	AA	1.26E+00	5.42E-03
2	Potom.002G05522-SNP60	20	Potom.003G08321-SNP14	Tree height	DD	-9.30E+00	5.42E-03
5	Potom.005G13176-SNP27	8	Potom.008G21952-SNP16	Holocellulose content	AD	1.53E+01	5.42E-03
8	Potom.008G22699-SNP108	12	Potom.012G28819-SNP45	Holocellulose content	AD	9.52E+00	5.42E-03
2	Potom.002G05522-SNP62	20	Potom.003G08321-SNP14	Tree height	DD	-9.36E+00	5.41E-03
10	Potom.010G25294-SNP173	10	Potom.010G25398-SNP26	Microfiber angle	AD	2.68E+00	5.41E-03
10	Potom.010G25398-SNP26	18	Potom.018G36220-SNP43	Stem volume	DA	-3.97E-01	5.40E-03
2	Potom.002G05522-SNP4	2	Potom.002G05522-SNP15	Holocellulose content	DA	-1.08E+01	5.39E-03
13	Potom.013G29538-SNP5	20	Potom.003G09473-SNP12	Stem volume	AD	-8.82E-01	5.39E-03
5	Pto-Wuschela-SNP10	16	Potom.016G33876-SNP74	Fiber length	DA	-5.68E-02	5.38E-03
10	Potom.010G24967-SNP43	16	Potom.016G33891-SNP6	Fiber length	AA	-7.63E-02	5.38E-03
5	Potom.005G13966-SNP90	10	Potom.010G25294-SNP212	Diameter at breast height	DD	-1.57E+01	5.37E-03

8	Potom.008G21952-SNP16	16	Potom.016G33891-SNP29	Fiber length	AD	-1.86E-01	5.36E-03
16	Potom.016G33891-SNP26	20	Potom.003G08321-SNP26	Stem volume	DD	-8.74E-01	5.36E-03
1	Potom.001G03193-SNP13	2	Potom.002G05522-SNP1	Stem volume	AA	-5.44E-01	5.35E-03
2	Potom.002G07963-SNP153	5	Potom.005G13176-SNP26	Hemicellulose content	DD	2.61E+01	5.35E-03
5	Pto-Wuschela-SNP10	16	Potom.016G33891-SNP17	Fiber width	DD	-3.90E+00	5.35E-03
5	Potom.005G13333-SNP13	16	Potom.016G34352-SNP45	Holocellulose content	AD	-2.49E+01	5.35E-03
5	Potom.005G13176-SNP117	13	Potom.013G29538-SNP72	Hemicellulose content	DD	1.44E+01	5.34E-03
1	Potom.001G03606-SNP26	5	Potom.005G13333-SNP13	Lignin content	DD	5.30E+00	5.33E-03
1	Potom.001G00363-SNP53	5	Pto-Wuschela-SNP14	Holocellulose content	AD	-1.55E+01	5.32E-03
2	Potom.002G05522-SNP62	20	Potom.003G08321-SNP13	Tree height	DD	-8.89E+00	5.32E-03
5	Potom.005G13176-SNP162	10	Potom.010G24967-SNP58	Holocellulose content	DA	2.43E+01	5.31E-03
1	Potom.001G03353-SNP24	13	Potom.013G29538-SNP9	Holocellulose content	DA	-2.09E+01	5.30E-03
1	Potom.001G03606-SNP27	5	Potom.005G13176-SNP4	α-cellulose content	DD	2.26E+01	5.30E-03
2	Potom.002G05522-SNP54	20	Potom.003G08321-SNP18	Stem volume	DA	-3.31E-01	5.29E-03
2	Potom.002G05522-SNP60	10	Potom.010G24967-SNP67	Stem volume	DA	-3.16E-01	5.29E-03
1	Potom.001G03353-SNP164	5	Pto-Wuschela-SNP14	Lignin content	AD	2.80E+00	5.28E-03
8	Potom.008G21952-SNP16	20	Potom.003G09473-SNP11	Stem volume	AA	-5.15E-01	5.28E-03
10	Potom.010G25398-SNP26	18	Potom.018G36220-SNP42	Stem volume	DA	-4.02E-01	5.27E-03
16	Potom.016G34352-SNP108	20	Potom.003G09473-SNP2	Fiber length	AD	9.21E-02	5.27E-03
1	Potom.001G03193-SNP20	8	Potom.008G21952-SNP16	Holocellulose content	AD	1.72E+01	5.26E-03
2	Potom.002G05522-SNP13	20	Potom.003G08321-SNP4	Stem volume	DA	9.09E-01	5.26E-03
2	Potom.002G05522-SNP14	20	Potom.003G08321-SNP4	Stem volume	DA	9.09E-01	5.26E-03
2	Potom.002G05522-SNP60	20	Potom.003G08321-SNP13	Tree height	DD	-8.82E+00	5.26E-03
1	Potom.001G03193-SNP30	10	Potom.010G25294-SNP212	Stem volume	DD	-9.53E-01	5.25E-03
2	Potom.002G05522-SNP62	20	Potom.003G08321-SNP32	Stem volume	AA	2.47E-01	5.25E-03
1	Potom.001G03193-SNP20	10	Potom.010G25398-SNP26	Holocellulose content	AA	-7.79E+00	5.24E-03

1	Potom.001G03353-SNP25	18	Potom.018G35976-SNP25	Fiber length	AD	1.00E-01	5.24E-03
2	Potom.002G05522-SNP12	20	Potom.003G09473-SNP9	Stem volume	DD	1.16E+00	5.24E-03
5	Potom.005G13176-SNP2	5	Potom.005G13985-SNP17	Stem volume	AA	-5.89E-01	5.24E-03
10	Potom.010G24967-SNP45	20	Potom.003G09473-SNP10	Holocellulose content	DA	1.88E+01	5.24E-03
14	Potom.014G31981-SNP25	16	Potom.016G34352-SNP98	Stem volume	AA	-2.67E-01	5.24E-03
14	Potom.014G31981-SNP25	20	Potom.003G09473-SNP13	Stem volume	AA	-5.25E-01	5.24E-03
5	Potom.005G13176-SNP44	8	Potom.008G21326-SNP66	Stem volume	AA	-3.80E-01	5.23E-03
8	Potom.008G22699-SNP74	10	Potom.010G24967-SNP43	Diameter at breast height	AD	-4.99E+00	5.23E-03
2	Potom.002G05522-SNP13	2	Potom.002G07337-SNP15	Diameter at breast height	AD	-1.03E+01	5.22E-03
2	Potom.002G05522-SNP14	2	Potom.002G07337-SNP15	Diameter at breast height	AD	-1.03E+01	5.22E-03
2	Potom.002G05522-SNP73	10	Potom.010G24967-SNP43	Lignin content	AD	2.42E+00	5.22E-03
5	Potom.005G13176-SNP4	16	Potom.016G34352-SNP98	α-cellulose content	AA	6.37E+00	5.22E-03
14	Potom.014G31981-SNP26	16	Potom.016G34352-SNP98	Stem volume	AA	-2.39E-01	5.22E-03
1	Potom.001G03353-SNP25	14	Potom.014G31981-SNP26	Fiber width	AD	2.95E+00	5.21E-03
2	Potom.002G07337-SNP147	10	Potom.010G25398-SNP26	Holocellulose content	AD	6.99E+00	5.21E-03
5	Potom.005G13985-SNP1	20	Potom.003G09473-SNP2	Fiber length	DA	-1.36E-01	5.21E-03
5	Potom.005G13985-SNP2	20	Potom.003G09473-SNP2	Fiber length	DA	-1.36E-01	5.21E-03
10	Potom.010G24967-SNP53	20	Potom.003G09473-SNP11	Hemicellulose content	AA	-1.23E+01	5.21E-03
16	Potom.016G33876-SNP25	20	Potom.003G08321-SNP17	Hemicellulose content	AD	1.94E+01	5.21E-03
1	Potom.001G00363-SNP19	10	Potom.010G24967-SNP45	Diameter at breast height	AA	-3.82E+00	5.20E-03
5	Potom.005G13176-SNP162	16	Potom.016G34352-SNP98	Stem volume	AA	-3.58E-01	5.20E-03
5	Pto-Wuschela-SNP5	5	Potom.005G13176-SNP154	Microfiber angle	AD	-5.21E+00	5.19E-03
8	Potom.008G21326-SNP55	10	Potom.010G24967-SNP58	Fiber length	AA	1.56E-01	5.19E-03
8	Potom.008G21952-SNP16	10	Potom.010G25398-SNP26	Diameter at breast height	AD	-9.55E+00	5.19E-03
8	Potom.008G22699-SNP71	10	Potom.010G25398-SNP26	Stem volume	DD	-8.13E-01	5.19E-03
13	Potom.013G29538-SNP6	20	Potom.003G08321-SNP17	Stem volume	AA	-3.31E-01	5.19E-03

2	Potom.002G05522-SNP3	2	Potom.002G05522-SNP4	Holocellulose content	DA	1.68E+01	5.18E-03
5	Potom.005G13985-SNP6	20	Potom.003G09473-SNP2	Fiber length	DD	-2.80E-01	5.18E-03
5	Pto-Wuschela-SNP18	10	Potom.010G24967-SNP53	Holocellulose content	DD	1.90E+01	5.17E-03
8	Potom.008G22699-SNP74	20	Potom.003G08321-SNP33	Diameter at breast height	AA	3.92E+00	5.17E-03
2	Potom.002G07963-SNP152	5	Potom.005G13176-SNP26	Hemicellulose content	DD	2.62E+01	5.16E-03
9	Potom.009G23029-SNP3	20	Potom.003G08321-SNP20	Holocellulose content	DA	1.24E+01	5.16E-03
2	Potom.002G05522-SNP13	20	Potom.003G08321-SNP4	Stem volume	AA	-6.94E-01	5.15E-03
2	Potom.002G05522-SNP14	20	Potom.003G08321-SNP4	Stem volume	AA	-6.94E-01	5.15E-03
5	Potom.005G13176-SNP47	20	Potom.003G08321-SNP5	Fiber width	AA	-2.66E+00	5.14E-03
13	Potom.013G29538-SNP72	16	Potom.016G33876-SNP108	Diameter at breast height	AA	-4.13E+00	5.14E-03
2	Potom.002G05522-SNP54	8	Potom.008G22699-SNP108	Fiber length	AA	-6.81E-02	5.13E-03
5	Pto-Wuschela-SNP12	8	Potom.008G22699-SNP150	α-cellulose content	AD	1.18E+01	5.13E-03
1	Potom.001G03193-SNP13	2	Potom.002G05522-SNP13	Stem volume	DA	7.33E-01	5.12E-03
1	Potom.001G03193-SNP13	2	Potom.002G05522-SNP14	Stem volume	DA	7.33E-01	5.12E-03
2	Potom.002G05769-SNP57	20	Potom.003G08321-SNP6	Stem volume	AA	5.20E-01	5.12E-03
2	Potom.002G07337-SNP229	5	Pto-Wuschela-SNP5	Diameter at breast height	AA	-2.47E+00	5.12E-03
5	Potom.005G13966-SNP90	16	Potom.016G33876-SNP31	Stem volume	DA	-5.73E-01	5.12E-03
1	Potom.001G03353-SNP25	18	Potom.018G35976-SNP25	Tree height	AD	3.31E+00	5.11E-03
2	Potom.002G05522-SNP52	8	Potom.008G22699-SNP70	α-cellulose content	AD	-1.16E+01	5.11E-03
5	Potom.005G13176-SNP162	5	Potom.005G13176-SNP193	Fiber length	AA	-2.12E-02	5.11E-03
13	Potom.013G29538-SNP5	20	Potom.003G09473-SNP10	Stem volume	AD	-8.78E-01	5.11E-03
2	Potom.002G07337-SNP56	5	Potom.005G13966-SNP89	Stem volume	AD	-8.99E-01	5.10E-03
5	Pto-Wuschela-SNP14	14	Potom.014G31981-SNP25	α-cellulose content	AA	-5.02E+00	5.10E-03
2	Potom.002G05522-SNP73	8	Potom.008G21952-SNP16	Fiber length	AA	6.41E-02	5.09E-03
2	Potom.002G07337-SNP15	12	Potom.012G28819-SNP8	Microfiber angle	DA	6.19E+00	5.09E-03
2	Potom.002G07963-SNP170	20	Potom.003G09473-SNP9	Holocellulose content	AA	1.14E+01	5.09E-03

5	Potom.005G13176-SNP2	20	Potom.003G09473-SNP2	Stem volume	DA	-8.07E-01	5.09E-03
5	Potom.005G13176-SNP115	16	Potom.016G33876-SNP25	Diameter at breast height	DA	-1.33E+01	5.09E-03
13	Potom.013G29538-SNP2	13	Potom.013G29538-SNP72	Holocellulose content	DA	1.01E+01	5.09E-03
2	Potom.002G05522-SNP23	16	Potom.016G33876-SNP74	Fiber width	DA	-1.59E+00	5.08E-03
1	Potom.001G03606-SNP41	2	Potom.002G05769-SNP43	Diameter at breast height	AA	-6.59E+00	5.07E-03
5	Pto-Wuschela-SNP12	5	Potom.005G13176-SNP162	Lignin content	DD	4.14E+00	5.07E-03
5	Potom.005G13966-SNP97	10	Potom.010G25398-SNP63	Diameter at breast height	AA	-7.30E+00	5.07E-03
5	Potom.005G13176-SNP41	13	Potom.013G29538-SNP71	α-cellulose content	AA	7.46E+00	5.06E-03
5	Potom.005G13966-SNP74	16	Potom.016G34352-SNP109	Holocellulose content	AA	1.04E+01	5.06E-03
5	Potom.005G13966-SNP89	8	Potom.008G22699-SNP74	Diameter at breast height	DA	-8.45E+00	5.06E-03
1	Potom.001G03193-SNP27	12	Potom.012G28819-SNP27	Fiber length	AD	-1.71E-01	5.05E-03
1	Potom.001G03606-SNP41	2	Potom.002G05522-SNP23	Tree height	AD	-2.81E+00	5.05E-03
5	Potom.005G13176-SNP115	16	Potom.016G33891-SNP29	Fiber length	AD	-1.51E-01	5.05E-03
1	Potom.001G03353-SNP25	2	Potom.002G05522-SNP60	Stem volume	AA	2.33E-01	5.04E-03
10	Potom.010G24967-SNP45	20	Potom.003G09473-SNP13	Hemicellulose content	AA	-8.35E+00	5.04E-03
2	Potom.002G05522-SNP1	20	Potom.003G08321-SNP17	Hemicellulose content	DA	1.48E+01	5.03E-03
2	Potom.002G05522-SNP73	13	Potom.013G29538-SNP72	Microfiber angle	AA	-3.19E+00	5.03E-03
2	Potom.002G05769-SNP70	12	Potom.012G28819-SNP27	Diameter at breast height	DD	2.01E+01	5.03E-03
5	Potom.005G13176-SNP115	16	Potom.016G33891-SNP27	Fiber length	AA	7.37E-02	5.03E-03
8	Potom.008G21326-SNP10	10	Potom.010G24967-SNP58	Fiber length	AA	1.55E-01	5.03E-03
10	Potom.010G25706-SNP89	13	Potom.013G29538-SNP117	Fiber width	DA	-1.86E+00	5.03E-03
13	Potom.013G29538-SNP7	20	Potom.003G08321-SNP17	Diameter at breast height	AA	-4.66E+00	5.03E-03
1	Potom.001G03606-SNP26	5	Potom.005G13985-SNP1	Fiber length	AA	1.11E-01	5.02E-03
1	Potom.001G03606-SNP26	5	Potom.005G13985-SNP2	Fiber length	AA	1.11E-01	5.02E-03
2	Potom.002G05522-SNP23	16	Potom.016G33891-SNP13	Diameter at breast height	DD	1.24E+01	5.02E-03
5	Pto-Wuschela-SNP12	20	Potom.003G08321-SNP3	Holocellulose content	DA	1.46E+01	5.02E-03

2	Potom.002G05769-SNP72	8	Potom.008G22699-SNP74	Stem volume	AD	5.08E-01	5.01E-03
1	Potom.001G03193-SNP20	5	Pto-Wuschela-SNP10	Holocellulose content	AD	-9.62E+00	5.00E-03
2	Potom.002G05522-SNP13	8	Potom.008G22699-SNP72	Hemicellulose content	AD	-1.58E+01	5.00E-03
2	Potom.002G05522-SNP14	8	Potom.008G22699-SNP72	Hemicellulose content	AD	-1.58E+01	5.00E-03
2	Potom.002G05522-SNP23	5	Potom.005G13966-SNP77	Tree height	DA	-1.43E+00	5.00E-03
5	Potom.005G13966-SNP97	20	Potom.003G08321-SNP18	Stem volume	AA	-2.67E-01	5.00E-03
5	Potom.005G13985-SNP1	10	Potom.010G25398-SNP63	Holocellulose content	AD	2.47E+01	5.00E-03
5	Potom.005G13985-SNP2	10	Potom.010G25398-SNP63	Holocellulose content	AD	2.47E+01	5.00E-03
20	Potom.003G08321-SNP9	20	Potom.003G09473-SNP2	Fiber length	AD	1.47E-01	5.00E-03
1	Potom.001G03606-SNP41	2	Potom.002G05769-SNP43	Stem volume	AA	-4.73E-01	4.99E-03
2	Potom.002G07337-SNP15	5	Potom.005G13333-SNP18	α-cellulose content	AA	-5.60E+00	4.99E-03
2	Potom.002G07337-SNP147	5	Potom.005G13985-SNP5	Fiber length	DD	-1.96E-01	4.99E-03
5	Pto-Wuschela-SNP10	20	Potom.003G08321-SNP7	α-cellulose content	AA	-4.64E+00	4.99E-03
5	Potom.005G13966-SNP97	20	Potom.003G09473-SNP2	Microfiber angle	AD	-5.60E+00	4.98E-03
2	Potom.002G05522-SNP23	5	Potom.005G13176-SNP44	Fiber length	DD	-1.32E-01	4.97E-03
5	Potom.005G13176-SNP68	5	Potom.005G13985-SNP20	Diameter at breast height	DA	8.98E+00	4.97E-03
2	Potom.002G05522-SNP54	13	Potom.013G29538-SNP4	Stem volume	AA	3.21E-01	4.96E-03
5	Pto-Wuschela-SNP10	14	Potom.014G31981-SNP25	Hemicellulose content	DA	-1.32E+01	4.96E-03
2	Potom.002G07963-SNP72	16	Potom.016G33891-SNP26	Fiber length	AA	-3.84E-02	4.95E-03
1	Potom.001G00363-SNP23	8	Potom.008G22699-SNP71	Holocellulose content	AA	1.41E+01	4.94E-03
5	Potom.005G13176-SNP44	12	Potom.012G28409-SNP23	Tree height	DA	-2.24E+00	4.94E-03
5	Potom.005G13176-SNP15	5	Potom.005G13966-SNP97	Hemicellulose content	AA	8.90E+00	4.93E-03
8	Potom.008G22699-SNP108	16	Potom.016G34352-SNP109	Tree height	DA	3.10E+00	4.93E-03
1	Potom.001G03193-SNP26	16	Potom.016G33876-SNP73	Diameter at breast height	AD	-9.05E+00	4.92E-03
5	Potom.005G13176-SNP2	13	Potom.013G29538-SNP9	Holocellulose content	AA	1.29E+01	4.92E-03
5	Potom.005G13176-SNP44	5	Potom.005G13333-SNP12	Lignin content	DA	2.66E+00	4.92E-03

1	Potom.001G00363-SNP23	2	Potom.002G07337-SNP15	Hemicellulose content	AD	1.41E+01	4.91E-03
2	Potom.002G07337-SNP229	20	Potom.003G08321-SNP2	Diameter at breast height	DA	9.02E+00	4.91E-03
8	Potom.008G21952-SNP16	10	Potom.010G25398-SNP65	α-cellulose content	AA	1.20E+01	4.91E-03
1	Potom.001G03353-SNP164	5	Pto-Wuschela-SNP14	Holocellulose content	AD	-1.08E+01	4.90E-03
5	Pto-Wuschela-SNP12	8	Potom.008G22699-SNP108	Holocellulose content	AD	-5.41E+00	4.90E-03
8	Potom.008G22516-SNP49	10	Potom.010G25706-SNP40	Microfiber angle	AA	6.50E+00	4.90E-03
12	Potom.012G28819-SNP8	16	Potom.016G33891-SNP6	Diameter at breast height	AA	-8.00E+00	4.90E-03
1	Potom.001G03193-SNP27	2	Potom.002G05632-SNP65	Lignin content	AA	-8.91E-01	4.89E-03
1	Potom.001G03353-SNP161	5	Potom.005G13966-SNP89	Diameter at breast height	DD	-1.48E+01	4.87E-03
5	Potom.005G13985-SNP4	16	Potom.016G33891-SNP29	Fiber length	DD	-3.03E-01	4.87E-03
10	Potom.010G24967-SNP67	10	Potom.010G25398-SNP26	Stem volume	AA	-2.20E-01	4.87E-03
2	Potom.002G05522-SNP62	10	Potom.010G24967-SNP43	Fiber length	DA	-7.46E-02	4.86E-03
2	Potom.002G05769-SNP13	5	Potom.005G13966-SNP90	Holocellulose content	AA	1.68E+01	4.86E-03
5	Potom.005G13176-SNP16	10	Potom.010G24967-SNP43	Stem volume	AD	-4.91E-01	4.85E-03
5	Potom.005G13176-SNP27	18	Potom.018G35976-SNP13	Stem volume	DD	8.98E-01	4.85E-03
10	Potom.010G25398-SNP26	20	Potom.003G08321-SNP17	Holocellulose content	DD	1.64E+01	4.85E-03
16	Potom.016G34352-SNP109	20	Potom.003G09473-SNP2	Tree height	AD	3.63E+00	4.85E-03
1	Potom.001G00363-SNP19	10	Potom.010G24967-SNP43	Diameter at breast height	AA	-3.86E+00	4.84E-03
1	Potom.001G00363-SNP23	2	Potom.002G07337-SNP15	Hemicellulose content	DD	2.22E+01	4.84E-03
2	Potom.002G05522-SNP12	8	Potom.008G22699-SNP3	Fiber width	AA	1.49E+00	4.84E-03
8	Potom.008G22699-SNP3	20	Potom.003G08321-SNP15	Fiber length	AA	9.51E-02	4.84E-03
10	Potom.010G24967-SNP43	10	Potom.010G25398-SNP26	Stem volume	DA	-5.67E-01	4.84E-03
16	Potom.016G33876-SNP94	20	Potom.003G09473-SNP10	Lignin content	DA	-4.67E+00	4.84E-03
1	Potom.001G03193-SNP20	2	Potom.002G05522-SNP62	Lignin content	AA	-1.98E+00	4.83E-03
1	Potom.001G03193-SNP27	16	Potom.016G34352-SNP108	Holocellulose content	AD	1.31E+01	4.83E-03
5	Potom.005G13985-SNP20	16	Potom.016G33891-SNP27	Stem volume	AD	7.13E-01	4.83E-03

2	Potom.002G05522-SNP12	10	Potom.010G25398-SNP26	Diameter at breast height	AD	-8.38E+00	4.82E-03
2	Potom.002G05769-SNP37	8	Potom.008G21326-SNP69	Stem volume	AA	-6.07E-01	4.82E-03
5	Potom.005G13966-SNP97	10	Potom.010G25706-SNP89	Stem volume	AD	5.68E-01	4.82E-03
8	Potom.008G21326-SNP69	20	Potom.003G09473-SNP13	Stem volume	AA	-4.84E-01	4.82E-03
5	Potom.005G13176-SNP2	10	Potom.010G25706-SNP89	Fiber width	DD	4.99E+00	4.81E-03
5	Potom.005G13176-SNP44	12	Potom.012G28819-SNP27	Diameter at breast height	DD	2.03E+01	4.81E-03
10	Potom.010G25706-SNP89	13	Potom.013G29538-SNP117	Diameter at breast height	AA	3.38E+00	4.81E-03
1	Potom.001G03606-SNP26	2	Potom.002G05522-SNP12	Tree height	DA	-5.40E+00	4.80E-03
1	Potom.001G03193-SNP20	13	Potom.013G29538-SNP72	Diameter at breast height	DA	-7.40E+00	4.79E-03
5	Potom.005G13333-SNP13	13	Potom.013G29538-SNP4	Holocellulose content	AA	5.31E+00	4.79E-03
5	Potom.005G13333-SNP18	12	Potom.012G28819-SNP9	Tree height	DD	-4.67E+00	4.79E-03
8	Potom.008G22699-SNP9	12	Potom.012G28409-SNP23	Stem volume	DA	-5.25E-01	4.78E-03
1	Potom.001G03606-SNP26	2	Potom.002G05522-SNP73	Fiber width	AD	3.49E+00	4.77E-03
2	Potom.002G05522-SNP12	5	Potom.005G13985-SNP4	Fiber length	DD	-2.88E-01	4.77E-03
2	Potom.002G05522-SNP60	5	Potom.005G13176-SNP26	Hemicellulose content	AD	-1.25E+01	4.77E-03
2	Potom.002G07206-SNP14	5	Pto-Wuschela-SNP10	Fiber width	AD	3.46E+00	4.77E-03
2	Potom.002G07337-SNP16	20	Potom.003G10089-SNP7	Microfiber angle	AD	-6.84E+00	4.77E-03
8	Potom.008G22699-SNP69	20	Potom.003G09473-SNP2	Stem volume	AD	6.51E-01	4.77E-03
16	Potom.016G34352-SNP108	20	Potom.003G09473-SNP2	Stem volume	AD	4.44E-01	4.77E-03
1	Potom.001G03606-SNP26	10	Potom.010G25398-SNP68	Hemicellulose content	AA	-1.20E+01	4.76E-03
2	Potom.002G05769-SNP43	8	Potom.008G21952-SNP16	α-cellulose content	AD	-1.43E+01	4.76E-03
1	Potom.001G03606-SNP26	2	Potom.002G05769-SNP13	Holocellulose content	DD	2.75E+01	4.75E-03
2	Potom.002G07337-SNP229	5	Potom.005G13176-SNP172	Diameter at breast height	AA	-7.46E+00	4.75E-03
5	Potom.005G13966-SNP74	16	Potom.016G34352-SNP109	Hemicellulose content	AD	1.41E+01	4.75E-03
9	Potom.009G23029-SNP3	13	Potom.013G29538-SNP4	Tree height	AA	-1.95E+00	4.75E-03
10	Potom.010G24967-SNP43	20	Potom.003G09473-SNP13	Hemicellulose content	AA	-8.41E+00	4.75E-03

2	Potom.002G05522-SNP12	20	Potom.003G08321-SNP21	Fiber width	AA	1.14E+00	4.74E-03
2	Potom.002G05522-SNP62	5	Potom.005G13176-SNP26	Hemicellulose content	AD	-1.25E+01	4.74E-03
2	Potom.002G07337-SNP34	20	Potom.003G08321-SNP7	Lignin content	AD	-1.71E+00	4.74E-03
1	Potom.001G00363-SNP23	8	Potom.008G22699-SNP108	Lignin content	DA	-3.87E+00	4.73E-03
2	Potom.002G05522-SNP13	14	Potom.014G31981-SNP25	Lignin content	AA	3.65E+00	4.73E-03
2	Potom.002G05522-SNP14	14	Potom.014G31981-SNP25	Lignin content	AA	3.65E+00	4.73E-03
2	Potom.002G07963-SNP72	5	Potom.005G13176-SNP115	Fiber length	AA	-4.16E-02	4.73E-03
10	Potom.010G25706-SNP79	12	Potom.012G28819-SNP9	Stem volume	DA	-3.95E-01	4.73E-03
1	Potom.001G01788-SNP70	2	Potom.002G05522-SNP60	Fiber length	AD	-7.98E-02	4.72E-03
2	Potom.002G07337-SNP229	16	Potom.016G34352-SNP98	α-cellulose content	AD	-9.01E+00	4.72E-03
20	Potom.003G08321-SNP20	20	Potom.003G09473-SNP11	Stem volume	AA	2.98E-01	4.72E-03
2	Potom.002G05522-SNP73	10	Potom.010G24967-SNP43	Hemicellulose content	AA	-7.03E+00	4.70E-03
2	Potom.002G07337-SNP15	16	Potom.016G33891-SNP13	Fiber width	DD	4.93E+00	4.69E-03
16	Potom.016G34352-SNP98	20	Potom.003G08321-SNP17	α-cellulose content	AD	5.23E+00	4.69E-03
1	Potom.001G00363-SNP23	20	Potom.003G08321-SNP2	Diameter at breast height	DA	-1.56E+01	4.68E-03
1	Potom.001G03193-SNP20	16	Potom.016G33876-SNP71	Lignin content	DA	-2.08E+00	4.68E-03
2	Potom.002G05522-SNP1	13	Potom.013G29538-SNP2	Holocellulose content	DD	-3.20E+01	4.68E-03
2	Potom.002G05522-SNP60	12	Potom.012G28409-SNP23	Tree height	AD	2.57E+00	4.68E-03
1	Potom.001G01788-SNP70	2	Potom.002G05522-SNP62	Fiber length	AD	-8.03E-02	4.67E-03
2	Potom.002G05632-SNP14	5	Potom.005G13176-SNP62	Diameter at breast height	AA	-3.74E+00	4.67E-03
16	Potom.016G34352-SNP44	20	Potom.003G09473-SNP12	Holocellulose content	AA	1.62E+01	4.67E-03
2	Potom.002G05522-SNP4	5	Potom.005G13966-SNP74	Holocellulose content	DA	-1.44E+01	4.66E-03
5	Pto-Wuschela-SNP14	14	Potom.014G31981-SNP26	α-cellulose content	AA	-4.48E+00	4.66E-03
8	Potom.008G22699-SNP3	8	Potom.008G22699-SNP72	Stem volume	AD	4.65E-01	4.66E-03
2	Potom.002G07337-SNP56	5	Potom.005G13176-SNP2	Holocellulose content	AA	1.35E+01	4.65E-03
1	Potom.001G00363-SNP53	5	Pto-Wuschela-SNP10	Hemicellulose content	AD	-1.24E+01	4.63E-03

1	Potom.001G03606-SNP27	14	Potom.014G31981-SNP25	Tree height	AA	-3.38E+00	4.62E-03
5	Potom.005G13966-SNP89	16	Potom.016G34352-SNP109	Hemicellulose content	AD	1.33E+01	4.62E-03
2	Potom.002G05769-SNP72	5	Potom.005G13333-SNP13	α-cellulose content	DA	1.56E+01	4.61E-03
2	Potom.002G05522-SNP3	13	Potom.013G29538-SNP5	Diameter at breast height	AD	-8.86E+00	4.59E-03
2	Potom.002G05522-SNP12	2	Potom.002G05522-SNP54	α-cellulose content	DA	-9.23E+00	4.58E-03
1	Potom.001G03606-SNP29	20	Potom.003G08321-SNP14	Diameter at breast height	DA	1.55E+01	4.57E-03
2	Potom.002G05522-SNP15	5	Pto-Wuschela-SNP12	Holocellulose content	AD	-1.12E+01	4.57E-03
2	Potom.002G07337-SNP229	20	Potom.003G09473-SNP13	Fiber length	AA	-7.32E-02	4.57E-03
8	Potom.008G22699-SNP83	13	Potom.013G29538-SNP14	Diameter at breast height	AD	1.30E+01	4.57E-03
1	Potom.001G03606-SNP29	5	Potom.005G13176-SNP68	Diameter at breast height	DA	1.52E+01	4.56E-03
5	Potom.005G13176-SNP194	13	Potom.013G29538-SNP14	Fiber length	AD	1.21E-01	4.56E-03
5	Potom.005G13333-SNP12	8	Potom.008G22699-SNP98	α-cellulose content	AD	2.17E+01	4.56E-03
13	Potom.013G29538-SNP71	20	Potom.003G08321-SNP3	Fiber width	DA	3.34E+00	4.56E-03
10	Potom.010G25398-SNP26	16	Potom.016G33891-SNP29	Stem volume	DA	-6.48E-01	4.55E-03
2	Potom.002G05522-SNP12	5	Potom.005G13966-SNP90	Stem volume	DD	-8.94E-01	4.54E-03
2	Potom.002G05522-SNP15	8	Potom.008G21326-SNP66	Diameter at breast height	AA	-5.59E+00	4.54E-03
5	Potom.005G13333-SNP18	20	Potom.003G08321-SNP3	α-cellulose content	AD	-9.97E+00	4.54E-03
8	Potom.008G22699-SNP76	20	Potom.003G08321-SNP2	Fiber length	DA	1.43E-01	4.54E-03
8	Potom.008G22699-SNP85	10	Potom.010G24967-SNP53	Lignin content	DD	-8.50E+00	4.54E-03
1	Potom.001G00363-SNP23	8	Potom.008G22699-SNP108	Fiber length	AD	1.36E-01	4.53E-03
2	Potom.002G07206-SNP38	16	Potom.016G33891-SNP28	Fiber width	DD	5.19E+00	4.53E-03
5	Potom.005G13176-SNP44	5	Potom.005G13333-SNP13	Fiber width	AD	-2.40E+00	4.53E-03
2	Potom.002G05522-SNP15	8	Potom.008G22699-SNP100	Diameter at breast height	DA	4.86E+00	4.52E-03
5	Potom.005G13333-SNP18	16	Potom.016G33876-SNP53	Holocellulose content	DD	2.23E+01	4.52E-03
20	Potom.003G08321-SNP17	20	Potom.003G10130-SNP25	α-cellulose content	AD	-1.14E+01	4.52E-03
2	Potom.002G05769-SNP25	8	Potom.008G21952-SNP16	Diameter at breast height	AA	-7.05E+00	4.51E-03

2	Potom.002G07963-SNP71	18	Potom.018G36220-SNP1	Fiber length	AD	-5.93E-02	4.51E-03
5	Potom.005G13176-SNP4	20	Potom.003G09473-SNP10	α-cellulose content	DD	-2.92E+01	4.51E-03
8	Potom.008G21326-SNP69	20	Potom.003G09473-SNP11	Stem volume	AA	-4.77E-01	4.51E-03
1	Potom.001G03606-SNP26	20	Potom.003G08321-SNP4	Tree height	DD	8.28E+00	4.50E-03
5	Potom.005G13176-SNP162	13	Potom.013G29538-SNP16	Lignin content	AA	-1.46E+00	4.50E-03
1	Potom.001G00363-SNP23	10	Potom.010G25398-SNP68	Stem volume	AD	1.05E+00	4.49E-03
1	Potom.001G03193-SNP30	2	Potom.002G05522-SNP62	Fiber length	AD	-1.31E-01	4.48E-03
2	Potom.002G05522-SNP60	2	Potom.002G07963-SNP163	Fiber length	AD	-1.03E-01	4.48E-03
2	Potom.002G05522-SNP62	2	Potom.002G07963-SNP163	Fiber length	AD	-1.03E-01	4.48E-03
2	Potom.002G05769-SNP43	8	Potom.008G21952-SNP16	Diameter at breast height	DA	-8.50E+00	4.48E-03
8	Potom.008G22516-SNP49	16	Potom.016G33876-SNP72	Fiber width	DA	-2.65E+00	4.48E-03
2	Potom.002G05522-SNP13	8	Potom.008G22699-SNP71	Hemicellulose content	AD	-1.62E+01	4.47E-03
2	Potom.002G05522-SNP14	8	Potom.008G22699-SNP71	Hemicellulose content	AD	-1.62E+01	4.47E-03
10	Potom.010G24967-SNP43	20	Potom.003G09473-SNP10	Holocellulose content	DA	1.92E+01	4.47E-03
10	Potom.010G24967-SNP45	10	Potom.010G25398-SNP26	Stem volume	DA	-5.63E-01	4.47E-03
14	Potom.014G31981-SNP26	20	Potom.003G08321-SNP17	Holocellulose content	AA	-9.68E+00	4.47E-03
1	Potom.001G01788-SNP70	2	Potom.002G05522-SNP60	Fiber length	DA	-9.19E-02	4.46E-03
1	Potom.001G03353-SNP25	5	Pto-Wuschela-SNP18	Lignin content	AA	1.18E+00	4.46E-03
10	Potom.010G24967-SNP40	20	Potom.003G09473-SNP12	Holocellulose content	DA	1.74E+01	4.46E-03
5	Pto-Wuschela-SNP14	5	Potom.005G13176-SNP62	Lignin content	DA	2.84E+00	4.45E-03
5	Potom.005G13966-SNP93	8	Potom.008G22699-SNP108	Fiber length	AD	1.17E-01	4.45E-03
5	Pto-Wuschela-SNP10	8	Potom.008G22699-SNP72	Holocellulose content	AD	1.02E+01	4.43E-03
5	Potom.005G13176-SNP3	20	Potom.003G09473-SNP10	α-cellulose content	DD	-3.01E+01	4.43E-03
5	Potom.005G13966-SNP77	13	Potom.013G29538-SNP72	Stem volume	AD	-2.39E-01	4.43E-03
2	Potom.002G07206-SNP24	10	Potom.010G24967-SNP45	Fiber length	AD	-8.71E-02	4.42E-03
1	Potom.001G03353-SNP25	5	Potom.005G13966-SNP93	Lignin content	DA	3.81E+00	4.41E-03

2	Potom.002G05522-SNP4	5	Potom.005G13176-SNP47	Hemicellulose content	AA	-5.73E+00	4.41E-03
5	Potom.005G13333-SNP12	16	Potom.016G34352-SNP109	Fiber width	AD	-2.74E+00	4.41E-03
2	Potom.002G05769-SNP57	5	Potom.005G13176-SNP26	Hemicellulose content	DD	2.64E+01	4.40E-03
5	Potom.005G13176-SNP41	8	Potom.008G22699-SNP74	α-cellulose content	AA	-4.31E+00	4.39E-03
5	Potom.005G13333-SNP18	13	Potom.013G29538-SNP117	α-cellulose content	AA	-3.34E+00	4.39E-03
5	Potom.005G13966-SNP77	20	Potom.003G08321-SNP3	Stem volume	AA	1.74E-01	4.39E-03
2	Potom.002G07337-SNP34	16	Potom.016G33891-SNP17	α-cellulose content	AD	-9.24E+00	4.37E-03
2	Potom.002G07337-SNP229	16	Potom.016G33876-SNP108	Stem volume	DA	-4.41E-01	4.37E-03
5	Potom.005G13176-SNP162	13	Potom.013G29538-SNP15	Lignin content	AA	-1.52E+00	4.37E-03
5	Potom.005G13333-SNP12	8	Potom.008G22699-SNP70	Microfiber angle	AD	7.45E+00	4.37E-03
5	Potom.005G13176-SNP16	10	Potom.010G24967-SNP45	Stem volume	AD	-4.98E-01	4.36E-03
5	Potom.005G13176-SNP27	8	Potom.008G22699-SNP3	Holocellulose content	DD	1.92E+01	4.36E-03
18	Potom.018G35976-SNP25	20	Potom.003G09473-SNP2	Diameter at breast height	AD	7.50E+00	4.36E-03
2	Potom.002G05522-SNP15	2	Potom.002G05522-SNP54	Tree height	AA	-3.20E+00	4.35E-03
1	Potom.001G03193-SNP30	2	Potom.002G05522-SNP60	Fiber length	AD	-1.31E-01	4.34E-03
2	Potom.002G07337-SNP229	16	Potom.016G34352-SNP98	Stem volume	AA	3.75E-01	4.34E-03
1	Potom.001G03193-SNP26	5	Potom.005G13333-SNP12	Stem volume	DA	5.62E-01	4.33E-03
5	Pto-Wuschela-SNP12	5	Potom.005G13176-SNP162	Hemicellulose content	DA	-7.35E+00	4.33E-03
5	Potom.005G13966-SNP77	10	Potom.010G25700-SNP13	Diameter at breast height	AA	-5.31E+00	4.33E-03
2	Potom.002G05522-SNP15	2	Potom.002G05522-SNP23	Fiber width	AD	2.60E+00	4.32E-03
8	Potom.008G22699-SNP108	14	Potom.014G31981-SNP26	Fiber length	DD	1.46E-01	4.31E-03
1	Potom.001G03606-SNP27	8	Potom.008G22699-SNP3	Stem volume	AD	-5.56E-01	4.30E-03
5	Pto-Wuschela-SNP18	5	Potom.005G13176-SNP41	Microfiber angle	AD	2.19E+00	4.30E-03
1	Potom.001G03193-SNP26	2	Potom.002G05522-SNP62	Lignin content	AA	-2.46E+00	4.29E-03
2	Potom.002G05522-SNP4	5	Potom.005G13176-SNP166	Holocellulose content	AD	-7.94E+00	4.29E-03
5	Potom.005G13176-SNP194	13	Potom.013G29538-SNP14	Diameter at breast height	AD	8.14E+00	4.29E-03

1	Potom.001G03193-SNP20	10	Potom.010G24967-SNP27	Microfiber angle	DD	9.12E+00	4.28E-03
1	Potom.001G03353-SNP161	10	Potom.010G24967-SNP27	α-cellulose content	AA	7.31E+00	4.28E-03
2	Potom.002G05769-SNP25	8	Potom.008G21326-SNP69	Stem volume	AA	-4.15E-01	4.28E-03
5	Potom.005G13176-SNP27	8	Potom.008G22699-SNP38	Diameter at breast height	AA	-9.35E+00	4.28E-03
12	Potom.012G28819-SNP45	20	Potom.003G08321-SNP17	Hemicellulose content	DA	-8.33E+00	4.28E-03
2	Potom.002G07963-SNP163	16	Potom.016G33876-SNP94	Fiber length	DA	-1.00E-01	4.27E-03
8	Potom.008G22699-SNP108	12	Potom.012G28819-SNP46	Holocellulose content	AD	1.17E+01	4.27E-03
1	Potom.001G01788-SNP70	2	Potom.002G05522-SNP62	Fiber length	DA	-9.30E-02	4.26E-03
2	Potom.002G05769-SNP57	20	Potom.003G08321-SNP32	Stem volume	AA	3.82E-01	4.26E-03
5	Potom.005G13966-SNP89	20	Potom.003G08321-SNP17	Diameter at breast height	DA	1.03E+01	4.26E-03
20	Potom.003G08321-SNP10	20	Potom.003G09473-SNP2	Microfiber angle	DD	-8.97E+00	4.26E-03
2	Potom.002G05522-SNP23	8	Potom.008G22699-SNP72	Stem volume	AD	4.45E-01	4.25E-03
2	Potom.002G05522-SNP62	10	Potom.010G24967-SNP45	Fiber length	AD	-8.01E-02	4.25E-03
2	Potom.002G05769-SNP43	8	Potom.008G22699-SNP74	Stem volume	DD	7.73E-01	4.25E-03
2	Potom.002G07337-SNP147	8	Potom.008G22699-SNP83	Lignin content	AA	1.48E+00	4.24E-03
10	Potom.010G24967-SNP45	16	Potom.016G33891-SNP6	Fiber length	AA	-7.44E-02	4.24E-03
16	Potom.016G34352-SNP45	20	Potom.003G09473-SNP10	Holocellulose content	DA	-2.26E+01	4.24E-03
2	Potom.002G05769-SNP13	8	Potom.008G22699-SNP72	Holocellulose content	DA	1.58E+01	4.23E-03
2	Potom.002G05769-SNP57	20	Potom.003G08321-SNP32	Diameter at breast height	AA	5.88E+00	4.23E-03
5	Potom.005G13966-SNP97	20	Potom.003G08321-SNP18	Stem volume	AD	6.00E-01	4.23E-03
1	Potom.001G00363-SNP21	10	Potom.010G25398-SNP68	Stem volume	AD	1.05E+00	4.22E-03
5	Potom.005G13985-SNP17	8	Potom.008G22699-SNP38	Tree height	DA	7.51E+00	4.22E-03
2	Potom.002G05522-SNP60	10	Potom.010G24967-SNP43	Fiber length	DA	-7.58E-02	4.20E-03
2	Potom.002G07337-SNP15	9	Potom.009G23029-SNP3	Holocellulose content	DD	1.77E+01	4.20E-03
5	Potom.005G13333-SNP13	8	Potom.008G22699-SNP74	α-cellulose content	DA	-7.79E+00	4.20E-03
8	Potom.008G22699-SNP22	12	Potom.012G28409-SNP11	Diameter at breast height	AA	-8.44E+00	4.19E-03

2	Potom.002G05522-SNP1	20	Potom.003G09473-SNP11	Hemicellulose content	DA	-1.79E+01	4.18E-03
10	Potom.010G25706-SNP79	13	Potom.013G29538-SNP16	Fiber width	DA	2.84E+00	4.18E-03
10	Potom.010G24967-SNP45	13	Potom.013G29538-SNP71	Hemicellulose content	AD	-1.32E+01	4.17E-03
13	Potom.013G29538-SNP72	20	Potom.003G08321-SNP21	Holocellulose content	AD	1.42E+01	4.17E-03
10	Potom.010G25706-SNP79	13	Potom.013G29538-SNP15	Fiber width	DA	2.86E+00	4.16E-03
16	Potom.016G34352-SNP98	20	Potom.003G08321-SNP17	Diameter at breast height	AD	-3.51E+00	4.16E-03
1	Potom.001G03353-SNP25	10	Potom.010G24967-SNP27	Holocellulose content	AA	9.14E+00	4.15E-03
2	Potom.002G05522-SNP54	20	Potom.003G08321-SNP17	Holocellulose content	DD	1.74E+01	4.15E-03
2	Potom.002G05769-SNP59	8	Potom.008G22516-SNP48	Diameter at breast height	AA	-9.78E+00	4.15E-03
5	Potom.005G13176-SNP16	12	Potom.012G28409-SNP23	Stem volume	DA	-2.60E-01	4.15E-03
10	Potom.010G25398-SNP68	20	Potom.003G08321-SNP4	Stem volume	DD	-1.39E+00	4.15E-03
5	Pto-Wuschela-SNP14	16	Potom.016G33891-SNP26	Lignin content	DA	3.24E+00	4.14E-03
1	Potom.001G03193-SNP26	16	Potom.016G33876-SNP73	Lignin content	DA	-2.84E+00	4.12E-03
10	Potom.010G25706-SNP89	13	Potom.013G29538-SNP5	Stem volume	AD	3.09E-01	4.12E-03
5	Potom.005G13966-SNP90	8	Potom.008G22699-SNP3	Stem volume	DA	-6.27E-01	4.11E-03
8	Potom.008G22699-SNP22	20	Potom.003G09473-SNP13	Stem volume	DD	1.08E+00	4.11E-03
2	Potom.002G05522-SNP23	8	Potom.008G22699-SNP22	Stem volume	DD	-8.57E-01	4.10E-03
2	Potom.002G05522-SNP60	20	Potom.003G08321-SNP32	Diameter at breast height	AA	3.69E+00	4.10E-03
2	Potom.002G05522-SNP1	20	Potom.003G09473-SNP9	Hemicellulose content	DD	3.00E+01	4.09E-03
8	Potom.008G22699-SNP108	18	Potom.018G35976-SNP25	Fiber length	DA	7.05E-02	4.09E-03
5	Potom.005G13966-SNP89	16	Potom.016G33876-SNP31	Diameter at breast height	DD	-1.35E+01	4.07E-03
8	Potom.008G22699-SNP71	8	Potom.008G22699-SNP108	Fiber length	AD	1.24E-01	4.07E-03
1	Potom.001G03193-SNP30	13	Potom.013G29538-SNP14	Diameter at breast height	AA	-9.52E+00	4.05E-03
13	Potom.013G29538-SNP9	20	Potom.003G08321-SNP17	Diameter at breast height	AA	-4.79E+00	4.05E-03
1	Potom.001G03193-SNP30	2	Potom.002G05522-SNP62	Tree height	AD	-4.52E+00	4.04E-03
8	Potom.008G21326-SNP52	10	Potom.010G24967-SNP58	Fiber length	AA	1.61E-01	4.04E-03

5	Potom.005G13176-SNP47	8	Potom.008G22699-SNP100	Lignin content	AD	2.81E+00	4.03E-03
8	Potom.008G22699-SNP38	12	Potom.012G28409-SNP11	Stem volume	AA	-6.25E-01	4.03E-03
8	Potom.008G22699-SNP72	13	Potom.013G29538-SNP9	Hemicellulose content	AA	1.08E+01	4.03E-03
13	Potom.013G29538-SNP9	20	Potom.003G08321-SNP13	Stem volume	DD	-1.30E+00	4.03E-03
2	Potom.002G07337-SNP129	10	Potom.010G25398-SNP63	α-cellulose content	AD	2.26E+01	4.02E-03
8	Potom.008G22699-SNP72	13	Potom.013G29538-SNP5	Holocellulose content	DD	2.17E+01	4.02E-03
8	Potom.008G22699-SNP38	12	Potom.012G28819-SNP46	Fiber length	AD	2.00E-01	4.01E-03
2	Potom.002G05522-SNP1	20	Potom.003G08321-SNP10	Fiber length	AD	1.45E-01	3.99E-03
2	Potom.002G05769-SNP37	20	Potom.003G08321-SNP26	Diameter at breast height	AD	-1.17E+01	3.99E-03
10	Potom.010G24967-SNP67	20	Potom.003G08321-SNP3	Diameter at breast height	AA	2.83E+00	3.99E-03
10	Potom.010G24967-SNP67	20	Potom.003G09473-SNP7	Diameter at breast height	AD	1.26E+01	3.99E-03
5	Potom.005G13966-SNP89	10	Potom.010G25706-SNP89	Diameter at breast height	DA	-8.73E+00	3.98E-03
8	Potom.008G22699-SNP71	14	Potom.014G31981-SNP25	Holocellulose content	AA	1.26E+01	3.98E-03
2	Potom.002G07206-SNP24	20	Potom.003G08321-SNP3	Lignin content	DD	4.37E+00	3.97E-03
1	Potom.001G03353-SNP25	10	Potom.010G24967-SNP45	Fiber length	AD	9.20E-02	3.96E-03
5	Potom.005G13176-SNP40	8	Potom.008G22699-SNP74	α-cellulose content	AA	-4.90E+00	3.96E-03
2	Potom.002G07337-SNP15	16	Potom.016G33891-SNP13	Fiber length	DD	2.08E-01	3.95E-03
2	Potom.002G07963-SNP229	8	Potom.008G22516-SNP49	Hemicellulose content	DD	-2.32E+01	3.95E-03
8	Potom.008G22699-SNP38	10	Potom.010G25706-SNP89	Stem volume	DA	5.94E-01	3.94E-03
2	Potom.002G05769-SNP25	20	Potom.003G08321-SNP17	Holocellulose content	AD	1.19E+01	3.92E-03
5	Potom.005G13176-SNP117	13	Potom.013G29538-SNP72	Hemicellulose content	DA	1.03E+01	3.91E-03
2	Potom.002G05522-SNP12	5	Potom.005G13966-SNP89	Stem volume	AD	7.35E-01	3.90E-03
8	Potom.008G22516-SNP49	12	Potom.012G28819-SNP8	Fiber width	AA	3.22E+00	3.90E-03
2	Potom.002G07337-SNP129	5	Potom.005G13176-SNP2	α-cellulose content	AD	1.86E+01	3.89E-03
1	Potom.001G00363-SNP23	20	Potom.003G09473-SNP9	Diameter at breast height	DD	-2.06E+01	3.88E-03
2	Potom.002G05522-SNP23	16	Potom.016G33876-SNP53	Diameter at breast height	DA	4.84E+00	3.88E-03

2	Potom.002G07337-SNP229	16	Potom.016G33891-SNP28	Stem volume	AA	-2.45E-01	3.88E-03
8	Potom.008G22699-SNP108	18	Potom.018G35976-SNP25	Lignin content	DA	-2.35E+00	3.87E-03
5	Potom.005G13966-SNP74	12	Potom.012G28819-SNP46	α-cellulose content	AA	1.04E+01	3.86E-03
2	Potom.002G05522-SNP23	5	Potom.005G13966-SNP89	Diameter at breast height	DD	-1.66E+01	3.85E-03
5	Potom.005G13333-SNP12	20	Potom.003G08321-SNP7	Fiber width	AD	2.86E+00	3.85E-03
1	Potom.001G03353-SNP25	5	Potom.005G13333-SNP18	Holocellulose content	AD	1.04E+01	3.84E-03
1	Potom.001G00363-SNP53	2	Potom.002G05522-SNP12	Hemicellulose content	DD	-2.64E+01	3.83E-03
2	Potom.002G05769-SNP13	10	Potom.010G24967-SNP43	Fiber length	AA	-1.05E-01	3.83E-03
8	Potom.008G22699-SNP3	14	Potom.014G31981-SNP25	Stem volume	DD	-7.24E-01	3.83E-03
16	Potom.016G33876-SNP108	16	Potom.016G33891-SNP28	Diameter at breast height	AD	-7.78E+00	3.83E-03
8	Potom.008G22699-SNP71	8	Potom.008G22699-SNP72	Hemicellulose content	AA	1.53E+01	3.82E-03
20	Potom.003G08321-SNP1	20	Potom.003G09473-SNP2	Stem volume	DA	1.06E+00	3.82E-03
2	Potom.002G05769-SNP57	12	Potom.012G28819-SNP8	Diameter at breast height	AA	-8.30E+00	3.81E-03
5	Pto-Wuschela-SNP14	20	Potom.003G10089-SNP11	Holocellulose content	DA	-1.27E+01	3.81E-03
10	Potom.010G24967-SNP43	13	Potom.013G29538-SNP71	Hemicellulose content	AD	-1.33E+01	3.81E-03
2	Potom.002G05522-SNP62	2	Potom.002G07337-SNP229	Tree height	AA	-1.46E+00	3.80E-03
2	Potom.002G05769-SNP43	2	Potom.002G07963-SNP185	α-cellulose content	AD	1.80E+01	3.80E-03
2	Potom.002G07337-SNP229	20	Potom.003G08321-SNP10	Stem volume	DD	-8.14E-01	3.80E-03
10	Potom.010G24967-SNP53	13	Potom.013G29538-SNP72	Fiber length	AA	-9.66E-02	3.80E-03
2	Potom.002G05522-SNP15	16	Potom.016G33876-SNP31	Holocellulose content	DA	-6.25E+00	3.79E-03
2	Potom.002G05769-SNP57	5	Potom.005G13176-SNP26	α-cellulose content	DD	-2.92E+01	3.79E-03
1	Potom.001G00363-SNP23	8	Potom.008G21952-SNP16	Holocellulose content	AD	1.91E+01	3.77E-03
5	Pto-Wuschela-SNP18	5	Potom.005G13176-SNP157	Microfiber angle	AD	2.68E+00	3.77E-03
1	Potom.001G03353-SNP25	2	Potom.002G07963-SNP72	Holocellulose content	AD	1.15E+01	3.76E-03
1	Potom.001G03606-SNP26	20	Potom.003G08321-SNP3	Holocellulose content	DD	-3.37E+01	3.76E-03
2	Potom.002G07337-SNP229	5	Potom.005G13176-SNP172	Stem volume	AA	-5.22E-01	3.76E-03

16	Potom.016G33876-SNP31	20	Potom.003G08321-SNP10	Fiber length	AD	-8.84E-02	3.76E-03
5	Potom.005G13176-SNP39	20	Potom.003G08321-SNP26	Diameter at breast height	AD	-7.93E+00	3.75E-03
8	Potom.008G22699-SNP108	12	Potom.012G28819-SNP27	Holocellulose content	AA	1.32E+01	3.75E-03
1	Potom.001G01788-SNP70	16	Potom.016G33876-SNP25	Stem volume	DA	-8.94E-01	3.74E-03
2	Potom.002G05522-SNP23	8	Potom.008G22699-SNP72	Stem volume	DD	6.77E-01	3.73E-03
5	Pto-Wuschela-SNP5	8	Potom.008G22699-SNP72	Holocellulose content	DD	-2.24E+01	3.73E-03
5	Pto-Wuschela-SNP14	5	Potom.005G13176-SNP47	Holocellulose content	DA	-1.13E+01	3.73E-03
5	Potom.005G13966-SNP89	8	Potom.008G22699-SNP9	Stem volume	DD	-1.18E+00	3.73E-03
8	Potom.008G22699-SNP74	16	Potom.016G33891-SNP15	α-cellulose content	DD	1.83E+01	3.73E-03
10	Potom.010G24967-SNP43	14	Potom.014G31981-SNP25	Stem volume	DA	-5.52E-01	3.73E-03
10	Potom.010G25706-SNP89	13	Potom.013G29538-SNP9	α-cellulose content	DA	-1.16E+01	3.72E-03
13	Potom.013G29538-SNP4	20	Potom.003G08321-SNP13	Holocellulose content	DA	1.81E+01	3.72E-03
2	Potom.002G05522-SNP60	10	Potom.010G24967-SNP45	Fiber length	AD	-8.08E-02	3.71E-03
5	Pto-Wuschela-SNP14	10	Potom.010G25700-SNP5	Holocellulose content	DA	-1.59E+01	3.71E-03
5	Potom.005G13333-SNP13	13	Potom.013G29538-SNP2	Holocellulose content	AA	6.71E+00	3.70E-03
13	Potom.013G29538-SNP71	20	Potom.003G08321-SNP7	α-cellulose content	AA	-7.16E+00	3.70E-03
1	Potom.001G00363-SNP26	5	Potom.005G13176-SNP4	Tree height	AD	5.55E+00	3.69E-03
2	Potom.002G05769-SNP57	2	Potom.002G07963-SNP229	Diameter at breast height	AA	-8.96E+00	3.69E-03
8	Potom.008G22699-SNP22	13	Potom.013G29538-SNP2	Fiber length	DD	1.63E-01	3.68E-03
1	Potom.001G03193-SNP20	2	Potom.002G05522-SNP23	Stem volume	DA	-4.70E-01	3.66E-03
2	Potom.002G07963-SNP231	5	Potom.005G13176-SNP2	Diameter at breast height	AA	-8.44E+00	3.66E-03
1	Potom.001G03193-SNP26	16	Potom.016G33876-SNP74	Lignin content	DA	-2.88E+00	3.65E-03
2	Potom.002G05769-SNP25	16	Potom.016G33876-SNP74	Fiber width	DA	-1.78E+00	3.65E-03
2	Potom.002G07337-SNP15	5	Potom.005G13333-SNP13	Holocellulose content	AA	7.99E+00	3.65E-03
13	Potom.013G29538-SNP15	16	Potom.016G33891-SNP26	Microfiber angle	AA	-2.97E+00	3.65E-03
20	Potom.003G08321-SNP11	20	Potom.003G08321-SNP20	Stem volume	DD	-9.51E-01	3.64E-03

2	Potom.002G05522-SNP62	10	Potom.010G24967-SNP67	Diameter at breast height	DA	-4.89E+00	3.63E-03
1	Potom.001G01788-SNP70	16	Potom.016G33876-SNP108	Microfiber angle	AA	2.24E+00	3.62E-03
2	Potom.002G05522-SNP15	13	Potom.013G29538-SNP9	Stem volume	AD	-5.90E-01	3.62E-03
5	Pto-Wuschela-SNP12	10	Potom.010G25398-SNP65	Hemicellulose content	DA	-1.45E+01	3.62E-03
5	Potom.005G13176-SNP16	8	Potom.008G22699-SNP108	Fiber width	DD	-4.06E+00	3.61E-03
5	Potom.005G13176-SNP44	8	Potom.008G22699-SNP74	Stem volume	AA	2.36E-01	3.61E-03
5	Potom.005G13333-SNP13	13	Potom.013G29538-SNP41	Hemicellulose content	DD	-2.04E+01	3.61E-03
8	Potom.008G22699-SNP108	16	Potom.016G33891-SNP26	Fiber length	DA	9.94E-02	3.61E-03
13	Potom.013G29538-SNP16	16	Potom.016G33891-SNP26	Microfiber angle	AA	-2.91E+00	3.61E-03
16	Potom.016G33876-SNP31	20	Potom.003G09473-SNP2	Fiber length	DD	1.50E-01	3.61E-03
1	Potom.001G03606-SNP29	10	Potom.010G25398-SNP68	α-cellulose content	DD	3.29E+01	3.60E-03
2	Potom.002G05522-SNP4	2	Potom.002G05522-SNP23	Holocellulose content	AD	8.75E+00	3.60E-03
10	Potom.010G25706-SNP89	20	Potom.003G08321-SNP20	Stem volume	DA	4.50E-01	3.59E-03
8	Potom.008G21952-SNP16	13	Potom.013G29538-SNP72	Diameter at breast height	AD	-7.89E+00	3.58E-03
2	Potom.002G05522-SNP62	20	Potom.003G08321-SNP32	Diameter at breast height	AA	3.74E+00	3.57E-03
2	Potom.002G05522-SNP60	8	Potom.008G21952-SNP16	Holocellulose content	AD	-1.33E+01	3.56E-03
2	Potom.002G05522-SNP62	8	Potom.008G21952-SNP16	Holocellulose content	AD	-1.33E+01	3.56E-03
2	Potom.002G05522-SNP1	20	Potom.003G09473-SNP13	Hemicellulose content	DA	-1.80E+01	3.55E-03
5	Pto-Wuschela-SNP14	9	Potom.009G23029-SNP3	Holocellulose content	DA	-1.05E+01	3.55E-03
5	Potom.005G13176-SNP26	20	Potom.003G09473-SNP13	α-cellulose content	DD	-2.78E+01	3.55E-03
8	Potom.008G22699-SNP71	13	Potom.013G29538-SNP72	Stem volume	DD	-1.12E+00	3.55E-03
1	Potom.001G03353-SNP24	8	Potom.008G22699-SNP22	Lignin content	DD	-8.21E+00	3.54E-03
2	Potom.002G07963-SNP72	20	Potom.003G09473-SNP7	Diameter at breast height	DD	1.57E+01	3.54E-03
5	Potom.005G13176-SNP2	20	Potom.003G09473-SNP2	Fiber length	AD	1.61E-01	3.54E-03
8	Potom.008G22699-SNP22	20	Potom.003G09473-SNP11	Stem volume	DD	1.07E+00	3.54E-03
1	Potom.001G00363-SNP21	2	Potom.002G07337-SNP15	Hemicellulose content	AD	1.48E+01	3.53E-03

5	Potom.005G13966-SNP74	10	Potom.010G24967-SNP45	Fiber length	AA	-6.37E-02	3.53E-03
2	Potom.002G07337-SNP56	20	Potom.003G08321-SNP9	Holocellulose content	AA	1.55E+01	3.51E-03
1	Potom.001G03193-SNP20	8	Potom.008G22699-SNP72	Holocellulose content	AD	-1.57E+01	3.50E-03
1	Potom.001G03193-SNP30	13	Potom.013G29538-SNP14	Stem volume	AA	-5.60E-01	3.50E-03
2	Potom.002G05522-SNP60	10	Potom.010G24967-SNP67	Diameter at breast height	DA	-4.92E+00	3.50E-03
2	Potom.002G07337-SNP229	20	Potom.003G09473-SNP11	Fiber length	AA	-7.30E-02	3.50E-03
1	Potom.001G03193-SNP31	5	Potom.005G13333-SNP12	Stem volume	AA	-4.59E-01	3.49E-03
2	Potom.002G07963-SNP170	20	Potom.003G09473-SNP7	Holocellulose content	AA	5.28E+00	3.49E-03
5	Potom.005G13333-SNP12	14	Potom.014G31981-SNP25	Microfiber angle	DA	-5.81E+00	3.49E-03
8	Potom.008G22699-SNP38	20	Potom.003G08321-SNP3	Stem volume	DA	-8.63E-01	3.49E-03
2	Potom.002G05522-SNP1	20	Potom.003G08321-SNP10	Hemicellulose content	DA	1.51E+01	3.48E-03
8	Potom.008G22699-SNP72	16	Potom.016G33891-SNP29	Diameter at breast height	AA	-8.41E+00	3.48E-03
2	Potom.002G05769-SNP43	20	Potom.003G08321-SNP32	Hemicellulose content	AD	-1.39E+01	3.47E-03
1	Potom.001G03606-SNP26	8	Potom.008G22699-SNP38	Stem volume	AA	-6.32E-01	3.46E-03
1	Potom.001G03606-SNP27	10	Potom.010G25398-SNP5	Holocellulose content	AA	8.62E+00	3.46E-03
2	Potom.002G05522-SNP73	10	Potom.010G24967-SNP40	Hemicellulose content	AA	-9.33E+00	3.46E-03
2	Potom.002G05522-SNP60	20	Potom.003G08321-SNP18	Fiber width	DA	1.80E+00	3.45E-03
8	Potom.008G21952-SNP16	8	Potom.008G22699-SNP150	Fiber width	AD	3.77E+00	3.45E-03
13	Potom.013G29538-SNP2	20	Potom.003G09473-SNP2	α-cellulose content	AA	-4.53E+00	3.45E-03
13	Potom.013G29538-SNP4	20	Potom.003G09473-SNP2	Fiber length	DD	1.50E-01	3.45E-03
13	Potom.013G29538-SNP7	20	Potom.003G08321-SNP18	Stem volume	AD	5.84E-01	3.45E-03
20	Potom.003G08321-SNP4	20	Potom.003G09473-SNP2	Stem volume	DA	1.09E+00	3.45E-03
1	Potom.001G00363-SNP23	2	Potom.002G07337-SNP15	Holocellulose content	AD	1.81E+01	3.44E-03
5	Pto-Wuschela-SNP12	16	Potom.016G33891-SNP26	Holocellulose content	DA	-1.15E+01	3.44E-03
2	Potom.002G05522-SNP12	10	Potom.010G24967-SNP67	Fiber width	AA	-9.48E-01	3.43E-03
5	Potom.005G13333-SNP18	8	Potom.008G22699-SNP70	Microfiber angle	DD	8.77E+00	3.43E-03

1	Potom.001G03193-SNP26	16	Potom.016G33876-SNP74	Diameter at breast height	AD	-9.51E+00	3.42E-03
5	Potom.005G13176-SNP15	12	Potom.012G28409-SNP11	Hemicellulose content	DA	1.67E+01	3.41E-03
10	Potom.010G25294-SNP173	13	Potom.013G29538-SNP135	Fiber width	AD	3.06E+00	3.41E-03
2	Potom.002G05522-SNP15	20	Potom.003G08321-SNP38	Stem volume	AA	-6.86E-01	3.40E-03
2	Potom.002G07337-SNP34	16	Potom.016G33891-SNP17	Lignin content	AA	-1.55E+00	3.40E-03
10	Potom.010G24967-SNP67	16	Potom.016G33876-SNP25	Diameter at breast height	AA	-8.83E+00	3.40E-03
2	Potom.002G05522-SNP12	5	Pto-Wuschela-SNP10	Fiber width	DA	-1.55E+00	3.39E-03
2	Potom.002G05769-SNP13	10	Potom.010G24967-SNP45	Fiber length	AA	-9.78E-02	3.39E-03
14	Potom.014G31981-SNP25	16	Potom.016G34352-SNP98	Holocellulose content	AA	7.44E+00	3.39E-03
2	Potom.002G05769-SNP57	20	Potom.003G08321-SNP33	Stem volume	AA	-5.61E-01	3.38E-03
10	Potom.010G24967-SNP43	20	Potom.003G08321-SNP10	Fiber length	DA	6.58E-02	3.37E-03
2	Potom.002G05769-SNP72	13	Potom.013G29538-SNP71	Fiber width	AD	-3.61E+00	3.36E-03
5	Pto-Wuschela-SNP10	8	Potom.008G22699-SNP72	Hemicellulose content	AD	9.28E+00	3.36E-03
5	Pto-Wuschela-SNP14	20	Potom.003G08321-SNP3	Lignin content	DA	-3.52E+00	3.36E-03
10	Potom.010G24967-SNP27	16	Potom.016G33891-SNP28	Diameter at breast height	AD	-8.94E+00	3.36E-03
5	Potom.005G13966-SNP97	13	Potom.013G29538-SNP7	Stem volume	AD	5.56E-01	3.35E-03
2	Potom.002G05522-SNP1	5	Potom.005G13176-SNP68	Stem volume	AA	-5.58E-01	3.34E-03
2	Potom.002G07337-SNP34	2	Potom.002G07963-SNP163	Fiber length	DD	-1.84E-01	3.34E-03
2	Potom.002G05522-SNP12	2	Potom.002G05522-SNP54	Fiber width	AD	2.36E+00	3.33E-03
2	Potom.002G07337-SNP15	8	Potom.008G22699-SNP9	Microfiber angle	DD	-9.73E+00	3.33E-03
1	Potom.001G03193-SNP27	8	Potom.008G22699-SNP108	Fiber length	AD	1.39E-01	3.32E-03
2	Potom.002G07337-SNP15	5	Potom.005G13333-SNP13	α-cellulose content	AA	7.43E+00	3.32E-03
5	Pto-Wuschela-SNP5	10	Potom.010G25398-SNP26	Holocellulose content	AD	-1.16E+01	3.30E-03
5	Pto-Wuschela-SNP5	20	Potom.003G08321-SNP3	Hemicellulose content	DD	-1.52E+01	3.30E-03
5	Potom.005G13333-SNP12	16	Potom.016G33891-SNP15	Stem volume	DA	6.96E-01	3.29E-03
13	Potom.013G29538-SNP9	20	Potom.003G08321-SNP18	Fiber width	DA	2.13E+00	3.29E-03

16	Potom.016G33876-SNP31	20	Potom.003G08321-SNP10	Diameter at breast height	AD	-6.06E+00	3.29E-03
16	Potom.016G33891-SNP28	20	Potom.003G09473-SNP2	Stem volume	AA	3.90E-01	3.29E-03
2	Potom.002G05769-SNP13	5	Potom.005G13176-SNP27	Diameter at breast height	AA	-9.22E+00	3.28E-03
5	Pto-Wuschela-SNP14	14	Potom.014G31981-SNP25	α-cellulose content	DD	-1.91E+01	3.28E-03
8	Potom.008G22699-SNP108	20	Potom.003G08321-SNP17	α-cellulose content	AA	-4.52E+00	3.28E-03
1	Potom.001G03193-SNP20	2	Potom.002G05522-SNP62	Stem volume	DA	-4.74E-01	3.27E-03
8	Potom.008G22699-SNP108	12	Potom.012G28819-SNP9	Fiber length	DA	8.42E-02	3.27E-03
10	Potom.010G25398-SNP68	13	Potom.013G29538-SNP5	Holocellulose content	DA	2.20E+01	3.27E-03
13	Potom.013G29538-SNP15	16	Potom.016G33891-SNP17	Hemicellulose content	AA	-7.19E+00	3.27E-03
2	Potom.002G05522-SNP4	10	Potom.010G24967-SNP40	Lignin content	AD	-2.59E+00	3.26E-03
2	Potom.002G05769-SNP70	2	Potom.002G07963-SNP163	Fiber length	AD	-1.26E-01	3.26E-03
2	Potom.002G07206-SNP24	12	Potom.012G28819-SNP27	Microfiber angle	DD	1.16E+01	3.26E-03
2	Potom.002G07337-SNP34	5	Potom.005G13176-SNP26	Hemicellulose content	AA	-4.60E+00	3.26E-03
5	Pto-Wuschela-SNP5	5	Potom.005G13176-SNP41	Fiber width	AD	-1.90E+00	3.26E-03
5	Potom.005G13176-SNP16	8	Potom.008G22699-SNP74	α-cellulose content	DD	1.44E+01	3.26E-03
5	Potom.005G13176-SNP26	20	Potom.003G08321-SNP32	Hemicellulose content	DD	-1.96E+01	3.26E-03
5	Potom.005G13176-SNP62	8	Potom.008G22699-SNP22	Stem volume	DD	-7.91E-01	3.26E-03
1	Potom.001G03606-SNP27	8	Potom.008G22699-SNP3	Lignin content	AA	-2.28E+00	3.24E-03
1	Potom.001G03606-SNP27	8	Potom.008G22699-SNP3	Diameter at breast height	AD	-7.96E+00	3.23E-03
16	Potom.016G33876-SNP108	16	Potom.016G33891-SNP28	Tree height	AA	-2.60E+00	3.23E-03
1	Potom.001G03606-SNP29	16	Potom.016G34352-SNP98	Holocellulose content	AA	1.23E+01	3.22E-03
2	Potom.002G05522-SNP62	20	Potom.003G08321-SNP18	Fiber width	DA	1.82E+00	3.22E-03
5	Potom.005G13176-SNP15	14	Potom.014G31981-SNP25	Hemicellulose content	DA	-1.64E+01	3.22E-03
13	Potom.013G29538-SNP2	20	Potom.003G08321-SNP13	Stem volume	DD	-1.30E+00	3.22E-03
1	Potom.001G00363-SNP23	14	Potom.014G31981-SNP6	Diameter at breast height	AD	-1.12E+01	3.21E-03
2	Potom.002G05769-SNP57	8	Potom.008G21952-SNP16	Stem volume	AA	-4.98E-01	3.21E-03

2	Potom.002G07337-SNP15	5	Potom.005G13176-SNP3	α-cellulose content	AD	1.31E+01	3.21E-03
20	Potom.003G08321-SNP9	20	Potom.003G08321-SNP20	α-cellulose content	DD	2.20E+01	3.20E-03
2	Potom.002G07963-SNP163	5	Potom.005G13985-SNP6	Fiber length	AD	2.07E-01	3.19E-03
5	Potom.005G13985-SNP20	16	Potom.016G33876-SNP25	Fiber length	DD	-2.76E-01	3.19E-03
13	Potom.013G29538-SNP72	16	Potom.016G34352-SNP109	Fiber width	DD	3.90E+00	3.19E-03
5	Potom.005G13176-SNP115	8	Potom.008G21326-SNP69	Diameter at breast height	AA	-5.06E+00	3.18E-03
5	Potom.005G13985-SNP5	20	Potom.003G09473-SNP2	Microfiber angle	DA	7.81E+00	3.18E-03
8	Potom.008G22699-SNP100	20	Potom.003G08321-SNP3	Stem volume	AD	3.83E-01	3.18E-03
1	Potom.001G00363-SNP19	2	Potom.002G05769-SNP57	Tree height	AA	-4.10E+00	3.17E-03
2	Potom.002G05769-SNP43	2	Potom.002G07963-SNP185	Hemicellulose content	AD	-1.70E+01	3.17E-03
2	Potom.002G05522-SNP1	13	Potom.013G29538-SNP5	Holocellulose content	DD	-3.26E+01	3.15E-03
2	Potom.002G05522-SNP12	5	Pto-Wuschela-SNP5	Holocellulose content	AA	6.82E+00	3.15E-03
2	Potom.002G07337-SNP16	8	Potom.008G22699-SNP108	Fiber length	AD	8.19E-02	3.15E-03
1	Potom.001G03193-SNP10	5	Potom.005G13176-SNP62	α-cellulose content	DD	1.89E+01	3.14E-03
2	Potom.002G05522-SNP12	2	Potom.002G05522-SNP54	Diameter at breast height	AA	-5.62E+00	3.14E-03
2	Potom.002G05769-SNP25	16	Potom.016G33876-SNP73	Fiber width	DA	-1.81E+00	3.14E-03
2	Potom.002G05769-SNP57	8	Potom.008G21326-SNP20	Microfiber angle	AD	-8.13E+00	3.14E-03
2	Potom.002G07206-SNP24	20	Potom.003G08321-SNP17	Lignin content	AA	-1.50E+00	3.14E-03
1	Potom.001G03193-SNP10	12	Potom.012G28819-SNP46	Hemicellulose content	DD	-2.00E+01	3.12E-03
2	Potom.002G05522-SNP15	13	Potom.013G29538-SNP5	Diameter at breast height	AD	-6.79E+00	3.12E-03
2	Potom.002G05769-SNP43	2	Potom.002G07963-SNP185	Diameter at breast height	DA	-4.23E+00	3.12E-03
2	Potom.002G05769-SNP72	2	Potom.002G07963-SNP163	Fiber width	DA	2.32E+00	3.12E-03
5	Pto-Wuschela-SNP14	14	Potom.014G31981-SNP26	α-cellulose content	DD	-1.93E+01	3.11E-03
8	Potom.008G21326-SNP69	16	Potom.016G33891-SNP26	Diameter at breast height	AA	-4.81E+00	3.11E-03
10	Potom.010G25398-SNP26	20	Potom.003G08321-SNP10	Stem volume	DD	-8.94E-01	3.11E-03
13	Potom.013G29538-SNP71	14	Potom.014G31981-SNP25	Fiber width	DA	-3.44E+00	3.11E-03

14	Potom.014G31981-SNP57	20	Potom.003G08321-SNP17	Tree height	AD	1.58E+00	3.11E-03
1	Potom.001G00363-SNP19	2	Potom.002G07206-SNP24	Holocellulose content	AA	9.17E+00	3.10E-03
5	Potom.005G13333-SNP13	5	Potom.005G13985-SNP5	Fiber length	AD	1.74E-01	3.10E-03
5	Potom.005G13985-SNP20	20	Potom.003G08321-SNP2	Holocellulose content	DD	-3.41E+01	3.10E-03
1	Potom.001G03193-SNP26	20	Potom.003G09473-SNP2	Holocellulose content	DA	1.33E+01	3.09E-03
2	Potom.002G05522-SNP23	20	Potom.003G10089-SNP7	Microfiber angle	AD	-9.41E+00	3.09E-03
5	Pto-Wuschela-SNP12	16	Potom.016G33891-SNP26	Hemicellulose content	DA	-1.01E+01	3.09E-03
10	Potom.010G24967-SNP43	20	Potom.003G08321-SNP10	Diameter at breast height	DD	-1.11E+01	3.09E-03
10	Potom.010G24967-SNP45	20	Potom.003G09473-SNP11	Hemicellulose content	AA	-8.81E+00	3.09E-03
1	Potom.001G00363-SNP23	10	Potom.010G25398-SNP26	Stem volume	AD	-7.39E-01	3.08E-03
1	Potom.001G03193-SNP31	2	Potom.002G05522-SNP23	Stem volume	DD	-1.11E+00	3.08E-03
2	Potom.002G05522-SNP23	16	Potom.016G34352-SNP22	Stem volume	AA	-3.53E-01	3.08E-03
1	Potom.001G01788-SNP70	2	Potom.002G05769-SNP43	Fiber length	AA	-8.01E-02	3.07E-03
2	Potom.002G05522-SNP23	16	Potom.016G33876-SNP53	Stem volume	DA	3.53E-01	3.07E-03
5	Potom.005G13176-SNP44	10	Potom.010G24967-SNP43	Diameter at breast height	AA	-3.96E+00	3.07E-03
10	Potom.010G24967-SNP27	16	Potom.016G33891-SNP28	Stem volume	AA	-3.84E-01	3.07E-03
2	Potom.002G05632-SNP15	13	Potom.013G29538-SNP71	Fiber width	AD	-3.08E+00	3.06E-03
9	Potom.009G23029-SNP3	13	Potom.013G29538-SNP4	Stem volume	AA	-3.00E-01	3.06E-03
16	Potom.016G33891-SNP14	20	Potom.003G09473-SNP2	Fiber width	DD	8.13E+00	3.06E-03
5	Pto-Wuschela-SNP14	14	Potom.014G31981-SNP26	Hemicellulose content	AA	4.51E+00	3.05E-03
10	Potom.010G24967-SNP45	14	Potom.014G31981-SNP25	Stem volume	DA	-5.60E-01	3.04E-03
10	Potom.010G25706-SNP89	13	Potom.013G29538-SNP7	α-cellulose content	AD	-1.09E+01	3.04E-03
1	Potom.001G03606-SNP27	8	Potom.008G22699-SNP71	Stem volume	AD	6.86E-01	3.03E-03
8	Potom.008G22699-SNP108	18	Potom.018G35976-SNP25	Fiber length	AD	1.08E-01	3.03E-03
2	Potom.002G05522-SNP1	5	Pto-Wuschela-SNP5	Holocellulose content	DA	-1.67E+01	3.02E-03
13	Potom.013G29538-SNP4	20	Potom.003G08321-SNP13	Stem volume	DD	-1.34E+00	3.01E-03

18	Potom.018G35976-SNP25	20	Potom.003G08321-SNP5	Fiber length	AA	-5.82E-02	3.01E-03
2	Potom.002G05522-SNP12	12	Potom.012G28819-SNP27	Fiber length	DD	2.14E-01	3.00E-03
2	Potom.002G05769-SNP25	14	Potom.014G31981-SNP26	Fiber width	DD	4.92E+00	3.00E-03
5	Potom.005G13985-SNP17	8	Potom.008G22699-SNP38	Stem volume	DA	1.06E+00	2.99E-03
1	Potom.001G03193-SNP13	8	Potom.008G21952-SNP16	Stem volume	DA	6.99E-01	2.98E-03
2	Potom.002G07206-SNP24	10	Potom.010G25706-SNP89	Stem volume	AD	-4.69E-01	2.98E-03
13	Potom.013G29538-SNP9	20	Potom.003G08321-SNP14	Stem volume	DD	-1.37E+00	2.98E-03
5	Potom.005G13176-SNP26	20	Potom.003G08321-SNP32	α-cellulose content	AD	1.29E+01	2.97E-03
2	Potom.002G05522-SNP62	10	Potom.010G24967-SNP43	Fiber length	AD	-8.33E-02	2.96E-03
5	Pto-Wuschela-SNP12	20	Potom.003G08321-SNP6	Hemicellulose content	DA	1.18E+01	2.96E-03
10	Potom.010G25700-SNP13	13	Potom.013G29538-SNP71	Fiber width	AA	2.65E+00	2.96E-03
13	Potom.013G29538-SNP9	20	Potom.003G08321-SNP13	Holocellulose content	AA	1.43E+01	2.96E-03
1	Potom.001G03606-SNP26	2	Potom.002G05522-SNP60	α-cellulose content	DD	2.29E+01	2.95E-03
2	Potom.002G07963-SNP72	5	Potom.005G13176-SNP44	Stem volume	AA	-2.38E-01	2.95E-03
2	Potom.002G07963-SNP72	20	Potom.003G09473-SNP7	Stem volume	DD	9.87E-01	2.95E-03
8	Potom.008G22699-SNP38	20	Potom.003G08321-SNP20	Stem volume	AA	4.03E-01	2.95E-03
1	Potom.001G03193-SNP30	2	Potom.002G05522-SNP60	Tree height	AD	-4.65E+00	2.94E-03
2	Potom.002G05769-SNP57	8	Potom.008G21952-SNP16	Diameter at breast height	AA	-8.15E+00	2.94E-03
5	Pto-Wuschela-SNP5	8	Potom.008G22699-SNP68	Fiber width	AA	-1.80E+00	2.94E-03
5	Potom.005G13333-SNP12	20	Potom.003G08321-SNP9	Lignin content	DD	-8.49E+00	2.94E-03
1	Potom.001G03193-SNP20	10	Potom.010G25398-SNP26	Diameter at breast height	AD	-7.97E+00	2.93E-03
8	Potom.008G22516-SNP49	10	Potom.010G25700-SNP19	Microfiber angle	AD	-1.04E+01	2.93E-03
1	Potom.001G00363-SNP21	2	Potom.002G07337-SNP15	Holocellulose content	AD	1.83E+01	2.92E-03
2	Potom.002G05522-SNP73	20	Potom.003G08321-SNP3	Lignin content	AD	1.66E+00	2.92E-03
2	Potom.002G05769-SNP57	20	Potom.003G08321-SNP33	Diameter at breast height	AA	-8.90E+00	2.92E-03
5	Pto-Wuschela-SNP14	14	Potom.014G31981-SNP25	Hemicellulose content	AA	5.16E+00	2.92E-03

2	Potom.002G07337-SNP15	10	Potom.010G24967-SNP67	Stem volume	DA	-2.17E-01	2.91E-03
5	Potom.005G13176-SNP26	13	Potom.013G29538-SNP71	Hemicellulose content	DD	-2.25E+01	2.91E-03
2	Potom.002G05522-SNP15	8	Potom.008G22699-SNP108	Tree height	AD	3.70E+00	2.90E-03
5	Pto-Wuschela-SNP12	20	Potom.003G08321-SNP17	Hemicellulose content	DD	-1.53E+01	2.90E-03
5	Potom.005G13966-SNP89	20	Potom.003G10089-SNP11	Stem volume	DD	-1.27E+00	2.90E-03
2	Potom.002G05769-SNP13	8	Potom.008G22699-SNP72	Hemicellulose content	DA	1.47E+01	2.89E-03
1	Potom.001G03193-SNP20	2	Potom.002G05522-SNP60	Stem volume	DA	-4.80E-01	2.88E-03
10	Potom.010G24967-SNP43	20	Potom.003G09473-SNP11	Hemicellulose content	AA	-8.88E+00	2.88E-03
1	Potom.001G03606-SNP26	10	Potom.010G25398-SNP68	Stem volume	AD	8.75E-01	2.87E-03
2	Potom.002G07337-SNP15	10	Potom.010G25398-SNP68	Holocellulose content	DA	1.93E+01	2.87E-03
8	Potom.008G22699-SNP22	10	Potom.010G25706-SNP89	Stem volume	DD	-8.99E-01	2.87E-03
12	Potom.012G28819-SNP8	16	Potom.016G33891-SNP15	Diameter at breast height	AA	-8.55E+00	2.87E-03
1	Potom.001G03193-SNP26	2	Potom.002G05632-SNP14	Stem volume	DA	-4.65E-01	2.85E-03
1	Potom.001G03606-SNP26	2	Potom.002G05522-SNP62	α-cellulose content	DD	2.30E+01	2.85E-03
5	Potom.005G13176-SNP47	20	Potom.003G08321-SNP18	Hemicellulose content	AD	1.10E+01	2.85E-03
8	Potom.008G22699-SNP38	8	Potom.008G22699-SNP74	Stem volume	DA	5.81E-01	2.85E-03
2	Potom.002G05522-SNP13	14	Potom.014G31981-SNP26	Lignin content	AA	3.82E+00	2.84E-03
2	Potom.002G05522-SNP14	14	Potom.014G31981-SNP26	Lignin content	AA	3.82E+00	2.84E-03
10	Potom.010G25398-SNP26	20	Potom.003G08321-SNP10	Microfiber angle	DA	-4.32E+00	2.84E-03
16	Potom.016G34352-SNP108	20	Potom.003G08321-SNP17	α-cellulose content	DD	1.50E+01	2.84E-03
5	Potom.005G13176-SNP3	10	Potom.010G25706-SNP89	Stem volume	DD	-8.33E-01	2.83E-03
5	Potom.005G13966-SNP90	8	Potom.008G22699-SNP9	Stem volume	DA	-9.76E-01	2.83E-03
14	Potom.014G31981-SNP25	16	Potom.016G34352-SNP98	α-cellulose content	AA	6.85E+00	2.83E-03
16	Potom.016G34352-SNP45	20	Potom.003G09473-SNP12	Holocellulose content	DA	-2.26E+01	2.83E-03
1	Potom.001G03193-SNP26	16	Potom.016G33876-SNP71	Lignin content	DA	-2.89E+00	2.82E-03
2	Potom.002G05522-SNP12	8	Potom.008G22699-SNP108	Tree height	AD	3.58E+00	2.82E-03

5	Potom.005G13176-SNP44	16	Potom.016G33876-SNP74	Microfiber angle	AA	2.29E+00	2.82E-03
5	Potom.005G13176-SNP168	10	Potom.010G25706-SNP89	Hemicellulose content	DD	1.81E+01	2.81E-03
8	Potom.008G22699-SNP38	8	Potom.008G22699-SNP98	Holocellulose content	AD	-2.66E+01	2.81E-03
8	Potom.008G22699-SNP72	20	Potom.003G08321-SNP17	Diameter at breast height	DD	1.20E+01	2.81E-03
10	Potom.010G25398-SNP26	20	Potom.003G09473-SNP2	Stem volume	DA	5.27E-01	2.81E-03
14	Potom.014G31981-SNP57	20	Potom.003G08321-SNP17	Diameter at breast height	AD	3.60E+00	2.81E-03
16	Potom.016G33876-SNP94	20	Potom.003G08321-SNP9	Holocellulose content	DA	1.63E+01	2.81E-03
5	Potom.005G13985-SNP17	8	Potom.008G22699-SNP38	Stem volume	AA	-6.75E-01	2.80E-03
20	Potom.003G08321-SNP3	20	Potom.003G08321-SNP21	Holocellulose content	DD	2.10E+01	2.80E-03
16	Potom.016G33876-SNP31	20	Potom.003G08321-SNP10	Stem volume	AD	-4.24E-01	2.79E-03
2	Potom.002G07337-SNP15	2	Potom.002G07963-SNP153	Tree height	DA	-4.75E+00	2.78E-03
5	Potom.005G13176-SNP47	8	Potom.008G22699-SNP108	Fiber length	AD	7.61E-02	2.77E-03
10	Potom.010G25398-SNP63	13	Potom.013G29538-SNP15	Microfiber angle	AA	-4.33E+00	2.77E-03
12	Potom.012G28819-SNP45	20	Potom.003G08321-SNP7	Stem volume	AA	5.59E-01	2.77E-03
20	Potom.003G08321-SNP7	20	Potom.003G08321-SNP21	Holocellulose content	AD	-1.65E+01	2.77E-03
1	Potom.001G03193-SNP30	5	Potom.005G13333-SNP12	Tree height	AA	-3.60E+00	2.76E-03
8	Potom.008G22699-SNP100	10	Potom.010G25700-SNP5	Microfiber angle	AD	5.28E+00	2.76E-03
1	Potom.001G03353-SNP164	2	Potom.002G07337-SNP34	Lignin content	AA	-1.08E+00	2.75E-03
1	Potom.001G03193-SNP26	16	Potom.016G33876-SNP71	Diameter at breast height	AD	-9.67E+00	2.74E-03
14	Potom.014G31981-SNP26	20	Potom.003G08321-SNP11	Stem volume	AD	-6.01E-01	2.74E-03
1	Potom.001G03193-SNP27	5	Potom.005G13176-SNP162	Holocellulose content	AD	1.42E+01	2.73E-03
2	Potom.002G05522-SNP12	2	Potom.002G05522-SNP62	Stem volume	AD	-4.26E-01	2.73E-03
2	Potom.002G05632-SNP14	5	Potom.005G13176-SNP16	Diameter at breast height	AA	-2.84E+00	2.72E-03
2	Potom.002G07963-SNP185	16	Potom.016G33891-SNP6	Diameter at breast height	AA	-3.36E+00	2.72E-03
5	Potom.005G13176-SNP44	10	Potom.010G24967-SNP45	Diameter at breast height	AA	-4.17E+00	2.72E-03
5	Potom.005G13333-SNP13	13	Potom.013G29538-SNP4	Holocellulose content	DD	-1.55E+01	2.72E-03

5	Potom.005G13985-SNP1	20	Potom.003G09473-SNP2	Fiber length	DD	2.01E-01	2.72E-03
5	Potom.005G13985-SNP2	20	Potom.003G09473-SNP2	Fiber length	DD	2.01E-01	2.72E-03
13	Potom.013G29538-SNP72	16	Potom.016G33876-SNP108	Stem volume	DA	-2.53E-01	2.72E-03
2	Potom.002G05522-SNP54	2	Potom.002G07206-SNP24	Stem volume	DA	-4.23E-01	2.71E-03
2	Potom.002G05769-SNP43	5	Potom.005G13333-SNP12	Fiber length	AD	1.36E-01	2.71E-03
5	Potom.005G13176-SNP2	14	Potom.014G31981-SNP26	α-cellulose content	DA	-1.74E+01	2.71E-03
10	Potom.010G24967-SNP45	20	Potom.003G08321-SNP10	Diameter at breast height	DD	-1.11E+01	2.71E-03
10	Potom.010G25398-SNP26	20	Potom.003G08321-SNP10	Holocellulose content	DA	1.01E+01	2.71E-03
14	Potom.014G31981-SNP25	20	Potom.003G09473-SNP11	Diameter at breast height	AA	-8.40E+00	2.71E-03
2	Potom.002G07337-SNP229	5	Potom.005G13176-SNP2	Stem volume	AD	4.83E-01	2.70E-03
5	Pto-Wuschela-SNP14	20	Potom.003G08321-SNP33	Lignin content	DA	4.16E+00	2.70E-03
5	Potom.005G13176-SNP27	20	Potom.003G08321-SNP13	α-cellulose content	DD	3.56E+01	2.70E-03
5	Potom.005G13176-SNP44	8	Potom.008G22699-SNP108	Fiber length	AD	8.60E-02	2.70E-03
8	Potom.008G22699-SNP98	20	Potom.003G08321-SNP17	Stem volume	DA	-7.56E-01	2.70E-03
5	Potom.005G13985-SNP17	10	Potom.010G24967-SNP58	Fiber length	AA	-1.76E-01	2.68E-03
2	Potom.002G05522-SNP4	2	Potom.002G05522-SNP12	Hemicellulose content	AD	-7.70E+00	2.67E-03
2	Potom.002G05769-SNP70	8	Potom.008G21326-SNP69	Diameter at breast height	AA	-6.54E+00	2.67E-03
5	Potom.005G13176-SNP2	20	Potom.003G09473-SNP13	Fiber length	AA	1.25E-01	2.67E-03
2	Potom.002G05522-SNP12	13	Potom.013G29538-SNP9	Stem volume	AD	-6.13E-01	2.66E-03
2	Potom.002G05522-SNP23	20	Potom.003G08321-SNP10	Diameter at breast height	DD	-1.00E+01	2.66E-03
2	Potom.002G07337-SNP144	8	Potom.008G21326-SNP69	Diameter at breast height	DD	-1.16E+01	2.66E-03
2	Potom.002G07337-SNP145	8	Potom.008G21326-SNP69	Diameter at breast height	DD	-1.16E+01	2.66E-03
13	Potom.013G29538-SNP9	20	Potom.003G08321-SNP18	Stem volume	AD	6.05E-01	2.66E-03
5	Pto-Wuschela-SNP5	5	Potom.005G13333-SNP18	Holocellulose content	DA	-1.30E+01	2.65E-03
5	Pto-Wuschela-SNP10	8	Potom.008G22516-SNP43	Fiber width	AA	2.69E+00	2.65E-03
5	Potom.005G13176-SNP16	5	Potom.005G13176-SNP284	Microfiber angle	DD	-8.42E+00	2.65E-03

2	Potom.002G07337-SNP34	16	Potom.016G33891-SNP17	Hemicellulose content	DD	-1.89E+01	2.64E-03
5	Pto-Wuschela-SNP14	8	Potom.008G22699-SNP70	Hemicellulose content	DD	-2.24E+01	2.64E-03
1	Potom.001G03193-SNP20	5	Potom.005G13333-SNP12	Stem volume	DA	5.94E-01	2.63E-03
5	Potom.005G13176-SNP16	20	Potom.003G08321-SNP37	Holocellulose content	AA	-1.62E+01	2.63E-03
5	Potom.005G13333-SNP12	20	Potom.003G08321-SNP6	Fiber width	AD	3.52E+00	2.63E-03
10	Potom.010G25398-SNP63	13	Potom.013G29538-SNP16	Microfiber angle	AA	-4.32E+00	2.63E-03
2	Potom.002G05522-SNP15	2	Potom.002G07963-SNP185	Microfiber angle	AA	1.39E+00	2.62E-03
2	Potom.002G05522-SNP73	13	Potom.013G29538-SNP72	Hemicellulose content	AA	7.15E+00	2.62E-03
2	Potom.002G05769-SNP13	5	Potom.005G13176-SNP2	Hemicellulose content	DA	1.87E+01	2.62E-03
8	Potom.008G22699-SNP38	20	Potom.003G09473-SNP12	Holocellulose content	AA	1.91E+01	2.62E-03
1	Potom.001G03193-SNP20	2	Potom.002G05632-SNP14	Stem volume	DA	-3.47E-01	2.61E-03
1	Potom.001G03193-SNP20	8	Potom.008G21326-SNP69	Diameter at breast height	AD	-8.82E+00	2.61E-03
2	Potom.002G05632-SNP14	5	Potom.005G13176-SNP16	Stem volume	AA	-2.03E-01	2.60E-03
13	Potom.013G29538-SNP7	20	Potom.003G08321-SNP13	Stem volume	DD	-1.37E+00	2.60E-03
2	Potom.002G05522-SNP12	13	Potom.013G29538-SNP5	Stem volume	AD	-4.82E-01	2.59E-03
1	Potom.001G03193-SNP10	8	Potom.008G21326-SNP69	Hemicellulose content	DD	-2.01E+01	2.58E-03
1	Potom.001G03193-SNP30	2	Potom.002G05522-SNP23	Stem volume	DD	-1.14E+00	2.57E-03
5	Potom.005G13176-SNP2	14	Potom.014G31981-SNP25	α-cellulose content	DA	-1.75E+01	2.57E-03
5	Potom.005G13333-SNP12	12	Potom.012G28819-SNP9	Fiber length	DA	7.92E-02	2.57E-03
5	Potom.005G13333-SNP12	16	Potom.016G33876-SNP94	Diameter at breast height	DD	1.41E+01	2.57E-03
8	Potom.008G22699-SNP38	16	Potom.016G33891-SNP28	Stem volume	AA	5.54E-01	2.57E-03
5	Potom.005G13176-SNP47	16	Potom.016G33876-SNP71	Fiber width	DD	-4.29E+00	2.56E-03
5	Potom.005G13333-SNP18	12	Potom.012G28819-SNP9	Stem volume	DD	-6.10E-01	2.56E-03
8	Potom.008G21952-SNP16	10	Potom.010G25398-SNP65	Hemicellulose content	AA	-1.33E+01	2.56E-03
8	Potom.008G22699-SNP108	10	Potom.010G25398-SNP65	Fiber length	DA	1.12E-01	2.56E-03
16	Potom.016G33876-SNP31	20	Potom.003G08321-SNP11	Diameter at breast height	AA	6.27E+00	2.56E-03

5	Potom.005G13985-SNP4	20	Potom.003G09473-SNP2	Fiber length	DD	-3.00E-01	2.55E-03
2	Potom.002G07963-SNP147	20	Potom.003G08321-SNP20	Stem volume	DD	-1.01E+00	2.54E-03
2	Potom.002G07337-SNP15	10	Potom.010G25398-SNP63	Holocellulose content	DA	1.96E+01	2.53E-03
2	Potom.002G05522-SNP60	10	Potom.010G24967-SNP43	Fiber length	AD	-8.41E-02	2.51E-03
2	Potom.002G05769-SNP57	20	Potom.003G08321-SNP7	Diameter at breast height	AA	8.36E+00	2.51E-03
2	Potom.002G05522-SNP13	13	Potom.013G29538-SNP5	Holocellulose content	AD	1.73E+01	2.50E-03
2	Potom.002G05522-SNP14	13	Potom.013G29538-SNP5	Holocellulose content	AD	1.73E+01	2.50E-03
5	Potom.005G13176-SNP2	20	Potom.003G09473-SNP11	Fiber length	AA	1.23E-01	2.50E-03
16	Potom.016G33876-SNP108	20	Potom.003G09473-SNP7	Stem volume	AD	6.90E-01	2.50E-03
5	Pto-Wuschela-SNP12	20	Potom.003G08321-SNP7	Hemicellulose content	DA	1.19E+01	2.49E-03
10	Potom.010G25398-SNP68	13	Potom.013G29538-SNP5	Diameter at breast height	DA	-1.40E+01	2.49E-03
10	Potom.010G25700-SNP19	20	Potom.003G10089-SNP7	Fiber width	AA	-3.73E+00	2.49E-03
1	Potom.001G03193-SNP13	10	Potom.010G25398-SNP26	Stem volume	DA	-8.59E-01	2.47E-03
2	Potom.002G05522-SNP4	9	Potom.009G23029-SNP3	Holocellulose content	AD	-1.36E+01	2.47E-03
2	Potom.002G05522-SNP23	20	Potom.003G10089-SNP11	Holocellulose content	DA	1.32E+01	2.46E-03
2	Potom.002G05769-SNP25	8	Potom.008G21952-SNP16	Holocellulose content	AD	-1.77E+01	2.46E-03
5	Potom.005G13985-SNP1	8	Potom.008G22699-SNP108	Microfiber angle	DD	-9.78E+00	2.46E-03
5	Potom.005G13985-SNP2	8	Potom.008G22699-SNP108	Microfiber angle	DD	-9.78E+00	2.46E-03
5	Potom.005G13985-SNP20	8	Potom.008G22699-SNP38	Stem volume	AA	-5.91E-01	2.46E-03
8	Potom.008G22699-SNP22	13	Potom.013G29538-SNP4	Fiber length	DD	1.69E-01	2.46E-03
5	Potom.005G13176-SNP26	5	Potom.005G13333-SNP18	Hemicellulose content	DA	1.76E+01	2.45E-03
8	Potom.008G22699-SNP108	10	Potom.010G24967-SNP67	Holocellulose content	DD	1.89E+01	2.45E-03
1	Potom.001G01788-SNP70	2	Potom.002G07206-SNP24	Fiber length	DA	-9.83E-02	2.44E-03
2	Potom.002G07963-SNP185	5	Potom.005G13966-SNP74	Microfiber angle	AD	3.37E+00	2.44E-03
8	Potom.008G22699-SNP72	16	Potom.016G33891-SNP29	Stem volume	DA	-7.20E-01	2.44E-03
8	Potom.008G22699-SNP100	20	Potom.003G08321-SNP3	Fiber length	DA	9.87E-02	2.44E-03

2	Potom.002G05522-SNP15	5	Potom.005G13966-SNP89	Stem volume	AD	8.11E-01	2.43E-03
16	Potom.016G33876-SNP108	18	Potom.018G36220-SNP43	Microfiber angle	AA	3.16E+00	2.43E-03
8	Potom.008G22699-SNP73	10	Potom.010G25706-SNP79	Fiber length	DD	-1.98E-01	2.42E-03
2	Potom.002G05522-SNP13	20	Potom.003G08321-SNP4	Diameter at breast height	AD	1.55E+01	2.41E-03
2	Potom.002G05522-SNP14	20	Potom.003G08321-SNP4	Diameter at breast height	AD	1.55E+01	2.41E-03
5	Pto-Wuschela-SNP10	20	Potom.003G08321-SNP32	Hemicellulose content	DD	1.81E+01	2.41E-03
2	Potom.002G07206-SNP24	2	Potom.002G07337-SNP229	Stem volume	AD	-4.90E-01	2.40E-03
10	Potom.010G25398-SNP68	13	Potom.013G29538-SNP7	α-cellulose content	DA	1.68E+01	2.40E-03
1	Potom.001G03193-SNP26	20	Potom.003G09473-SNP2	α-cellulose content	DA	1.37E+01	2.39E-03
2	Potom.002G05522-SNP73	20	Potom.003G08321-SNP17	Holocellulose content	DD	1.61E+01	2.39E-03
2	Potom.002G05769-SNP37	2	Potom.002G07963-SNP163	Fiber length	AD	-1.57E-01	2.39E-03
8	Potom.008G21326-SNP66	16	Potom.016G33891-SNP26	Stem volume	AA	-4.36E-01	2.39E-03
8	Potom.008G21952-SNP16	20	Potom.003G09473-SNP13	Diameter at breast height	AA	-7.91E+00	2.39E-03
8	Potom.008G22699-SNP108	16	Potom.016G33891-SNP6	Fiber length	AA	-8.40E-02	2.38E-03
1	Potom.001G00363-SNP19	20	Potom.003G09473-SNP7	Stem volume	AD	9.00E-01	2.37E-03
5	Potom.005G13966-SNP90	10	Potom.010G25294-SNP212	Stem volume	DD	-1.21E+00	2.37E-03
5	Potom.005G13966-SNP89	10	Potom.010G25294-SNP212	Stem volume	DD	-1.23E+00	2.36E-03
1	Potom.001G00363-SNP21	5	Potom.005G13333-SNP12	Lignin content	DD	7.63E+00	2.35E-03
2	Potom.002G05522-SNP4	20	Potom.003G09473-SNP25	Holocellulose content	DA	-1.59E+01	2.35E-03
5	Potom.005G13176-SNP27	5	Potom.005G13966-SNP97	Hemicellulose content	AD	-1.57E+01	2.35E-03
12	Potom.012G28819-SNP8	16	Potom.016G33891-SNP26	Diameter at breast height	AA	-8.46E+00	2.35E-03
2	Potom.002G05522-SNP12	2	Potom.002G05522-SNP60	Stem volume	AD	-4.33E-01	2.32E-03
5	Potom.005G13176-SNP27	8	Potom.008G22699-SNP2	Stem volume	AA	-6.77E-01	2.32E-03
8	Potom.008G22699-SNP9	13	Potom.013G29538-SNP72	Tree height	AD	5.07E+00	2.32E-03
9	Potom.009G23029-SNP3	13	Potom.013G29538-SNP4	Holocellulose content	DD	1.98E+01	2.32E-03
2	Potom.002G05522-SNP62	10	Potom.010G25398-SNP68	Diameter at breast height	AD	-1.42E+01	2.31E-03

8	Potom.008G22699-SNP3	8	Potom.008G22699-SNP98	Holocellulose content	DD	-3.09E+01	2.31E-03
13	Potom.013G29538-SNP9	20	Potom.003G08321-SNP13	Hemicellulose content	AA	1.38E+01	2.31E-03
1	Potom.001G00363-SNP19	16	Potom.016G33876-SNP71	Fiber width	DA	-1.91E+00	2.30E-03
1	Potom.001G03193-SNP10	5	Potom.005G13176-SNP26	Hemicellulose content	DA	-1.34E+01	2.30E-03
2	Potom.002G05769-SNP37	20	Potom.003G08321-SNP26	Stem volume	AD	-8.96E-01	2.30E-03
5	Potom.005G13176-SNP26	8	Potom.008G22699-SNP3	Diameter at breast height	AD	-7.90E+00	2.30E-03
13	Potom.013G29538-SNP2	20	Potom.003G09473-SNP2	Holocellulose content	AA	-6.06E+00	2.30E-03
1	Potom.001G00363-SNP19	16	Potom.016G33876-SNP74	Fiber width	DA	-1.98E+00	2.29E-03
2	Potom.002G05522-SNP1	13	Potom.013G29538-SNP4	Holocellulose content	DD	-3.35E+01	2.29E-03
5	Pto-Wuschela-SNP18	5	Potom.005G13176-SNP162	Microfiber angle	AD	2.32E+00	2.29E-03
5	Potom.005G13176-SNP26	20	Potom.003G09473-SNP11	α-cellulose content	DD	-2.81E+01	2.29E-03
5	Potom.005G13176-SNP41	20	Potom.003G08321-SNP32	α-cellulose content	AD	1.32E+01	2.29E-03
5	Potom.005G13176-SNP284	18	Potom.018G36220-SNP43	Microfiber angle	DA	-6.44E+00	2.29E-03
8	Potom.008G21952-SNP16	20	Potom.003G08321-SNP17	Hemicellulose content	DD	1.91E+01	2.29E-03
2	Potom.002G05522-SNP60	10	Potom.010G25398-SNP68	Diameter at breast height	AD	-1.42E+01	2.28E-03
12	Potom.012G28819-SNP45	20	Potom.003G10089-SNP11	Holocellulose content	AA	1.38E+01	2.28E-03
8	Potom.008G22699-SNP150	20	Potom.003G08321-SNP17	α-cellulose content	DA	-1.48E+01	2.27E-03
1	Potom.001G03193-SNP46	2	Potom.002G05522-SNP4	Hemicellulose content	DA	-1.21E+01	2.25E-03
5	Pto-Wuschela-SNP14	5	Potom.005G13966-SNP74	Holocellulose content	DA	-1.63E+01	2.25E-03
5	Potom.005G13966-SNP89	16	Potom.016G33876-SNP31	Stem volume	DA	-5.48E-01	2.25E-03
8	Potom.008G22699-SNP85	10	Potom.010G24967-SNP53	Holocellulose content	DA	-2.76E+01	2.25E-03
8	Potom.008G22699-SNP108	10	Potom.010G25398-SNP65	Tree height	DA	3.86E+00	2.25E-03
1	Potom.001G00363-SNP23	10	Potom.010G24967-SNP53	Diameter at breast height	DD	1.77E+01	2.24E-03
2	Potom.002G05522-SNP60	2	Potom.002G07337-SNP229	Tree height	AA	-1.59E+00	2.24E-03
1	Potom.001G03353-SNP25	18	Potom.018G35976-SNP25	Microfiber angle	DD	6.48E+00	2.23E-03
2	Potom.002G05522-SNP4	5	Potom.005G13176-SNP41	Holocellulose content	AA	-7.56E+00	2.23E-03

2	Potom.002G05522-SNP12	8	Potom.008G21952-SNP16	Hemicellulose content	AA	-1.34E+01	2.23E-03
8	Potom.008G22699-SNP38	16	Potom.016G33891-SNP29	Holocellulose content	AD	1.81E+01	2.23E-03
2	Potom.002G05632-SNP14	20	Potom.003G08321-SNP3	Hemicellulose content	DD	-2.28E+01	2.22E-03
5	Potom.005G13176-SNP40	8	Potom.008G22699-SNP108	Fiber length	AD	7.54E-02	2.22E-03
10	Potom.010G24967-SNP43	16	Potom.016G33891-SNP28	Diameter at breast height	DD	-1.35E+01	2.22E-03
12	Potom.012G28819-SNP27	20	Potom.003G09473-SNP10	Fiber length	DA	-2.21E-01	2.22E-03
13	Potom.013G29538-SNP9	20	Potom.003G08321-SNP14	Hemicellulose content	AA	1.57E+01	2.22E-03
2	Potom.002G05522-SNP4	5	Potom.005G13176-SNP166	Holocellulose content	AA	-7.54E+00	2.21E-03
2	Potom.002G05522-SNP15	8	Potom.008G22699-SNP85	Holocellulose content	AD	2.58E+01	2.20E-03
1	Potom.001G03606-SNP27	20	Potom.003G09473-SNP2	α-cellulose content	DA	-1.52E+01	2.19E-03
5	Potom.005G13966-SNP90	13	Potom.013G29538-SNP9	Stem volume	DA	-7.35E-01	2.19E-03
20	Potom.003G08321-SNP11	20	Potom.003G09473-SNP2	Fiber length	DD	1.92E-01	2.19E-03
5	Potom.005G13176-SNP26	20	Potom.003G08321-SNP18	α-cellulose content	DA	-1.03E+01	2.18E-03
5	Potom.005G13176-SNP157	16	Potom.016G34352-SNP110	Microfiber angle	AA	3.23E+00	2.18E-03
1	Potom.001G03606-SNP26	10	Potom.010G24967-SNP53	Lignin content	DA	-4.67E+00	2.17E-03
2	Potom.002G05522-SNP1	14	Potom.014G31981-SNP26	Hemicellulose content	DA	2.04E+01	2.16E-03
2	Potom.002G07337-SNP15	2	Potom.002G07963-SNP152	Tree height	DA	-4.80E+00	2.16E-03
5	Pto-Wuschela-SNP12	10	Potom.010G25700-SNP5	Holocellulose content	DA	-1.81E+01	2.16E-03
5	Pto-Wuschela-SNP18	5	Potom.005G13176-SNP166	Lignin content	DD	4.63E+00	2.16E-03
5	Potom.005G13176-SNP284	18	Potom.018G36220-SNP42	Microfiber angle	DA	-6.72E+00	2.16E-03
2	Potom.002G05769-SNP25	16	Potom.016G33876-SNP71	Fiber width	DA	-1.75E+00	2.15E-03
10	Potom.010G24967-SNP45	16	Potom.016G33891-SNP28	Diameter at breast height	DD	-1.34E+01	2.15E-03
2	Potom.002G05522-SNP54	2	Potom.002G07337-SNP15	Fiber length	DD	1.62E-01	2.14E-03
2	Potom.002G05769-SNP25	8	Potom.008G21952-SNP16	Stem volume	AA	-5.23E-01	2.14E-03
5	Potom.005G13176-SNP27	8	Potom.008G21952-SNP16	Holocellulose content	DD	2.35E+01	2.14E-03
2	Potom.002G05522-SNP1	5	Potom.005G13985-SNP20	Hemicellulose content	DA	-1.64E+01	2.12E-03

5	Pto-Wuschela-SNP18	20	Potom.003G08321-SNP19	Lignin content	DA	-2.83E+00	2.12E-03
10	Potom.010G25398-SNP26	10	Potom.010G25398-SNP68	Holocellulose content	DA	1.95E+01	2.12E-03
1	Potom.001G03353-SNP25	2	Potom.002G05522-SNP62	Diameter at breast height	AA	3.74E+00	2.11E-03
2	Potom.002G07206-SNP24	12	Potom.012G28819-SNP46	Holocellulose content	AA	-8.23E+00	2.11E-03
5	Potom.005G13985-SNP4	8	Potom.008G22699-SNP22	Fiber length	DA	2.35E-01	2.11E-03
1	Potom.001G03606-SNP27	5	Potom.005G13333-SNP18	Stem volume	DA	7.36E-01	2.10E-03
2	Potom.002G05769-SNP57	20	Potom.003G08321-SNP6	Diameter at breast height	AA	8.69E+00	2.10E-03
8	Potom.008G22699-SNP74	10	Potom.010G25706-SNP79	Tree height	AD	3.99E+00	2.10E-03
16	Potom.016G34352-SNP98	20	Potom.003G08321-SNP17	Stem volume	AD	-2.64E-01	2.10E-03
5	Potom.005G13176-SNP26	8	Potom.008G22699-SNP3	Stem volume	AD	-5.26E-01	2.09E-03
5	Potom.005G13176-SNP117	13	Potom.013G29538-SNP72	α-cellulose content	DD	-1.59E+01	2.09E-03
5	Potom.005G13966-SNP97	20	Potom.003G09473-SNP2	Diameter at breast height	DA	9.56E+00	2.09E-03
2	Potom.002G05769-SNP57	20	Potom.003G10089-SNP11	α-cellulose content	AA	1.55E+01	2.08E-03
2	Potom.002G07206-SNP24	10	Potom.010G25294-SNP212	Holocellulose content	AA	-1.01E+01	2.08E-03
8	Potom.008G22699-SNP85	10	Potom.010G25398-SNP5	Holocellulose content	DA	-2.58E+01	2.08E-03
5	Potom.005G13966-SNP89	10	Potom.010G25398-SNP26	Stem volume	AD	-6.29E-01	2.07E-03
8	Potom.008G22699-SNP74	20	Potom.003G08321-SNP17	α-cellulose content	AD	-7.00E+00	2.07E-03
1	Potom.001G01788-SNP13	2	Potom.002G05522-SNP54	Stem volume	AD	3.06E-01	2.06E-03
2	Potom.002G05522-SNP15	13	Potom.013G29538-SNP5	Stem volume	AD	-5.07E-01	2.06E-03
2	Potom.002G05769-SNP13	2	Potom.002G07206-SNP24	Holocellulose content	AD	-1.66E+01	2.06E-03
1	Potom.001G03606-SNP27	10	Potom.010G25398-SNP26	Diameter at breast height	AD	-9.96E+00	2.04E-03
2	Potom.002G07337-SNP1	20	Potom.003G08321-SNP18	Fiber length	AA	6.37E-02	2.04E-03
9	Potom.009G23029-SNP3	13	Potom.013G29538-SNP5	Holocellulose content	DD	2.00E+01	2.03E-03
1	Potom.001G03193-SNP20	5	Pto-Wuschela-SNP10	Holocellulose content	AA	5.93E+00	2.01E-03
2	Potom.002G05522-SNP23	2	Potom.002G07337-SNP229	Stem volume	DA	-2.64E-01	1.99E-03
2	Potom.002G05522-SNP52	8	Potom.008G22699-SNP70	Hemicellulose content	AD	1.24E+01	1.99E-03

8	Potom.008G21952-SNP16	20	Potom.003G09473-SNP11	Diameter at breast height	AA	-7.99E+00	1.99E-03
1	Potom.001G00363-SNP19	2	Potom.002G05522-SNP4	Hemicellulose content	AA	-9.15E+00	1.98E-03
1	Potom.001G03193-SNP13	8	Potom.008G22699-SNP9	Stem volume	DA	-8.63E-01	1.98E-03
5	Potom.005G13176-SNP15	14	Potom.014G31981-SNP26	Hemicellulose content	DA	-1.71E+01	1.98E-03
14	Potom.014G31981-SNP25	20	Potom.003G09473-SNP13	Diameter at breast height	AA	-8.87E+00	1.98E-03
16	Potom.016G34352-SNP109	20	Potom.003G09473-SNP2	Fiber length	AD	1.17E-01	1.98E-03
2	Potom.002G07963-SNP231	5	Potom.005G13176-SNP2	Holocellulose content	AA	1.48E+01	1.97E-03
5	Potom.005G13176-SNP26	13	Potom.013G29538-SNP72	Hemicellulose content	DD	-2.04E+01	1.97E-03
5	Potom.005G13176-SNP39	8	Potom.008G22699-SNP108	Fiber length	AD	7.73E-02	1.96E-03
1	Potom.001G03193-SNP20	2	Potom.002G05632-SNP14	Diameter at breast height	DA	-5.08E+00	1.95E-03
2	Potom.002G05522-SNP15	13	Potom.013G29538-SNP71	Fiber width	DD	-5.09E+00	1.95E-03
10	Potom.010G25706-SNP89	13	Potom.013G29538-SNP8	α-cellulose content	AD	-1.21E+01	1.95E-03
1	Potom.001G00363-SNP23	10	Potom.010G25398-SNP68	Diameter at breast height	AD	1.61E+01	1.94E-03
5	Potom.005G13176-SNP27	20	Potom.003G08321-SNP13	Holocellulose content	DD	3.95E+01	1.94E-03
8	Potom.008G22699-SNP150	10	Potom.010G25706-SNP81	Hemicellulose content	AA	-1.18E+01	1.94E-03
1	Potom.001G00363-SNP23	10	Potom.010G25398-SNP26	Diameter at breast height	AD	-1.08E+01	1.92E-03
1	Potom.001G03606-SNP27	10	Potom.010G25398-SNP26	Stem volume	AD	-7.04E-01	1.92E-03
5	Potom.005G13333-SNP18	16	Potom.016G34352-SNP98	Stem volume	DA	-2.32E-01	1.91E-03
2	Potom.002G07337-SNP229	5	Potom.005G13176-SNP2	α-cellulose content	AD	-1.23E+01	1.89E-03
8	Potom.008G21326-SNP69	8	Potom.008G22699-SNP85	Holocellulose content	AD	-2.78E+01	1.88E-03
5	Pto-Wuschela-SNP5	5	Potom.005G13176-SNP162	Fiber width	AD	-1.90E+00	1.87E-03
5	Potom.005G13966-SNP74	12	Potom.012G28409-SNP23	Stem volume	AA	-2.98E-01	1.87E-03
1	Potom.001G03606-SNP26	10	Potom.010G25398-SNP68	Diameter at breast height	AD	1.27E+01	1.85E-03
2	Potom.002G05522-SNP1	5	Potom.005G13985-SNP1	Holocellulose content	DA	-2.60E+01	1.85E-03
2	Potom.002G05522-SNP1	5	Potom.005G13985-SNP2	Holocellulose content	DA	-2.60E+01	1.85E-03
2	Potom.002G05522-SNP15	2	Potom.002G05522-SNP54	Fiber width	AD	3.02E+00	1.85E-03

2	Potom.002G05769-SNP13	8	Potom.008G22699-SNP72	Fiber width	DA	-3.82E+00	1.85E-03
2	Potom.002G05769-SNP43	20	Potom.003G10089-SNP11	Holocellulose content	AA	1.65E+01	1.85E-03
2	Potom.002G05769-SNP70	8	Potom.008G22699-SNP74	Stem volume	DD	7.40E-01	1.85E-03
8	Potom.008G21952-SNP16	8	Potom.008G22699-SNP108	α-cellulose content	AD	-1.33E+01	1.85E-03
1	Potom.001G03353-SNP25	10	Potom.010G24967-SNP43	Fiber length	AD	9.87E-02	1.84E-03
2	Potom.002G05632-SNP14	20	Potom.003G08321-SNP32	Hemicellulose content	AD	-1.26E+01	1.84E-03
2	Potom.002G07963-SNP170	10	Potom.010G25700-SNP13	Fiber length	AA	-7.31E-02	1.84E-03
13	Potom.013G29538-SNP72	20	Potom.003G09473-SNP2	Fiber length	AD	1.14E-01	1.84E-03
2	Potom.002G05769-SNP43	5	Potom.005G13966-SNP77	Diameter at breast height	AA	-3.80E+00	1.83E-03
10	Potom.010G25706-SNP89	13	Potom.013G29538-SNP6	α-cellulose content	AD	-1.21E+01	1.83E-03
1	Potom.001G00363-SNP21	10	Potom.010G25398-SNP68	Diameter at breast height	AD	1.61E+01	1.81E-03
2	Potom.002G05522-SNP1	5	Potom.005G13176-SNP68	Diameter at breast height	AA	-8.30E+00	1.81E-03
2	Potom.002G07206-SNP24	20	Potom.003G08321-SNP3	Holocellulose content	AD	1.05E+01	1.81E-03
8	Potom.008G22699-SNP3	10	Potom.010G25398-SNP68	Stem volume	AD	-9.97E-01	1.81E-03
1	Potom.001G03193-SNP20	2	Potom.002G05522-SNP23	Hemicellulose content	AD	1.19E+01	1.80E-03
1	Potom.001G03193-SNP26	2	Potom.002G05632-SNP14	Diameter at breast height	DA	-6.82E+00	1.80E-03
2	Potom.002G05522-SNP23	8	Potom.008G22699-SNP108	Fiber length	AA	-6.48E-02	1.80E-03
2	Potom.002G07337-SNP229	5	Potom.005G13176-SNP44	Diameter at breast height	AA	-3.73E+00	1.80E-03
1	Potom.001G03193-SNP27	10	Potom.010G25398-SNP5	Fiber length	AA	5.89E-02	1.79E-03
5	Potom.005G13176-SNP44	5	Potom.005G13966-SNP90	Stem volume	DD	-1.43E+00	1.79E-03
5	Potom.005G13333-SNP12	20	Potom.003G10089-SNP11	Microfiber angle	DA	-5.99E+00	1.79E-03
2	Potom.002G05522-SNP23	16	Potom.016G33876-SNP53	Stem volume	AA	3.32E-01	1.78E-03
2	Potom.002G05522-SNP54	5	Potom.005G13176-SNP44	Fiber length	DA	1.20E-01	1.78E-03
2	Potom.002G07206-SNP14	8	Potom.008G22516-SNP43	Microfiber angle	DA	-7.41E+00	1.77E-03
16	Potom.016G33891-SNP28	20	Potom.003G08321-SNP21	Stem volume	DD	-1.03E+00	1.77E-03
10	Potom.010G25398-SNP68	13	Potom.013G29538-SNP2	Stem volume	DA	-8.73E-01	1.76E-03

2	Potom.002G05522-SNP23	20	Potom.003G10089-SNP7	Fiber width	AD	5.36E+00	1.75E-03
2	Potom.002G05522-SNP60	10	Potom.010G25706-SNP89	Stem volume	AD	4.96E-01	1.75E-03
5	Potom.005G13176-SNP44	13	Potom.013G29538-SNP71	α-cellulose content	DA	8.61E+00	1.75E-03
12	Potom.012G28819-SNP45	20	Potom.003G08321-SNP6	Stem volume	AA	5.85E-01	1.75E-03
13	Potom.013G29538-SNP10	20	Potom.003G08321-SNP17	Stem volume	AA	-3.60E-01	1.75E-03
8	Potom.008G21952-SNP16	16	Potom.016G33891-SNP13	Holocellulose content	DA	-2.45E+01	1.74E-03
10	Potom.010G24967-SNP67	20	Potom.003G08321-SNP3	Stem volume	AA	2.10E-01	1.74E-03
5	Potom.005G13333-SNP13	13	Potom.013G29538-SNP4	α-cellulose content	AA	6.17E+00	1.73E-03
8	Potom.008G22699-SNP9	8	Potom.008G22699-SNP72	Stem volume	AD	6.81E-01	1.73E-03
9	Potom.009G22902-SNP9	9	Potom.009G23017-SNP55	Hemicellulose content	DD	-2.65E+01	1.72E-03
5	Potom.005G13176-SNP40	20	Potom.003G08321-SNP32	Hemicellulose content	AA	5.57E+00	1.71E-03
1	Potom.001G00363-SNP23	10	Potom.010G24967-SNP53	Lignin content	DD	7.39E+00	1.70E-03
8	Potom.008G22699-SNP22	13	Potom.013G29538-SNP7	Stem volume	DA	-6.84E-01	1.70E-03
10	Potom.010G24967-SNP67	16	Potom.016G33891-SNP28	Fiber width	DA	-3.23E+00	1.70E-03
2	Potom.002G05522-SNP73	20	Potom.003G08321-SNP3	Fiber length	AA	-4.67E-02	1.69E-03
2	Potom.002G05769-SNP70	8	Potom.008G21326-SNP69	Stem volume	AA	-4.69E-01	1.68E-03
2	Potom.002G07337-SNP34	5	Potom.005G13176-SNP26	Hemicellulose content	AD	1.18E+01	1.68E-03
2	Potom.002G07963-SNP231	5	Potom.005G13176-SNP15	Holocellulose content	DD	-2.95E+01	1.67E-03
8	Potom.008G21952-SNP16	13	Potom.013G29538-SNP71	Hemicellulose content	DD	-2.63E+01	1.67E-03
8	Potom.008G22699-SNP72	16	Potom.016G33891-SNP29	Diameter at breast height	AD	8.59E+00	1.67E-03
2	Potom.002G05522-SNP12	2	Potom.002G05522-SNP73	Holocellulose content	AD	-1.11E+01	1.66E-03
16	Potom.016G34352-SNP58	20	Potom.003G08321-SNP3	Holocellulose content	DD	-3.69E+01	1.66E-03
1	Potom.001G03193-SNP10	8	Potom.008G21326-SNP10	Hemicellulose content	DD	-2.14E+01	1.65E-03
1	Potom.001G03193-SNP31	2	Potom.002G05522-SNP23	Stem volume	DA	-7.95E-01	1.65E-03
5	Pto-Wuschela-SNP10	13	Potom.013G29538-SNP16	Hemicellulose content	DD	1.52E+01	1.65E-03
5	Potom.005G13176-SNP44	5	Potom.005G13333-SNP12	Stem volume	AD	4.62E-01	1.65E-03

5	Potom.005G13333-SNP13	20	Potom.003G08321-SNP9	Lignin content	DD	-8.88E+00	1.64E-03
10	Potom.010G25398-SNP68	13	Potom.013G29538-SNP2	α-cellulose content	DA	1.94E+01	1.64E-03
1	Potom.001G01788-SNP13	10	Potom.010G25294-SNP173	Microfiber angle	AA	1.16E+00	1.63E-03
2	Potom.002G05522-SNP23	5	Potom.005G13333-SNP18	α-cellulose content	DD	1.41E+01	1.63E-03
14	Potom.014G31981-SNP26	16	Potom.016G34352-SNP98	α-cellulose content	AA	6.63E+00	1.63E-03
1	Potom.001G03353-SNP164	2	Potom.002G05522-SNP4	Holocellulose content	AA	-7.95E+00	1.62E-03
1	Potom.001G03606-SNP26	10	Potom.010G25398-SNP68	Holocellulose content	AA	-1.39E+01	1.61E-03
16	Potom.016G33876-SNP108	20	Potom.003G09473-SNP7	Diameter at breast height	AD	1.14E+01	1.61E-03
5	Potom.005G13176-SNP115	16	Potom.016G33891-SNP13	Diameter at breast height	AA	-6.88E+00	1.60E-03
13	Potom.013G29538-SNP72	16	Potom.016G33876-SNP108	Stem volume	AA	-3.13E-01	1.60E-03
2	Potom.002G05522-SNP23	16	Potom.016G34352-SNP22	Diameter at breast height	AA	-5.31E+00	1.59E-03
8	Potom.008G22699-SNP108	16	Potom.016G34352-SNP108	Tree height	DA	2.37E+00	1.59E-03
16	Potom.016G33876-SNP108	18	Potom.018G36220-SNP42	Microfiber angle	AA	3.53E+00	1.57E-03
2	Potom.002G05769-SNP43	8	Potom.008G21952-SNP16	Stem volume	AA	-5.58E-01	1.56E-03
8	Potom.008G22699-SNP71	20	Potom.003G08321-SNP3	Holocellulose content	DD	-3.53E+01	1.56E-03
2	Potom.002G07206-SNP24	5	Potom.005G13966-SNP89	Diameter at breast height	AD	9.63E+00	1.55E-03
1	Potom.001G01788-SNP70	12	Potom.012G28409-SNP11	Fiber length	AA	8.76E-02	1.54E-03
1	Potom.001G03353-SNP164	14	Potom.014G31981-SNP6	Diameter at breast height	AD	-7.10E+00	1.54E-03
2	Potom.002G05522-SNP54	10	Potom.010G25706-SNP89	Stem volume	AD	6.07E-01	1.54E-03
18	Potom.018G35976-SNP25	20	Potom.003G09473-SNP2	Microfiber angle	AD	-5.18E+00	1.54E-03
2	Potom.002G07337-SNP144	5	Potom.005G13985-SNP5	Fiber length	DD	-2.17E-01	1.53E-03
2	Potom.002G07337-SNP145	5	Potom.005G13985-SNP5	Fiber length	DD	-2.17E-01	1.53E-03
8	Potom.008G21326-SNP69	16	Potom.016G33891-SNP28	Stem volume	AD	-6.36E-01	1.53E-03
20	Potom.003G08321-SNP9	20	Potom.003G08321-SNP20	Diameter at breast height	DD	-1.52E+01	1.53E-03
2	Potom.002G05769-SNP13	5	Potom.005G13176-SNP2	Holocellulose content	DA	2.14E+01	1.52E-03
5	Pto-Wuschela-SNP5	5	Potom.005G13176-SNP117	Holocellulose content	AA	4.81E+00	1.52E-03

2	Potom.002G07337-SNP229	5	Pto-Wuschela-SNP5	Stem volume	AA	-1.82E-01	1.51E-03
5	Potom.005G13176-SNP27	12	Potom.012G28819-SNP27	Holocellulose content	DA	2.80E+01	1.51E-03
10	Potom.010G24967-SNP45	20	Potom.003G08321-SNP10	Fiber length	DA	7.08E-02	1.51E-03
16	Potom.016G33891-SNP28	20	Potom.003G09473-SNP2	Stem volume	DA	6.42E-01	1.51E-03
20	Potom.003G08321-SNP7	20	Potom.003G08321-SNP21	α-cellulose content	AD	-1.48E+01	1.51E-03
1	Potom.001G03353-SNP25	8	Potom.008G22699-SNP3	Diameter at breast height	DD	-8.72E+00	1.50E-03
2	Potom.002G05522-SNP15	18	Potom.018G36220-SNP10	Lignin content	DA	-1.56E+00	1.50E-03
8	Potom.008G21952-SNP16	10	Potom.010G25398-SNP26	Holocellulose content	DD	-2.78E+01	1.50E-03
8	Potom.008G22699-SNP74	8	Potom.008G22699-SNP150	α-cellulose content	AD	-1.36E+01	1.50E-03
10	Potom.010G25398-SNP68	13	Potom.013G29538-SNP4	Stem volume	DA	-8.82E-01	1.50E-03
2	Potom.002G05522-SNP12	5	Pto-Wuschela-SNP5	Lignin content	DA	-2.74E+00	1.48E-03
5	Potom.005G13176-SNP68	16	Potom.016G33891-SNP27	Diameter at breast height	DA	1.12E+01	1.48E-03
9	Potom.009G23029-SNP3	13	Potom.013G29538-SNP4	Holocellulose content	AA	7.17E+00	1.48E-03
1	Potom.001G03193-SNP26	5	Potom.005G13176-SNP2	Stem volume	DD	1.46E+00	1.47E-03
5	Pto-Wuschela-SNP14	20	Potom.003G08321-SNP5	Holocellulose content	DA	-1.50E+01	1.47E-03
5	Potom.005G13176-SNP16	20	Potom.003G08321-SNP26	Diameter at breast height	AD	-8.40E+00	1.47E-03
5	Potom.005G13176-SNP47	16	Potom.016G33876-SNP73	Fiber width	DD	-4.58E+00	1.47E-03
16	Potom.016G33876-SNP25	20	Potom.003G08321-SNP17	Holocellulose content	AD	2.25E+01	1.47E-03
20	Potom.003G08321-SNP4	20	Potom.003G09473-SNP2	α-cellulose content	DA	-2.33E+01	1.47E-03
2	Potom.002G05769-SNP70	8	Potom.008G21952-SNP16	Diameter at breast height	AA	-7.51E+00	1.46E-03
5	Potom.005G13333-SNP12	10	Potom.010G25706-SNP89	Stem volume	AA	-3.06E-01	1.46E-03
2	Potom.002G05522-SNP12	8	Potom.008G21952-SNP16	α-cellulose content	DA	-1.45E+01	1.45E-03
2	Potom.002G07206-SNP24	12	Potom.012G28819-SNP27	Holocellulose content	AA	-1.58E+01	1.45E-03
2	Potom.002G07206-SNP24	12	Potom.012G28819-SNP45	Holocellulose content	AA	-8.73E+00	1.45E-03
5	Potom.005G13176-SNP62	8	Potom.008G22699-SNP3	Stem volume	DD	-6.12E-01	1.44E-03
5	Potom.005G13966-SNP93	16	Potom.016G34352-SNP98	Holocellulose content	AA	7.74E+00	1.44E-03

8	Potom.008G22699-SNP9	8	Potom.008G22699-SNP72	Diameter at breast height	DD	1.59E+01	1.44E-03
10	Potom.010G25706-SNP89	13	Potom.013G29538-SNP9	α-cellulose content	AD	-1.14E+01	1.44E-03
5	Pto-Wuschela-SNP5	8	Potom.008G22699-SNP85	Holocellulose content	AD	-2.80E+01	1.43E-03
5	Potom.005G13176-SNP117	8	Potom.008G22699-SNP74	Hemicellulose content	DA	-1.04E+01	1.43E-03
8	Potom.008G22699-SNP150	10	Potom.010G25706-SNP79	Hemicellulose content	AA	-1.22E+01	1.43E-03
2	Potom.002G05522-SNP62	8	Potom.008G22699-SNP71	Diameter at breast height	DD	-1.27E+01	1.42E-03
10	Potom.010G25398-SNP68	13	Potom.013G29538-SNP4	α-cellulose content	DA	1.92E+01	1.42E-03
1	Potom.001G03193-SNP20	2	Potom.002G05522-SNP23	Stem volume	DD	-7.76E-01	1.41E-03
2	Potom.002G05522-SNP12	5	Pto-Wuschela-SNP5	Holocellulose content	DA	1.00E+01	1.41E-03
2	Potom.002G05522-SNP60	8	Potom.008G22699-SNP71	Diameter at breast height	DD	-1.27E+01	1.41E-03
8	Potom.008G22699-SNP74	16	Potom.016G33876-SNP56	Stem volume	DD	8.34E-01	1.41E-03
13	Potom.013G29538-SNP9	16	Potom.016G33891-SNP29	Stem volume	AA	-4.98E-01	1.41E-03
1	Potom.001G00363-SNP19	16	Potom.016G33876-SNP73	Fiber width	DA	-2.05E+00	1.40E-03
5	Potom.005G13176-SNP3	16	Potom.016G33876-SNP31	α-cellulose content	DD	1.93E+01	1.40E-03
5	Potom.005G13985-SNP5	20	Potom.003G09473-SNP2	Fiber length	DA	-1.59E-01	1.40E-03
8	Potom.008G22699-SNP22	13	Potom.013G29538-SNP9	Stem volume	DA	-7.04E-01	1.40E-03
2	Potom.002G05522-SNP62	10	Potom.010G25706-SNP89	Stem volume	AD	5.10E-01	1.39E-03
1	Potom.001G03193-SNP30	2	Potom.002G05522-SNP23	Stem volume	DA	-8.09E-01	1.38E-03
1	Potom.001G03606-SNP27	8	Potom.008G22699-SNP69	Fiber length	AA	1.62E-01	1.38E-03
2	Potom.002G05522-SNP62	12	Potom.012G28409-SNP23	Stem volume	AD	4.29E-01	1.38E-03
5	Potom.005G13176-SNP44	5	Potom.005G13333-SNP12	Fiber width	AD	-2.44E+00	1.38E-03
8	Potom.008G21326-SNP66	16	Potom.016G33891-SNP26	Diameter at breast height	AA	-6.61E+00	1.38E-03
1	Potom.001G03193-SNP46	5	Pto-Wuschela-SNP14	Hemicellulose content	AD	-9.93E+00	1.37E-03
1	Potom.001G03353-SNP25	2	Potom.002G05522-SNP60	Diameter at breast height	AA	3.82E+00	1.37E-03
2	Potom.002G05522-SNP62	5	Potom.005G13333-SNP12	Stem volume	DD	7.14E-01	1.37E-03
16	Potom.016G33876-SNP31	20	Potom.003G08321-SNP11	Stem volume	AA	4.65E-01	1.37E-03

8	Potom.008G22699-SNP63	18	Potom.018G35976-SNP25	Microfiber angle	DA	-1.01E+01	1.36E-03
10	Potom.010G25398-SNP65	18	Potom.018G35976-SNP25	Fiber length	AD	1.27E-01	1.36E-03
1	Potom.001G00363-SNP19	2	Potom.002G05522-SNP23	Stem volume	AA	-3.92E-01	1.35E-03
2	Potom.002G05522-SNP15	13	Potom.013G29538-SNP7	Stem volume	AD	-6.34E-01	1.35E-03
5	Pto-Wuschela-SNP5	10	Potom.010G24967-SNP27	Hemicellulose content	AA	-6.17E+00	1.35E-03
5	Pto-Wuschela-SNP14	20	Potom.003G08321-SNP5	Lignin content	DA	3.97E+00	1.35E-03
1	Potom.001G03606-SNP26	10	Potom.010G25398-SNP68	Holocellulose content	DD	-2.78E+01	1.34E-03
1	Potom.001G03193-SNP10	10	Potom.010G24967-SNP27	Hemicellulose content	DD	-2.18E+01	1.33E-03
8	Potom.008G22516-SNP49	16	Potom.016G33876-SNP72	Hemicellulose content	AD	1.78E+01	1.33E-03
2	Potom.002G05769-SNP70	16	Potom.016G34352-SNP108	Hemicellulose content	AA	-6.39E+00	1.32E-03
8	Potom.008G22699-SNP98	20	Potom.003G08321-SNP13	Stem volume	AD	-1.17E+00	1.32E-03
13	Potom.013G29538-SNP72	20	Potom.003G09473-SNP2	Microfiber angle	AD	-6.36E+00	1.32E-03
5	Potom.005G13176-SNP39	8	Potom.008G22699-SNP74	α-cellulose content	AA	-5.04E+00	1.31E-03
2	Potom.002G05522-SNP3	8	Potom.008G21952-SNP16	Holocellulose content	AD	1.89E+01	1.30E-03
2	Potom.002G05522-SNP13	5	Potom.005G13176-SNP2	Stem volume	AA	-7.48E-01	1.30E-03
2	Potom.002G05522-SNP13	20	Potom.003G08321-SNP1	Diameter at breast height	DA	1.44E+01	1.30E-03
2	Potom.002G05522-SNP14	5	Potom.005G13176-SNP2	Stem volume	AA	-7.48E-01	1.30E-03
2	Potom.002G05522-SNP14	20	Potom.003G08321-SNP1	Diameter at breast height	DA	1.44E+01	1.30E-03
2	Potom.002G05522-SNP60	5	Potom.005G13333-SNP12	Stem volume	DD	7.14E-01	1.30E-03
5	Potom.005G13176-SNP162	10	Potom.010G24967-SNP58	Fiber length	DD	3.31E-01	1.30E-03
8	Potom.008G22699-SNP98	20	Potom.003G08321-SNP14	Stem volume	AD	-1.16E+00	1.29E-03
10	Potom.010G25398-SNP63	16	Potom.016G33876-SNP59	Stem volume	DD	1.72E+00	1.29E-03
10	Potom.010G25398-SNP63	16	Potom.016G33876-SNP60	Stem volume	DD	1.72E+00	1.29E-03
14	Potom.014G31981-SNP26	16	Potom.016G34352-SNP98	Holocellulose content	AA	7.51E+00	1.29E-03
2	Potom.002G05522-SNP60	5	Potom.005G13333-SNP12	Diameter at breast height	DD	1.04E+01	1.28E-03
5	Potom.005G13966-SNP89	10	Potom.010G25294-SNP212	Stem volume	DA	-9.46E-01	1.28E-03

5	Potom.005G13966-SNP89	20	Potom.003G08321-SNP21	Holocellulose content	AD	1.75E+01	1.28E-03
8	Potom.008G22699-SNP71	8	Potom.008G22699-SNP72	Holocellulose content	AA	1.87E+01	1.27E-03
10	Potom.010G25398-SNP26	20	Potom.003G08321-SNP14	Holocellulose content	DA	2.21E+01	1.27E-03
2	Potom.002G05522-SNP62	10	Potom.010G24967-SNP67	Fiber length	DD	-1.92E-01	1.26E-03
2	Potom.002G05769-SNP57	20	Potom.003G10089-SNP11	Holocellulose content	AA	1.80E+01	1.26E-03
2	Potom.002G07337-SNP15	8	Potom.008G22699-SNP3	Fiber length	DA	8.50E-02	1.26E-03
5	Pto-Wuschela-SNP10	13	Potom.013G29538-SNP15	Hemicellulose content	DD	1.57E+01	1.26E-03
5	Potom.005G13333-SNP13	8	Potom.008G22699-SNP74	Stem volume	DA	4.24E-01	1.26E-03
5	Potom.005G13333-SNP12	10	Potom.010G25700-SNP13	Diameter at breast height	DA	1.66E+01	1.25E-03
8	Potom.008G22699-SNP72	20	Potom.003G08321-SNP17	Fiber width	DA	3.79E+00	1.25E-03
1	Potom.001G03606-SNP26	2	Potom.002G05522-SNP73	Holocellulose content	DA	1.10E+01	1.24E-03
5	Pto-Wuschela-SNP18	5	Potom.005G13176-SNP168	Lignin content	DD	4.61E+00	1.24E-03
1	Potom.001G03193-SNP31	10	Potom.010G25294-SNP212	Diameter at breast height	DD	-1.47E+01	1.23E-03
5	Pto-Wuschela-SNP12	14	Potom.014G31981-SNP26	Holocellulose content	DA	-1.54E+01	1.23E-03
5	Pto-Wuschela-SNP12	20	Potom.003G08321-SNP3	Hemicellulose content	DA	1.52E+01	1.23E-03
8	Potom.008G22699-SNP73	8	Potom.008G22699-SNP108	Fiber length	AD	-9.63E-02	1.23E-03
2	Potom.002G05522-SNP4	20	Potom.003G08321-SNP3	Holocellulose content	AA	9.77E+00	1.22E-03
5	Potom.005G13176-SNP62	8	Potom.008G22699-SNP108	Fiber length	DD	1.31E-01	1.22E-03
1	Potom.001G03193-SNP46	5	Pto-Wuschela-SNP14	Diameter at breast height	DD	1.51E+01	1.21E-03
8	Potom.008G22516-SNP43	20	Potom.003G08321-SNP5	Fiber width	DA	5.24E+00	1.21E-03
8	Potom.008G22699-SNP85	16	Potom.016G34352-SNP43	Holocellulose content	DA	-3.08E+01	1.21E-03
2	Potom.002G05522-SNP23	20	Potom.003G08321-SNP20	Diameter at breast height	AA	3.49E+00	1.20E-03
2	Potom.002G05522-SNP60	10	Potom.010G24967-SNP67	Fiber length	DD	-1.93E-01	1.20E-03
2	Potom.002G07337-SNP56	5	Potom.005G13966-SNP89	Holocellulose content	AA	2.05E+01	1.20E-03
10	Potom.010G25398-SNP26	20	Potom.003G08321-SNP14	Stem volume	AD	-1.17E+00	1.20E-03
13	Potom.013G29538-SNP2	16	Potom.016G33891-SNP29	Stem volume	DD	9.68E-01	1.20E-03

10	Potom.010G24967-SNP45	20	Potom.003G08321-SNP32	Hemicellulose content	AD	-1.35E+01	1.19E-03
2	Potom.002G05769-SNP13	5	Potom.005G13176-SNP27	Stem volume	AA	-7.19E-01	1.18E-03
8	Potom.008G22699-SNP85	10	Potom.010G25398-SNP26	Hemicellulose content	DD	-3.22E+01	1.18E-03
1	Potom.001G00363-SNP26	5	Potom.005G13176-SNP4	Stem volume	AD	9.48E-01	1.17E-03
1	Potom.001G03193-SNP27	12	Potom.012G28409-SNP23	Stem volume	AA	-3.76E-01	1.17E-03
1	Potom.001G03193-SNP13	2	Potom.002G05522-SNP3	Stem volume	DA	8.31E-01	1.16E-03
2	Potom.002G07337-SNP34	16	Potom.016G33876-SNP72	Lignin content	AA	-1.26E+00	1.16E-03
5	Potom.005G13333-SNP17	9	Potom.009G23017-SNP54	Fiber width	DD	6.02E+00	1.16E-03
8	Potom.008G22699-SNP108	16	Potom.016G34352-SNP109	Fiber length	DA	9.93E-02	1.16E-03
10	Potom.010G25398-SNP26	20	Potom.003G08321-SNP14	Tree height	AA	4.64E+00	1.16E-03
12	Potom.012G28819-SNP45	20	Potom.003G08321-SNP5	Diameter at breast height	AA	-8.77E+00	1.16E-03
2	Potom.002G07963-SNP163	16	Potom.016G33891-SNP6	Fiber length	DA	-1.48E-01	1.15E-03
5	Pto-Wuschela-SNP12	20	Potom.003G08321-SNP33	Holocellulose content	DA	-1.72E+01	1.15E-03
13	Potom.013G29538-SNP9	16	Potom.016G33891-SNP29	Fiber length	AD	-1.93E-01	1.15E-03
1	Potom.001G00363-SNP19	2	Potom.002G05522-SNP23	Stem volume	AD	-6.01E-01	1.14E-03
2	Potom.002G05522-SNP60	20	Potom.003G09473-SNP2	Fiber length	DD	1.71E-01	1.14E-03
2	Potom.002G05522-SNP62	20	Potom.003G09473-SNP2	Fiber length	DD	1.71E-01	1.14E-03
2	Potom.002G07206-SNP24	2	Potom.002G07337-SNP229	Stem volume	AA	3.01E-01	1.14E-03
13	Potom.013G29538-SNP5	20	Potom.003G08321-SNP13	Stem volume	DD	-1.44E+00	1.14E-03
8	Potom.008G22699-SNP3	20	Potom.003G10089-SNP11	Holocellulose content	DA	1.51E+01	1.13E-03
16	Potom.016G33876-SNP108	16	Potom.016G33891-SNP28	Stem volume	DA	7.56E-01	1.13E-03
1	Potom.001G03193-SNP26	2	Potom.002G05522-SNP23	Stem volume	DD	-8.42E-01	1.12E-03
5	Potom.005G13176-SNP47	16	Potom.016G33876-SNP74	Fiber width	DD	-4.68E+00	1.12E-03
5	Potom.005G13966-SNP90	8	Potom.008G22699-SNP72	Stem volume	DD	-1.17E+00	1.12E-03
1	Potom.001G03606-SNP27	12	Potom.012G28819-SNP27	Fiber length	AA	1.43E-01	1.11E-03
5	Potom.005G13176-SNP41	12	Potom.012G28409-SNP23	Stem volume	DA	-2.82E-01	1.11E-03

1	Potom.001G03193-SNP10	5	Potom.005G13176-SNP41	Hemicellulose content	DA	-1.55E+01	1.10E-03
2	Potom.002G05522-SNP62	5	Potom.005G13333-SNP12	Diameter at breast height	DD	1.06E+01	1.10E-03
5	Potom.005G13176-SNP30	8	Potom.008G22699-SNP108	Fiber length	AD	7.26E-02	1.10E-03
10	Potom.010G25706-SNP89	20	Potom.003G08321-SNP20	Stem volume	AA	4.10E-01	1.10E-03
1	Potom.001G00363-SNP19	2	Potom.002G05769-SNP43	Stem volume	AA	-5.85E-01	1.09E-03
5	Potom.005G13966-SNP97	13	Potom.013G29538-SNP9	Stem volume	DA	7.40E-01	1.09E-03
9	Potom.009G22902-SNP16	10	Potom.010G25398-SNP63	Stem volume	AD	-1.27E+00	1.09E-03
16	Potom.016G33891-SNP17	20	Potom.003G08321-SNP26	Stem volume	AD	-7.03E-01	1.09E-03
2	Potom.002G05522-SNP4	2	Potom.002G05522-SNP12	Holocellulose content	AA	-6.92E+00	1.08E-03
8	Potom.008G22699-SNP3	14	Potom.014G31981-SNP25	Diameter at breast height	DD	-1.28E+01	1.08E-03
10	Potom.010G24967-SNP43	20	Potom.003G08321-SNP32	Hemicellulose content	AD	-1.36E+01	1.08E-03
5	Potom.005G13333-SNP12	16	Potom.016G33891-SNP15	Tree height	DA	5.02E+00	1.07E-03
1	Potom.001G03193-SNP46	5	Pto-Wuschela-SNP14	Hemicellulose content	AA	1.20E+01	1.06E-03
5	Potom.005G13176-SNP117	8	Potom.008G22699-SNP74	α-cellulose content	AA	-6.33E+00	1.06E-03
8	Potom.008G21952-SNP16	10	Potom.010G25294-SNP212	Diameter at breast height	AA	-8.45E+00	1.06E-03
1	Potom.001G03606-SNP26	2	Potom.002G05522-SNP60	Holocellulose content	DD	2.63E+01	1.05E-03
5	Potom.005G13176-SNP44	16	Potom.016G34352-SNP98	Stem volume	AA	-4.16E-01	1.05E-03
1	Potom.001G03353-SNP25	13	Potom.013G29538-SNP8	Stem volume	DD	-1.03E+00	1.04E-03
5	Pto-Wuschela-SNP5	5	Potom.005G13176-SNP16	Holocellulose content	AA	7.54E+00	1.04E-03
5	Pto-Wuschela-SNP14	5	Potom.005G13176-SNP16	Holocellulose content	DA	-1.55E+01	1.04E-03
2	Potom.002G05769-SNP72	8	Potom.008G21952-SNP16	Diameter at breast height	AA	-7.98E+00	1.03E-03
2	Potom.002G07963-SNP163	16	Potom.016G34352-SNP87	Fiber length	DD	1.89E-01	1.03E-03
2	Potom.002G07963-SNP163	16	Potom.016G34352-SNP88	Fiber length	DD	1.89E-01	1.03E-03
1	Potom.001G00363-SNP19	12	Potom.012G28819-SNP45	Diameter at breast height	AA	-7.47E+00	1.02E-03
2	Potom.002G05522-SNP60	12	Potom.012G28409-SNP23	Stem volume	AD	4.39E-01	1.02E-03
13	Potom.013G29538-SNP9	20	Potom.003G08321-SNP17	α-cellulose content	AA	8.59E+00	1.02E-03

1	Potom.001G03606-SNP26	2	Potom.002G05522-SNP62	Holocellulose content	DD	2.65E+01	1.01E-03
5	Potom.005G13176-SNP16	8	Potom.008G22699-SNP108	Fiber length	AD	1.06E-01	1.01E-03
5	Potom.005G13966-SNP90	20	Potom.003G08321-SNP17	Stem volume	DA	9.87E-01	1.01E-03
10	Potom.010G25706-SNP89	20	Potom.003G08321-SNP20	Stem volume	AD	6.28E-01	1.01E-03
5	Pto-Wuschela-SNP12	14	Potom.014G31981-SNP25	Holocellulose content	DA	-1.59E+01	9.99E-04
8	Potom.008G22699-SNP3	14	Potom.014G31981-SNP25	Holocellulose content	DA	1.37E+01	9.94E-04
2	Potom.002G07206-SNP24	10	Potom.010G24967-SNP43	Fiber length	AD	-9.89E-02	9.92E-04
1	Potom.001G00363-SNP19	2	Potom.002G07337-SNP16	Diameter at breast height	AD	-8.09E+00	9.87E-04
5	Pto-Wuschela-SNP14	5	Potom.005G13176-SNP47	Lignin content	DA	3.24E+00	9.86E-04
8	Potom.008G22699-SNP100	20	Potom.003G08321-SNP3	Diameter at breast height	AD	6.15E+00	9.86E-04
13	Potom.013G29538-SNP72	14	Potom.014G31981-SNP25	Fiber width	DA	-3.51E+00	9.81E-04
20	Potom.003G08321-SNP1	20	Potom.003G09473-SNP2	α-cellulose content	DA	-2.32E+01	9.77E-04
2	Potom.002G05769-SNP37	20	Potom.003G08321-SNP17	Holocellulose content	AD	1.94E+01	9.74E-04
2	Potom.002G07337-SNP15	5	Potom.005G13966-SNP90	Stem volume	AD	-8.98E-01	9.74E-04
2	Potom.002G05769-SNP13	5	Potom.005G13176-SNP27	Holocellulose content	DA	2.34E+01	9.73E-04
16	Potom.016G33891-SNP28	20	Potom.003G08321-SNP32	Stem volume	DA	6.20E-01	9.73E-04
16	Potom.016G33891-SNP27	20	Potom.003G08321-SNP17	Stem volume	DA	7.05E-01	9.66E-04
2	Potom.002G05522-SNP13	8	Potom.008G22699-SNP72	Diameter at breast height	DA	8.10E+00	9.64E-04
2	Potom.002G05522-SNP14	8	Potom.008G22699-SNP72	Diameter at breast height	DA	8.10E+00	9.64E-04
2	Potom.002G05769-SNP13	8	Potom.008G22699-SNP72	Diameter at breast height	AD	1.25E+01	9.58E-04
1	Potom.001G03353-SNP25	13	Potom.013G29538-SNP6	Stem volume	DD	-1.03E+00	9.57E-04
2	Potom.002G05522-SNP15	2	Potom.002G07963-SNP185	Diameter at breast height	AA	-2.16E+00	9.56E-04
5	Potom.005G13966-SNP90	13	Potom.013G29538-SNP72	Stem volume	DA	-8.42E-01	9.54E-04
2	Potom.002G05769-SNP72	5	Potom.005G13333-SNP12	Stem volume	AD	7.74E-01	9.53E-04
2	Potom.002G05522-SNP1	10	Potom.010G25398-SNP68	Hemicellulose content	DA	2.50E+01	9.45E-04
1	Potom.001G00363-SNP19	20	Potom.003G09473-SNP7	Diameter at breast height	AD	1.52E+01	9.44E-04

2	Potom.002G05522-SNP4	5	Potom.005G13176-SNP39	Holocellulose content	AA	-9.45E+00	9.44E-04
10	Potom.010G25294-SNP212	10	Potom.010G25706-SNP89	Stem volume	AD	7.42E-01	9.40E-04
2	Potom.002G05769-SNP43	8	Potom.008G21952-SNP16	Holocellulose content	AD	-2.02E+01	9.30E-04
2	Potom.002G05522-SNP13	5	Potom.005G13176-SNP2	Diameter at breast height	AA	-1.11E+01	9.20E-04
2	Potom.002G05522-SNP14	5	Potom.005G13176-SNP2	Diameter at breast height	AA	-1.11E+01	9.20E-04
2	Potom.002G05769-SNP70	8	Potom.008G21952-SNP16	Stem volume	AA	-5.33E-01	9.17E-04
1	Potom.001G03193-SNP27	16	Potom.016G34352-SNP109	Holocellulose content	AD	1.58E+01	9.12E-04
2	Potom.002G07963-SNP153	5	Potom.005G13176-SNP26	α-cellulose content	DD	-3.26E+01	9.11E-04
5	Potom.005G13966-SNP90	8	Potom.008G22699-SNP72	Diameter at breast height	AA	-7.76E+00	9.08E-04
14	Potom.014G31981-SNP57	20	Potom.003G08321-SNP17	Stem volume	AD	2.57E-01	9.08E-04
2	Potom.002G07337-SNP229	20	Potom.003G08321-SNP2	Stem volume	DA	7.25E-01	9.04E-04
8	Potom.008G22699-SNP3	8	Potom.008G22699-SNP72	Diameter at breast height	DD	1.18E+01	9.01E-04
5	Potom.005G13176-SNP27	8	Potom.008G22699-SNP3	Diameter at breast height	AD	-9.04E+00	8.97E-04
5	Potom.005G13176-SNP115	13	Potom.013G29538-SNP71	Fiber width	AD	-3.79E+00	8.96E-04
8	Potom.008G22699-SNP38	20	Potom.003G09473-SNP10	Holocellulose content	AA	2.15E+01	8.95E-04
8	Potom.008G22699-SNP108	8	Potom.008G22699-SNP153	Fiber length	DD	1.43E-01	8.95E-04
2	Potom.002G05522-SNP23	10	Potom.010G25706-SNP89	Fiber width	DA	-2.04E+00	8.93E-04
5	Potom.005G13176-SNP2	10	Potom.010G25706-SNP46	α-cellulose content	DA	-1.44E+01	8.93E-04
1	Potom.001G03193-SNP20	8	Potom.008G22699-SNP72	Stem volume	DA	-7.17E-01	8.87E-04
2	Potom.002G05769-SNP43	2	Potom.002G07963-SNP185	Microfiber angle	DA	3.13E+00	8.86E-04
5	Potom.005G13333-SNP12	10	Potom.010G25294-SNP212	Tree height	DA	4.68E+00	8.84E-04
1	Potom.001G03353-SNP25	5	Potom.005G13176-SNP16	Holocellulose content	AA	-9.68E+00	8.83E-04
10	Potom.010G24967-SNP40	20	Potom.003G09473-SNP10	Holocellulose content	DA	2.13E+01	8.79E-04
2	Potom.002G05522-SNP1	14	Potom.014G31981-SNP25	Hemicellulose content	DA	2.20E+01	8.78E-04
13	Potom.013G29538-SNP71	20	Potom.003G08321-SNP7	Fiber width	DD	-5.58E+00	8.78E-04
5	Potom.005G13333-SNP18	14	Potom.014G31981-SNP25	Fiber width	DD	5.06E+00	8.66E-04

8	Potom.008G22699-SNP71	16	Potom.016G33876-SNP31	Stem volume	DA	-4.86E-01	8.65E-04
5	Potom.005G13176-SNP27	20	Potom.003G08321-SNP13	Tree height	DD	-1.20E+01	8.64E-04
5	Potom.005G13333-SNP13	13	Potom.013G29538-SNP5	α-cellulose content	AA	8.07E+00	8.64E-04
2	Potom.002G07206-SNP38	16	Potom.016G33891-SNP28	Stem volume	DD	-1.16E+00	8.61E-04
8	Potom.008G22699-SNP72	16	Potom.016G33891-SNP28	Stem volume	DA	7.15E-01	8.58E-04
8	Potom.008G22699-SNP74	13	Potom.013G29538-SNP7	Stem volume	AD	4.67E-01	8.58E-04
16	Potom.016G34352-SNP109	20	Potom.003G09473-SNP2	Diameter at breast height	AD	8.90E+00	8.55E-04
5	Potom.005G13176-SNP27	5	Potom.005G13966-SNP97	Stem volume	AA	-6.03E-01	8.37E-04
13	Potom.013G29538-SNP7	20	Potom.003G08321-SNP17	α-cellulose content	AA	8.58E+00	8.35E-04
13	Potom.013G29538-SNP9	20	Potom.003G08321-SNP17	Holocellulose content	AA	9.20E+00	8.33E-04
1	Potom.001G00363-SNP23	16	Potom.016G34352-SNP98	Holocellulose content	AA	1.34E+01	8.32E-04
8	Potom.008G22699-SNP38	16	Potom.016G33891-SNP29	Stem volume	AA	-7.10E-01	8.32E-04
8	Potom.008G22699-SNP72	20	Potom.003G08321-SNP17	Stem volume	DA	-6.52E-01	8.28E-04
2	Potom.002G05522-SNP60	20	Potom.003G08321-SNP14	Stem volume	DD	-1.59E+00	8.26E-04
8	Potom.008G22699-SNP22	10	Potom.010G25398-SNP63	Stem volume	DD	1.58E+00	8.26E-04
2	Potom.002G05522-SNP54	2	Potom.002G07206-SNP24	Stem volume	AA	-3.63E-01	8.20E-04
8	Potom.008G21326-SNP69	20	Potom.003G09473-SNP11	Diameter at breast height	AA	-7.91E+00	8.20E-04
13	Potom.013G29538-SNP2	16	Potom.016G33876-SNP31	Stem volume	AD	5.18E-01	8.20E-04
13	Potom.013G29538-SNP7	16	Potom.016G33891-SNP29	Fiber length	AD	-1.98E-01	8.19E-04
2	Potom.002G07963-SNP152	5	Potom.005G13176-SNP26	α-cellulose content	DD	-3.30E+01	8.18E-04
5	Potom.005G13176-SNP2	10	Potom.010G25706-SNP45	α-cellulose content	DA	-1.44E+01	8.18E-04
2	Potom.002G05522-SNP4	5	Potom.005G13176-SNP40	Holocellulose content	AA	-9.61E+00	8.13E-04
2	Potom.002G05522-SNP23	20	Potom.003G08321-SNP20	Stem volume	AA	2.53E-01	8.13E-04
2	Potom.002G07337-SNP15	20	Potom.003G09473-SNP2	Fiber length	DD	1.62E-01	8.07E-04
8	Potom.008G22699-SNP74	16	Potom.016G33876-SNP72	α-cellulose content	DD	1.92E+01	8.04E-04
1	Potom.001G03193-SNP13	8	Potom.008G21952-SNP16	α-cellulose content	AA	1.46E+01	7.99E-04

2	Potom.002G05522-SNP62	20	Potom.003G08321-SNP14	Stem volume	DD	-1.59E+00	7.97E-04
1	Potom.001G00363-SNP54	5	Pto-Wuschela-SNP14	Hemicellulose content	AA	7.24E+00	7.93E-04
1	Potom.001G00363-SNP55	5	Pto-Wuschela-SNP14	Hemicellulose content	AA	7.24E+00	7.93E-04
5	Potom.005G13176-SNP115	16	Potom.016G33876-SNP25	Stem volume	DA	-1.12E+00	7.87E-04
8	Potom.008G22699-SNP98	20	Potom.003G08321-SNP17	Holocellulose content	DA	2.14E+01	7.84E-04
1	Potom.001G00363-SNP53	2	Potom.002G07337-SNP15	Holocellulose content	AD	2.22E+01	7.79E-04
2	Potom.002G05522-SNP4	5	Potom.005G13176-SNP62	Holocellulose content	AA	-8.96E+00	7.78E-04
5	Potom.005G13966-SNP97	20	Potom.003G09473-SNP9	Holocellulose content	DA	-2.45E+01	7.75E-04
1	Potom.001G03193-SNP20	8	Potom.008G21952-SNP16	Hemicellulose content	AA	-1.45E+01	7.71E-04
1	Potom.001G03193-SNP10	5	Potom.005G13176-SNP26	α-cellulose content	AD	1.33E+01	7.67E-04
8	Potom.008G22699-SNP71	10	Potom.010G25398-SNP26	Stem volume	DA	-6.65E-01	7.64E-04
20	Potom.003G08321-SNP21	20	Potom.003G09473-SNP13	Hemicellulose content	AD	9.07E+00	7.54E-04
1	Potom.001G03353-SNP24	8	Potom.008G22699-SNP22	Holocellulose content	DA	-2.42E+01	7.53E-04
1	Potom.001G00363-SNP21	5	Potom.005G13333-SNP13	Lignin content	DD	8.08E+00	7.51E-04
2	Potom.002G05522-SNP60	8	Potom.008G22699-SNP190	Holocellulose content	AD	-1.64E+01	7.49E-04
1	Potom.001G00363-SNP54	2	Potom.002G07337-SNP15	Holocellulose content	AD	2.24E+01	7.42E-04
1	Potom.001G00363-SNP55	2	Potom.002G07337-SNP15	Holocellulose content	AD	2.24E+01	7.42E-04
2	Potom.002G07337-SNP34	9	Potom.009G23017-SNP55	Hemicellulose content	DD	-2.69E+01	7.38E-04
1	Potom.001G01788-SNP70	16	Potom.016G33891-SNP28	Diameter at breast height	DD	-1.56E+01	7.36E-04
1	Potom.001G03193-SNP10	8	Potom.008G22699-SNP108	Hemicellulose content	DA	1.49E+01	7.36E-04
2	Potom.002G05522-SNP4	8	Potom.008G22699-SNP85	Holocellulose content	AD	2.94E+01	7.35E-04
2	Potom.002G05769-SNP70	8	Potom.008G21952-SNP16	Holocellulose content	AD	-1.86E+01	7.34E-04
1	Potom.001G00363-SNP21	16	Potom.016G34352-SNP98	Holocellulose content	AA	1.39E+01	7.31E-04
2	Potom.002G05522-SNP4	16	Potom.016G34352-SNP108	Hemicellulose content	DD	1.59E+01	7.17E-04
8	Potom.008G21326-SNP69	20	Potom.003G09473-SNP13	Diameter at breast height	AA	-8.02E+00	7.12E-04
2	Potom.002G05522-SNP62	8	Potom.008G22699-SNP190	Holocellulose content	AD	-1.65E+01	7.10E-04

5	Potom.005G13333-SNP18	10	Potom.010G25294-SNP6	Lignin content	DA	-1.21E+00	6.89E-04
5	Potom.005G13333-SNP18	10	Potom.010G25294-SNP7	Lignin content	DA	-1.21E+00	6.89E-04
2	Potom.002G07337-SNP229	8	Potom.008G22699-SNP3	Stem volume	DA	5.34E-01	6.87E-04
1	Potom.001G03193-SNP46	5	Pto-Wuschela-SNP5	Hemicellulose content	AA	5.51E+00	6.85E-04
1	Potom.001G00363-SNP26	20	Potom.003G08321-SNP14	Stem volume	AD	1.36E+00	6.79E-04
2	Potom.002G05522-SNP12	8	Potom.008G21952-SNP16	α-cellulose content	AA	1.37E+01	6.73E-04
2	Potom.002G05522-SNP62	12	Potom.012G28409-SNP23	Stem volume	DA	-3.77E-01	6.73E-04
2	Potom.002G05522-SNP1	12	Potom.012G28409-SNP11	Hemicellulose content	DA	-2.09E+01	6.67E-04
2	Potom.002G07337-SNP15	20	Potom.003G08321-SNP3	Holocellulose content	DD	2.11E+01	6.50E-04
8	Potom.008G22699-SNP71	8	Potom.008G22699-SNP72	Hemicellulose content	DA	-1.46E+01	6.45E-04
1	Potom.001G03193-SNP31	10	Potom.010G25398-SNP26	Stem volume	DD	-1.13E+00	6.44E-04
2	Potom.002G05522-SNP23	5	Potom.005G13966-SNP89	Stem volume	AD	-9.44E-01	6.40E-04
2	Potom.002G07337-SNP229	5	Potom.005G13176-SNP44	Stem volume	AA	-2.77E-01	6.40E-04
1	Potom.001G03193-SNP27	5	Potom.005G13966-SNP90	Stem volume	AD	-9.32E-01	6.38E-04
5	Potom.005G13966-SNP77	16	Potom.016G33891-SNP28	Diameter at breast height	AD	-1.00E+01	6.37E-04
2	Potom.002G05522-SNP60	12	Potom.012G28409-SNP23	Stem volume	DA	-3.77E-01	6.36E-04
5	Potom.005G13966-SNP77	16	Potom.016G33891-SNP6	Stem volume	AA	-2.99E-01	6.35E-04
8	Potom.008G22699-SNP9	8	Potom.008G22699-SNP72	Fiber width	AD	-4.64E+00	6.28E-04
8	Potom.008G21326-SNP69	16	Potom.016G33876-SNP55	Stem volume	AD	-6.02E-01	6.26E-04
1	Potom.001G03193-SNP20	5	Potom.005G13176-SNP2	Stem volume	DD	1.52E+00	6.24E-04
2	Potom.002G07963-SNP185	16	Potom.016G33891-SNP15	Microfiber angle	AD	2.88E+00	6.17E-04
5	Potom.005G13176-SNP27	8	Potom.008G22699-SNP3	Stem volume	AD	-6.66E-01	6.17E-04
5	Potom.005G13176-SNP27	5	Potom.005G13966-SNP97	Diameter at breast height	AA	-8.70E+00	6.12E-04
5	Pto-Wuschela-SNP12	14	Potom.014G31981-SNP26	Hemicellulose content	DA	-1.45E+01	6.10E-04
10	Potom.010G25398-SNP26	16	Potom.016G33876-SNP31	Holocellulose content	AD	1.55E+01	6.06E-04
1	Potom.001G00363-SNP19	2	Potom.002G05769-SNP43	Diameter at breast height	AA	-8.60E+00	6.01E-04

2	Potom.002G05522-SNP23	16	Potom.016G33876-SNP53	Diameter at breast height	AA	5.16E+00	6.00E-04
8	Potom.008G21326-SNP69	10	Potom.010G24967-SNP45	Microfiber angle	AA	2.95E+00	5.98E-04
16	Potom.016G34352-SNP109	20	Potom.003G09473-SNP2	Stem volume	AD	6.29E-01	5.96E-04
20	Potom.003G08321-SNP21	20	Potom.003G09473-SNP11	Hemicellulose content	AD	9.33E+00	5.94E-04
2	Potom.002G05522-SNP60	8	Potom.008G22699-SNP98	Holocellulose content	AD	-2.27E+01	5.93E-04
1	Potom.001G03193-SNP10	5	Potom.005G13176-SNP117	Hemicellulose content	AD	6.41E+00	5.92E-04
2	Potom.002G05522-SNP62	10	Potom.010G25398-SNP68	α-cellulose content	AD	2.39E+01	5.92E-04
1	Potom.001G03353-SNP164	14	Potom.014G31981-SNP6	Diameter at breast height	DD	-1.13E+01	5.87E-04
1	Potom.001G03193-SNP46	2	Potom.002G05522-SNP4	Holocellulose content	AA	-7.62E+00	5.85E-04
2	Potom.002G05522-SNP15	2	Potom.002G05522-SNP54	Diameter at breast height	AA	-7.87E+00	5.85E-04
1	Potom.001G03193-SNP10	8	Potom.008G21326-SNP55	Hemicellulose content	DD	-2.54E+01	5.81E-04
2	Potom.002G05522-SNP60	10	Potom.010G25398-SNP68	α-cellulose content	AD	2.39E+01	5.79E-04
1	Potom.001G00363-SNP19	8	Potom.008G21952-SNP16	Holocellulose content	AD	2.18E+01	5.74E-04
16	Potom.016G33876-SNP72	20	Potom.003G09473-SNP2	Microfiber angle	AD	-6.72E+00	5.70E-04
2	Potom.002G05522-SNP12	2	Potom.002G07337-SNP15	Fiber width	DD	-4.43E+00	5.65E-04
2	Potom.002G05522-SNP62	8	Potom.008G22699-SNP98	Holocellulose content	AD	-2.28E+01	5.65E-04
2	Potom.002G05769-SNP43	8	Potom.008G21952-SNP16	Diameter at breast height	AA	-8.62E+00	5.60E-04
2	Potom.002G05769-SNP13	2	Potom.002G07206-SNP24	Hemicellulose content	DA	-2.08E+01	5.59E-04
2	Potom.002G05522-SNP23	5	Potom.005G13966-SNP77	Diameter at breast height	DA	-3.69E+00	5.54E-04
2	Potom.002G05522-SNP1	2	Potom.002G07963-SNP231	Hemicellulose content	DA	-2.19E+01	5.53E-04
10	Potom.010G25398-SNP26	13	Potom.013G29538-SNP72	Lignin content	AD	-3.29E+00	5.50E-04
16	Potom.016G33891-SNP28	16	Potom.016G34352-SNP108	Stem volume	DD	-1.07E+00	5.45E-04
13	Potom.013G29538-SNP5	20	Potom.003G09473-SNP2	Fiber length	DD	1.70E-01	5.44E-04
1	Potom.001G03193-SNP10	20	Potom.003G08321-SNP3	Hemicellulose content	DD	-2.46E+01	5.36E-04
10	Potom.010G25398-SNP68	13	Potom.013G29538-SNP5	α-cellulose content	DA	2.30E+01	5.34E-04
2	Potom.002G05522-SNP15	5	Potom.005G13176-SNP284	Microfiber angle	AD	-7.81E+00	5.33E-04

5	Potom.005G13966-SNP89	8	Potom.008G22699-SNP9	Stem volume	DA	-1.05E+00	5.32E-04
10	Potom.010G25700-SNP13	13	Potom.013G29538-SNP71	Fiber width	AD	-4.91E+00	5.32E-04
5	Potom.005G13176-SNP47	5	Potom.005G13333-SNP13	Hemicellulose content	AD	-8.66E+00	5.29E-04
8	Potom.008G22699-SNP72	20	Potom.003G08321-SNP17	Stem volume	DD	9.16E-01	5.22E-04
5	Potom.005G13985-SNP17	8	Potom.008G22699-SNP98	Stem volume	AA	-8.50E-01	5.17E-04
8	Potom.008G21952-SNP16	16	Potom.016G33891-SNP6	Stem volume	AA	-6.81E-01	5.02E-04
5	Potom.005G13966-SNP89	16	Potom.016G34352-SNP109	Holocellulose content	AD	1.86E+01	5.00E-04
10	Potom.010G25398-SNP68	13	Potom.013G29538-SNP72	Holocellulose content	DD	3.98E+01	4.98E-04
1	Potom.001G03606-SNP26	12	Potom.012G28819-SNP27	Fiber length	AA	1.41E-01	4.95E-04
5	Potom.005G13966-SNP77	10	Potom.010G25700-SNP13	Stem volume	AA	-4.31E-01	4.94E-04
2	Potom.002G07206-SNP14	8	Potom.008G22516-SNP43	Fiber width	DA	4.53E+00	4.85E-04
5	Pto-Wuschela-SNP14	5	Potom.005G13176-SNP16	Hemicellulose content	DA	-1.44E+01	4.85E-04
2	Potom.002G05522-SNP54	8	Potom.008G21952-SNP16	Stem volume	AA	-5.48E-01	4.83E-04
2	Potom.002G05522-SNP15	8	Potom.008G21952-SNP16	α-cellulose content	DA	-1.61E+01	4.82E-04
5	Potom.005G13176-SNP27	18	Potom.018G35976-SNP13	Stem volume	AD	-7.64E-01	4.82E-04
8	Potom.008G21952-SNP16	13	Potom.013G29538-SNP71	Fiber width	AD	-3.68E+00	4.77E-04
2	Potom.002G05522-SNP15	8	Potom.008G21952-SNP16	α-cellulose content	AA	1.67E+01	4.69E-04
2	Potom.002G05522-SNP13	8	Potom.008G22699-SNP72	Stem volume	DA	5.96E-01	4.65E-04
2	Potom.002G05522-SNP14	8	Potom.008G22699-SNP72	Stem volume	DA	5.96E-01	4.65E-04
8	Potom.008G21952-SNP16	16	Potom.016G33891-SNP6	Holocellulose content	DA	-2.15E+01	4.61E-04
5	Potom.005G13966-SNP90	16	Potom.016G33876-SNP31	Stem volume	DD	-1.20E+00	4.59E-04
2	Potom.002G05522-SNP54	10	Potom.010G24967-SNP27	Fiber length	AA	-9.51E-02	4.58E-04
2	Potom.002G05769-SNP72	8	Potom.008G21952-SNP16	Holocellulose content	AD	-2.05E+01	4.56E-04
1	Potom.001G03193-SNP20	5	Pto-Wuschela-SNP5	Holocellulose content	AA	8.77E+00	4.54E-04
10	Potom.010G24967-SNP67	16	Potom.016G33891-SNP28	Diameter at breast height	AA	-4.55E+00	4.54E-04
8	Potom.008G22699-SNP72	16	Potom.016G33891-SNP28	Stem volume	AD	7.86E-01	4.53E-04

5	Potom.005G13176-SNP27	8	Potom.008G22699-SNP38	Stem volume	AA	-8.12E-01	4.52E-04
5	Potom.005G13333-SNP13	12	Potom.012G28409-SNP23	Stem volume	AD	-6.29E-01	4.48E-04
10	Potom.010G25706-SNP89	13	Potom.013G29538-SNP5	Stem volume	AA	2.38E-01	4.43E-04
2	Potom.002G07337-SNP1	5	Pto-Wuschela-SNP10	Fiber width	DA	2.12E+00	4.39E-04
1	Potom.001G00363-SNP26	20	Potom.003G08321-SNP13	Stem volume	AD	1.35E+00	4.38E-04
2	Potom.002G05522-SNP1	5	Potom.005G13985-SNP1	Hemicellulose content	DA	-2.43E+01	4.38E-04
2	Potom.002G05522-SNP1	5	Potom.005G13985-SNP2	Hemicellulose content	DA	-2.43E+01	4.38E-04
8	Potom.008G22699-SNP72	20	Potom.003G09473-SNP13	Stem volume	DD	-1.02E+00	4.38E-04
5	Potom.005G13966-SNP74	16	Potom.016G34352-SNP109	Holocellulose content	AD	1.92E+01	4.37E-04
1	Potom.001G00363-SNP19	8	Potom.008G21952-SNP16	α-cellulose content	AD	1.88E+01	4.34E-04
8	Potom.008G21326-SNP69	16	Potom.016G33876-SNP56	Stem volume	AD	-5.72E-01	4.34E-04
5	Potom.005G13176-SNP27	5	Potom.005G13966-SNP97	Holocellulose content	AD	-1.92E+01	4.30E-04
1	Potom.001G03606-SNP29	10	Potom.010G25398-SNP68	Diameter at breast height	DD	-2.57E+01	4.29E-04
2	Potom.002G05522-SNP54	8	Potom.008G22699-SNP72	Stem volume	AD	7.09E-01	4.29E-04
1	Potom.001G03193-SNP13	5	Potom.005G13333-SNP13	Stem volume	DA	1.05E+00	4.26E-04
1	Potom.001G03193-SNP10	5	Potom.005G13176-SNP62	Hemicellulose content	DD	-2.13E+01	4.25E-04
1	Potom.001G03193-SNP26	2	Potom.002G05522-SNP23	Diameter at breast height	DD	-1.28E+01	4.24E-04
2	Potom.002G07337-SNP229	10	Potom.010G25398-SNP63	Stem volume	AD	1.04E+00	4.24E-04
12	Potom.012G28819-SNP27	20	Potom.003G09473-SNP2	Microfiber angle	DA	8.84E+00	4.23E-04
5	Potom.005G13176-SNP44	16	Potom.016G33891-SNP13	Fiber length	AD	1.37E-01	4.22E-04
10	Potom.010G25706-SNP89	13	Potom.013G29538-SNP8	Stem volume	AD	6.38E-01	4.21E-04
2	Potom.002G05522-SNP13	20	Potom.003G08321-SNP1	Stem volume	DA	1.11E+00	4.19E-04
2	Potom.002G05522-SNP14	20	Potom.003G08321-SNP1	Stem volume	DA	1.11E+00	4.19E-04
13	Potom.013G29538-SNP5	20	Potom.003G08321-SNP13	Holocellulose content	DA	2.24E+01	4.18E-04
5	Potom.005G13966-SNP97	13	Potom.013G29538-SNP7	Stem volume	DA	7.64E-01	4.11E-04
13	Potom.013G29538-SNP5	20	Potom.003G08321-SNP14	Stem volume	DD	-1.60E+00	4.11E-04

2	Potom.002G07206-SNP24	20	Potom.003G09473-SNP2	Fiber length	DD	1.78E-01	4.10E-04
2	Potom.002G05522-SNP1	10	Potom.010G25398-SNP63	Hemicellulose content	DA	2.65E+01	3.97E-04
8	Potom.008G22699-SNP69	20	Potom.003G09473-SNP2	Fiber length	AD	1.66E-01	3.92E-04
8	Potom.008G22699-SNP9	20	Potom.003G09473-SNP11	Stem volume	AD	-9.09E-01	3.88E-04
1	Potom.001G03193-SNP31	10	Potom.010G25294-SNP212	Stem volume	DD	-1.16E+00	3.87E-04
2	Potom.002G07337-SNP229	5	Potom.005G13176-SNP68	Stem volume	DD	-1.04E+00	3.83E-04
8	Potom.008G22699-SNP9	20	Potom.003G09473-SNP13	Stem volume	AD	-9.18E-01	3.82E-04
10	Potom.010G25706-SNP89	13	Potom.013G29538-SNP6	Stem volume	AD	6.38E-01	3.81E-04
13	Potom.013G29538-SNP8	20	Potom.003G08321-SNP18	Stem volume	AA	-3.56E-01	3.74E-04
8	Potom.008G21326-SNP69	10	Potom.010G24967-SNP43	Microfiber angle	AA	3.21E+00	3.70E-04
5	Potom.005G13176-SNP3	20	Potom.003G08321-SNP17	α-cellulose content	DA	-1.41E+01	3.69E-04
5	Potom.005G13966-SNP90	10	Potom.010G25398-SNP63	Holocellulose content	AA	1.94E+01	3.69E-04
1	Potom.001G03193-SNP20	5	Pto-Wuschela-SNP10	Hemicellulose content	AD	-1.16E+01	3.67E-04
5	Pto-Wuschela-SNP14	9	Potom.009G23029-SNP3	Hemicellulose content	DA	-1.16E+01	3.67E-04
1	Potom.001G03193-SNP27	8	Potom.008G21952-SNP16	α-cellulose content	AA	1.49E+01	3.66E-04
5	Potom.005G13966-SNP89	13	Potom.013G29538-SNP2	Stem volume	DA	-7.18E-01	3.66E-04
2	Potom.002G05522-SNP23	20	Potom.003G09473-SNP2	Fiber length	AD	1.19E-01	3.65E-04
8	Potom.008G22699-SNP9	8	Potom.008G22699-SNP72	Stem volume	DD	1.19E+00	3.63E-04
1	Potom.001G03193-SNP26	8	Potom.008G21326-SNP69	Diameter at breast height	AD	-1.07E+01	3.52E-04
5	Potom.005G13176-SNP62	8	Potom.008G22699-SNP3	Diameter at breast height	DD	-9.48E+00	3.52E-04
13	Potom.013G29538-SNP6	20	Potom.003G08321-SNP18	Stem volume	AA	-3.55E-01	3.49E-04
2	Potom.002G05769-SNP43	2	Potom.002G07963-SNP163	Fiber length	AD	-1.55E-01	3.44E-04
2	Potom.002G07337-SNP229	16	Potom.016G33876-SNP55	Stem volume	DA	4.23E-01	3.36E-04
1	Potom.001G03606-SNP29	10	Potom.010G25398-SNP68	Stem volume	DD	-1.83E+00	3.35E-04
16	Potom.016G33891-SNP28	20	Potom.003G09473-SNP13	Stem volume	DD	-1.16E+00	3.31E-04
8	Potom.008G22699-SNP9	20	Potom.003G09473-SNP2	Microfiber angle	AD	-8.17E+00	3.30E-04

8	Potom.008G22699-SNP72	20	Potom.003G09473-SNP11	Stem volume	DD	-1.03E+00	3.30E-04
2	Potom.002G05769-SNP43	20	Potom.003G08321-SNP32	Diameter at breast height	AA	6.77E+00	3.28E-04
10	Potom.010G24967-SNP67	16	Potom.016G33891-SNP28	Stem volume	AD	-6.52E-01	3.26E-04
2	Potom.002G05522-SNP13	2	Potom.002G07337-SNP15	Holocellulose content	AD	2.12E+01	3.22E-04
2	Potom.002G05522-SNP14	2	Potom.002G07337-SNP15	Holocellulose content	AD	2.12E+01	3.22E-04
8	Potom.008G22699-SNP71	13	Potom.013G29538-SNP72	Stem volume	DA	-9.40E-01	3.22E-04
2	Potom.002G05522-SNP4	5	Potom.005G13176-SNP47	Holocellulose content	AA	-8.39E+00	3.16E-04
2	Potom.002G05522-SNP13	8	Potom.008G22699-SNP71	Holocellulose content	AA	1.61E+01	3.16E-04
2	Potom.002G05522-SNP14	8	Potom.008G22699-SNP71	Holocellulose content	AA	1.61E+01	3.16E-04
13	Potom.013G29538-SNP7	16	Potom.016G33891-SNP29	Stem volume	AA	-5.23E-01	3.16E-04
1	Potom.001G00363-SNP31	13	Potom.013G29538-SNP14	Holocellulose content	AA	2.23E+01	3.15E-04
1	Potom.001G01788-SNP70	16	Potom.016G33876-SNP59	Stem volume	DD	1.79E+00	3.09E-04
1	Potom.001G01788-SNP70	16	Potom.016G33876-SNP60	Stem volume	DD	1.79E+00	3.09E-04
5	Potom.005G13176-SNP162	8	Potom.008G22699-SNP108	Fiber length	DD	1.43E-01	3.09E-04
16	Potom.016G34352-SNP109	20	Potom.003G08321-SNP20	Stem volume	DD	-9.62E-01	3.09E-04
2	Potom.002G05769-SNP72	8	Potom.008G21952-SNP16	Stem volume	AA	-5.91E-01	3.07E-04
8	Potom.008G22699-SNP74	13	Potom.013G29538-SNP9	Stem volume	AD	5.05E-01	3.05E-04
2	Potom.002G05769-SNP43	20	Potom.003G08321-SNP32	Stem volume	AA	4.75E-01	3.04E-04
5	Potom.005G13966-SNP74	8	Potom.008G21952-SNP16	α-cellulose content	AA	1.54E+01	3.00E-04
8	Potom.008G21952-SNP16	13	Potom.013G29538-SNP71	α-cellulose content	DD	2.94E+01	3.00E-04
2	Potom.002G05522-SNP15	13	Potom.013G29538-SNP8	Stem volume	AD	-7.66E-01	2.97E-04
8	Potom.008G21952-SNP16	16	Potom.016G33891-SNP6	Diameter at breast height	AA	-1.03E+01	2.96E-04
16	Potom.016G33891-SNP28	20	Potom.003G08321-SNP32	Diameter at breast height	AA	9.36E+00	2.96E-04
16	Potom.016G33891-SNP28	20	Potom.003G09473-SNP11	Stem volume	DD	-1.15E+00	2.96E-04
5	Potom.005G13966-SNP90	8	Potom.008G22699-SNP72	Stem volume	AA	-5.72E-01	2.88E-04
2	Potom.002G05522-SNP1	5	Pto-Wuschela-SNP5	Hemicellulose content	DA	-1.82E+01	2.86E-04

5	Pto-Wuschela-SNP12	14	Potom.014G31981-SNP25	Hemicellulose content	DA	-1.56E+01	2.81E-04
5	Pto-Wuschela-SNP14	9	Potom.009G23029-SNP3	Hemicellulose content	AA	9.22E+00	2.80E-04
12	Potom.012G28819-SNP45	20	Potom.003G08321-SNP7	Diameter at breast height	AA	9.51E+00	2.78E-04
1	Potom.001G03193-SNP10	5	Potom.005G13176-SNP26	Hemicellulose content	AD	-1.33E+01	2.77E-04
2	Potom.002G07337-SNP56	5	Potom.005G13176-SNP15	Holocellulose content	AD	2.32E+01	2.74E-04
1	Potom.001G03193-SNP31	5	Potom.005G13333-SNP12	Tree height	AA	-4.23E+00	2.70E-04
5	Potom.005G13176-SNP2	10	Potom.010G25706-SNP89	Stem volume	DD	-1.17E+00	2.70E-04
10	Potom.010G25706-SNP89	20	Potom.003G08321-SNP20	Fiber width	DA	-2.95E+00	2.70E-04
2	Potom.002G05522-SNP15	13	Potom.013G29538-SNP6	Stem volume	AD	-7.65E-01	2.68E-04
5	Potom.005G13966-SNP89	20	Potom.003G10089-SNP11	Stem volume	DA	-1.09E+00	2.68E-04
10	Potom.010G25706-SNP89	13	Potom.013G29538-SNP117	Stem volume	AA	2.90E-01	2.67E-04
5	Potom.005G13333-SNP13	8	Potom.008G22699-SNP74	Diameter at breast height	DA	6.81E+00	2.62E-04
2	Potom.002G07337-SNP229	16	Potom.016G33876-SNP59	Stem volume	AD	1.35E+00	2.57E-04
2	Potom.002G07337-SNP229	16	Potom.016G33876-SNP60	Stem volume	AD	1.35E+00	2.57E-04
2	Potom.002G05522-SNP60	8	Potom.008G22699-SNP108	Fiber length	DA	-1.01E-01	2.56E-04
2	Potom.002G05522-SNP12	10	Potom.010G25398-SNP63	Stem volume	AD	1.22E+00	2.54E-04
12	Potom.012G28819-SNP27	20	Potom.003G09473-SNP12	Fiber length	DA	-2.54E-01	2.52E-04
20	Potom.003G08321-SNP13	20	Potom.003G08321-SNP20	Stem volume	DD	-1.71E+00	2.48E-04
5	Pto-Wuschela-SNP12	10	Potom.010G25700-SNP5	Hemicellulose content	DA	-1.90E+01	2.46E-04
5	Pto-Wuschela-SNP12	5	Potom.005G13176-SNP16	Hemicellulose content	DA	-1.17E+01	2.44E-04
2	Potom.002G05522-SNP62	8	Potom.008G22699-SNP108	Fiber length	DA	-1.01E-01	2.43E-04
2	Potom.002G05522-SNP73	8	Potom.008G21952-SNP16	Holocellulose content	AD	-1.11E+01	2.41E-04
2	Potom.002G05522-SNP12	13	Potom.013G29538-SNP8	Stem volume	AD	-7.82E-01	2.37E-04
5	Potom.005G13966-SNP89	13	Potom.013G29538-SNP10	Stem volume	DA	-9.67E-01	2.32E-04
2	Potom.002G05522-SNP4	5	Potom.005G13176-SNP162	Holocellulose content	AA	-8.73E+00	2.31E-04
2	Potom.002G05522-SNP23	5	Potom.005G13966-SNP89	Stem volume	DD	-1.40E+00	2.30E-04

10	Potom.010G24967-SNP67	16	Potom.016G33876-SNP25	Stem volume	AA	-7.21E-01	2.27E-04
13	Potom.013G29538-SNP9	20	Potom.003G08321-SNP18	Stem volume	AA	-3.33E-01	2.24E-04
10	Potom.010G25398-SNP63	20	Potom.003G08321-SNP4	Holocellulose content	AA	2.20E+01	2.23E-04
5	Potom.005G13333-SNP12	8	Potom.008G22699-SNP74	α-cellulose content	DA	-8.17E+00	2.21E-04
1	Potom.001G03193-SNP13	16	Potom.016G33891-SNP29	Stem volume	DA	1.08E+00	2.20E-04
2	Potom.002G05522-SNP23	5	Potom.005G13966-SNP77	Stem volume	DA	-2.77E-01	2.19E-04
2	Potom.002G05522-SNP13	8	Potom.008G22516-SNP12	Holocellulose content	AA	2.33E+01	2.18E-04
2	Potom.002G05522-SNP14	8	Potom.008G22516-SNP12	Holocellulose content	AA	2.33E+01	2.18E-04
1	Potom.001G03606-SNP27	20	Potom.003G08321-SNP14	Stem volume	AD	1.37E+00	2.15E-04
2	Potom.002G05522-SNP12	13	Potom.013G29538-SNP6	Stem volume	AD	-7.81E-01	2.14E-04
5	Potom.005G13966-SNP90	10	Potom.010G25398-SNP26	Stem volume	DA	-1.22E+00	2.14E-04
5	Pto-Wuschela-SNP14	14	Potom.014G31981-SNP26	Holocellulose content	DA	-1.93E+01	2.10E-04
1	Potom.001G03193-SNP10	2	Potom.002G07337-SNP34	Hemicellulose content	DA	-1.33E+01	2.09E-04
1	Potom.001G00363-SNP53	5	Pto-Wuschela-SNP14	Hemicellulose content	AA	7.35E+00	2.07E-04
12	Potom.012G28819-SNP45	20	Potom.003G08321-SNP6	Diameter at breast height	AA	9.71E+00	2.07E-04
2	Potom.002G05522-SNP62	10	Potom.010G25398-SNP68	Stem volume	AD	-1.20E+00	2.06E-04
1	Potom.001G00363-SNP23	5	Potom.005G13333-SNP13	Lignin content	DD	8.32E+00	2.04E-04
2	Potom.002G07337-SNP229	20	Potom.003G09473-SNP13	Stem volume	DD	-1.15E+00	2.02E-04
2	Potom.002G05522-SNP60	10	Potom.010G25398-SNP68	Stem volume	AD	-1.21E+00	2.00E-04
5	Potom.005G13966-SNP97	20	Potom.003G09473-SNP2	Fiber length	AD	1.46E-01	1.99E-04
16	Potom.016G33876-SNP108	16	Potom.016G33891-SNP28	Stem volume	AD	-6.60E-01	1.98E-04
1	Potom.001G03193-SNP13	5	Potom.005G13333-SNP12	Stem volume	DA	9.66E-01	1.97E-04
2	Potom.002G05769-SNP43	5	Potom.005G13966-SNP77	Stem volume	AA	-3.06E-01	1.97E-04
2	Potom.002G07337-SNP229	16	Potom.016G33891-SNP28	Diameter at breast height	AD	-1.03E+01	1.95E-04
5	Potom.005G13966-SNP97	20	Potom.003G09473-SNP2	Stem volume	DA	7.88E-01	1.95E-04
1	Potom.001G03193-SNP10	20	Potom.003G10089-SNP11	Hemicellulose content	DA	-1.89E+01	1.93E-04

10	Potom.010G25706-SNP89	13	Potom.013G29538-SNP9	Stem volume	AD	6.20E-01	1.91E-04
13	Potom.013G29538-SNP7	20	Potom.003G08321-SNP18	Stem volume	AA	-3.35E-01	1.91E-04
16	Potom.016G33876-SNP59	20	Potom.003G09473-SNP2	Stem volume	DA	-1.41E+00	1.88E-04
16	Potom.016G33876-SNP60	20	Potom.003G09473-SNP2	Stem volume	DA	-1.41E+00	1.88E-04
2	Potom.002G05769-SNP57	16	Potom.016G33891-SNP15	Fiber width	AD	4.61E+00	1.83E-04
2	Potom.002G05522-SNP62	2	Potom.002G07337-SNP229	Stem volume	AA	-2.69E-01	1.82E-04
5	Potom.005G13176-SNP44	12	Potom.012G28409-SNP23	Stem volume	AA	-4.29E-01	1.80E-04
8	Potom.008G22699-SNP85	10	Potom.010G25398-SNP26	Holocellulose content	DD	-4.47E+01	1.80E-04
2	Potom.002G05769-SNP13	2	Potom.002G07206-SNP24	Holocellulose content	DA	-2.43E+01	1.72E-04
2	Potom.002G05522-SNP23	5	Potom.005G13966-SNP90	Stem volume	DD	-1.26E+00	1.70E-04
2	Potom.002G07337-SNP229	20	Potom.003G09473-SNP11	Stem volume	DD	-1.14E+00	1.67E-04
8	Potom.008G22699-SNP108	16	Potom.016G33891-SNP17	α-cellulose content	AD	1.14E+01	1.64E-04
10	Potom.010G25398-SNP26	20	Potom.003G08321-SNP13	Stem volume	AD	-1.32E+00	1.64E-04
2	Potom.002G05522-SNP12	8	Potom.008G22699-SNP71	Stem volume	AD	7.90E-01	1.63E-04
5	Potom.005G13176-SNP16	10	Potom.010G24967-SNP67	Diameter at breast height	DA	-3.98E+00	1.62E-04
2	Potom.002G05522-SNP4	2	Potom.002G05522-SNP12	Holocellulose content	AD	-1.10E+01	1.60E-04
10	Potom.010G25706-SNP89	13	Potom.013G29538-SNP7	Stem volume	AD	6.31E-01	1.60E-04
2	Potom.002G05522-SNP60	20	Potom.003G08321-SNP13	Stem volume	DD	-1.62E+00	1.59E-04
2	Potom.002G05522-SNP62	20	Potom.003G08321-SNP13	Stem volume	DD	-1.62E+00	1.56E-04
2	Potom.002G05522-SNP1	10	Potom.010G25706-SNP46	Hemicellulose content	DA	1.91E+01	1.51E-04
16	Potom.016G33876-SNP72	20	Potom.003G09473-SNP2	Fiber length	AD	1.45E-01	1.46E-04
16	Potom.016G33876-SNP108	16	Potom.016G33891-SNP28	Diameter at breast height	AA	-7.22E+00	1.45E-04
8	Potom.008G22699-SNP71	20	Potom.003G09473-SNP2	Fiber length	AD	1.66E-01	1.44E-04
5	Potom.005G13966-SNP90	10	Potom.010G25398-SNP68	Holocellulose content	AA	1.95E+01	1.42E-04
2	Potom.002G05522-SNP1	10	Potom.010G25706-SNP45	Hemicellulose content	DA	1.91E+01	1.41E-04
2	Potom.002G05522-SNP60	2	Potom.002G07337-SNP229	Stem volume	AA	-2.82E-01	1.41E-04

2	Potom.002G05522-SNP4	5	Potom.005G13176-SNP157	Holocellulose content	AA	-1.00E+01	1.40E-04
5	Potom.005G13966-SNP89	20	Potom.003G08321-SNP17	Stem volume	DA	9.62E-01	1.38E-04
5	Potom.005G13176-SNP16	10	Potom.010G24967-SNP67	Stem volume	DA	-2.80E-01	1.37E-04
2	Potom.002G05522-SNP23	10	Potom.010G24967-SNP67	Stem volume	AA	-3.18E-01	1.34E-04
10	Potom.010G25398-SNP26	20	Potom.003G09473-SNP10	Holocellulose content	DA	2.77E+01	1.29E-04
5	Pto-Wuschela-SNP14	14	Potom.014G31981-SNP25	Holocellulose content	DA	-2.01E+01	1.27E-04
18	Potom.018G35976-SNP25	20	Potom.003G09473-SNP2	Fiber length	AD	1.30E-01	1.26E-04
10	Potom.010G24967-SNP27	16	Potom.016G33891-SNP28	Stem volume	AD	-7.75E-01	1.24E-04
10	Potom.010G24967-SNP43	16	Potom.016G33891-SNP28	Stem volume	DD	-1.16E+00	1.21E-04
1	Potom.001G00363-SNP19	2	Potom.002G05522-SNP23	Diameter at breast height	AD	-9.87E+00	1.20E-04
16	Potom.016G33891-SNP17	20	Potom.003G08321-SNP26	Diameter at breast height	AD	-1.11E+01	1.18E-04
2	Potom.002G07337-SNP15	20	Potom.003G09473-SNP2	α-cellulose content	AA	-6.87E+00	1.16E-04
5	Potom.005G13333-SNP12	12	Potom.012G28409-SNP23	Stem volume	AD	-7.83E-01	1.16E-04
10	Potom.010G25398-SNP63	16	Potom.016G33891-SNP29	Stem volume	DA	1.16E+00	1.13E-04
8	Potom.008G22699-SNP108	16	Potom.016G33891-SNP17	α-cellulose content	DA	-1.60E+01	1.10E-04
10	Potom.010G25398-SNP68	13	Potom.013G29538-SNP5	Stem volume	DA	-1.23E+00	1.10E-04
13	Potom.013G29538-SNP9	20	Potom.003G08321-SNP17	Stem volume	AA	-4.23E-01	1.08E-04
1	Potom.001G03353-SNP161	5	Potom.005G13966-SNP89	Stem volume	DD	-1.34E+00	1.07E-04
2	Potom.002G05522-SNP62	10	Potom.010G25398-SNP68	Holocellulose content	AD	2.75E+01	1.04E-04
10	Potom.010G24967-SNP45	16	Potom.016G33891-SNP28	Stem volume	DD	-1.16E+00	1.04E-04
1	Potom.001G03193-SNP13	2	Potom.002G05522-SNP15	Stem volume	DA	1.24E+00	1.03E-04
2	Potom.002G07337-SNP229	16	Potom.016G33876-SNP56	Stem volume	DA	4.75E-01	1.03E-04
2	Potom.002G05522-SNP60	10	Potom.010G25398-SNP68	Holocellulose content	AD	2.75E+01	9.97E-05
8	Potom.008G22699-SNP22	20	Potom.003G08321-SNP20	Stem volume	DD	-1.30E+00	9.80E-05
1	Potom.001G03193-SNP20	2	Potom.002G05522-SNP23	Diameter at breast height	DD	-1.33E+01	9.34E-05
1	Potom.001G00363-SNP19	2	Potom.002G05769-SNP57	Stem volume	AA	-7.72E-01	9.23E-05

2	Potom.002G05522-SNP4	5	Potom.005G13176-SNP16	Holocellulose content	AA	-7.92E+00	9.21E-05
2	Potom.002G05522-SNP15	2	Potom.002G05522-SNP54	Stem volume	DA	5.81E-01	9.14E-05
5	Potom.005G13176-SNP27	20	Potom.003G08321-SNP13	Stem volume	DD	-1.89E+00	9.09E-05
1	Potom.001G00363-SNP19	2	Potom.002G05522-SNP4	Holocellulose content	AA	-1.31E+01	8.86E-05
2	Potom.002G05522-SNP54	8	Potom.008G21952-SNP16	Holocellulose content	AD	-1.79E+01	8.43E-05
2	Potom.002G05522-SNP23	10	Potom.010G24967-SNP67	Diameter at breast height	AA	-4.74E+00	8.36E-05
8	Potom.008G22699-SNP70	8	Potom.008G22699-SNP108	Fiber length	AD	1.44E-01	8.07E-05
10	Potom.010G25398-SNP26	20	Potom.003G09473-SNP12	Holocellulose content	DA	2.77E+01	8.07E-05
8	Potom.008G21326-SNP69	16	Potom.016G33891-SNP28	Diameter at breast height	AA	-8.31E+00	7.78E-05
2	Potom.002G05522-SNP60	8	Potom.008G22699-SNP71	Stem volume	DD	-1.10E+00	7.63E-05
2	Potom.002G05522-SNP62	8	Potom.008G22699-SNP71	Stem volume	DD	-1.11E+00	7.41E-05
2	Potom.002G05522-SNP12	5	Potom.005G13966-SNP90	Stem volume	AD	8.15E-01	7.36E-05
5	Potom.005G13966-SNP89	16	Potom.016G33876-SNP94	Stem volume	DA	-9.63E-01	7.19E-05
13	Potom.013G29538-SNP7	20	Potom.003G08321-SNP17	Stem volume	AA	-4.28E-01	7.08E-05
1	Potom.001G03606-SNP27	20	Potom.003G08321-SNP13	Stem volume	AD	1.43E+00	6.45E-05
1	Potom.001G03193-SNP13	10	Potom.010G25706-SNP89	Stem volume	DA	-9.42E-01	6.13E-05
5	Potom.005G13966-SNP89	10	Potom.010G25398-SNP26	Stem volume	DA	-1.19E+00	5.94E-05
10	Potom.010G25398-SNP26	10	Potom.010G25398-SNP63	Holocellulose content	DA	2.34E+01	5.49E-05
16	Potom.016G34352-SNP108	20	Potom.003G08321-SNP20	Stem volume	DD	-1.09E+00	5.47E-05
5	Potom.005G13966-SNP89	8	Potom.008G22699-SNP74	Stem volume	DA	-8.26E-01	5.18E-05
2	Potom.002G07337-SNP229	5	Potom.005G13966-SNP90	Stem volume	AD	1.01E+00	5.11E-05
20	Potom.003G08321-SNP10	20	Potom.003G09473-SNP2	Fiber length	DD	2.42E-01	4.93E-05
1	Potom.001G03353-SNP25	5	Potom.005G13966-SNP90	Stem volume	DD	1.84E+00	4.89E-05
5	Potom.005G13966-SNP97	10	Potom.010G25398-SNP63	Holocellulose content	AA	1.73E+01	4.48E-05
8	Potom.008G21952-SNP16	20	Potom.003G10089-SNP7	Fiber width	AD	7.33E+00	4.04E-05
1	Potom.001G03193-SNP20	2	Potom.002G05522-SNP4	Holocellulose content	DA	-1.34E+01	4.01E-05

1	Potom.001G03193-SNP46	2	Potom.002G05522-SNP4	Holocellulose content	DA	-1.82E+01	3.88E-05
1	Potom.001G03606-SNP29	20	Potom.003G08321-SNP14	Stem volume	AD	1.48E+00	3.88E-05
5	Potom.005G13176-SNP44	12	Potom.012G28409-SNP23	Stem volume	DA	-4.73E-01	3.88E-05
2	Potom.002G05522-SNP60	20	Potom.003G09473-SNP12	Holocellulose content	AA	1.61E+01	3.59E-05
5	Potom.005G13176-SNP26	20	Potom.003G08321-SNP18	Hemicellulose content	DA	1.36E+01	3.32E-05
5	Potom.005G13966-SNP89	16	Potom.016G33876-SNP31	Stem volume	DD	-1.28E+00	3.21E-05
1	Potom.001G01788-SNP70	16	Potom.016G33891-SNP28	Stem volume	DD	-1.30E+00	3.19E-05
2	Potom.002G05522-SNP62	20	Potom.003G09473-SNP12	Holocellulose content	AA	1.62E+01	3.10E-05
10	Potom.010G24967-SNP67	16	Potom.016G33891-SNP28	Stem volume	AA	-3.51E-01	3.05E-05
5	Potom.005G13966-SNP89	10	Potom.010G25706-SNP89	Stem volume	DA	-8.57E-01	2.93E-05
8	Potom.008G22699-SNP72	16	Potom.016G33891-SNP29	Stem volume	AA	-8.06E-01	2.48E-05
5	Potom.005G13333-SNP18	14	Potom.014G31981-SNP26	Fiber width	DD	6.27E+00	2.47E-05
2	Potom.002G05522-SNP60	20	Potom.003G09473-SNP10	Holocellulose content	AA	1.67E+01	2.41E-05
2	Potom.002G05522-SNP54	8	Potom.008G21952-SNP16	α-cellulose content	AD	-1.66E+01	2.28E-05
8	Potom.008G22699-SNP3	8	Potom.008G22699-SNP72	Stem volume	DD	1.02E+00	2.14E-05
2	Potom.002G05522-SNP62	20	Potom.003G09473-SNP10	Holocellulose content	AA	1.69E+01	2.08E-05
1	Potom.001G00363-SNP19	2	Potom.002G05769-SNP57	Diameter at breast height	AA	-1.30E+01	2.02E-05
5	Potom.005G13966-SNP77	16	Potom.016G33891-SNP28	Stem volume	AD	-8.40E-01	1.77E-05
10	Potom.010G25398-SNP68	20	Potom.003G08321-SNP4	Holocellulose content	AA	2.51E+01	1.60E-05
5	Potom.005G13966-SNP90	10	Potom.010G25398-SNP68	Stem volume	DD	1.99E+00	1.57E-05
8	Potom.008G21326-SNP69	16	Potom.016G33891-SNP28	Stem volume	AA	-6.07E-01	1.47E-05
5	Pto-Wuschela-SNP12	20	Potom.003G08321-SNP33	Hemicellulose content	DA	-1.86E+01	1.31E-05
5	Potom.005G13966-SNP89	16	Potom.016G34352-SNP109	Stem volume	DD	1.87E+00	1.22E-05
5	Potom.005G13966-SNP90	8	Potom.008G22699-SNP72	Holocellulose content	AA	1.70E+01	1.21E-05
8	Potom.008G22699-SNP72	16	Potom.016G33891-SNP29	Stem volume	AD	8.04E-01	9.59E-06
16	Potom.016G33891-SNP28	20	Potom.003G08321-SNP32	Stem volume	AA	7.68E-01	9.01E-06

2	Potom.002G05522-SNP13	8	Potom.008G22699-SNP71	Holocellulose content	AD	-2.72E+01	8.05E-06
2	Potom.002G05522-SNP14	8	Potom.008G22699-SNP71	Holocellulose content	AD	-2.72E+01	8.05E-06
2	Potom.002G05522-SNP15	5	Potom.005G13966-SNP90	Stem volume	AD	1.14E+00	7.87E-06
2	Potom.002G05769-SNP57	16	Potom.016G33891-SNP17	Fiber width	AD	4.96E+00	7.35E-06
2	Potom.002G05522-SNP12	2	Potom.002G05522-SNP54	Stem volume	AA	-5.29E-01	7.33E-06
2	Potom.002G05769-SNP13	8	Potom.008G22699-SNP72	Stem volume	AD	1.13E+00	6.75E-06
2	Potom.002G07337-SNP229	16	Potom.016G33891-SNP28	Stem volume	AD	-8.45E-01	4.74E-06
2	Potom.002G07206-SNP24	5	Potom.005G13966-SNP89	Stem volume	AD	9.22E-01	3.20E-06
20	Potom.003G08321-SNP9	20	Potom.003G08321-SNP20	Stem volume	DD	-1.48E+00	1.97E-06
2	Potom.002G05522-SNP15	2	Potom.002G05522-SNP54	Stem volume	AA	-7.22E-01	7.78E-07
16	Potom.016G33876-SNP108	16	Potom.016G33891-SNP28	Stem volume	AA	-6.47E-01	4.25E-07
